# Impact of enlarged perivascular spaces on endovascular therapy outcomes in patients with large ischaemic core: A post-hoc analysis of the ANGEL-ASPECT trial

**DOI:** 10.1515/jtim-2026-0036

**Published:** 2026-03-26

**Authors:** Zan Wang, Chenhui Liu, Mengxing Wang, Shuning Cai, Ximing Nie, Liping Liu, Xiaochuan Huo, Yuesong Pan, Zhongrong Miao, Yilong Wang, The ANGEL-ASPECT Study Group

**Affiliations:** Department of Neurology, Beijing Tiantan Hospital, Capital Medical University, Beijing, China; China National Clinical Research Center for Neurological Diseases, Beijing, China; Department of Neurology, The first Affiliated Hospital of Zhengzhou University, Zhengzhou, Henan Province, China; Cerebrovascular Disease Department, Neurological Disease Centre, Beijing Anzhen Hospital, Capital Medical University, Beijing, China; Intervenrional Neuroradiology, Department of Neurology, Beijing Tiantan Hospital, Capital Medical University, Beijing, China; Chinese Institute for Brain Research, Beijing, China; National Center for Neurological Disorders, Beijing, China; Beijing Laboratory of Oral Health, Capital Medical University, Beijing, China; Laboratory for Clinical Medicine, Capital Medical University, Beijing, China; Advanced Innovation Center for Human Brian Protection, Capital Medical University, Beijing, China

**Keywords:** acute ischaemic stroke, endovascular therapy, enlarged perivascular spaces, large vessel occlusion, large infarct core, post-hoc analysis

## Abstract

**Background and Objectives:**

Enlarged perivascular spaces (EPVSs), particularly in the basal ganglia (BG), are indicators of microvascular dysfunction and impaired glymphatic clearance, which may influence stroke outcomes. Determining the threshold below which endovascular therapy (EVT) confers no additional benefit is clinically important for patients with large ischaemic infarcts.

**Methods:**

This post-hoc analysis utilized data from the ANGEL-ASPECT trial (NCT04551664), a multicentre, randomized controlled trial of 456 acute ischaemic stroke (AIS) patients with anterior circulation large vessel occlusion (LVO) and a large ischaemic core. Among these patients, 226 who with completed, high-quality brain magnetic resonance imaging (MRI) were included. BG-EPVS severity was assessed on T2-weighted MRI and categorized as none-to-mild, moderate, or severe. The primary outcome was the 90-day modified Rankin scale (mRS) score.

**Results:**

EVT significantly improved 90-day mRS outcome in patients with none-to-mild (adjusted common odds ratio [cOR] 5.50, 95% CI: 2.72–11.16, *P* < 0.001) and moderate (adjusted cOR 4.03, 95% CI, 1.46–11.15, *P* = 0.007) BG-EPVS. However, the benefit was markedly attenuated and not statistically significant in patients with severe BG-EPVS (adjusted cOR, 1.07 95% CI, 0.25–4.67, *P* = 0.926). EVT also increased the likelihood of achieving favourable functional outcomes (mRS scores of 0–2 and 0–3) and early neurological improvement (ENI) in the none-to-mild BG-EPVS subgroup, and favourable outcome (mRS score of 0–2) in the moderate BG-EPVS subgroup, but not in the severe BG-EPVS subgroup. Significant treatment–by–BG-EPVS interactions were observed for achieving an mRS score of 0–3 (*P*_interaction = 0.005) and ENI (*P*_interaction = 0.029).

**Conclusions:**

EVT was associated with significantly improved 90-day functional outcomes in LVO-AIS patients with a large ischaemic core and none-to-mild or moderate BG-EPVS, whereas this benefit was not observed in those with severe BG-EPVS. Given the limited power in subgroup analyses, these findings should be considered hypothesis-generating and warrant validation in larger, adequately powered randomized controlled trials.

## Introduction

Endovascular therapy (EVT) is currently recommended as the standard treatment for acute ischaemic stroke (AIS) due to large vessel occlusion (LVO) in the anterior circulation.^[1]^ Recent randomized controlled trials (RCTs) have demonstrated that, compared with medical management alone, EVT yields better functional outcomes, even in LVO-AIS patients with a large ischaemic core.^[2, 3, 4, 5, 6, 7]^ However, a substantial proportion of patients still experience poor functional outcomes at 90-days despite successful recanalization.^[8]^ Determining the threshold below which EVT does not confer additional benefits to patients with a large ischaemic infarct is crucial for optimizing patient selection.

Recent studies have shown that cerebral small vessel disease (CSVD) is more prevalent in AIS patients than in the general population, and that a severe CSVD burden is associated with an increased risk of symptomatic intracranial haemorrhage (ICH) and poor outcomes in LVO-AIS patients following EVT.^[9, 10, 11]^ Prevalent CSVD markers, such as white matter hyperintensity (WMH), lacunes and cerebral microbleeds (CMBs), have also been linked to unfavourable post-EVT outcomes.^[8,12,13,14]^ Another marker, enlarged perivascular spaces (EPVSs), particularly those located in the basal ganglia (BG), has been identified as a reliable predictor of increased risk for incident ischaemic stroke, poor functional outcomes, stroke recurrence, and poststroke cognitive impairment in stroke survivors.^[15, 16, 17]^ However, the association between BG-EPVS and outcomes following EVT has not been well explored.

Perivascular spaces (PVSs) are physiological spaces surrounding small vessel walls as they traverse the brain parenchyma from the subarachnoid space.^[18]^ EPVS visible on brain magnetic resonance imaging (MRI) are considered indicative of PVS dysfunction, reflecting impaired clearance of interstitial fluid and metabolic waste from brain tissue.^[19]^ In AIS patients with a high degree of EPVS, ineffective interstitial fluid drainage and the accumulation of inflammatory cytokines can exacerbate the inflammatory response.^[20]^ This cascade can, in turn, lead to increased blood–brain barrier (BBB) permeability, worsening cerebral oedema, and expansion of the ischaemic lesion.^[21, 22, 23]^ Moreover, the number of EPVS, particularly in the BG, increases with age and vascular risk factors.^[24]^ A greater BG-EPVS burden has been associated with reduced cerebrovascular reactivity ^[25]^ and more severe underlying microvascular lesions.^[26,27]^ Based on these findings, we hypothesize that a higher degree of EVPS, especially in the BG, is associated with less favourable functional outcomes in LVO-AIS patients undergoing EVT. If validated, this marker may aid in identifying patients with large infarcts who are most likely to benefit from EVT.

More importantly, it remains unclear whether EVT is safe and effective in patients with large infarctions who also have a high degree of BG-EPVS. Therefore, we further compared the outcomes of EVT versus medical management across patients with none-to-mild, moderate, and severe BG-EPVS, based on a post-hoc analysis of the Endovascular Therapy in Acute Anterior Circulation Large Vessel Occlusive Patients with a Large Infarct Core (ANGEL-ASPECT) trial.^[4]^ Additionally, given that EPVS in the BG and centrum semiovale (CSO) may reflect distinct pathophysiological processes,^[28]^ a supplementary analysis was performed to evaluate the efficacy and safety of EVT in patients with anterior-circulation LVO and large infarctions, further stratified by CSO-EPVS severity.

## Materials and methods

### Study population

ANGEL-ASPECT (NCT04551664) is a multicentre, prospective, randomized, open-label, blinded endpoint clinical trial conducted at 46 comprehensive stroke centres across China.^[4]^ From October 2020 to May 2022, a total of 456 LVO-AIS patients were enrolled and randomly assigned (1: 1) to receive EVT plus medical management (MM) or MM alone. The trial was approved by the institutional review boards at Beijing Tiantian Hospital (IRB approval number: No. KY2020–072–02) and each participating site (Supplementary Table S1). Written informed consent was obtained from all patients or their representatives prior to enrolment. The detailed study protocol has been reported previously^[4,29,30]^ and is available in Supplement 1.

### Data collection

Baseline characteristics, including age, sex, baseline National Institutes of Health Stroke Scale (NIHSS) score, medical history, blood pressure, laboratory test results, stroke aetiology, intravenous thrombolysis, wake-up stroke, and time from symptom onset, were recorded.

An independent imaging core laboratory adjudicated the baseline Alberta Stroke Program Early Computed Tomography Score (ASPECTS), site of LVO, reperfusion status, target recanalization level at 36 h, and the presence of ICH. Infarct core volume was assessed using RAPID software and defined as the area with a relative cerebral blood flow of less than 30% of normal tissue on computed tomography (CT) perfusion or an apparent diffusion coefficient value of less than 620×10^–6^ mm^2^/s on MRI. At 36 h, the recanalization status in both the EVT and MM groups was assessed based on the modified arterial occlusive lesion grade on follow-up CT or MR angiography.

### Quantification of MRI-Visible BG-EPVS

All included patients underwent brain MRI (1.5T or 3.0T) either upon admission or, on average, two days after symptom onset. EPVSs were assessed on axial T2-weighted images in accordance with the STandars for ReportIng Vascular changes on nEuroimaging (STRIVE) criteria,^[31]^ by a trained rater (C. L.) who was blinded to the clinical information and treatment allocation. EPVS in the BG were rated using a validated 5-point visual rating scale (0, no EPVS; 1, < 10 EPVS; 2, 11–20 EPVS; 3, 21–40 EPVS; and 4, > 40 EPVS).^[28]^ BG-EPVS were evaluated only in the hemisphere contralateral to the site of the known LVO. Based on the severity of BG-EPVS, patients were categorized into three subgroups: none-to-mild (rating scale 0–1), moderate (scale 2), and severe (scales 3–4) (Supplementary Figure S1).^[27,28]^

To ensure reliability and minimize potential measurement bias, a randomly selected subset of 100 T2-weighted MR images was independently graded by two trained raters (C. L. and Z. W.), who were both blinded to all clinical and outcome data. Interrater agreement was quantified using both the proportion of exact agreement and Cohen’s weighted *kappa* statistic. Interrater agreement for BG-EPVS was substantial (weighted *kappa* = 0.77, 86.3% exact agreement). Discrepancies were adjudicated by a senior radiologist to reach a consensus. After confirming high interrater reliability, the full cohort was rated by a single trained rater (C. L.) who was blinded to all clinical and outcome information, to maximize internal consistency and reduce intrastudy heterogeneity.

### Outcome assessment

The primary outcome was the 90-day modified Rankin scale (mRS) score. Secondary outcomes included the proportion of patients who achieved an mRS score of 0–2 or 0–3; early neurological improvement (ENI), defined as an NIHSS score of 0 or an improvement of ≥10 points at 36 h; and a change in infarct volume, defined as the difference between baseline imaging (CT perfusion or diffusion-weighted MRI) and follow-up noncontrast CT at 7 days or at discharge, or MRI at 36 h; and target artery recanalization at 36 h, defined as a modified arterial occlusive lesion grade of 2–3. Safety outcomes included symptomatic ICH within 48 h, assessed according to the Heidelberg Bleeding Classification; any ICH; death (mRS score of 6) within 90 days; and decompressive hemicraniectomy during hospitalization.

### Statistical analysis

Continuous and ordinal variables are reported as medians with interquartile ranges (IQRs), and categorical variables are presented as counts and percentages. The Pearson *χ*^2^ test or Fisher’s exact test was used for comparisons of categorical variables, and the Kruskal–Wallis test was applied for continuous variables.

To evaluate outcomes between the EVT and MM groups within each of the three BG-EPVS strata, an ordinal logistic regression model was used to calculate the common odds ratio (cOR) with 95% confidence intervals (CIs) for the ordinal shift in 90-day mRS scores. A Cox proportional hazards regression model was used to estimate the hazard ratio (HR) with 95% CIs for 90-day mortality. A general linear model was used to calculate mean differences with 95% CIs for changes in infarct core volume. Binary logistic regression models were used to estimate odds ratios (ORs) with 95% CIs for other secondary and safety outcomes. The interaction effect between the treatment (EVT *vs*. MM) and BG-EPVS subgroups was assessed by including a multiplicative interaction term in each model. Additionally, all analyses were repeated after adjustment for baseline characteristics that were unbalanced between the EVT and MM groups within each BG-EPVS stratum, as well as for differences in baseline characteristics between included and excluded patients (Model 2), as detailed in Supplementary Table S2.

All the statistical analyses were performed using SAS software (version 9.4), and a two-tailed *P* value < 0.05 was considered to indicate statistical significance.

### Supplementary analyses: Modification of the treatment eEffect of EVT by CSO-EPVS severity

We divided all included patients into three CSO-EPVS subgroups according the severity of CSO-EPVS: none-to-mild (rating scale 0–1), moderate (scale 2) and severe (scales 3–4) (Supplementary Table S3). For CSO-EPVS, interrater agreement was almost perfect (weighted *kappa* = 0.83, 88.2% exact agreement). All regression models were adjusted for baseline characteristics that were unbalanced between the EVT and MM groups within each CSO-EPVS stratum, as well as for differences in baseline characteristics between included and excluded patients (Model 2), as detailed in Supplementary Table S4.

## Results

### Subgroup population for the BG-EPVS study

**Figure 1:**
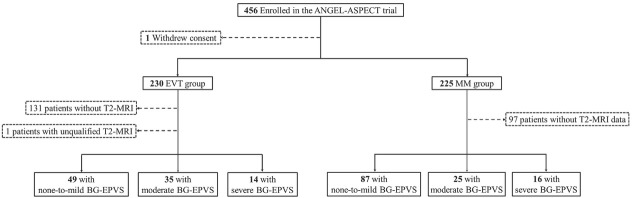
Flowchart of patient selection for the post-hoc analysis. ANGEL-ASPECT, indicates endovascular therapy in acute anterior circulation large vessel occlusive patients with a large infarct core; BG-EPVS: enlarged perivascular spaces in the basal ganglia; EVT: endovascular therapy; MM: medical management.

Among the 226 included patients, 136 (60.2%) had none-to-mild BG-EPVS (MM group: *n* = 87; EVT group: *n* = 49), 60 (26.5%) had moderate BG-EPVS (MM group: *n* = 25; EVT group: *n* = 35), and 30 (13.3%) had severe BG-EPVS (MM group: *n* = 16; EVT group: *n* = 14). A detailed flowchart of patient selection is presented in Figure 1, and the baseline demographic and clinical characteristics are summarized in Table 1 and Supplementary Table S5.

**Table 1:** Baseline demographic and clinical characteristics of patients in the ANGEL-ASPECT trial, stratified by BG-EPVS severity

Baseline characteristics	None-to-mild (*n* = 136)	Moderate (*n* = 60)	Severe (*n* = 30)	*P* value
Age, median (IQR), years	64 (56-71)	69 (64-74)	72 (65-77)	< 0.001
Male Sex, *n* (%)	99 (72.8)	34 (56.7)	20 (66.7)	0.083
Hypertension, *n* (%)	74 (54.4)	39 (65.0)	19 (63.3)	0.322
Diabetes mellitus, *n* (%)	16 (11.8)	16 (26.7)	4 (13.3)	0.029
Hyperlipidemia, *n* (%)	5 (3.7)	4 (6.7)	1 (3.3)	0.613
Atrial fibrillation, *n* (%)	24 (17.7)	11 (18.3)	6 (20.0)	0.954
Coronary heart disease, *n* (%)	21 (15.4)	13 (21.7)	3 (10.0)	0.332
Ischemic stroke, *n* (%)	16 (11.8)	14 (23.3)	6 (20.0)	0.101
Cigarette smoking, *n* (%)	53 (39.0)	18 (30.0)	9 (30.0)	0.386
Wake-up Stroke, *n* (%)	44 (32.4)	20 (33.3)	10 (33.3)	0.988
SBP, median (IQR), mmHg	148 (127–163)	147 (129–167)	155 (141–171)	0.194
DBP, median (IQR), mmHg	84 (77–93)	85 (73–93)	89 (77–100)	0.482
Baseline NIHSS, median (IQR)	15 (12–18)	16 (13–19)	17 (12–20)	0.340
ASPECTS, median (IQR)	3 (3–4)	3 (3–4)	3 (3–5)	0.571
Occlusion site, *n* (%)				0.455
ICA	51 (37.5)	20 (33.3)	6 (20.0)	
M1	83 (61.0)	39 (65.0)	23 (76.7)	
M2	2 (1.5)	1 (1.7)	1 (3.3)	
Ipsilateral extracranial ICAO, *n* (%)	27 (19.9)	10 (16.7)	2 (6.7)	0.222
Intravenous thrombolysis, *n* (%)	41 (30.2)	17 (28.3)	10 (33.3)	0.888
Stroke classification, *n* (%)				0.360
Atherothrombotic	32 (23.5)	22 (36.7)	7 (23.3)	
Cardioembolic	56 (41.2)	22 (36.7)	14 (46.7)	
Undetermined or other	48 (35.3)	16 (26.7)	9 (30.0)	
Infarct core volume, median (IQR), mL	62 (38–81)	62 (42–80)	68 (42–94)	0.637
Time from onset to door, median (IQR), min	367 (205–659)	311 (209–649)	310 (127–611)	0.661
Time from onset to imaging, median (IQR), min	439 (276–748)	429 (268–704)	352 (190–716)	0.549
Time from onset to randomization, median (IQR), min	480 (321–784)	486 (305–758)	392 (239–762)	0.506

ASPECTS: Alberta Stroke Program early CT Scores; BG-EPVS: enlarged perivascular spaces (EPVS) in the basal ganglia (BG); DBP: diastolic blood pressure; EVT: endovascular therapy; IQR: interquartile range; ICA: internal carotid artery; ICAO: internal carotid artery occlusion; M1: main trunk of the middle cerebral artery; M2 segment: first-order branch of the main trunk; MM: medical management; mRS: modified Rankin Scale; NIHSS: National Institutes of Health Stroke Scale; SBP: systolic blood pressure.

### Relationships between BG-EPVS Severity and functional outcomes and safety endpoints among EVT patients

As shown in Supplementary Table S6, univariate regression analyses revealed that, compared with patients with none-to-mild BG-EPVS, those with moderate or severe BG-EPVS had worse 90-day mRS scores, lower rates of achieving an mRS score of 0–2, and lower rates of achieving an mRS score of 0–3.

After adjusting for age, sex, baseline NIHSS score, ASPECTS, intravenous thrombolysis, and time from onset to randomization, severe BG-EPVS remained associated with worse 90-day mRS scores (adjusted cOR 0.34, 95% CI: 0.11–1.04, *P =* 0.058) and lower odds of achieving an mRS score of 0–2 (adjusted OR 0.23, 95% CI: 0.05–1.03, *P =* 0.055), although these associations did not reach the predefined threshold for statistical significance. However, both moderate and severe BG-EPVS remained significantly associated with lower rates of achieving an mRS score of 0–3 (adjusted OR 0.32, 95% CI: 0.11–0.95, *P =* 0.040; adjusted OR 0.12, 95% CI: 0.03–0.59, *P =* 0.009) after controlling for the aforementioned confounders. With regard to safety outcomes, compared with patients with none-to-mild BG-EPVS, patients with moderate or severe BG-EPVS did not have a significantly increased risk of symptomatic ICH, any ICH within 48 h, or 90-day mortality (all adjusted *P* > 0.05).

### Modification of the effect of EVT treatment on functional outcomes and safety endpoints by BG-EPVS severity

**Figure 2:**
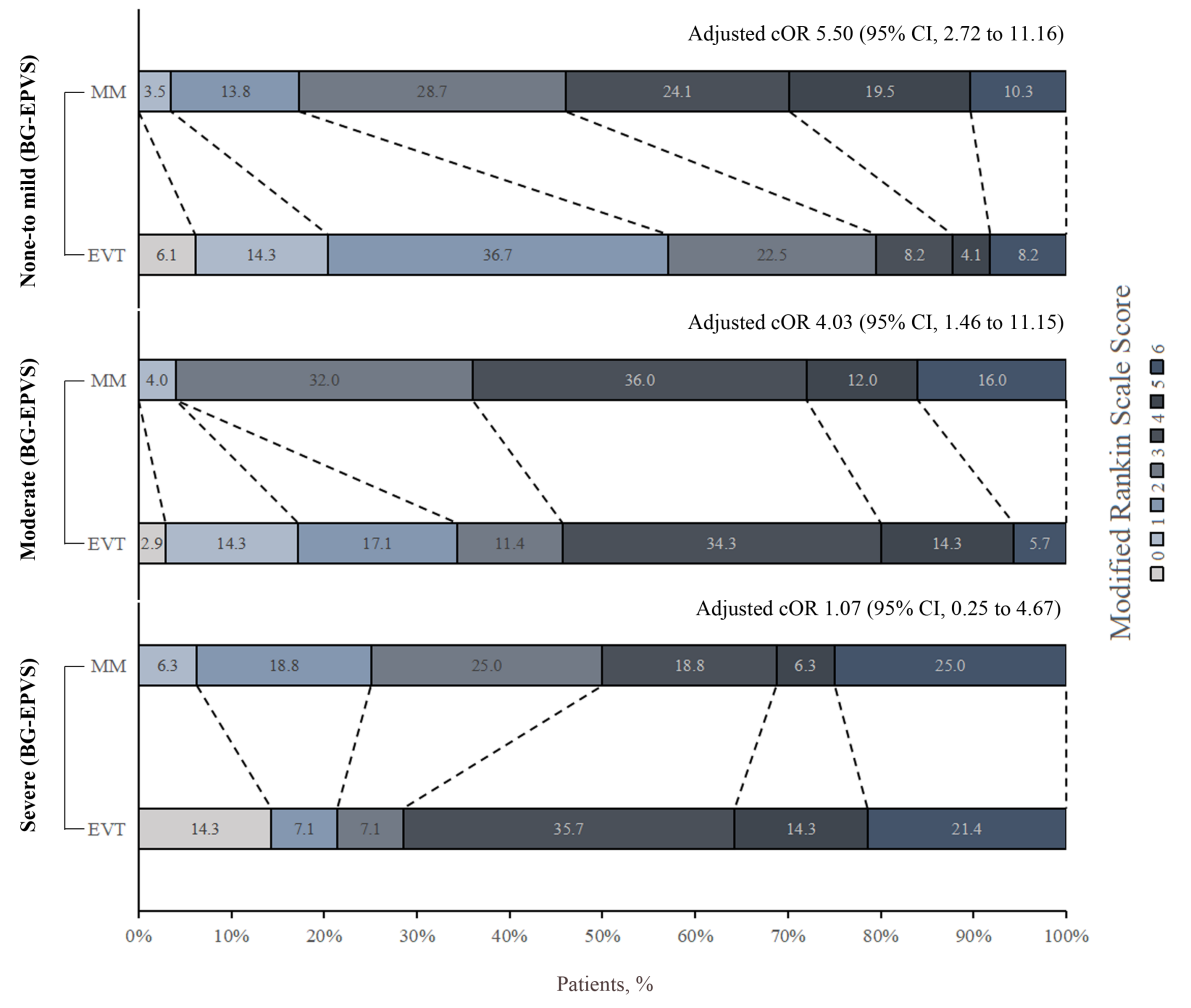
Distribution of the modified Rankin scale at 90 days across three subgroups stratified by BG-EPVS severity. BG-EPVS: enlarged perivascular spaces in the basal ganglia; EVT: endovascular therapy; MM: medical management.

In none-to-mild BG-EPVS subgroup, a shift in the distribution of 90-day mRS scores towards better outcome was observed in the EVT group than in the MM group (adjusted cOR 5.50, 95% CI: 2.72–11.16, *P <* 0.001). A similar significant shift was noted in the moderate BG-EPVS subgroup (adjusted cOR 4.03, 95% CI: 1.46–11.15, *P =* 0.007), but not in the severe BG-EPVS subgroup (adjusted cOR 1.07, 95% CI: 0.25–4.67, *P =* 0.926). A marginal interaction effect across the three BG-EPVS strata was observed (*P* = 0.052) (Table 2 and Figure 2).

**Table 2:** Modification of treatment effect of EVT by BG-EPVS severity

Outcome	MM group (*n* = 1 28)a	EVT group (*n* = 98)^a^	Model 1: Unadjusted Effect size (95% Cl)^b^	*P* value	*P* value (Interaction)	Model 2: Adjusted Effect size (95% Cl)^b,c^	*P* value	*P* value (Interaction)
Primary outcome								
mRS score, median (IQR)					0.009			0.052
None-to-mild (*n* = 136)	4 (3-5)	2 (2-3)	5.20 (2.64 to 10.25)	<0.001		5.50 (2.72 to 11.16)	<0.001	
Moderate (*n* = 60)	4 (3-5)	4 (2-4)	2.12 (0.84 to 5.40)	0.1 14		4.03 (1.46 to 11.15)	0.007	
Severe (*n* = 30)	4 (3-6)	4 (3-5)	0.77 (0.22 to 2.76)	0.692		1.07 (0.25 to 4.67)	0.926	
Secondary outcomes								
mRS score 0-2, *n* (%)					0.068			0.068
None-to-mild (*n* = 136)	15 (17.2)	28 (57.1)	6.40 (2.90 to 14.15)	<0.001		7.83 (3.22 to 19.06)	<0.001	
Moderate (*n* = 60)	1 (4.0)	12 (34.3)	12.52 (1.51 to 104.12)	0.019		25.89 (2.31 to 290.55)	0.008	
Severe (*n* = 30)	4 (25.0)	3 (21.4)	0.82 (0.15 to 4.51)	0.818		0.78 (0.06 to 9.51)	0.844	
mRS score 0-3, *n* (%)					0.004			0.005
None-to-mild (*n* = 136)	40 (46.0)	39 (79.6)	4.58 (2.03 to 10.33)	<0.001		5.86 (2.27 to 15.14)	<0.001	
Moderate (*n* = 60)	9 (36.0)	16 (45.7)	1.50 (0.52 to 4.29)	0.453		2.66 (0.67 to 10.56)	0.165	
Severe (*n* = 30)	8 (50.0)	4 (28.6)	0.40 (0.09 to 1.83)	0.237		0.43 (0.06 to 3.21)	0.409	
ENI, *n* (%)					0.034			0.029
None-to-mild (*n* = 136)	2 (2.3)	9 (18.4)	9.56 (1.97 to 46.31)	0.005		8.14 (1.59 to 41.78)	0.012	
Moderate (*n* = 60)	1 (4.2)	2 (5.7)	1.39 (0.12 to 16.29)	0.791		4.29 (0.01 to >999.999)	0.645	
Severe (*n* = 30)	1 (6.3)	0 (0.0)	NA	0.961		NA	0.976	
Change in ICV from baseline to follow-up, median (IQR), mL					0.478			0.508
None-to-mild (*n* = 136)	90.7 (28.9-141.2)	48.7 (10.9-86.3)	-33.29 (-60.52 to -6.06)	0.017		-30.73 (-59.12 to -2.34)	0.034	
Moderate (*n* = 60)	92.8 (37.5-152.4)	50.7 (30.4-98.5)	-35.65 (-76.17 to 4.88)	0.084		-31.15 (-75.16 to 12.85)	0.161	
Severe (*n* = 30)	62.7 (22.8-168.3)	63.4 (8.4-149.8)	-3.41 (-70.73 to 63.90)	0.918		16.57 (-58.93 to 92.06)	0.654	
Target artery recanalization at 36 h, *n* (%)					0.940			0.784
None-to-mild (*n* = 136)	30 (37.0)	40 (83.3)	8.50 (3.52 to 20.55)	<0.001		7.17 (2.90 to 17.75)	<0.001	
Moderate (*n* = 60)	9 (39.1)	30 (88.2)	1 1.67 (3.06 to 44.46)	<0.001		17.19 (3.32 to 89.06)	0.001	
Severe (*n* = 30)	5 (31.3)	1 1 (78.6)	8.07 (1.54 to 42.32)	0.014		54.85 (2.08 to >999.999)	0.016	
Safety								
SICH within 48h, *n* (%)					0.690			0.875
None-to-mild (*n* = 136)	1 (1.2)	1 (2.0)	1.79 (0.1 1 to 29.29)	0.683		4.74 (0.13 to 173.82)	0.398	
Moderate (*n* = 60)	1 (4.0)	0 (0.0)	NA	0.955		NA	0.886	
Severe (*n* = 30)	0 (0.0)	1 (7.1)	NA	0.958		NA	0.867	
Any ICH within 48h, *n* (%)					0.783			0.836
None-to-mild (*n* = 136)	1 1 (12.6)	14 (28.6)	2.76 (1.14 to 6.70)	0.024		2.55 (1.01 to 6.49)	0.049	
Moderate (*n* = 60)	4 (16.0)	12 (34.3)	2.74 (0.76 to 9.82)	0.122		2.14 (0.53 to 8.61)	0.282	
Severe (*n* = 30)	2 (12.5)	5 (35.7)	3.89 (0.62 to 24.52)	0.148		1 1.25 (0.92 to 138.28)	0.059	
Death within 90-day, *n* (%)					0.963			0.824
None-to-mild (*n* = 136)	9 (10.3)	4 (8.2)	0.77 (0.24 to 2.49)	0.657		0.93 (0.26 to 3.34)	0.914	
Moderate (*n* = 60)	4 (16.0)	2 (5.7)	0.33 (0.06 to 1.77)	0.194		0.17 (0.02 to 1.17)	0.071	
Severe (*n* = 30)	4 (25.0)	3 (21.4)	0.82 (0.18 to 3.67)	0.796		0.79 (0.14 to 4.41)	0.789	
Decompressive hemicraniectomy during hospitalization, *n* (%)					0.940			0.949
None-to-mild (*n* = 136)	2 (2.3)	1 (2.0)	0.89 (0.08 to 10.02)	0.922		0.57 (0.04 to 9.56)	0.699	
Moderate (*n* = 60)	0 (0.0)	0 (0.0)	NA	NA		NA	NA	
Severe (*n* = 30)	0 (0.0)	1 (7.1)	NA	0.958		NA	0.964	

a Data are presented as number (percentage) of patients for categorical values and median (IQR) for continuous or ordinal variables.

b Treatment effects are reported as generalized odds ratio (95% Cl) for the ordinal shift across the range of mRS scores toward a better outcome by the ordinal logistic regression model (primary outcome), hazard ratio (95% Cl) for death by a Cox proportional hazards regression model, mean difference (95% Cl) for ICV change by the general linear model, and odds ratio (OR) with the corresponding 95% CIs for other outcomes by the binary logistic regression models.

c Model 1: Unadjusted. BG-EPVS: enlarged perivascular spaces (EPVS) in the basal ganglia (BG); ENI: early neurological improvement; EVT: endovascular therapy; ICH: intracranial hemorrhage; ICV: infarct core volume; MM: medical management; mRS: modified Rankin Scale; NA: not applicable; SICH: symptomatic intracranial hemorrhage.

In terms of secondary outcomes, compared with MM, EVT was associated with significantly greater odds of achieving an mRS score of 0–2 in both the none-to-mild (adjusted OR 7.83, 95% CI: 3.22–19.06, *P <* 0.001) and moderate (adjusted OR 25.89, 95% CI: 2.31–290.55, *P =* 0.008) BG-EPVS subgroups but not in the severe BG-EPVS subgroup (adjusted OR 0.78, 95% CI: 0.06–9.51, *P* = 0.844). A marginal treatment–by–BG-EPVS interaction was observed for this outcome (*P =* 0.068) (Table 2). Additionally, EVT was associated with significantly higher rates of achieving an mRS score of 0–3 (adjusted OR 5.86, 95% CI: 2.27–15.14, *P <* 0.001) and ENI (adjusted OR 8.14, 95% CI: 1.59–41.78, *P =* 0.012) in the none-to-mild BG-EPVS subgroup but not in the moderate or severe BG-EPVS subgroup. Significant treatment–by–BG-EPVS interactions were detected for patients who achieved both an mRS score of 0–3 (*P =* 0.005) and an ENI (*P =* 0.029) (Table 2).

With respect to infarct growth, compared with MM, EVT was associated with a significantly smaller increase in infarct core volume in the none-to-mild BG-EPVS subgroup (mean difference-33.29, 95% CI-60.52 to-6.06, *P =* 0.017) but not in the moderate (mean difference-35.65, 95% CI-76.17 to 4.88, *P =* 0.084) or severe (mean difference-3.41, 95% CI-70.73 to 63.90, *P =* 0.918) BG-EPVS subgroups (Table 2).

With respect to safety outcomes, compared with MM, EVT was associated with a slightly greater rate of any ICH within 48 h in the none-to-mild BG-EPVS subgroup (adjusted OR 2.55, 95% CI: 1.01–6.49, *P =* 0.049), but not in the moderate (adjusted OR 2.14, 95% CI: 0.53–8.61, *P =* 0.282) or severe (adjusted OR 11.25, 95% CI: 0.92–138.28, *P =* 0.059) BG-EPVS subgroups. No significant differences were observed in the rates of symptomatic ICH or mortality between the EVT and MM groups across the three BG-EPVS strata (Table 2).

### Sensitivity analyses

Further adjustment for additional CSVD imaging markers, including lacunes and periventricular and deep WMH, yielded results that were highly consistent with those of the primary analyses (Supplementary Table S7), indicating that the observed modifying effect of BG-EPVS severity on post-EVT outcomes were not fully explained by other CSVD markers.

In additional sensitivity analyses that further adjusted for study centre, MRI field strength, and slice thickness, the effect estimates for EVT and the treatment–by–BG-EPVS interactions remained similar to those of the primary models, and none of the main conclusions was materially altered (Supplementary Table S8).

### Supplementary analyses: modification of EVT treatment effect by CSO-EPVS severity

Univariate and multivariate regression analyses indicated that CSO-EPVS severity was not significantly associated with functional outcomes or safety endpoints among patients treated with EVT (all *P >* 0.05) (Supplementary Table S9).

Furthermore, across all three CSO-EPVS subgroups, a favourable shift in the distribution of 90-day mRS scores was consistently observed in the EVT group compared with the MM group (all adjusted *P <* 0.05) (Supplementary Figure S2, Supplementary Table S10–12). Detailed results for the secondary and safety outcomes are provided in Supplement 2.

## Discussion

Current evidence from published RCTs demonstrates that compared with medical management alone, EVT yields superior functional outcomes, even in anterior circulation LVO patients with large infarctions.^[3, 4, 5, 6, 7]^ However, the issue of futile recanalization remains a significant challenge, underscoring the need for more precise stratification to identify patients most likely to benefit from EVT and thereby optimize outcomes in LVO-AIS patients. In this context, we conducted a post-hoc analysis of the ANGEL-ASPECT trial, focusing on the influence of BG-EPVS severity on both functional and safety outcomes in LVO-AIS patients with large ischaemic cores who underwent EVT.

Our results suggested that EVT was associated with significantly improved 90-day functional outcomes in patients with none-to-mild and moderate BG-EPVS, whereas this benefit was not observed in patients with severe BG-EPVS. Secondary outcome analyses further supported this trend, showing that EVT was associated with higher rates of achieving favourable outcomes (*e.g*., mRS scores of 0–2 and 0–3) and early neurological improvement among patients with lower BG-EPVS burdens. Nevertheless, these results should be interpreted with caution. The absence of significant benefit observed in the severe BG-EPVS subgroup may be attributable to the limited sample size (*n* = 30) and reduced statistical power, rather than definitive evidence of no treatment effect. Thus, while our findings raise concerns regarding the potential impact of severe BG-EPVS on EVT efficacy, BG-EPVS severity alone may not be sufficient as an absolute contraindication to EVT in clinical decision-making. Taken together, our results indicate that while EVT confers substantial benefit in patients with none-to-mild and moderate BG-EPVS, its effectiveness appears less certain in those with severe BG-EPVS. The observed trend towards diminished benefit in this subgroup requires further validation. Given the exploratory nature of this post-hoc analysis and the inherent limitations in sample size, these results should be considered hypothesis-generating. Further studies with larger cohorts or pooled analyses from randomized trials are be needed to clarify the clinical implications of BG-EPVS severity for optimizing EVT selection in patients with LVO-AIS.

In this study, we observed that the benefit of EVT in LVO-AIS patients with a large ischaemic core and severe BG-EPVS burden was attenuated compared to those with none-to-mild or moderate BG-EPVS. We hypothesize that this reduction in EVT benefit may be related to underlying cerebrovascular dysfunction and impaired brain-fluid dynamics. Specifically, BG-EPVS has been shown to increase with vascular risk factors^[24]^ and has been associated with microvascular damage and impaired cerebrovascular reactivity^[23,25]^ —both critical determinants of the brain’s resilience to ischaemic injury and its capacity for recovery. Consistent with previous reports, we found that patients with moderate and severe BG-EPVS burden had greater deep and periventricular WMH burden, as well as a higher prevalence of lacunes, compared with those with none-to-mild BG-EPVS (Supplementary Table S13). These findings support the notion that greater BG-EPVS burden is closely related to more severe microcirculatory dysfunction.^[32, 33, 34]^ Recent studies have further demonstrated that BG-EPVS severity is associated with compromised BBB integrity. A study by Li *et al*.^[23]^ found that BG-EPVS severity was correlated with increased BBB permeability, reinforcing the idea that BG-EPVS may reflect underlying BBB dysfunction. Another plausible explanation for the attenuated EVT benefit in patients with severe BG-EPVS is impaired glymphatic clearance. EPVS visible on brain MRI are considered indicators of perivascular space dysfunction, which has been linked to impaired clearance of interstitial fluid and metabolic waste from brain tissue.^[19,35]^ To provide additional supportive evidence, we performed an ancillary analysis in an independent community-based cohort (PRECISE,^[36]^ *n* = 2219; mean age 61.3 ± 6.9 years, 45.9% male) and found that higher BG-EPVS scores were significantly associated with a lower diffusion tensor image analysis along the perivascular space (DTI-ALPS) index^[37]^ (Supplementary Table S14), which has been proposed as a noninvasive approach to evaluate glymphatic system function *in vivo* (with lower values suggesting impaired glymphatic clearance). Taken together, in patients with severe BG-EPVS burden, these processes may be more pronounced, thereby potentially contributing to persistent neuroinflammation,^[38,39]^ vasogenic oedema, and expansion of the infarct core.^[20,21,40]^ However, while our findings align with these hypotheses, our study cannot directly test these mechanisms, as we did not obtain advanced MRI sequences or biomarkers to assess glymphatic function or BBB integrity in this post-hoc analysis. Therefore, these proposed mechanisms should be regarded as hypothesis-generating and require confirmation in future studies. To validate these hypotheses, future research should incorporate advanced imaging techniques and biomarkers—for example, noninvasive glymphatic MRI approaches (*e.g*., the DTI-ALPS index) and dynamic contrast-enhanced MRI (DCE-MRI) to quantitatively assess glymphatic system function and BBB permeability, respectively. These approaches are crucial for testing these hypotheses and further validating the role of BG-EPVS burden in modifying EVT outcomes.

Additionally, our supplementary analyses provide valuable insights into how EPVS severity in different brain regions may differentially influence EVT outcomes. Specifically, we found that the modifying effect of CSO-EPVS severity on EVT outcomes was much less pronounced than that of BG-EPVS. These regional disparities may be attributed to distinct vascular pathophysiological mechanisms and anatomical characteristics between the BG-EPVS and CSO-EPVS.^[21,27,41]^ Our supplementary results (Supplementary Table S13) further illustrate these differences: in the BG-EPVS subgroups, both deep and periventricular WMH burden—particularly deep WMH—demonstrated a clear increasing trend with increasing BG-EPVS severity, indicating a strong association between greater BG-EPVS burden and more severe microcirculation dysfunction. In contrast, the prevalence of lacunes in the BG-EPVS subgroups did not increase stepwise. However, for the CSO-EPVS subgroups, the prevalence of lacunes increased steadily with increasing CSO-EPVS burden, while the associations with WMH burden were less pronounced. Collectively, these findings suggest that BG-EPVS is closely linked to more severe microvascular injury in our cohort. Prior studies have further demonstrated that BG-EPVS is associated with deep perforating artery arteriolosclerosis, impaired cerebrovascular reactivity and BBB disruption.^[21,23,27,33]^ Importantly, a higher BG-EPVS burden is also particularly associated with more severe glymphatic dysfunction. Around the cortical arteries at the CSO, the PVS is composed of monolayer pia mater; around the cortical arteries at the BG, the PVS is composed of double-layered pia mater.^[42,43]^ It has been speculated that this anatomical variability may account for differences in clearance efficiency along these drainage pathways.^[41]^ Thus, LOV-AIS patients with a greater burden of BG-EPVS may exhibit more pronounced impairment of glymphatic clearance and aggravated neuroinflammation.^[38,40]^ Together, these mechanisms may help explain the attenuated benefit of EVT observed in patients with severe BG-EPVS. In contrast, CSO-EPVS is more frequently associated with age-related degeneration and less severe microvascular damage, potentially reflecting cerebral amyloid angiopathy.^[27]^ Additionally, the simpler perivascular structure of CSO-EPVS may be associated with less pronounced impairment of waste clearance, which could partly explain the more limited influence of CSO-EPVS severity on EVT outcomes observed in our study.

With regard to safety outcomes, EVT was associated with a borderline increased risk of any ICH within 48 h in the none-to-mild BG-EPVS subgroup, and a nonsignificant trend towards increased risk in the severe BG-EPVS subgroup, but not in the moderate BG-EPVS subgroup. Importantly, there were no significant differences in the rates of symptomatic ICH or mortality between the EVT and medical management groups across any of the BG-EPVS strata. These findings suggest that LVO-AIS patients with large ischaemic cores and none-to-mild or moderate BG-EPVS may achieve functional benefit from EVT with regard to independent ambulation, albeit with a potentially increased risk of any ICH. Although several previous studies have reported associations between the BG-EPVS burden and ICH risk in AIS patients,^[44, 45, 46]^ the evidence remains inconsistent. In our analysis, we found no clear evidence that BG-EPVS severity independently increased or modified the risk of clinically significant haemorrhagic complications or mortality after EVT. Therefore, the observed increased risk of any ICH among patients with none-to-mild or severe BG-EPVS who received EVT may largely reflect minor or asymptomatic haemorrhagic changes related to ischaemic-reperfusion injury,^[47]^ rather than a direct effect of BG-EPVS burden. However, notably, the wide confidence intervals for effect estimates in the moderate and severe BG-EPVS subgroups, especially in the severe subgroup, indicate imprecision due to limited small sample size and power. Further studies with larger, adequately powered cohorts are needed to confirm and extend these findings.

The present findings preliminarily suggest that BG-EPVS burden on routine 2D T2-weighted MRI may represent a readily available imaging feature that could help explore heterogeneity in EVT treatment effects among LVO-AIS patients with large ischaemic cores. In clinical practice, visual assessment of BG-EPVS severity is rapid and straightforward and typically requires less than 2–3 min per scan when performed by trained clinicians using validated ordinal rating scales. Our study confirmed substantial interrater reliability, supporting the reproducibility of visual grading among experienced raters. Nevertheless, visual assessment remains inherently subjective and may be influenced by rater experience or imaging quality. These limitations highlight the potential value of AI-assisted automated assessment or quantitative volumetric analysis of BG-EPVS burden using 3D T2-weighted MR images in the future.^[33]^ Importantly, the incremental prognostic value of the BG-EPVS burden as a stratification marker should be considered in the broader clinical context. Established imaging markers such as ASPECTS^[2,30]^ and collateral scores^[48,49]^ remain fundamental to EVT decision-making, whereas BG-EPVS may provide complementary insights into chronic microvascular injury, impaired cerebrovascular reactivity, and the ability of the brain to resist ischaemia. Whether routine assessment of the BG-EPVS burden has additional prognostic value and whether it can be efficiently integrated into the time-sensitive workflow of acute stroke care, should be tested in adequately powered, prospective, multicentre studies with pre-specified analyses and external validation. Standardization of rating protocols and the development of AI-assisted automated assessment may help address current challenges and clarify the clinical utility of BG-EPVS in stroke triage.

The present study has several limitations. First, the exclusion of patients who either did not undergo MRI or had poor-quality imaging may have introduced selection bias; this, combined with the relatively small sample sizes in the subgroups—particularly the severe BG-EPVS subgroup—may have led to insufficient statistical power to detect treatment effects or interactions. Second, CMBs could not be assessed in this study. Although we conducted sensitivity analyses adjusting for other CSVD markers (*e.g*., lacunes, deep and periventricular WMHs), the potential influence of unmeasured CMBs cannot be excluded. Third, MRI-based assessments of EPVS, WMH, and lacunes were performed either before or shortly after EVT (on average within 2 days after onset). However, given that these are chronic CSVD markers, their burden is unlikely to change significantly within 72 h, and this timing variability is unlikely to have substantially affected the results. Moreover, brain MRI for EPVS assessment was performed according to routine clinical MRI protocols at the participating stroke centers rather than a fully standardized trial-specific protocol. Consequently, some variability in scanner type and acquisition parameters (*e.g*., MRI field strength and slice thickness) may have some influence on the visibility and rating of EPVS and other CSVD imaging markers. Fourth, our data were derived from the ANGEL-ASPECT trial, which focused on patients with anterior-circulation LVO and large ischemic cores, thereby limiting the generalizability of our findings. To increase generalizability, future pooled analyses of randomized clinical trials involving more heterogeneous patient populations—such as those with different stroke subtypes or varying degrees of baseline stroke severity—are warranted. In addition, all participants were recruited from stroke centres in China; therefore, further validation in non-Chinese and non-Asian populations is needed to assess the generalizability of our findings. Fifth, EPVS severity was assessed using a visual semi-quantitative scale on conventional 2D T2-weighted MRI. Although standardized STRIVE-based criteria with substantial inter-rater reliability were applied, visual grading may still be susceptible to rater variability. While emerging AI-based approaches now allow automated semi-quantitative EPVS grading on 2D T2-weighted MR images, and high-resolution 3D T2-weighted MRI enables direct volumetric quantification, both require additional acquisition time, technical infrastructure, model development and training, and external validation. Further studies incorporating these advanced and validated automated pipelines are warranted to enable more precise and objective EPVS assessment and to facilitate external validation of our findings. Sixth, as this was a post-hoc, exploratory analysis without prespecified power and no formal adjustment for multiple comparisons, all findings should be considered hypothesis-generating rather than definitive, and interpreted with appropriate caution. Finally, our findings were derived from a single randomized clinical trial, and the observed modification of EVT benefit by BG-EPVS severity has not been validated in independent cohorts. Whether similar patterns exist in other EVT trials or real-world stroke cohorts remains uncertain. Replication in independent randomized trials and prospective EVT registries is needed before BG-EPVS can be considered for risk stratification or treatment selection in clinical practice.

Beyond these limitations, the present findings may also help to inform the design of future confirmatory studies. Because statistical tests of interaction typically require substantially larger sample sizes than main-effect analyses, future EVT trials or pooled analyses should prospectively plan sample size and power to detect modest effect modification by BG-EPVS burden, ensuring adequate representation of patients across BG-EPVS strata, particularly those with severe BG-EPVS burden. The effect sizes and confidence intervals observed in the current study may provide a useful reference for such planning, while recognizing that formal sample size calculations will need to be tailored to specific trial designs. In addition, collaborative multicentre efforts using harmonized MRI acquisition protocols (*e.g*., standardized T2-weighted and FLAIR sequences with predefined spatial resolution and slice thickness) and unified EPVS rating criteria or validated automated pipelines, together with data-sharing initiatives or individual participant data meta-analyses across EVT trials and large stroke registries, may offer an efficient framework to validate and refine BG-EPVS–based risk stratification strategies.

## Conclusion

In this post-hoc analysis of the ANGEL-ASPECT trial, we found that higher severity of BG-EPVS was associated with poorer functional outcomes following EVT in LVO-AIS patients with large ischaemic cores. While EVT significantly improved outcomes in patients with none-to-mild and moderate BG-EPVS, these benefits were not observed in those with severe BG-EPVS. However, given the exploratory, hypothesis-generating nature of this analysis and the relatively small sample size in the moderate and severe BG-EPVS subgroups, these findings should be interpreted with caution. Larger, prospective randomized controlled trials are warranted to validate whether the severity of BG-EPVS can inform patient selection and optimize EVT outcomes in LVO-AIS patients.

## Supplementary Material

Supplementary Material Details
