# Supplementary material for: Impact of enlarged perivascular spaces on endovascular therapy outcomes in patients with large ischaemic core: A post-hoc analysis of the ANGEL-ASPECT trial
Source: J Transl Int Med. 2026 Mar 26;14(2):294–305. doi: 10.1515/jtim-2026-0036 (PMC13110465; doi:10.1515/jtim-2026-0036)
Supplement: Supplementary file 1 — Supplementary Material Details [file jtim-2026-0036_sm.zip › 10 JTIM-D-25-00291 SI 1.pdf]

Supplement 1

Protocol

Protocol for: Huo X, Ma G, Tong X, et al. Trial of endovascular therapy for acute ischemic stroke with large infarct. N Engl J Med 2023;388:1272-83. DOI: 10.1056/NEJMoa2213379

This trial protocol has been provided by the authors to give readers additional information about the work.

Protocol and Statistical Analysis Plan

This trial protocol has been provided by the authors to give readers additional information about their work.

Protocol for: Study of Endovascular Therapy in Acute Anterior Circulation Large VeSsel Occlusive Patients with a LargE InfarCT Core: A Multicenter, Prospective, Open-Label, Blinded-Endpoint, Randomized Controlled Trial.

This supplement contains the following items:

1. **Original protocol in English (page 2 to 64), final protocol in English (page 65 to 128, summary of changes in English (page 129 to 131)**

2. **Original statistical analysis plan in English (page 132 to 145), final statistical analysis plan in English (page 146 to 159), summary of changes in English (page 160 to 161)**

3. **Final protocol in Chinese (page 162 to 230)**

Study of Endovascular Therapy in Acute Anterior Circulation Large VeSsel Occlusive Patients with a LargE InfarCT Core: A Multicenter, Prospective, Open-Label, Blinded-Endpoint, Randomized Controlled Trial (ANGEL-ASPECT)

Protocol

Research team: Beijing Tiantan Hospital, Capital Medical University, Beijing, China

Principal Investigator: Zhongrong Miao, MD, Professor of Neurology

Co- Principal Investigator: Zeguang Ren, Vitor Mendes Pereira

Protocol Version 6.0

Date: July 23, 2020

Table of Contents

Protocol Signature Page **7**

ANGEL-ASPECT Protocol Synopsis **8**

Abbreviations **14**

1. Background **16**

1.1 The rational of EVT for large infarct core volume 16

1.2 Image modality to identify large infarct core volume 18

1.3 ASPECTS and infarct core volume selection 19

1.4 EVT time window for large infarct core volume 19

1.5 ANGEL-ASPECT Trial design 20

2. Study objective **21**

2.1 Primary objective 21

2.2 Secondary objective 21

3. Study design **21**

3.1 Study design 21

3.2 Randomization 22

3.3 Follow-up schedule 22

3.4 Blind design 22

4. Participant selection **23**

4.1 Inclusion Criteria 23

4.1.1 Center Inclusion Criteria 23

4.1.2 Clinical Inclusion Criteria 23

4.1.3 Neuroimaging Inclusion Criteria 24

4.2 Exclusion Criteria 24

4.2.1 Center Exclusion Criteria 24

4.2.2 Clinical Exclusion Criteria 24

4.2.3 Neuroimaging Exclusion Criteria 25

5. Imaging protocol **25**

5.1 Baseline imaging 25

5.2 Intraoperative and follow-up imaging: 27

5.3 Imaging core lab 28

6. Treatments....28

6.1 Endovascular Therapy (EVT)....28

6.2 Best Medical Management (BMM)....29

7. Study endpoints....30

7.1 Primary efficacy endpoint....30

7.2 Secondary efficacy endpoint....30

7.3 Primary safety endpoint....30

7.4 Secondary safety endpoint....31

8. Data collection and Study procedure....31

8.1 Screening and Inclusion....31

8.2 Data to be collected during procedure....32

8.3 Post Treatment (Through Hospital Discharge)....33

8.4 Follow-up Visit at Day 30 (±3), Day 90 (±7) and 12 Months (±14 days)....34

8.5 Unscheduled Follow-up Visit....34

8.6 Schedule of activities and assessments....35

9. Study risk pre-assessment and risk management....36

9.1 Monitoring of adverse events....36

9.2 Definitions of adverse events....36

9.2.1 Adverse event (AE)....36

9.2.2 Serious adverse event (SAE)....36

9.3 Recording of adverse events....37

9.4 Causal relationship between adverse events and study:....37

9.5 Obligation of the investigator regarding safety reporting....37

9.5.1 Adverse events....37

9.5.2 Serious adverse event....37

9.5.3 Follow-up and risk management....38

10. Statistical Analysis....38

10.1 Sample size estimation....38

10.2 Data collection and entry....39

10.2.1 Paper-based CRF filled out by investigator....39

10.2.2 Data entry to the EDC system by CRC....39

10.2.3 Submission to the EDC system after the approval of investigator 40
10.2.4 Data monitoring and query by CRA via EDC 40
10.2.5 Data exportation from the EDC system 40
10.3 Statistical considerations 40
10.3.1 Analysis sets 40
10.3.2 Statistical considerations 41
10.4 Interim analysis 42
11. Ethical standards 43
11.1 Ethical standards 43
11.2 Law and regulations 44
11.3 Informed consent 44
11.4 Institutional review board/institutional ethics committee (IRB/IEC) 44
12. Confidentiality and publication of research findings 45
13. Study Organization 45
13.1 Constitution 45
13.2 Site training and certification 47
14. Study monitoring and quality assurance control 48
14.1 Responsibilities of the investigator(s) 48
14.2 Study monitoring 48
15. Data retention 49
16. Data Security Monitoring 49
17. Registration and Publication 50
17.1 Registration of study summary and results 50
17.2 Publication of study results 50
18. Ownership and use of data 51
18.1 Ownership of the data 51
18.2 Use of collected data 51
19. Funding and conflict of interest 51
20. Reference 52
21. Appendix 55
Appendix Table 1. Modified Rankin Scale 55

Appendix Table 2. Extended Treatment In Cerebral Ischemia (eTICI) Scale 56
Appendix Table 3. National Institute of Health Stroke Scale (NIHSS) 57
Appendix Table 4. EuroQoL 5D-5L..61
Appendix Table 5. Heidelberg bleeding classification...63
Appendix Table 6. Alberta Stroke Program Early CT Score (ASPECTS)...........................64

Protocol Signature Page

I have read this protocol and agree to adhere to the requirements.

By signing this document we confirm that the clinical study will be conducted in accordance with the protocol and all applicable laws and regulations including, but not limited to, the International Conference on Harmonisation Guideline for Good Clinical Practice (GCP) and the ethical principles that have their origins in the Declaration of Helsinki.

____

Clinical Site

______________________________ ________________________

Site Principal Investigator Signature Date

ANGEL-ASPECT Protocol Synopsis

**Official Title**		Study of Endovascular Therapy in Acute Anterior Circulation Large Vessel Occlusive Patients with a Large Infarct Core: A Multicenter, Prospective, Open-Label, Blinded-Endpoint, Randomized Controlled Trial
**Acronym**		ANGEL-ASPECT
**Sponsor**		Beijing Tiantan Hospital, Capital Medical University
**Study Centers**		~50 centers in China
**Statement of Hypothesis**		Best medical management (BMM) combined with endovascular Therapy (EVT) might be superior to BMM alone in acute anterior circulation large vessel occlusive (LVO) patients with a large infarct core.
**Study Objectives**	**Primary objective**	To evaluate if acute ischemic stroke patients with anterior circulation LVO and large infarct core at 0-24 hours after stroke onset have improved neurological functional outcomes when treated with BMM plus EVT compared to BMM alone.
	**Secondary objective**	To assess if acute ischemic stroke patients with anterior circulation LVO and large infarct core at 0-24 hours after stroke onset have increased risk of symptomatic intracranial hemorrhage (sICH) when treated with BMM plus EVT compared to BMM alone.
**Study settings**		Multicenter, Prospective, Randomized, Open-label, Blinded End-point (PROBE) design
**Randomization**		Participants will be randomized in a 1:1 ratio based on simple randomization of the central network

<table>
<tr><td colspan="2"></td><td>randomization system to receive BMM plus EVT or BMM alone.</td></tr>
<tr><td colspan="2">Sample Size</td><td>A total of 488 patients are planned to be enrolled. Interim analysis will take place when 1/2 (244 cases) and 3/4 (366 cases) have completed 3-month follow-up.</td></tr>
<tr><td rowspan="2">Efficacy Endpoints</td><td>Primary Endpoint</td><td>90 days (±7 days) modified Rankin Scale (mRS)</td></tr>
<tr><td>Secondary Endpoints</td><td>(1) 90 days (±7 days) mRS 0-2
(2) 90 days (±7 days) mRS 0-3
(3) 36 hours (±12 hours) NIHSS 0-1 or decrease ≥10 from baseline
(4) Infarct core volume change from baseline, at 7 days (±1 day) or at discharge assessed with NCCT or at 36 hours (±12 hours) assessed with MRI
(5) 36 hour (±12 hours) target artery recanalization rate assessed with CTA or MRA</td></tr>
<tr><td rowspan="2">Safety Endpoints</td><td>Primary Safety Endpoint</td><td>Rate of sICH within 48 hours from randomization (Heidelberg Bleeding Classification)</td></tr>
<tr><td>Secondary Safety Endpoints</td><td>(1) All-cause mortality within 90 days (±7 days)
(2) Any intracranial hemorrhage within 48 hours from randomization (Heidelberg Bleeding Classification)
(3) Decompressive hemicraniectomy during hospitalization</td></tr>
<tr><td>Participants</td><td>Inclusion Criteria</td><td>Center Inclusion Criteria
(1) Equipped with emergency department and neurology department for stroke patients</td></tr>
</table>

		(2) Equipped with stroke team operating 24/7<br>(3) Capable of EVT and intravenous (IV) thrombolysis for acute ischemic stroke patients<br>**Clinical Inclusion Criteria:**<br>(1) Age 18-80 years<br>(2) Presenting with symptoms consistent with acute ischemic stroke<br>(3) Pre-stroke mRS score 0-1<br>(4) NIHSS score 6-30 at the time of randomization<br>(5) Randomization can be finished within 24 hours from stroke onset (stroke onset time is defined as last known well time)<br>(6) Informed consent signed<br>**Neuroimaging Inclusion Criteria:**<br>(1) CTA or MRA proven occlusion of the Internal Carotid Artery (ICA) terminus or M1 segment of Middle Cerebral Artery<br>(2) Imaging evidence of low Alberta Stroke Program Early CT Score (ASPECTS) (based on non-contrast CT) or large infarct Core (defined as rCBF <30% on CT perfusion or ADC<620 $\times 10^{-6}$ mm^2/s on MRI) fulfilling one of the following criteria:<br>1) ASPECTS 3–5<br>2) ASPECTS >5 (6-24 h) with infarct core volume 70-100 ml<br>3) ASPECTS <3 with infarct core volume 70-100 ml<br>(3) Mismatch ratio on CT perfusion or MRI (Tmax>6s volume / Ischemic core volume) >1.2

	**Exclusion Criteria**	**Center Exclusion Criteria**<br>(1) Centers in which the number of acute ischemic stroke cases treated with endovascular procedures are less than 20 per year;<br>(2) Centers unable to comply with the research protocol<br>**Clinical Exclusion Criteria**<br>(1) Females who are pregnant, or those of childbearing potential with positive urine or serum beta Human Chorionic Gonadotropin test<br>(2) Known severe allergy (more severe than skin rash) to contrast agents uncontrolled by medications<br>(3) Refractory hypertension that is difficult to control by medication (defined as persistent systolic blood pressure >185 mmHg or diastolic blood pressure >110 mmHg)<br>(4) Known hemorrhagic tendency (including but not limited to): Baseline platelet count $< 100\times10^9$/L; Heparin was administered within 48 hours with aPTT≥35s; on anticoagulant therapy with warfarin and International Normalized Ratio (INR) > 1.7 (Patients with no history or suspected coagulopathy do not need to wait for laboratory results of INR or aPTT prior to enrollment)<br>(5) Parenchymal organ surgery and biopsy were performed in the past one month<br>(6) Any active bleeding or recent bleeding (gastrointestinal bleeding, urinary bleeding, etc.)

		in the past one month<br>(7) Undergoing hemodialysis or peritoneal dialysis; Known severe renal insufficiency with glomerular filtration rate <30 ml/min or serum creatinine >220 mmol/L (2.5mg/dl)<br>(8) Brain tumor (with mass effect)<br>(9) The expected survival time is less than 1 year (such as comorbidity with malignant tumor, serious heart and lung diseases, etc.)<br>(10) Participation in other interventional randomized clinical trials that may confound the outcome assessment of the trial<br>(11) Other circumstances that the investigator considers inappropriate for participation in the trial or that may pose significant risk to the patient (such as inability to understand and/or follow the study procedures and/or follow up due to mental disorders, cognitive or emotional disorders)<br>**Neuroimaging Exclusion Criteria**<br>(1) Midline shift or herniation, mass effect with effacement of the ventricles<br>(2) Evidence of acute intracranial hemorrhage<br>(3) Acute bilateral strokes or multiple intracranial vessel occlusion
**Treatment Allocation**	**Study Arm**	BMM plus EVT
	**Control Arm**	BMM alone

**Follow-up schedule**	Study visits will take place on the day of randomization, at 36 hours (± 12 hours), 7 days (±1 day)/at discharge whichever is earlier, 30 days (±3 days), 90 days (±7 days) and 12 months (± 14 days).
**Subgroup analysis**	Subgroup analysis will be performed based on the following variables:<br>(1) Age (< 70 years vs. ≥70 years)<br>(2) Last known well to randomization time (< 6 h vs. ≥ 6 h)<br>(3) Stroke severity before randomization (NIHSS<16 vs. NIHSS≥16)<br>(4) IV thrombolysis<br>(5) Occlusion site (intracranial ICA vs. M1 segment)<br>(6) ASPECTS score (< 3 vs. ≥3 points)<br>(7) Infarct core volume (< 70ml vs. ≥70ml)<br>(8) Etiological subtype of stroke (cardiac embolism vs. large artery atherosclerosis)
**Study duration**	August 2020 to October 2022 (enrolment completed October 2021)

Abbreviations

ADC	Apparent Diffusion Coefficient
AE	Adverse Event
aPTT	Activated Partial Thromboplastin Time
ASPECTS	Alberta Stroke Program Early CT Score
BMM	Best Medical Management
CEC	Clinical Events Adjudication Committee
CI	Confidence Interval
CRA	Clinical Research Associate
CRC	Clinical research coordinator
CRF	Case Report Form
CSA	Chinese Stroke Association
CT	Computer Tomography
CTA	Computed Tomography Angiography
CTP	Computed Tomography Perfusion Imaging
DAWN	DWI or CTP Assessment with Clinical Mismatch in the Triage of Wake-Up and Late Presenting Strokes Undergoing Neurointervention with Trevo
DEFUSE 3	Endovascular Therapy Following Imaging Evaluation for Ischemic Stroke 3
DICOM	Digital Imaging and Communications in Medicine
DSA	Digital Subtraction Angiography
DSMB	Data Safety Monitoring Board
DWI	Diffusion Weighted Imaging
ECG	Electrocardiogram
EDC	Electronic Data Capture
EQ-5D-5L	EuroQoL 5-Dimensions 5-Level questionnaire
eTICI	Expanded Thrombolysis in Cerebral Infarction
EVT	Endovascular Therapy
FAS	Full Analysis Set
FLAIR	FLuid Attenuated Inversion Recovery
GCP	Good Clinical Practice
GRE	Gradient Echo
GSR-ET	German Stroke Registry – Endovascular Treatment
ICA	Internal Carotid Artery
ICH	The International Council for Harmonisation of Technical Requirements for Pharmaceutical for Human Use
ICMJE	International Committee of Medical Journal Editors
IEC	Institutional Ethics Committee
INR	International Normalized Ratio

IRB	Institutional Review Board
ITT	Intention-To-Treat
IV	Intravenous
LICV	Large Infarct Core Volume
LLC	Limited Liability Company
LVO	Large Vessel Occlusive
MCA	Middle Cerebral Artery
MM	Medical Management
MRA	Magnetic Resonance Angiography
MRI	Magnetic Resonance Imaging
mRS	Modified Rankin Scale
NCCT	Non-contrast computed tomography
NCSS	Number Cruncher Statistical System
NIHSS	National Institute of Health stroke scale
NMPA	National Medical Products Administration
OR	Odd Ratio
PASS	Power Analysis and Sample Size
PPS	Per Protocol Set
PROBE	Prospective, Randomized, Open-label, Blinded End-point
PWI	perfusion weighted imaging
SAE	Serious Adverse Event
SAP	Statistical Analysis Plan
SAS	Safety Analysis Set
SELECT	Optimizing Patient's Selection for Endovascular Treatment in Acute Ischemic Stroke
sICH	Symptomatic intracranial hemorrhage
THRACE	Mechanical thrombectomy after intravenous alteplase versus alteplase alone after stroke
TICI	Thrombolysis In Cerebral Infarction
T-NICE	Tiantan Neuroimaging Center of Excellence
TOAST	Trial of ORG 10172 in Acute Stroke Treatment

1. Background

Large clinical trials on early and late window stroke patients have helped to establish the indications for endovascular treatment (EVT) of acute ischemic stroke (AIS) patients with large vessel occlusion (LVO).[1,2] The neuroimaging criteria upon which the trials have shown benefit includes patients presenting with Alberta Stroke Program Early CT Scores (ASPECTS) score ≥ 6 within 6 hours,[3-7] and patients meeting DWI or CTP Assessment with Clinical Mismatch in the Triage of Wake-Up and Late Presenting Strokes Undergoing Neurointervention with Trevo (DAWN) and Endovascular Therapy Following Imaging Evaluation for Ischemic Stroke (DEFUSE 3) trial imaging criteria at 6-24 and 6-16 hours from time last seen well, respectively.[8,9] Since then, many clinical trials have undergone an expansion of the indications of EVT for AIS patients with LVO. Whether patients with large infarct core volume (LICV) are suitable for EVT is one of the unanswered questions.

1.1 The rational of EVT for large infarct core volume

Several retrospective studies, prospective studies, and meta-analyses suggest that patients with LICV may benefit from EVT. The Mechanical thrombectomy after intravenous alteplase versus alteplase alone after stroke (THRACE) trial was one of the early randomized trials to enroll patients with ASPECTS<6.[10] A subgroup analysis of the THRACE trial showed that among 53 subjects with a diffusion-weighted imaging (DWI) volume of >70 ml, 12 (22.6%) patients of the EVT group had good clinical outcomes (mRS ≤ 2 at 90 days).[11] The prospective German Stroke Registry – Endovascular Treatment (GSR-ET) also showed that 22% of 152 thrombectomy patients with ASPECTS <6 achieved independence with mRS 0-2 at 90 days.[12] The Highly Effective Reperfusion evaluated in Multiple Endovascular Stroke Trials (HERMES) collaboration pooled their data from six early window trials. It showed a benefit of EVT over control was observed in patients with ASPECTS 0–4 or DWI-determined infarct core volume ≥ 70 ml. Functional improvement (mRS 0-2 at 90 days)

rates in the EVT group compared with the control group were 25% vs. 14% and 30% vs.20%, respectively.[13,14]

In the Optimizing Patient's Selection for Endovascular Treatment in Acute Ischemic Stroke (SELECT) trial, the prespecified secondary analysis of 105 patients (of whom 62 received EVT) with ASPECTS ≤ 5 or CTP-determined ischemic core volume ≥ 50 ml showed that functional independence was achieved in 31% in the EVT group vs. 14% in the control group.[15] Incidence rates of death, neurologic decline, and symptomatic intracerebral hemorrhage (sICH) were similar in both groups. In addition, EVT was associated with less infarct growth (44 vs. 98 mL; p=0.006) and smaller final infarct volume (97 vs. 190 mL; p=0.001) compared to medical management (MM).

One meta-analysis including 17 studies and 1378 patients with ASPECTS 0–6 (1194 EVT, 184 MT) found that mRS 0–2 was achieved in 30.1% of cases after EVT and in 3.2% after MM (OR 4.76, p=0.01).[16] The marked lower rate in the MM group compared to previous RCTs (HERMES: 14%, SELECT: 14%) is likely due to the imbalance of baseline characteristics of the patients in these retrospective studies. For example, patients in the MM group were older (75 years vs 68.7 years), had higher NIHSS scores (19 vs. 18), lower rate of intravenous (IV) thrombolysis (47.8% vs. 56.8%) and longer symptom onset to admission time (130 min vs. 115 min). Successful recanalization (Thrombolysis in Cerebral Infarction (TICI) grade 2b–3) led to higher odds of mRS 0–2 than unsuccessful reperfusion (OR 5.2, p=0.001). Another pooled random-effect meta-analysis, including 12 studies of large core patients (ASPECTS <6 or ischemic core volume ≥ 50 ml), demonstrated higher functional independence (mRS 0–2) rates with EVT (25% vs 7%; pooled OR: 4.39, 95% CI: 2.53 to 7.64), and decreased mortality (23% vs. 33%; pooled OR: 0.53, 95% CI: 0.40 to 0.71).[17]

In a matched case-control study of 56 patients (28 pairs) with ICA, M1 and M2 occlusion and CTP-determined infarct core > 50mL, EVT led to higher rates of functional independence (90-day mRS 0-2, 25% vs 0%; p=0.04), and smaller final infarct volumes (87 vs 242 mL; p <0.001).[18] One control (4%) and two treatment patients (7%) developed a parenchymal hematoma type 2 (p>0.99). The rates of

hemicraniectomy (7% vs 21%; p=0.10) and 90-day mortality (29% vs 48%; p=0.75) were lower in the EVT arm. Sensitivity analysis for patients with a baseline infarct core volume greater than 70 mL (12 pairs) revealed a significant reduction in final infarct volumes (110 vs. 319 mL; p<0.001) but only a nonsignificant improvement in the overall distribution of mRS scores favoring the treatment group (p=0.18).

Interestingly, one observational cohort study included a consecutive sample of 170 patients with anterior circulation stroke and initial ASPECTS ≤ 5 (99 patients in the EVT group, 71 patients in the MM group). The study showed that clinical outcome after failed or incomplete EVT (TICI 0–2b) was significantly better compared to patients with MM only (median mRS 5, interquartile range 4–6 vs. 5–6, p=0.03). Failed EVT (TICI 0–2a) was not associated with a worse outcome than MM.[19]

1.2 Image modality to identify large infarct core volume

Generally speaking, there are two imaging evaluation methods for large infarct core, one is a semi-quantitative evaluation based on CT/MRI-ASPECTS, and the other is a quantitative evaluation based on CTP/MRI with the aid of automated artificial intelligence software. ASPECTS is a widely accepted tool used to assess infarct volume. In general, ASPECTS < 6 is regarded as a “large core infarct.” However, multiple studies have shown low interrater agreement with ASPECTS.[20,21] An inaccurate ASPECTS can misclassify patients between the EVT and control groups, weakening any trial conclusions. Quantitative determination of infarct core volume using CTP/MRI could compensate for poor inter-rater reliability in the interpretation of ASPECTS.

Notably, the correlation between the CTP/MRI-determined infarct core volume and ASPECTS is not well established. Therefore, the optimal imaging modality for evaluating patients with LICV in clinical trials remains to be explored. The subgroup analysis in a meta-analysis comparing outcomes between these two imaging modalities did not find significant heterogeneity in the results when LICV was defined based on ASPECTS or ischemic core volume of CTP.[17] While one study[22] found a good

correlation between ASPECTS and CTP/MRI volume, others found them to be discordant.[15,17,23] To expedite enrollment, this study allowed the use of non-contrast CT (NCCT)-ASPECTS and/or CTP/MRI imaging modalities to screen patients with LICV.

1.3 ASPECTS and infarct core volume selection

A recent meta-analysis of 17 studies and 1378 patients reported functional independence, or a mRS 0-2, was achieved by 37.7%, 33.3%, 22.1%, and 17.1% of patients with ASPECTS 6, 5, 4 and 0-4 respectively.[16] The studies by Mourand et al.[24] and Inoue et al.[25] showed favorable outcomes in between 16% and 20% of patients with ASPECTS 0–3 after EVT. Another meta-analysis showed that patients presenting with an ASPECTS 0-2 had better outcomes with MM instead of EVT.[13] The benefit of EVT declined with decreasing in ASPECTS, especially with ASPECTS < 3, because the infarct core volume was very large and there was less salvageable brain tissue, which might make EVT ineffective.[13,26,27] Therefore, this study limited the ASPECTS of enrolled patients to 3-5.

There is some debate about whether "large core" should be defined as 50ml vs. 70ml on CTP. In ANGEL-ASPECT, the infarct core volume > 70 ml was defined as LICV. Similar to patients with ASPECTS 0-2, patients with excessive infarct core volume may also be less likely to benefit from EVT. Previous studies showed a lack of benefit if CTP-determined core volume exceeded 100 ml and 150 ml.[14,15] Therefore, when patients were enrolled only based on the infarct core volume as assessed by CTP/MRI, this study limited the infarct core volume to 70 ml-100 ml.

1.4 EVT time window for large infarct core volume

Patients in the hyperacute phase of stroke may exhibit an increased ASPECTS lesion growth from imaging to recanalization, suggesting a benefit of faster recanalization in these patients.[28] A meta-analysis by Cagnazzo et al. demonstrated that a shorter time from onset to reperfusion was associated with a higher probability of

functional independence after EVT in patients with ASPECTS 0–6.[16] The SELECT trial found that patients with LICV had a gradual decline in functional outcomes with prolonged treatment time, with a lower likelihood of benefit from EVT after 12 hours from symptom onset or time last seen well.[15] This suggested that for LICV patients, earlier EVT may be more beneficial. However, a recent meta-analysis found that patients with LICV did not show a significant difference in outcomes among studies reporting <6 hours, <12 hours, and <24 hours for stroke to EVT time windows.[17] This may be because most LICV patients presented in the early time window, reducing the power to detect a difference between early and late windows. The reason may also be that the efficacy of MM declines over time, thus preserving the efficacy of EVT. In this context, it is important to study whether EVT also benefits patients with LICV in the late time window, therefore the time window of ANGEL-ASPECT is 0-24 hours.

1.5 ANGEL-ASPECT Trial design

The ANGEL-ASPECT trial is a PROBE study initiated by researchers to explore the effectiveness and safety of EVT in patients with anterior circulation large vessel occlusion stroke with ASPECTS 3-5 or infarction core volume 70-100ml within 24 hours of symptom onset. The ANGEL-ASPECT trial allows multiple imaging modalities to screen for LICV patients, but at the same time imposes limitations on the range of ASEPCTS or infarct core volume. The purpose was to reduce the risk of EVT while enrolling as many LICV patients as possible. The primary image inclusion criteria of ANGEL-ASPECT was NCCT-ASPECTS 3-5, and the infarct core volume of 70 ml-100 ml was used as auxiliary inclusion criteria. Briefly, the inclusion criteria for LICV are: (1) If NCCT-ASPECTS is 3-5 and presentation is within 24 hours of onset, patients are enrolled without limitation of infarct core volume. (2) For NCCT-ASPECTS 0-2 and core infarction volume 70 ml-100 ml, patients are enrolled. (3) If NCCT-ASPECTS is >5 and between 6 to 24 hours from symptom onset, only patients with infarct core volume 70 ml-100 ml are enrolled.

Subgroup analysis will focus on age, LKW to randomization time, NIHSS score,

IV thrombolysis, occlusion site, ASPECTS, infarct core volume, and stroke etiology.

ANGEL-ASPECT is the only randomized controlled trial conducted in China for LICV patients thus far. The results of this trial will clarify whether EVT is effective and safe in Chinese patients with LICV.

2. Study objective

2.1 Primary objective

To evaluate if acute ischemic stroke patients with anterior circulation LVO and large infarct core at 0-24 hours after stroke onset have improved neurological functional outcomes when treated with BMM plus EVT compared to BMM alone.

2.2 Secondary objective

To assess if acute ischemic stroke patients with anterior circulation LVO and large infarct core at 0-24 hours after stroke onset have an increased risk of sICH when treated with BMM plus EVT compared to BMM alone.

3. Study design

3.1 Study design

Multicenter, Prospective, Randomized, Open-label, Blinded End-point (PROBE) trial design (Figure 1).

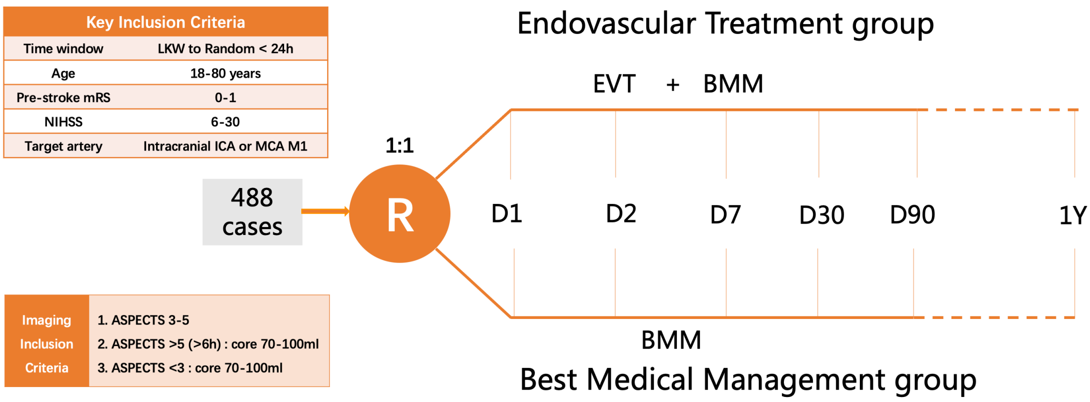

Figure 1. Study design: Randomization Algorithm

3.2 Randomization

The random code will be generated by a central network randomization system with 24h real-time randomization online based on the simple randomization method. The researcher in each center will obtain the random code from the central network randomization system according to the enrollment order. Patients who meet the inclusion criteria and in whom written informed consent can be obtained, will be randomly assigned to the following treatment groups in a 1:1 ratio:

- BMM plus EVT group: patients will receive EVT with stent retriever or contact aspiration as first-line devices for thrombectomy plus BMM;
- BMM group: Patients will receive BMM alone.

3.3 Follow-up schedule

(1) Face-to-face visit: day of randomization, 36 hours (±12 hours), 7 days (±1 day) /at discharge whichever is earlier

(2) Telephone visit: 30 days (±3 days), 90 days (±7 days), and 12 months ±14 days

3.4 Blind design

(1) Only the patient and the treating physician are aware of the randomization

information, and the evaluation of information at baseline and in-hospital visits that relate to the study endpoints should be evaluated by an investigator who is not aware of the patient groups and actual treatment.

(2) The primary endpoint visits are standardized visits conducted by trained third party personnel who are not aware of the patient's randomization assignment and their treatment. All follow-up calls will be recorded, and a follow-up report will be formed.

(3) All imaging data related to the study will be collected for centralized interpretation. The images at each visit site will be interpreted independently by a core lab, and the readers will be unaware about the baseline, treatment received (except EVT angiography images), and prognosis.

4. Participant selection

4.1 Inclusion Criteria

4.1.1 Center Inclusion Criteria

(1) Equipped with an emergency department and neurology department for stroke patients

(2) Equipped with a stroke team operating on 24/7

(3) Capable of endovascular therapy and IV thrombolysis for acute ischemic stroke patients

4.1.2 Clinical Inclusion Criteria

(1) 18 to 80 years of age

(2) Presenting with symptoms consistent with an acute ischemic stroke

(3) Pre-stroke mRS score 0-1

(4) NIHSS score 6-30 at the time of randomization

(5) Randomization can be finished within 24 hours of stroke onset (stroke onset time is defined as last known well time)

(6) Informed consent signed by the patient or legally authorized representative

4.1.3 Neuroimaging Inclusion Criteria

(1) CTA or MRA proven occlusion of the Internal Carotid Artery (ICA) terminus or M1 segment of the Middle Cerebral Artery (MCA)

(2) Imaging evidence of low ASPECTS (based on NCCT) or large infarct Core (defined as rCBF <30% on CT perfusion or ADC<620 $\times 10^{-6}$ mm^2/s on MRI) fulfill one of the following criteria:

  1) ASPECTS 3-5

  2) ASPECTS >5 (6h-24 h) with infarct core volume 70-100 ml

  3) ASPECTS <3 with infarct core volume 70-100 ml

(3) Mismatch ratio on CT perfusion or MRI (Tmax>6s volume / Ischemic core volume) >1.2

4.2 Exclusion Criteria

4.2.1 Center Exclusion Criteria

(1) Centers in which the number of acute ischemic stroke cases treated with endovascular procedures are less than 20

(2) Incapable of complying with the protocol to proceed with the research

4.2.2 Clinical Exclusion Criteria

(1) Females who are pregnant, or those of childbearing potential with positive urine or serum beta Human Chorionic Gonadotropin test

(2) Known severe allergy (more than a rash) to contrast media uncontrolled by medication

(3) Refractory hypertension that is difficult to be controlled by medication (defined as persistent systolic blood pressure >185 mmHg or diastolic blood pressure >110 mmHg)

(4) Known hemorrhagic tendency (including but not limited to): Baseline platelet count < 100×10^9/L; Heparin was administered within 48 hours with aPTT≥35s; on anticoagulant therapy with warfarin and International Normalized Ratio (INR) > 1.7 (Patients with no history or suspected coagulopathy do not need to wait for laboratory results of INR or

aPTT prior to enrollment)

(5) Parenchymal organ surgery and biopsy were performed in the past one month

(6) Any active bleeding or recent bleeding (gastrointestinal bleeding, urinary bleeding, etc.) in the past one month

(7) Undergoing hemodialysis or peritoneal dialysis; Known severe renal insufficiency with glomerular filtration rate <30ml/min or serum creatinine >220mmol/L (2.5mg/dl)

(8) Brain tumor (with mass effect)

(9) The expected survival time is less than 1 year (such as comorbidity with malignant tumor, advanced heart or lung disease, etc.)

(10) Participation in another interventional randomized clinical trial that may confound outcome assessment of the study

(11) Other circumstances that the investigator considers inappropriate for participation in the study or that may pose significant risks to patients (such as inability to understand and/or follow the study procedures and/or follow up due to mental disorders, cognitive or emotional disorders)

4.2.3 Neuroimaging Exclusion Criteria

(1) Midline shift or herniation, mass effect with effacement of the ventricles

(2) Evidence of acute intracranial hemorrhage

(3) Acute bilateral strokes or multiple intracranial vessels occlusions

5. Imaging protocol

5.1 Baseline imaging

All researchers will be trained in the course of the imaging protocol, the use of RAPID software, participate in the network training, simulation test and examination of NCCT-ASPECTS before enrollment. The ASPECTS training and test are conducted through the online training system of the trial website (http://angel-aspect.org). Those

who pass the exam (accuracy rate more than 80%) will obtain the ASPECTS assessment qualification certificate and be qualified for imaging assessment. During imaging screening, researchers in the sub-center with imaging evaluation qualifications and two trained neuroradiologists from the trial team will conduct real-time online image evaluation of ASPECTS, occlusion site, infarct core volume to ensure the accuracy of the imaging assessment (Figure 2).

(1) **ASPECTS:** All patients presenting within 24h of symptom onset will undergo a plain CT scan. After the preliminary screening of ASPECTS by trained clinicians in research centers, two dedicated clinicians from the trial team will conduct real-time online evaluation. When the ASPECT score reaches a consensus that is between 3 to 5, the patient is suitable for enrollment. NCCT-ASPECTS will be manually determined independently before RAPID ASPECTS® (version 5.0.4, iSchemaView, CA, USA) assessment.

(2) **Infarct core volume:** The infarct core volume will be automatically evaluated by iSchemaView automated RAPID® software (version 5.0.4, iSchemaView, CA, USA), and the infarction core volume is defined as rCBF<30% based on CTP or ADC<620 $\times 10^{-6}$ mm^2/s based on MRI. For patients who present with NCCT-ASPECTS 0-2 within 6 hours from symptom onset, if the infarct core volume is between 70ml and 100ml, then the patient is eligible for enrollment. If the infarct core volume is between 70ml and 100ml in an extended time window (6-24 hours) of stroke onset, the patient is also suitable for inclusion regardless of ASPECTS.

(3) **Target arterial occlusion:** The site of arterial occlusion will be determined by CTA or MRA. Occlusion of the ICA or M1 segment of the MCA is suitable for enrollment. Patients with ipsilateral extracranial internal carotid artery occlusion or stenosis with the above artery occlusion will also be included in this trial. As a tandem lesion can be difficult to distinguish on CTA or MRA, patients with tandem lesion in the EVT group will be confirmed during angiography.

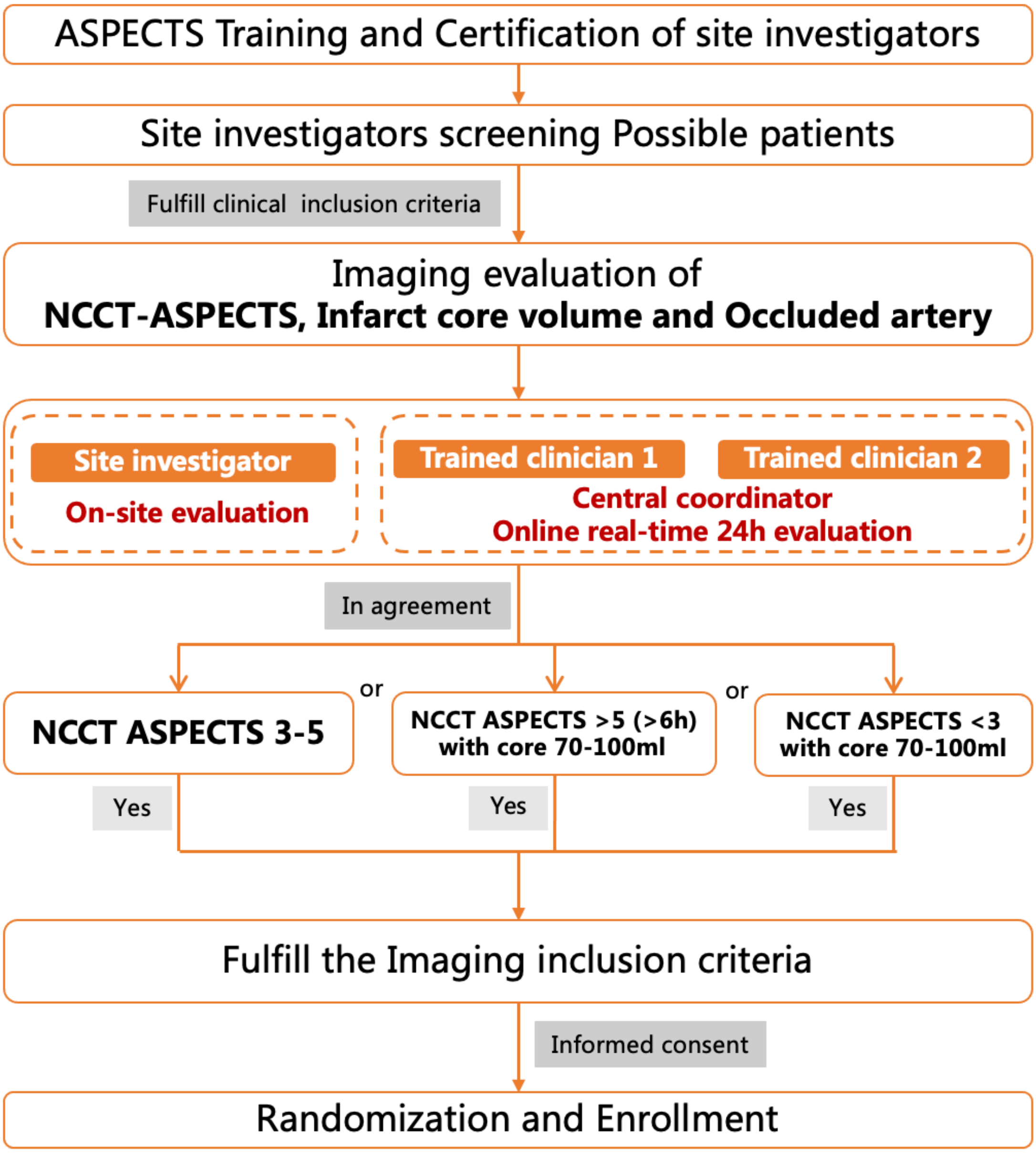

Figure 2. Imaging evaluation workflow

5.2 Intraoperative and follow-up imaging:

(1) **Intraoperative imaging:** In the EVT group, preoperative DSA will be required to determine the site of vascular occlusion and eTICI score,[29] and postoperative angiography will be required to evaluate the eTICI score. It is recommended to conduct NCCT or cone-beam CT immediately after EVT to exclude bleeding.

(2) **Imaging evaluation of hemorrhage:** The NCCT scan 36 hours (±12 hours) after

randomization will be utilized as the main criterion to adjudicate hemorrhage, and the Heidelberg Bleeding Classification will be used for hemorrhage classification.[30]

(3) **Vascular imaging follow-up:** Vascular imaging (CTA/MRA) will be conducted within 36 hours (±12 hours) h after randomization to determine vascular patency.[31]

(4) **Evaluation of postoperative infarct volume:** The infarct core volume will be determined at 7 days (±1 day) or at discharge as assessed with NCCT or at 36 hours (±12 hours) assessed with MRI, and the infarct core volume will be determined manually by the imaging core lab using validated automated software.[32]

5.3 Imaging core lab

The Tiantan Neuroimaging Center of Excellence (T-NICE) is the imaging core lab of this trial. During the period from patient presentation to discharge, all imaging data (CT, CTA, CTP, MRI, MRA, PWI, DSA) will be collected by CRO in DICOM format. All images will be quality controlled, rendered anonymous and sent to T-NICE for central adjudication. The final results will be reviewed and confirmed by the imaging assessment committee, and then input into the database.

6. Treatments

Patients meeting the eligibility criteria and signing the informed consent will be randomized. Patients randomized to the interventional arm will receive BMM plus EVT. Artery puncture should be performed within 1 hour of randomization. Patients randomized to the medical arm will receive BMM.

6.1 Endovascular Therapy (EVT)

When the patient's condition permits, local anesthesia is the first choice for rapid initiation of puncture and endovascular therapy. Conscious sedation can be used, and intubation can be considered for patients at high risk of airway collapse. If the patient is expected to have poor intraprocedure cooperation, is at high risk of using sedation or

if there are other airway conditions due to the patient's illness, general anesthesia should be used. Return to the Neuro-Intensive Care Unit (NICU) with intubation or not will be determined according to the procedural results.

Systemic heparinization is not recommended for preoperative and intraoperative treatment. Femoral artery is suggested for arterial puncture, and a long sheath, guiding catheter or balloon guide catheter can be used. Stent retriever (Solitaire, EMBOTRAP, Reco, Captor or other first-line stent retriever systems) and/or contact aspiration (Penumbra aspiration system or other first-line aspiration system) are recommended as the first-line choice for thrombectomy. If successful reperfusion (eTICI 2b50-3) is not achieved after routine thrombectomy, other techniques are allowed for rescue treatment, including replacement of thrombectomy technique, replacement of thrombectomy device, intra-arterial thrombolysis, balloon angioplasty or stent implantation, etc. The need for rescue treatment is defined (including, but not limited to, a decision made by the investigator based on intraprocedure conditions) as follows: three thrombectomy passes with the same device (stent retriever or aspiration catheter) without successful recanalization; Target vessels were successfully recanalized and reoccluded; Target vessel dissection or stenosis ≥70%, with any degree of antegrade blood flow disturbance; New intraluminal/stent thrombosis resulting in a decrease of eTICI score.

All the above procedures should be performed using devices approved by the National Medical Products Administration (NMPA) and should be performed in accordance with the approved intended use and operating instructions.

6.2 Best Medical Management (BMM)

All enrolled patients should receive BMM in accordance with the recommendation of the “Chinese Stroke Association guidelines for clinical management of cerebrovascular disorders” by the Chinese Stroke Association (CSA).[33] This includes IV thrombolysis therapy for patients meeting the guideline. Patients who meet criteria should receive IV thrombolysis therapy according to the guidelines. Patients who plan to undergo or are undergoing IV thrombolysis therapy can decide whether to terminate

IV thrombolysis therapy in advance according to the investigator's judgment after enrollment. Patients who had completed IV thrombolysis prior to randomization are also eligible for inclusion in this study. All patients will be required to record the name, dosage and time of IV thrombolysis medication in detail. Antiplatelet agents are not recommended within 24 hours after IV thrombolysis unless the patient has undergone balloon dilatation or stent implantation, at which time the antithrombotic strategy is determined by the investigator. Based on the time window and infarct core volume for ANGEL-ASPECT, it is anticipated that most patients enrolled in ANGEL-ASPECT will not have received IV thrombolysis prior to randomization. Non-IV thrombolysis patients will be treated with aspirin, unless an indication for early anticoagulation is present.

7. Study endpoints

7.1 Primary efficacy endpoint

90 days (±7 days) modified Rankin Scale (mRS)

7.2 Secondary efficacy endpoint

(1) 90 days (±7 days) mRS 0-2
(2) 90 days (±7 days) mRS 0-3
(3) 36 hours (±12 hours) NIHSS 0-1 or decrease ≥10 from baseline
(4) Infarct core volume change from baseline, at 7 days (±1 day) or at discharge assessed with NCCT or at 36 hours (±12 hours) assessed with MRI
(5) 36 hour (±12 hours) target artery recanalization rate assessed with CTA or MRA

7.3 Primary safety endpoint

Rate of sICH within 48 hours from randomization (Heidelberg Bleeding Classification, Appendix 5)

7.4 Secondary safety endpoint

(1) All-cause mortality within 90 days (±7 days)
(2) Any intracranial hemorrhage within 48 hours from randomization (Heidelberg Bleeding Classification)
(3) Decompressive hemicraniectomy during hospitalization

8. Data collection and Study procedure

Investigator(s) should keep a record, the eligibility Case Report Form (CRF), of subjects who enter pre-study screening. The sub-center number must be indicated. The screening table will be used to analyze and determine whether the enrolled patients in different study sites are representative. Referring to the procedure manual and data collection guidelines, investigators should guarantee the input of CRF is precise, complete and timely, and answer the queries in time. Brain imaging which includes: CT, CTA, CTP, MRI (T1+T2+DWI+FLAIR+ADC+GRE-T2*/SWI+MRA±PWI) and DSA will be collected as DICOM format. Laboratory results will be collected via photocopies of the reports.

8.1 Screening and Inclusion

- Basic data collection: hospital name, name of patient identification, age, sex, allergy to contrast agent.
- History of present event: time of onset (time of last known well), time of arrival at the hospital, type of onset, IV thrombolysis after onset (initial time, name and dose of the drug).
- Medical history and medication before onset

  Medical history (smoking, drinking, hypertension, diabetes, dyslipidemia, cardiac arrhythmia, valvular heart disease, cardiac insufficiency, coronary atherosclerotic heart disease, peripheral arterial disease, TIA,

cerebral infarction, cerebral hemorrhage, intracranial tumors), pre-stroke mRS;

Combination therapy: Antiplatelet drugs (aspirin, clopidogrel, cilostazol, prasugrel, ticagrelor, ticlopidine, etc.), anticoagulants (warfarin, dabigatran, rivaroxaban, apixaban, edoxaban, heparin, argatroban, etc.), statins (atorvastatin, pitavastatin, rosuvastatin, pravastatin, simvastatin, fluvastatin, etc.).

- Physical examination

Height and weight, blood pressure, pulse, neurological evaluation (NIHSS and Glasgow scale), 12-lead electrocardiogram (ECG) .

- Emergency laboratory examinations

Emergency blood routine, emergency renal function, emergency liver function (transaminase), emergency coagulation, random blood glucose, etc.

- Imaging

A head CT is required to rule out hemorrhage and perform ASPECTS evaluation; a CTA or MRA is essential to confirm the site of artery occlusion; a CTP or MRI is required to calculate the infarct core volume

- All participants or his/her authorized representative need to sign a written informed consent form
- Included subjects will be randomized

8.2 Data to be collected during procedure

- General anesthesia with intubation or conscious sedation with local anesthesia at start of procedure

- Procedure times: groin puncture time, time of each pass finish, time of initial flow restoration, time of successful recanalization and the end of the procedure
- eTICI score: pre-procedure and post-procedure
- Details of procedure: Accessory and adjunctive devices used (guide catheter, guidewire, intermediate catheter, microcatheter), number and type of devices for thrombectomy, number of recanalization attempt deployments, rescue procedures with medications besides thrombectomy
- Medication during the procedure: heparin, tirofiban, alteplase, urokinase.
- Intraprocedural complications: Presence of vasospasm (time of onset, vessels involved, time resolved, treatment required), evidence of clot migration or embolization, dissection, perforation, etc.

8.3 Post Treatment (Through Hospital Discharge)

A face-to-face observation will be performed at 36 hours (±12 hours) and 7 days (± 1 day)/at discharge whichever is earlier after randomization.

- Brain imaging performed at 36 (±12) hours after randomization: CT/CTA or MRI/MRA.
- A physical exam, as well as clinical and neurological assessments, will be completed at 36 (±12) hours and 7 (±1) days/at discharge whichever is earlier after randomization. Data to be collected include: vital signs (blood pressure and heart rate), relevant concomitant medications (including antiplatelet, anticoagulant, and antihypertensive agents), significant findings from clinical assessment and physical exam (i.e. all new, worsening, or improved conditions), all significant neurological findings, NIHSS Score (both), mRS (7 days or discharge only), and adverse event (AE).
- Head CT evaluation is needed at 7 days (±1 day) or at discharge whichever is earlier
- Laboratory examinations will be collected at 24 hours (±12 hours) and 7 days (± 1 day) or at discharge whichever is earlier after randomization, including: blood routine, renal function, liver function, coagulation, fasting blood glucose, etc.

8.4 Follow-up Visit at Day 30 (±3), Day 90 (±7) and 12 Months (±14 days)

These follow-up evaluations can be performed via telephone if it's not convenient for an in-person visit at the investigational site. All subjects entered into the study will undergo a standard neurological assessment by experienced physicians who are blinded to treatment assignment. Data to be collected include:

- mRS
- Patient-reported functional health status and quality of life using EuroQoL 5-Dimensions 5-Level questionnaire (EQ-5D-5L)[34]
- Relevant concomitant medications
- Significant findings from clinical assessment and physical exam (i.e. all new, worsening, or improved conditions since discharge)

8.5 Unscheduled Follow-up Visit

If an unscheduled follow-up visit occurs after randomization at the investigational site during the study, the incidence of any new or unresolved AEs will be assessed. If the visit is due to a change in neurological status, NIHSS and mRS will be completed by a certified rater.

8.6 Schedule of activities and assessments

Measurements	Baseline	36-hour (±12 hours) visit	7±1 day /at discharge visit	30-day (±3 days) visit	90-day (±7 days) visit	12-month ±14 days visit
Informed Consent	x					
Inclusion/Exclusion	x					
Randomization	x					
Demographic characteristics	x					
History of present illness	x					
Past medical history	x					
Relevant Concomitant Medications	x	x	x	x	x	x
mRS	x		x	x	x	x
NIHSS	x	x	x			
Head CT	x		x[3]			
CTA±CTP or MRI*+MRA±PWI	x[1]					
CT+CTA or MRI*+SWI+MRA		x[2]				
Carotid CTA/MRA/ultrasound		x[4]				
ASPECTS on CT	x					
Ischemic volume on CTP/DWI	x	x[5]	x[5]			
Laboratory examinations	x	x	x			
Electrocardiogram	x					
TOAST			x			
EQ-5D-5L scale				x	x	x
AE/SAE		x	x	x	x	x

[1] For all enrolled cases, CT+CTA+CTP examination will be the first choice before randomization

[2] All the enrolled cases should be reviewed with multi-modal imaging 24-48 hours after randomization, and the imaging evaluation method should be the same as before randomization

[3] All enrolled cases should be examined by head CT at 7 days (±1 day) after randomization or at discharge whichever is earlier

[4] Only applicable to patients in the standard medical treatment group who did not undergo cervical angiography prior to randomization

[5] Based on head CT or MRI, determined by the core imaging laboratory

*MRI sequence includes T1+T2+DWI+ADC+FLAIR sequence

ADC: apparent diffusion coefficient; AE: adverse event; ASPECTS: Alberta stroke program early computed tomography score; CT: computed tomography; CTA: computed tomography angiography; CTP: computed tomography perfusion; EQ-5D-5L: EuroQoL 5-Dimensions 5-Level questionnaire; FLAIR: fluid attenuated inversion recovery; MRA: magnetic resonance angiography; MRI: magnetic resonance imaging; mRS: modified Rankin scale; NIHSS: National Institutes of Health Stroke Scale; PWI: perfusion weighted imaging; SAE: serious adverse event; SWI: susceptibility weighting imaging; TOAST: Trial of ORG 10172 in Acute Stroke Treatment.

9. Study risk pre-assessment and risk management

9.1 Monitoring of adverse events

All AEs will be managed and reported in compliance with all applicable regulations and will be included in the final Clinical Study Report (CSR).

9.2 Definitions of adverse events

9.2.1 Adverse event (AE)

Adverse Events, as long as they occur from the first visit planned in the Clinical Trial Protocol/signature of the informed consent (i.e., occurring during the washout period) to the last visit planned in the protocol, are adverse medical events or deterioration of qualifying event. AEs include symptoms (ie, nausea, chest pain), signs (ie, tachycardia, liver enlargement) and abnormal laboratory results (ie, laboratory or ECG abnormalities). AEs can be classified as serious adverse events (SAEs) and non-serious AEs.

9.2.2 Serious adverse event (SAE)

A Serious adverse event refers to an event that：

- Results in death, or
- Is life-threatening, or

Note: The term "life-threatening" in the definition of "serious" refers to an event in which the patient is at risk of death at the time of the event; it does not refer to an event which hypothetically might have caused death if it was more severe.

- Requires inpatient hospitalization or prolongation of existing hospitalization, or

- Results in persistent or significant disability/incapacity, or
- Is a congenital anomaly/birth defect, or
- Is a medically important event

9.3 Recording of adverse events

Non-serious AE: Only some of the non-serious AEs will be collected from time of randomization throughout the treatment/follow-up periods to the Study Closure Visit. Other non-serious AEs are up to the investigator to decide whether to collect.

SAE: All SAEs will be collected and recorded.

9.4 Causal relationship between adverse events and study:

Attribution of: (1) Definite; (2) Probably; (3) Possibly; (4) Unlikely; (5) Not related; (6) Not applicable.

9.5 Obligation of the investigator regarding safety reporting

9.5.1 Adverse events

All AEs will be recorded on the corresponding page(s) in the CRF. Whenever possible, symptoms should be grouped as a single syndrome or diagnosis. The Investigator should specify the date of onset, intensity, action taken with respect to the Investigational Product, corrective treatment/therapy given, outcome and his/her opinion about whether it is possible that the AE is caused by the study intervention, related to the index stroke, other cause, or intercurrent condition.

9.5.2 Serious adverse event

For SAEs, the investigator must immediately take corresponding measures:

Immediately notify the representative of the Monitoring Team, send the signed and dated corresponding pages in the CRF to the representative of the Monitoring Team,

and attach a photocopy of all examinations conducted and the examination dates. For laboratory results, include the laboratory normal ranges. The contact information (name, address and fax number) of the representative is on the Clinical Trial Protocol. These measures should be completed no later than **24 hours** after SAE.

Care should be taken to ensure that the patient's identity is protected and the patient's identifiers in the Clinical Trial are properly noted on all copies of source documents provided to the Sponsor.

9.5.3 Follow-up and risk management

The Investigator should take all appropriate measures to ensure the safety of the patients.

Screening of subjects should strictly follow the inclusion and exclusion criteria of the study. If an AE occurs during the study period, relevant evaluations will be performed, including blood routine examination, coagulation, creatinine, hepatic function, renal function, arterial blood gas analysis, ultrasound and computer tomography. Targeted treatment and necessary consultation should be carried out in a timely manner. When addressing SAEs, it is important to make sure the patient’s airway is clear, respiration, blood pressure and heart rate are stable.

Notably, the investigator should follow up the outcome of any AEs (clinical signs, laboratory values or other, etc.) until the patient's condition returns to normal or stabilizes. The follow-up will continue even if the patient withdraws from the clinical trial, and the patient will be interviewed by telephone or face-to-face at the scheduled visit time. The monitoring team may request additional visits and investigations.

10. Statistical Analysis

10.1 Sample size estimation

In this study, a multicenter, open, randomized, parallel control design method will be used. The primary measure of efficacy will be the mRS score at 90 days (±7 days) after enrollment

(considered as ordered variable). According to the literature data and clinical expert opinions, the parameters were set as follows: (1) The proportion of mRS score 0-6 in control group will be 3%, 4%, 10%, 17%, 16%, 12% and 38%, respectively; (2) The average treatment effect of EVT improves the outcome with the common OR value for improvement of mRS reaching 1.74; (3) Two Interim analysis are considered. The adjusted level α is 0.050 and power 1-β is 0.90. (4) The randomization will be allocated to the intervention and the control group in a 1:1 ratio. Based on these parameters, the sample size will be 219 in each group. Considering a 10% attrition rate, the final total sample size will be 488 patients, with 244 patients in each group.

Interim analysis will take place when 1/2 (244) and 3/4 (366) of patients will have completed 3-month follow-up." The corresponding significance levels based on the O'Brien & Fleming boundary are 0.003 (stage 1), 0.018 (stage 2) and 0.044 (stage 3, final analysis).

The PASS software (NCSS, LLC, version 11) was used to calculate the sample size.

10.2 Data collection and entry

Paper-based CRF and electronic data capture (EDC) system will be used for data collection and input. All the content required by the protocol in the system must be filled. The unfilled content should be explained, and the reason needs to be marked in the EDC system.

10.2.1 Paper-based CRF filled out by the investigator

Site investigators should use black or blue-black recording pens to fill out the paper-based CRF neatly and clearly to ensure that the data is clear and readable. If the paper-based CRF information needs to be modified, it should not be altered or overwritten. The correct information should be written next to the original information, signed and dated by the person who modified it. The clinical research monitor (Clinical Research Associate, CRA) will review the completeness and accuracy of the CRF and guide the investigator to make necessary corrections and supplements.

10.2.2 Data entry to the EDC system by CRC

After the paper-based CRF is completed, the Clinical research coordinator (CRC) will

input the content of the paper CRF into the EDC system.

10.2.3 Submission to the EDC system after the approval of the investigator

The paper-based CRF will be submitted after the investigator has approved it. After the data is submitted, all data revisions and feedback are carried out through the EDC system. If the EDC system has submitted a form that needs to be modified, contact the CRA of this center. After the CRA opens the form, the investigator can guide the CRC to modify the data in EDC system.

10.2.4 Data monitoring and query by CRA via EDC

10.2.5 Data exportation from the EDC system

After the data from the EDC system is exported to the database, it will be proofread by the data administrator. Obvious errors will be corrected by the data administrator. Other errors or missing values will be filled in the data query form, and the query form will be sent to the participating center for solutions through email, express, telephone or WeChat.

The participating centers are responsible for correcting the data in the EDC system after verifying the original data and related information. Site investigators must answer these queries by verifying or modifying relevant information or data.

10.3 Statistical considerations

This section is an overview of the statistical considerations. It provides the general specifications for the analysis of the data to be collected and presented in the CSR. A final Statistical Analysis Plan (SAP) will be issued prior to database lockdown and before code breaking. The SAP will define all “pre-specified, planned analyses.”

All programming will be performed using SAS Version 9.4.

10.3.1 Analysis sets

(1) Full Analysis Set (FAS):

Based on the principles of the Intent-to-Treat (ITT) analysis, all randomized subjects, either treated with medication or with EVT will be included in the FAS. The primary efficacy endpoint analysis of this study will be performed on the FAS.

(2) Per Protocol Set (PPS)

The PPS is a subset that includes all subjects who were treated with the treatment assignment to which they were randomized and there are no clinically meaningful deviations from the protocol. Severe deviations from the protocol will be defined during the data auditing process, including but not limited to the following:

1) The subject is not in line with the inclusion criteria.

2) There are other treatments that potentially confound the appraisal of efficacy of the planned treatment.

3) Poor compliance.

4) Follow-up interval exceeds the required time window.

Secondary analysis will be conducted on the PPS. If its result are not consistent with that of the FAS, a detailed analysis to examine the difference(s) will be required.

(3) Safety Analysis Set (SAS)

The SAS consists of all subjects who received treatment with at least one evaluation of the safety outcome.

10.3.2 Statistical considerations

(1) Baseline characteristics comparisons

T-test or Wilcoxon rank sum test will be used for comparison between continuous variables, and Chi-squared tests, Fisher's exact test or Wilcoxon sum rank test will be used for comparison between categorical variables.

(2) Efficacy Analysis

Primary efficacy endpoint: Based on an ITT basis, an ordinal logistic regression model is used to calculate the common odds ratio between the two treatment groups. All statistics will be two-sided with $p<0.05$ considered significant.

Secondary Efficacy Analyses: Endpoints including the 90-day mRS 0-2 will be analyzed using a binary logistic regression model. The infarct core volume change from baseline will be analyzed using student t-test or Wilcoxon rank sum test as appropriate.

(3) Safety Analysis

Safety events in the two treatment groups will be described based on the SAS dataset. Logistic regression will be used to compare the differences in safety endpoints such as intracranial bleeding events between the two groups. Chi-square test and Fisher's exact test will be used to compare the differences in the incidence of AEs and SAEs between the two groups.

(4) Subgroup analysis

The mRS at 90 days will be presented for each level of the covariates listed below:

(1) Age (< 70 years vs. ≥70 years)

(2) Last known well to randomization time (< 6 h vs. ≥ 6 h)

(3) Stroke severity before randomization (NIHSS<16 vs. NIHSS≥16 points)

(4) IV thrombolysis or not

(5) Occlusion site (intracranial ICA vs. M1 segment)

(6) ASPECTS (< 3 points vs. ≥3 points)

(7) Infarct core volume (< 70ml vs. ≥70ml)

(8) Etiological subtype of stroke (cardiac embolism vs. large artery atherosclerosis)

10.4 Interim analysis

Interim analysis will take place when 1/2 (244 cases) and 3/4 (366 cases) have completed 3-month follow-up. The O'Brien-Fleming boundaries will be used at the interim analysis as follows:

There are no established techniques for the assessment of interim trial efficacy boundaries using an ordinal logistic regression model (proportional odds model). Instead, we will revert to a simple dichotomous analysis of the mRS score at 0-2 defined at 90 days from randomization. The Z-statistic for this analysis shall be derived from the normal approximation of the binomial distribution as an unadjusted two-sample test of proportions. For a RCT comparing two treatment groups with respect to a binary outcome and two interim analysis, corresponding significance levels based on the O'Brien & Fleming boundary are 0.003 (stage 1), 0.018 (stage 2) and 0.044 (stage 3, final analysis).

With the result of the interim analysis, the DSMB will make the decision to continue or halt the study according to the test boundaries. The study will stop prematurely for futility if the result from the interim analysis indicate that an effective conclusion with the current sample size can't be achieved. Premature stopping for early success will be achieved if the interim analysis result proves the effect of the intervention arm at significance level. Otherwise, the study will be continued until the predefined termination date. In interim analysis, the final sample size can be adjusted if the estimation of the primary outcome is drastically different from the actual data.

11. Ethical standards

11.1 Ethical standards

This Clinical Trial will be conducted in accordance with the principles set by the 18th World Medical Assembly (Helsinki, 1964) and all applicable amendments by the World Medical Assemblies and the ICH guidelines for Good Clinical Practice. Prior to initiating the study, each site will obtain Institutional review board (IRB) or institutional ethics committee (IEC) approval for the protocol, informed consent forms and material used to recruit subjects. Before each subject is enrolled, the investigator is responsible for fully and comprehensively introducing the purpose, procedures and possible risks of the study to the patient or his/her agent, signing a written informed consent form, and informing the patient that he/she has the right to withdraw from this study at any time. The informed consent should be kept as a clinical

study document for future reference. The personal privacy and data confidentiality of subjects will be protected during the study process.

11.2 Law and regulations

This Clinical Trial will be conducted in compliance with all international laws and regulations, Chinese laws and regulations, as well as any applicable guidelines.

11.3 Informed consent

The Investigator/sub-investigator should fully inform the patient of all pertinent aspects of the Clinical Trial, including the written information approved/preferred by the Ethics Committee (IRB/IEC). The Informed Consent Form used by the Investigator for obtaining the patient's informed consent must be reviewed and approved by the Sponsor and then submitted to the Ethics Committee (IRB/IEC) for approval.

All participants should be informed to the fullest extent possible about the study, in language and terms they are able to understand. Prior to a patient's participation in the Clinical Trial, an informed Consent Form should be signed and dated by the patient or by the patient's legal representative and by the person who conducted the informed consent discussion. A copy of the signed and dated Informed Consent Form will be provided to the patient.

11.4 Institutional review board/ Institutional ethics committee (IRB/IEC)

The Investigator or the Sponsor must submit this Clinical Trial Protocol to the appropriate Ethics Committee (IRB/IEC), and the Ethics Committee is required to forward to the Sponsor a copy of the written approval/favorable opinion signed and dated by the Chairman with Ethics Committee (IRB/IEC) composition.

The Clinical Trial (study number, Clinical Trial Protocol title and version number), the documents reviewed (Clinical Trial Protocol, Informed Consent Form, Investigator's Brochure, Investigator's CV, etc.), the list of voting members along with their qualification and the date of the review should be clearly stated on the written (IRB/IEC) approval.

During the Clinical Trial, any amendment or modification to the Clinical Trial Protocol

should be submitted to the Ethics Committee (IRB/IEC). It should also be informed of any event likely to affect the safety of patients or the continued conduct of the Clinical Trial, in particular any change in safety. All updates to the Investigator's Brochure will be sent to the Ethics Committee (IRB/IEC). If requested, an annual progress report, as well as final summary of the Clinical Trial's outcome at the end of the Clinical Trial, will also be sent to the Ethics Committee (IRB/IEC).

12. Confidentiality and publication of research findings

The principal investigator has complete intellectual property rights. The entire research process and data analysis process will strictly protect the patient's information. Publication of the results of this trial will be governed by the policies and procedures developed by the Executive Committee. The trial results will be published as soon as possible after database lockdown. This trial will produce detailed data on treatment effects, medical care, and outcomes. Biostatisticians will be consulted to ensure that it is impossible to uniquely identify any participant. Diskettes with the data in comma-delimited text format, along with a data dictionary in a text file, will be sent to interested parties.

13. Study Organization

13.1 Constitution

- **The steering committee**

  - ✓ The steering committee will provide scientific and strategic direction for the trial and will have overall responsibility for its design, execution, and publication.
  - ✓ The steering committee will also be responsible for ensuring that study execution and management are of the highest quality.

- ✓ It will approve the protocol and the operational guidelines of the trial prior to its commencement.
- ✓ The steering committee will meet regularly by teleconference or face-to-face meetings to discuss and report the progress of the study.
- ✓ The composition of the steering committee and its responsibilities are described in a charter which will be finalized before the start of the trial.

- **Executive committee**

The executive committee is responsible for reviewing the status of the trial and available blinded data and will take appropriate actions regarding the conduct of the study. Executive Committee meetings will be organized to make major decisions. The composition of the Executive Committee and its responsibilities are described in a charter which will be finalized before the start of the trial.

- **Data safety and monitoring board (DSMB)**

The DSMB will meet regularly and monitor the study progress to ensure that the study meets the highest standards of ethics and patient safety. It is composed of Academic Members, including an independent statistician, who does not participate in the trial. A DSMB charter including membership, role and responsibilities will be approved by both the DSMB and the Executive Committee before the start of the trial.

Written recommendations and their rationale will be provided to the Chairs of the Steering Committee immediately after each DSMB meeting.

- **Clinical event committee (CEC)**

Clinical events and safety endpoint will be reviewed by CEC. A CEC charter including membership, role and responsibilities will be approved before the start of the trial by the CEC and the Executive Committee.

- **Imaging assessment committee**

13.2 Site training and certification

The executive committee will provide training to their participating sites in Good Clinical Practice Guidelines and in some outcome assessments. Prior to initiation of patient enrollment, Site Investigators and Coordinators must complete all training programs.

The training programs that need to be completed are as follows:

(1) Study procedures
(2) ANGEL-ASPECT eligibility criteria
(3) mRS
(4) NIHSS
(5) ASPECTS
(6) iSchemaView automated RAPID® software
(7) eTICI
(8) TOAST etiology subtyping
(9) Heidelberg Bleeding Classification
(10) Collecting DICOM imaging data

Successful completion of the training program is mandatory before a site begins to enroll patients. A conference call will be held intermittently, and PI and key staff will be available to answer questions.

A detailed Manual of Procedures will serve as the primary document describing all study related procedures. It will serve as a guide to train clinical center personnel and will be updated periodically throughout the study on the ANGEL-ASPECT website, as needed. A system composed of members of the executive committee and CRA will be implemented for the clinical centers to ask any procedural questions by phone, fax, or e-mail. The ANGEL-ASPECT executive committee and monitoring committee will formulate answers in consultation with the Steering Committee and will periodically distribute to the participating centers a set of frequently asked questions and answers.

The members of the executive committee will manage and conduct site visits to ensure the integrity and validity of the data on the CRF. During the trial period, each site should be visited at least once. If there are data quality problems or recruitment problems, it should be visited as needed.

14. Study monitoring and quality assurance control

14.1 Responsibilities of the investigator(s)

The Investigator(s) should conduct the Clinical Trial in accordance with the Clinical Trial Protocol, The International Council for Harmonisation of Technical Requirements for Pharmaceutical for Human Use (ICH) guidelines for Good Clinical Practice and the applicable regulatory requirements.

The Investigator is required to ensure compliance with all procedures required by the Clinical Trial Protocol and with all study procedures provided by the Sponsor (including security rules). The Investigator should provide reliable data and all information requested by the Protocol (with the help of the CRF, Discrepancy Resolution Form or other appropriate instruments) in an accurate and legible manner and ensure direct access to source documents by Sponsor representatives.

The Investigator may appoint other individuals as Sub-Investigators, as he/she thinks appropriate. All Sub-Investigators shall be appointed and listed in a timely manner and will be supervised by the Investigator. The Investigator will provide them a copy of the Clinical Trial Protocol and all necessary information. The Sponsor is responsible for taking all reasonable steps to ensure the proper conduct of the Clinical Trial Protocol as regards to ethics, Clinical Trial Protocol compliance, integrity and validity of the data on the CRF.

14.2 Study monitoring

The main responsibility of the monitoring team is to help researchers to ensure that

all aspects of clinical trials are ethical, scientific, professional, and standardized. According to the ICH guidelines for Good Clinical Practice (GCP), the Monitoring Team must check the CRF entries according to the source documents, except for the pre-identified.

The monitoring team will regularly contact each center through site visits or an online webinar, and will send inspectors to evaluate the research progress, adherence of the investigators and patients to the research protocol and to solve urgent problems. During these inspection visits, the inspector will work together with the site-investigators. The main aspects of inspection and monitoring are as follows (not exclusive): patient's informed consent, patient recruitment and follow-up, documentation and reporting of SAEs and data quality.

15. Data retention

The double reviewed CRF and imaging data will be sent to the trial-designated data management center by CRAs. The person in charge of the data management center will check and sign the receipt form. The CRF will be kept by the research center after data entry is completed.

16. Data Security Monitoring

The data safety monitoring board (DSMB) is established to monitor the safety of participants, protect participants and ensure the integrity of the study. All AEs should be recorded, handled and tracked until they are properly resolved or stabilized. Any SAEs and unexpected events should be reported in a timely manner to the ethics committee in accordance with the relevant provisions, the competent department, the sponsor and the supervisory and administrative departments. The principal researchers should regularly review all AEs and set up meetings to assess the risks and benefits of the study if necessary. An independent data safety monitoring committee will be

appointed to review safety data, evaluate the effectiveness of data monitoring, and decide whether to make new proposal.

During the clinical trial, the data of the subjects should be collected anonymously in the CRF. The subjects are identified only by the subject number and the abbreviation of the initials. Due to safety reason and administrative instructions, when the subject's identity is leaked, researchers shall share the responsibility of confidentiality. In the informed consent form, the patient allows authorized research staff, ethics committee, and the authority to refer directly to the relevant original data on the case report (such as the patient's medical file case, booking records, the original laboratory records, etc.). The above personnel shall comply with occupational confidentiality rules and must keep all patient identity and medical information confidential.

17. Registration and Publication

17.1 Registration of study summary and results

The study representatives will register a study summary in ClinicalTrials.gov (https: //clinicaltrials.gov) before the start of the study and update the summary as appropriate according to changes in the protocol or progress of the study. When the study is completed, the study representatives will register a study result without delay.

17.2 Publication of study results

When the study is completed, the study database will be closed within one month after the last scheduled follow-up date of the last included patient. A manuscript which describes the study and the answer to the primary research question will be submitted to a major clinical journal within 3 months from closure of the database. The study representatives will publish the results of the study after taking necessary measures (e.g., to prevent identification of specific study patients) to protect the human rights of patients and related parties or the rights and benefits of patients and related parties.

The manuscript will be shared with the financial sponsor(s) one month before submission, but the financial sponsor(s) will have no influence on its contents. Author(s) of the paper are determined by the study representatives according to the Uniform Requirements for Manuscripts Submitted to Biomedical Journals (http: //www.icmje.org/) by the International Committee of Medical Journal Editors (ICMJE). All authors should review and agree to the details of the paper prior to submission. The same goes for authors of conference presentations.

18. Ownership and use of data

18.1 Ownership of the data

The results, data, intellectual property rights, etc. obtained in this study belong to the study representatives and not to the patients. Whether the intellectual property rights of the study representatives belong to the individual or to the study institution is determined by the agreement of the participating hospital.

18.2 Use of collected data

The study Steering Committee determines whether to use the data obtained in this study (hereinafter, "study data") for further study conducted by the Study representatives or sub-investigators as a secondary analysis.

If the analysis is judged to be beyond the scope of secondary analysis, or if the study data is used by a person except for the study representatives or sub-investigators of this study, the Study Steering Committee prepares a separate protocol and conducts the study after undergoing ethical review in accordance with relevant laws, regulations and ethical guidelines for medical research on human subjects.

19. Funding and conflict of interest

The study was funded by unrestricted grants from Covidien Healthcare

International Trading (Shanghai) Co., Ltd., Johnson & Johnson MedTech, Genesis MedTech（Shanghai）Co., Ltd. and Shanghai HeartCare Medical Technology Co., Ltd.

20. Reference

1. Powers WJ, Rabinstein AA, Ackerson T, et al. Guidelines for the Early Management of Patients With Acute Ischemic Stroke: 2019 Update to the 2018 Guidelines for the Early Management of Acute Ischemic Stroke: A Guideline for Healthcare Professionals From the American Heart Association/American Stroke Association. Stroke 2019;50(12):e344-e418. DOI: 10.1161/STR.0000000000000211.
2. Turc G, Bhogal P, Fischer U, et al. European Stroke Organisation (ESO)- European Society for Minimally Invasive Neurological Therapy (ESMINT) guidelines on mechanical thrombectomy in acute ischemic stroke. J Neurointerv Surg 2019;11(6):535-538. DOI: 10.1136/neurintsurg-2018-014568.
3. Berkhemer OA, Fransen PS, Beumer D, et al. A randomized trial of intraarterial treatment for acute ischemic stroke. N Engl J Med 2015;372(1):11-20. DOI: 10.1056/NEJMoa1411587.
4. Campbell BC, Mitchell PJ, Kleinig TJ, et al. Endovascular therapy for ischemic stroke with perfusion-imaging selection. N Engl J Med 2015;372(11):1009-18. DOI: 10.1056/NEJMoa1414792.
5. Goyal M, Demchuk AM, Menon BK, et al. Randomized assessment of rapid endovascular treatment of ischemic stroke. N Engl J Med 2015;372(11):1019-30. DOI: 10.1056/NEJMoa1414905.
6. Jovin TG, Chamorro A, Cobo E, et al. Thrombectomy within 8 hours after symptom onset in ischemic stroke. N Engl J Med 2015;372(24):2296-306. DOI: 10.1056/NEJMoa1503780.
7. Saver JL, Goyal M, Bonafe A, et al. Stent-retriever thrombectomy after intravenous t-PA vs. t-PA alone in stroke. N Engl J Med 2015;372(24):2285-95. DOI: 10.1056/NEJMoa1415061.
8. Nogueira RG, Jadhav AP, Haussen DC, et al. Thrombectomy 6 to 24 Hours after Stroke with a Mismatch between Deficit and Infarct. N Engl J Med 2018;378(1):11-21. DOI: 10.1056/NEJMoa1706442.
9. Albers GW, Marks MP, Kemp S, et al. Thrombectomy for Stroke at 6 to 16 Hours with Selection by Perfusion Imaging. N Engl J Med 2018;378(8):708-718. DOI: 10.1056/NEJMoa1713973.
10. Bracard S, Ducrocq X, Mas JL, et al. Mechanical thrombectomy after intravenous alteplase versus alteplase alone after stroke (THRACE): a randomised controlled trial. Lancet Neurol 2016;15(11):1138-47. DOI: 10.1016/S1474-4422(16)30177-6.
11. Gautheron V, Xie Y, Tisserand M, et al. Outcome After Reperfusion Therapies in Patients With Large Baseline Diffusion-Weighted Imaging Stroke Lesions: A THRACE Trial (Mechanical Thrombectomy After Intravenous Alteplase Versus Alteplase Alone After Stroke) Subgroup Analysis. Stroke 2018;49(3):750-753. DOI: 10.1161/STROKEAHA.117.020244.
12. Deb-Chatterji M, Pinnschmidt H, Flottmann F, et al. Predictors of independent outcome of thrombectomy in stroke patients with large baseline infarcts in clinical practice: a multicenter analysis. J Neurointerv Surg 2020;12(11):1064-1068. DOI: 10.1136/neurintsurg-2019-015641.

13. Roman LS, Menon BK, Blasco J, et al. Imaging features and safety and efficacy of endovascular stroke treatment: a meta-analysis of individual patient-level data. Lancet Neurol 2018;17(10):895-904. DOI: 10.1016/S1474-4422(18)30242-4.
14. Campbell BCV, Majoie C, Albers GW, et al. Penumbral imaging and functional outcome in patients with anterior circulation ischaemic stroke treated with endovascular thrombectomy versus medical therapy: a meta-analysis of individual patient-level data. Lancet Neurol 2019;18(1):46-55. DOI: 10.1016/S1474-4422(18)30314-4.
15. Sarraj A, Hassan AE, Savitz S, et al. Outcomes of Endovascular Thrombectomy vs Medical Management Alone in Patients With Large Ischemic Cores: A Secondary Analysis of the Optimizing Patient's Selection for Endovascular Treatment in Acute Ischemic Stroke (SELECT) Study. JAMA Neurol 2019;76(10):1147-1156. DOI: 10.1001/jamaneurol.2019.2109.
16. Cagnazzo F, Derraz I, Dargazanli C, et al. Mechanical thrombectomy in patients with acute ischemic stroke and ASPECTS </=6: a meta-analysis. J Neurointerv Surg 2020;12(4):350-355. DOI: 10.1136/neurintsurg-2019-015237.
17. Sarraj A, Grotta JC, Pujara DK, Shaker F, Tsivgoulis G. Triage imaging and outcome measures for large core stroke thrombectomy - a systematic review and meta-analysis. J Neurointerv Surg 2020;12(12):1172-1179. DOI: 10.1136/neurintsurg-2019-015509.
18. Rebello LC, Bouslama M, Haussen DC, et al. Endovascular Treatment for Patients With Acute Stroke Who Have a Large Ischemic Core and Large Mismatch Imaging Profile. JAMA Neurol 2017;74(1):34-40. DOI: 10.1001/jamaneurol.2016.3954.
19. Broocks G, Flottmann F, Schonfeld M, et al. Incomplete or failed thrombectomy in acute stroke patients with Alberta Stroke Program Early Computed Tomography Score 0-5 - how harmful is trying? European journal of neurology 2020;27(10):2031-2035. DOI: 10.1111/ene.14358.
20. Nicholson P, Hilditch CA, Neuhaus A, et al. Per-region interobserver agreement of Alberta Stroke Program Early CT Scores (ASPECTS). J Neurointerv Surg 2020;12(11):1069-1071. DOI: 10.1136/neurintsurg-2019-015473.
21. van Horn N, Kniep H, Broocks G, et al. ASPECTS Interobserver Agreement of 100 Investigators from the TENSION Study. Clin Neuroradiol 2021. DOI: 10.1007/s00062-020-00988-x.
22. Demeestere J, Garcia-Esperon C, Garcia-Bermejo P, et al. Evaluation of hyperacute infarct volume using ASPECTS and brain CT perfusion core volume. Neurology 2017;88(24):2248-2253. DOI: 10.1212/WNL.0000000000004028.
23. Sarraj A, Hassan AE, Grotta J, et al. Optimizing Patient Selection for Endovascular Treatment in Acute Ischemic Stroke (SELECT): A Prospective, Multicenter Cohort Study of Imaging Selection. Ann Neurol 2020;87(3):419-433. DOI: 10.1002/ana.25669.
24. Mourand I, Abergel E, Mantilla D, et al. Favorable revascularization therapy in patients with ASPECTS </= 5 on DWI in anterior circulation stroke. J Neurointerv Surg 2018;10(1):5-9. DOI: 10.1136/neurintsurg-2017-013358.
25. Inoue M, Olivot JM, Labreuche J, et al. Impact of diffusion-weighted imaging Alberta stroke program early computed tomography score on the success of endovascular reperfusion therapy. Stroke 2014;45(7):1992-8. DOI: 10.1161/STROKEAHA.114.005084.
26. Han M, Choi JW, Rim NJ, et al. Cerebral infarct volume measurements to improve patient selection for endovascular treatment. Medicine (Baltimore) 2016;95(35):e4702. DOI:

10.1097/MD.0000000000004702.
27. Manceau PF, Soize S, Gawlitza M, et al. Is there a benefit of mechanical thrombectomy in patients with large stroke (DWI-ASPECTS </= 5)? European journal of neurology 2018;25(1):105-110. DOI: 10.1111/ene.13460.
28. Broocks G, Rajput F, Hanning U, et al. Highest Lesion Growth Rates in Patients With Hyperacute Stroke. Stroke 2018:STROKEAHA118023457. DOI: 10.1161/STROKEAHA.118.023457.
29. Goyal M, Fargen KM, Turk AS, et al. 2C or not 2C: defining an improved revascularization grading scale and the need for standardization of angiography outcomes in stroke trials. J Neurointerv Surg 2014;6(2):83-6. DOI: 10.1136/neurintsurg-2013-010665.
30. von Kummer R, Broderick JP, Campbell BC, et al. The Heidelberg Bleeding Classification: Classification of Bleeding Events After Ischemic Stroke and Reperfusion Therapy. Stroke; a journal of cerebral circulation 2015;46(10):2981-6. DOI: 10.1161/STROKEAHA.115.010049.
31. Zaidat OO, Yoo AJ, Khatri P, et al. Recommendations on angiographic revascularization grading standards for acute ischemic stroke: a consensus statement. Stroke; a journal of cerebral circulation 2013;44(9):2650-63. DOI: 10.1161/STROKEAHA.113.001972.
32. Boers AM, Marquering HA, Jochem JJ, et al. Automated cerebral infarct volume measurement in follow-up noncontrast CT scans of patients with acute ischemic stroke. AJNR Am J Neuroradiol 2013;34(8):1522-7. DOI: 10.3174/ajnr.A3463.
33. Liu L, Chen W, Zhou H, et al. Chinese Stroke Association guidelines for clinical management of cerebrovascular disorders: executive summary and 2019 update of clinical management of ischaemic cerebrovascular diseases. Stroke Vasc Neurol 2020;5(2):159-176. DOI: 10.1136/svn-2020-000378.
34. EuroQol G. EuroQol--a new facility for the measurement of health-related quality of life. Health Policy 1990;16(3):199-208. DOI: 10.1016/0168-8510(90)90421-9.

21. Appendix

Appendix Table 1. Modified Rankin Scale

The modified Rankin Scale (mRS) is an ordinal scale ranging from 0 to 5, with higher scores indicating more severe disability. A score of 6 indicates death.

**Category**	**Short description**	**Long description**
0	No symptoms	No symptoms
1	Symptoms, no disability	Minor symptoms that do not interfere with lifestyle
2	Slight disability	Slight disability, symptoms that lead to some restriction in lifestyle, but do not interfere with the patient's capacity to look after himself.
3	Moderate disability	Moderate disability, symptoms that significantly restrict lifestyle and prevent totally independent existence
4	Moderately severe disability	Moderately severe disability, symptoms that clearly prevent independent existence though not needing constant attention
5	Severe disability	Severe disability, totally dependent patient requiring constant attention day and night.
6	Death	Death

Appendix Table 2. Extended Thrombolysis In Cerebral Ischemia (eTICI) Scale

**eTICI grade**	**Short description**	**Long description**
**0**	No perfusion	No antegrade flow beyond the point of occlusion
**1**	Limited reperfusion	Antegrade reperfusion past the initial occlusion, but limited distal branch filling with little or slow distal reperfusion
**2a**	<50% reperfusion	Antegrade reperfusion of less than half of the occluded target artery previously ischemic territory (eg, in 1 major division of the MCA and its territory)
**2b**	≥50% and <90% reperfusion	Antegrade reperfusion of more than half of the previously occluded target artery ischemic territory (eg, in 2 major divisions of the MCA and its territories)
**2c**	≥90% reperfusion	Near complete antegrade reperfusion of the previously occluded target artery ischemic territory, except for slow flow or distal emboli in a few distal cortical vessels
**3**	100% reperfusion	Complete antegrade reperfusion of the previously occluded target artery ischemic territory, with absence of visualized occlusion in all distal branches

MCA: middle cerebral artery; eTICI; extended thrombolysis in cerebral ischemia scale

Appendix Table 3. National Institute of Health Stroke Scale (NIHSS)

The NIHSS is an ordinal scale to evaluate the severity of stroke by assessing a patient's performance in the neurological exam. Scores range from 0 to 42, with higher scores indicating a more severe deficit. Administer stroke scale items in the order listed. Record performance in each category after each subscale exam. Do not go back and change scores. Follow directions provided for each exam technique. Scores should reflect what the patient does, not what the clinician thinks the patient can do. The clinician should record answers while administering the exam and work quickly. Except where indicated, the patient should not be coached (i.e. repeated requests to patient to make a special effort).

Instructions	Scale definition
**1a. Level of consciousness.** The investigator must choose a response if a full evaluation is prevented by such obstacles as an endotracheal tube, language barrier, orotracheal trauma/bandages. A 3 is scored only if the patient makes no movement (other than reflexive posturing) in response to noxious stimulation.	0 = Alert; keenly responsive.<br>1 = Not alert; but arousable by minor stimulation to obey, answer, or respond.<br>2 = Not alert; required repeated stimulation to attend, or is obtunded and requires strong or painful stimulation to make movements (not stereotyped).<br>3 = Responds only with reflex motor or autonomic effects or totally unresponsive, flaccid and areflexic.
**1b. LOC Questions:** The patient is asked the month and his/her age. The answer must be correct – there is not partial credit for being close. Phasic and stuporous patients who do not comprehend the questions will score 2. Patients unable to speak because of endotracheal intubation, orotracheal trauma, severe dysarthria from any cause, language barrier, or any other problem not secondary to aphasia are given a 1. It is important that only the initial answer be graded and that the examiners not "help" the patient with verbal or non-verbal clues.	0 = Answers both questions correctly.<br>1 = Answers one question correctly.<br>2 = Answers neither question correctly.
**1c. LOC Commands:** The patient is asked to open and close the eyes and then to grip and release the non-paretic hand. Substitute another one step command if the hand cannot be used. Credit is given if an unequivocal attempt is made but not completed due to weakness. If the patient does not respond to command, the task should be demonstrated to him or her (pantomime), and the result scored (i.e. follows none, one or	0 = Performs both tasks correctly.<br>1 = Performs one task correctly.<br>2 = Performs neither task correctly.

two commands). Patients with trauma, amputation, or other physical impediments should be given suitable one-step commands. Only the first attempt is scored.	
**2. Best Gaze:** Only horizontal eye movements will be tested. Voluntary or reflexive (oculocephalic) eye movements will be scored, but caloric testing is not done. If the patient has a conjugate deviation of the eyes that can be overcome by voluntary or reflexive activity, the score will be a 1. If a patient has an isolated peripheral nerve paresis (CN III, IV or VI), score a 1. Gaze is testable in all aphasic patients. Patients with ocular trauma, bandages, preexisting blindness, or other disorder of visual acuity or fields should be tested with reflexive movements, and a choice made by the investigator. Establishing eye contact and then moving about the patient from side to side will occasionally clarify the presence of a partial gaze palsy.	0= Normal.<br>1= Partial gaze palsy; gaze is abnormal in one or both eyes, but forced deviation or total gaze paresis is not present.<br>2= Forced deviation; or total gaze paresis not overcome by the oculocephalic maneuver.
**3. Visual:** Visual fields (upper and lower quadrants) are tested by confrontation, using finger counting or visual threat, as appropriate. Patients may be encouraged, but if they look at the side of the moving finger appropriately, this can be scored as normal. If there is unilateral blindness or enucleation, visual fields in the remaining eye are scored. Score 1 only if a clear-cut asymmetry, including quadrantanopia, is found. If patient is blind from any cause, score 3.<br>Double simultaneous stimulation is performed in this case.<br>If there is extinction, the patient receives a 1, and the results are used to respond to item 11.	0= No visual loss.<br>1= Partial hemianopia.<br>2= Complete hemianopia.<br>3= Bilateral hemianopia (blind including cortical blindness)
**4. Facial palsy:** Ask or use pantomime to encourage the patient to show teeth or raise eyebrows and close eyes. Score symmetry of grimace in response to noxious stimuli in the poorly response or non-comprehending patient. If facial trauma/bandages, orotracheal tube, tape or other physical barriers obscure the face, these should be removed to the extent possible.	0 = Normal symmetrical movements.<br>1= Minor paralysis (flattened nasolabial fold, asymmetry on smiling)<br>2= Partial paralysis (total or near-total paralysis of lower face)<br>3= Complete paralysis of one or both sides (absence of facial movement in the upper and lower face).
**5. Motor arm:** The limb is placed in the appropriate position: extend the arms (palms down) 90 degrees (if sitting) or 45 degrees (if supine). Drift is scored if the arm falls before 10 seconds. The aphasic patient is encouraged using urgency in the voice and pantomime, but not noxious stimulation. Each limb is tested in turn, beginning with the non-paretic arm. Only in the case of amputation or joint fusion at the shoulder, the examiner should record the score as untestable (UN), and	0= No drift; limb holds 90 (or 45) degrees for full 10 seconds.<br>1= Drift; limb holds 90 (or 45) degrees, but drifts down before full 10 seconds; does not hit bed or other support.<br>2= Some effort against gravity; limb cannot get to or maintain (if cued) 90 (or 45) degrees, drifts down to bed, but has some effort against

clearly write the explanation for this choice.	gravity.<br>3= No effort against gravity; limb falls.<br>4= No movement.<br>UN = Amputation or joint fusion: explain:<br>5a = Left Arm.<br>5b = Right arm.
**6. Motor leg:** The limb is placed in the appropriate position: hold the leg at 30 degrees (always tested supine). Drift is scored if the leg falls before 5 seconds. The aphasic patient is encouraged using urgency in the voice and pantomime, but not noxious stimulation. Each limb is tested in turn, beginning with the non-paretic leg. Only in the case of amputation or joint fusion at the hip, the examiner should record the score as untestable (UN), and clearly write the explanation for this choice.	0= No drift; leg holds 30-degree position for full 5 seconds.<br>1= Drift; leg falls by the end of the 5-second period but does not hit bed.<br>2= Some effort against gravity; leg falls to bed by 5 seconds, but has some effort against gravity.<br>3= No effort against gravity; leg falls to bed immediately.<br>4= No movement.<br>UN = Amputation or joint fusion: explain:<br>6a. Left Leg<br>6b. Right Leg.
**7. Limb ataxia:** This item is aimed at finding evidence of a unilateral cerebellar lesion. Test with eyes open. In case of visual defect, ensure testing is done in intact visual field. The finger-nose-finger and heel-shin tests are performed on both sides, and ataxia is scored only if present out of proportion to weakness. Ataxia is absent in the patient who cannot understand or is paralyzed. Only in the case of amputation or joint fusion, the examiner should record the score as untestable (UN), and clearly write the explanation for this choice. In case of blindness, test by having the patient touch nose from extended arm position.	0= Absent.<br>1= Present in one limb.<br>2= Present in two limbs.<br>UN = Amputation or joint fusion: explain:
**8. Sensory:** Sensation or grimace to pinprick when tested, or withdrawal from noxious stimulus in the obtunded or aphasic patient. Only sensory loss attributed to stroke is scored as abnormal and the examiner should test as many body areas (arms [not hands], legs, trunk, face) as needed to accurately check for hemisensory loss. A score of 2, 'severe or total sensory loss', should only be given when a severe or total loss of sensation can be clearly demonstrated. Stuporous and aphasic patients will, therefore, probably score 1 or 0. The patient with brainstem stroke who has bilateral loss of sensation is scored 2. If the patient does not respond and is quadriplegic, score 2.<br>Patients in a coma (item 1a=3) are automatically given a 2 on this item.	0= Normal; no sensory loss.<br>1= Mild-to-moderate sensory loss; patients feels pinprick is less sharp or is dull on the affected side; or there is a loss of superficial pain with pinprick, but patient is aware of being touched.<br>2= Severe to total sensory loss; patient is not aware of being touched in the face, arm and leg.

**9. Best language:** A great deal of information about comprehension will be obtained during the preceding sections of the examination. For this scale item, the patient is asked to describe what is happening in the attached picture, to name the items on the attached naming sheet and to read from the attached list of sentences. Comprehension is judged from responses here, as well as to all of the commands in the preceding general neurological exam. If visual loss interferes with the tests, ask the patient to identify objects placed in the hand, repeat, and produce speech. The intubated patient should be asked to write. The patient in a coma (item 1a=3) will automatically score 3 on this item. The examiner must choose a score for the patient with stupor or limited cooperation, but a score of 3 should be used only if the patient is mute and follows no one-step commands.	0= No aphasia; normal<br>1= Mild-to-moderate aphasia; some obvious loss of fluency or facility of comprehension, without significant limitation on ideas expressed or form of expression. Reduction of speech and/or comprehension, however, makes<br>conservation about provided materials difficult or impossible. For example, in conversation about provided materials, examiner can identify picture or naming card content from patient's response.<br>2= Severe aphasia; all communication is through fragmentary expression; great need for inference, questioning, and guessing by the listener. Range of information that can be exchanged is limited; listener carries burden of communication. Examiner cannot identify materials provided from patient response.<br>3 = Mute, global aphasia: no usable speech or auditory comprehension.
**10. Dysarthria:** If patient is thought to be normal, an adequate sample of speech must be obtained by asking patient to read or repeat words from the attached list. If the patient has severe aphasia, the clarity of articulation of spontaneous speech can be rated. Only if patient is intubated or has other physical barriers to producing speech, the examiner should record the score as untestable (UN), and clearly write an explanation for this choice. Do not tell the patient why he or she is being tested.	0= Normal.<br>1= Mild-to-moderate dysarthria; patient slurs at least some words and, at worst, can be understood by some difficulty.<br>2= Severe dysarthria: patient's speech is so slurred as to be unintelligible in the absence of or out of proportion to any dysphasia, or is mute/anarthric.<br>UN = Intubated or other physical barrier.
**11. Extinction and Inattention** (formerly Neglect): Sufficient information to identify neglect may be obtained during the prior testing. If the patient has a severe visual loss preventing visual double simultaneous stimulation, and the cutaneous stimuli are normal, the score is normal. If the patient has aphasia but does appear to attend to both sides, the score is normal. The presence of visual spatial neglect or anosagnosia may also be taken as evidence of abnormality. Since the abnormality is scored only if present, the item is never untestable.	0= No abnormality.<br>1= Visual, tactile, auditory, spatial, or personal inattention or extinction to bilateral simultaneous stimulation in one of the sensory modalities.<br>2= Profound hemi-inattention or extinction to more than one modality; does not recognize own hand or orients to only one side of space.

Appendix Table 4. EuroQoL 5D-5L

Under each heading, please tick the ONE box that best describes your health TODAY.

Mobility

I have no problems in walking about ☐
I have slight problems in walking about ☐
I have moderate problems in walking about ☐
I have severe problems in walking about ☐
I am unable to walk about ☐

Self-care

I have no problems washing or dressing myself ☐
I have slight problems washing or dressing myself ☐
I have moderate problems washing or dressing myself ☐
I have severe problems washing or dressing myself ☐
I am unable to wash or dress myself ☐

Usual activities (e.g. work, study, housework, family or leisure activities)

I have no problems doing my usual activities ☐
I have slight problems doing my usual activities ☐
I have moderate problems doing my usual activities ☐
I have severe problems doing my usual activities ☐
I am unable to do my usual activities ☐

Pain/discomfort

I have no pain or discomfort ☐
I have slight pain or discomfort ☐
I have moderate pain or discomfort ☐
I have severe pain or discomfort ☐
I have extreme pain or discomfort ☐

Anxiety/depression

I am not anxious or depressed ☐
I am slightly anxious or depressed ☐
I am moderately anxious or depressed ☐
I am severely anxious or depressed ☐
I am extremely anxious or depressed ☐

- We would like to know how good or bad your health is TOADY.
- This scale is numbered from 0 to 100.
- 100 means the best health you can imagine.
  0 means the worst health you can imagine.
- Mark an X on the scale to indicate how your health is TODAY.
- Now, please write the number you marked on the scale in the box below.

YOUR HEALEH TOADY = ☐

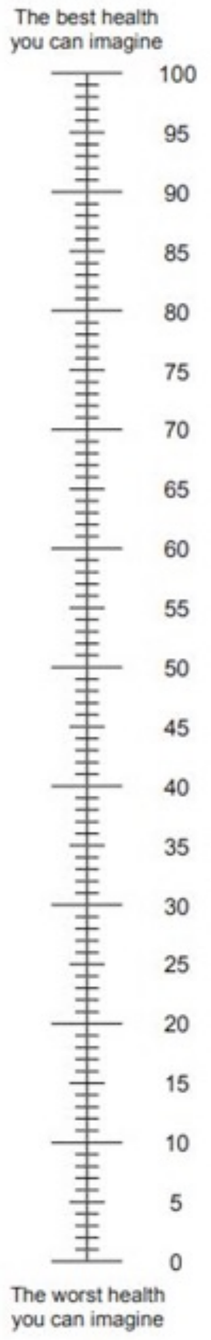

Appendix Table 5. Heidelberg bleeding classification

Symptomatic intracranial hemorrhage (SICH): new intracranial hemorrhage detected by brain imaging associated with any of the items below:

1. ≥4 points decline in the total NIHSS at the time of diagnosis compared to immediately before worsening. Note that a 4 point change is not compared with the baseline admission NIHSS score but instead to the immediate predeterioration neurological status

2. ≥2 point decline in one NIHSS category. The rationale for this is to capture new hemorrhages that produce new neurological symptoms, making them clearly symptomatic but not causing worsening in the original stroke territory. For example, a new remote hemorrhage in the contralateral occipital lobe may cause new hemianopia that is clearly symptomatic but the patient will not have worsening of ≥4 points on the NIHSS score

Leading to intubation/hemicraniectomy/EVD placement or other major medical/surgical intervention.

3. Absence of alternative explanation for deterioration

Anatomic Description of Intracranial Hemorrhages

Class	Type	Description
1	Hemorrhagic transformation of infarcted brain tissue	
1a	HI1	Scattered small petechiae, no mass effect
1b	HI2	Confluent petechiae, no mass effect
1c	PH1	Hematoma within infarcted tissue, occupying <30%, no substantive mass effect
2	Intracerebral hemorrhage within and beyond infarcted brain tissue	
	PH2	Hematoma occupying 30% or more of the infarcted tissue, with obvious mass effect
3	Intracerebral hemorrhage outside the infarcted brain tissue or intracranial-extracerebral hemorrhage	
3a		Parenchymal hematoma remote from infarcted brain tissue
3b		Intraventricular hemorrhage
3c		Subarachnoid hemorrhage
3d		Subdural hemorrhage

HI indicates hemorrhagic infarction; and PH, parenchymatous hematoma.

Appendix Table 6. Alberta Stroke Program Early CT Score (ASPECTS)

The Alberta Stroke Program Early CT Score (ASPECTS) is a semiquantitative method of estimation of infarct size with non-contrast CT during the acute phase. The territory of the middle cerebral artery is allotted 10 points. 1 point is subtracted for an area of early ischaemic change, such as focal swelling, or parenchymal hypoattenuation, for each of the defined regions. A normal CT scan has an ASPECTS value of 10 points. A score of 0 indicates diffuse ischaemia throughout the territory of the middle cerebral artery.

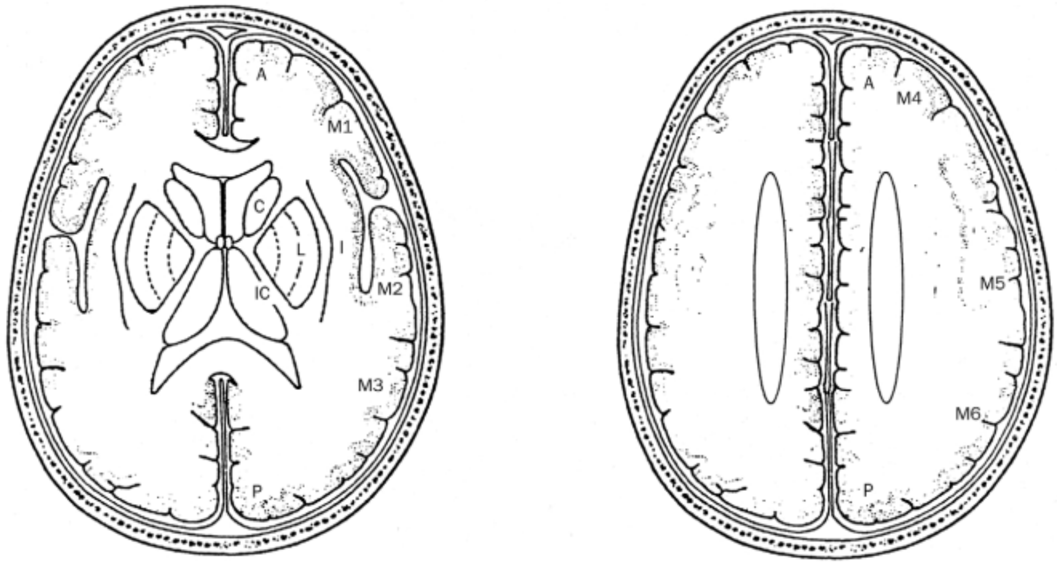

A=anterior circulation; P=posterior circulation; C=caudate; L=lentiform; IC=internal capsule; I=insular ribbon; MCA=middle cerebral artery; M1=anterior MCA cortex; M2=MCA cortex lateral to insular ribbon; M3=posterior MCA cortex; M4, M5, and M6 are anterior, lateral, and posterior MCA territories immediately superior to M1, M2, and M3, rostral to basal ganglia.

Subcortical structures are allotted 3 points (C, L, and IC). MCA cortex is allotted 7 points (insular cortex, M1, M2, M3, M4, M5, and M6).

Study of Endovascular Therapy in Acute Anterior Circulation Large VeSsel Occlusive Patients with a LargE InfarCT Core: A Multicenter, Prospective, Open-Label, Blinded-Endpoint, Randomized Controlled Trial (ANGEL-ASPECT)

Protocol

Research team: Beijing Tiantan Hospital, Capital Medical University, Beijing, China

Principal Investigator: Zhongrong Miao, MD, Professor of Neurology

Co- Principal Investigator: Zeguang Ren, Vitor Mendes Pereira

Protocol Version 7.1

Trial Registration: ClinicalTrials.gov NCT04551664

Date: May 18, 2021

Table of Contents

Protocol Signature Page 70

ANGEL-ASPECT Protocol Synopsis 71

Abbreviations 77

1. Background 79

1.1 The rational of EVT for large infarct core volume 79

1.2 Image modality to identify large infarct core volume 81

1.3 ASPECTS and infarct core volume selection 82

1.4 EVT time window for large infarct core volume 82

1.5 ANGEL-ASPECT Trial design 83

2. Study objective 84

2.1 Primary objective 84

2.2 Secondary objective 84

3. Study design 84

3.1 Study design 85

3.2 Randomization 85

3.3 Follow-up schedule 85

3.4 Blind design 86

4. Participant selection 86

4.1 Inclusion Criteria 86

4.1.1 Center Inclusion Criteria 86

4.1.2 Clinical Inclusion Criteria 86

4.1.3 Neuroimaging Inclusion Criteria 87

4.2 Exclusion Criteria 87

4.2.1 Center Exclusion Criteria 87

4.2.2 Clinical Exclusion Criteria 87

4.2.3 Neuroimaging Exclusion Criteria 88

5. Imaging protocol 88

5.1 Baseline imaging 88

5.2 Intraoperative and follow-up imaging: 89

5.3 Imaging core lab 91

6. Treatments 91

6.1 Endovascular Therapy (EVT) 91

6.2 Best Medical Management (BMM) 92

7. Study endpoints 93

7.1 Primary efficacy endpoint 93

7.2 Secondary efficacy endpoint 93

7.3 Primary safety endpoint 93

7.4 Secondary safety endpoint 93

8. Data collection and Study procedure 94

8.1 Screening and Inclusion 94

8.2 Data to be collected during procedure 96

8.3 Post Treatment (Through Hospital Discharge) 96

8.4 Follow-up Visit at Day 30 (±3), Day 90 (±7) and 12 Months (±14 days) 97

8.5 Unscheduled Follow-up Visit 97

8.6 Schedule of activities and assessments 98

9. Study risk pre-assessment and risk management 99

9.1 Monitoring of adverse events 99

9.2 Definitions of adverse events 99

9.2.1 Adverse event (AE) 99

9.2.2 Serious adverse event (SAE) 99

9.3 Recording of adverse events 100

9.4 Causal relationship between adverse events and study: 100

9.5 Obligation of the investigator regarding safety reporting 100

9.5.1 Adverse events 100

9.5.2 Serious adverse event 100

9.5.3 Follow-up and risk management 101

10. Statistical Analysis 101

10.1 Sample size estimation 101

10.2 Data collection and entry 102

10.2.1 Paper-based CRF filled out by investigator 102

10.2.2 Data entry to the EDC system by CRC 102

10.2.3 Submission to the EDC system after the approval of investigator......102
10.2.4 Data monitoring and query by CRA via EDC......103
10.2.5 Data exportation from the EDC system......103
10.3 Statistical considerations......103
10.3.1 Analysis sets......103
10.3.2 Statistical considerations......104
10.4 Interim analysis......105
11. Ethical standards......106
11.1 Ethical standards......106
11.2 Law and regulations......107
11.3 Informed consent......107
11.4 Institutional review board/institutional ethics committee (IRB/IEC)......107
12. Confidentiality and publication of research findings......108
13. Study Organization......108
13.1 Constitution......108
13.2 Site training and certification......110
14. Study monitoring and quality assurance control......112
14.1 Responsibilities of the investigator(s)......112
14.2 Study monitoring......112
15. Data retention......113
16. Data Security Monitoring......113
17. Registration and Publication......114
17.1 Registration of study summary and results......114
17.2 Publication of study results......114
18. Ownership and use of data......115
18.1 Ownership of the data......115
18.2 Use of collected data......115
19. Funding and conflict of interest......115
20. Reference......115
21. Appendix......119
Appendix Table 1. Modified Rankin Scale......119
Appendix Table 2. Extended Treatment In Cerebral Ischemia (eTICI) Scale......120

Appendix Table 3. National Institute of Health Stroke Scale (NIHSS) 121
Appendix Table 4. EuroQoL 5D-5L...125
Appendix Table 5. Heidelberg bleeding classification..127
Appendix Table 6. Alberta Stroke Program Early CT Score (ASPECTS).........................128

Protocol Signature Page

I have read this protocol and agree to adhere to the requirements.

By signing this document we confirm that the clinical study will be conducted in accordance with the protocol and all applicable laws and regulations including, but not limited to, the International Conference on Harmonisation Guideline for Good Clinical Practice (GCP) and the ethical principles that have their origins in the Declaration of Helsinki.

____

Clinical Site

______________________________	________________________
Site Principal Investigator Signature	Date

ANGEL-ASPECT Protocol Synopsis

**Official Title**		Study of Endovascular Therapy in Acute Anterior Circulation Large Vessel Occlusive Patients with a Large Infarct Core: A Multicenter, Prospective, Open-Label, Blinded-Endpoint, Randomized Controlled Trial
**Acronym**		ANGEL-ASPECT
**Sponsor**		Beijing Tiantan Hospital, Capital Medical University
**Study Centers**		~50 centers in China
**Statement of Hypothesis**		Best medical management (BMM) combined with endovascular Therapy (EVT) might be superior to BMM alone in acute anterior circulation large vessel occlusive (LVO) patients with a large infarct core.
**Study Objectives**	**Primary objective**	To estimate if acute ischemic stroke patients with anterior circulation LVO and large infarct core at 0-24 hours after stroke onset have improved neurological functional outcomes when treated with BMM plus EVT compared to BMM alone.
	**Secondary objective**	To estimate if acute ischemic stroke patients with anterior circulation LVO and large infarct core at 0-24 hours after stroke onset have increased the risk of symptomatic intracranial hemorrhage (sICH) when treated with BMM plus EVT compared to BMM alone.
**Study settings**		Multicenter, Prospective, Randomized, Open-label, Blinded End-point (PROBE) design
**Randomization**		Participants will be randomized in a 1:1 ratio based on simple randomization of the central network

		randomization system to receive BMM plus EVT or BMM alone.
**Sample Size**		A total of 502 patients are planned to be enrolled. Interim analysis will take place when 1/3 (168 cases) and 2/3 (336 cases) have completed 3-month follow-up.
**Effective Endpoints**	**Primary Endpoint**	90 days (±7 days) modified Ranking Scale (mRS)
	**Secondary Endpoints**	(1) 90 days (±7 days) mRS 0-2<br>(2) 90 days (±7 days) mRS 0-3<br>(3) 36 hours (±12 hours) NIHSS 0-1 or decrease ≥10 from baseline<br>(4) Infarct core volume change from baseline, at 7 days (±1 day) or at discharge assessed with NCCT or at 36 hours (±12 hours) assessed with MRI<br>(5) 36 hours (±12 hours) target artery recanalization rate assessed with CTA or MRA
**Safety Endpoints**	**Primary Safety Endpoint**	Rate of sICH within 48 hours from randomization (Heidelberg Bleeding Classification)
	**Secondary Safety Endpoints**	(1) All-cause mortality within 90 days (±7 days)<br>(2) Any intracranial hemorrhage within 48 hours from randomization (Heidelberg Bleeding Classification)<br>(3) Decompressive hemicraniectomy during hospitalization
**Participants**	**Inclusion Criteria**	**Center Inclusion Criteria**

		(1) Equipped with emergency department and neurology department for stroke patients<br>(2) Equipped with stroke team operating on 24/7<br>(3) Capable of EVT and intravenous (IV) thrombolysis for acute ischemic stroke patients<br>**Clinical Inclusion Criteria:**<br>(1) Age 18-80 years<br>(2) Presenting with symptoms consistent with acute ischemic stroke<br>(3) Pre-stroke mRS score 0-1<br>(4) NIHSS score 6-30 at the time of randomization<br>(5) Randomization can be finished within 24 hours from stroke onset (stroke onset time is defined as last known well time)<br>(6) Informed consent signed<br>**Neuroimaging Inclusion Criteria:**<br>(1) CTA or MRA proved occlusion of Internal Carotid Artery (ICA) terminus or M1 segment of Middle Cerebral Artery<br>(2) Imaging evidence of low Alberta Stroke Program Early CT Score (ASPECTS) (based on non-contrast CT) or large infarct Core (defined as rCBF <30% on CT perfusion or ADC<620 on MRI) fill one of the following criteria:<br>1） ASPECTS 3–5<br>2） ASPECTS >5 (6 h-24 h) with infarct core volume 70-100 ml<br>3） ASPECTS <3 with infarct core volume 70-100 ml

	**Exclusion Criteria**	**Center Exclusion Criteria**<br>(1) Centers in which the number of acute ischemic stroke cases treated with endovascular procedures are less than 20 per year;<br>(2) Incapable of complying with the protocol to proceed with the research.<br>**Clinical Exclusion Criteria**<br>(1) Females who are pregnant, or those of childbearing, potential with positive urine or serum beta Human Chorionic Gonadotropin test<br>(2) Known severe allergy (more severe than skin rash) to contrast agents uncontrolled by medications<br>(3) Refractory hypertension that is difficult to be controlled by drugs (defined as persistent systolic blood pressure >185 mmHg or diastolic blood pressure >110 mmHg)<br>(4) Known hemorrhagic tendency (including but not limited to): Baseline platelet count $< 100\times10^9$/L; Heparin was administered within 48 hours with aPTT≥35s; on anticoagulant therapy with warfarin and International Normalized Ratio (INR) > 1.7 (Patients with no history or suspected coagulopathy do not need to wait for laboratory results of INR or aPTT prior to enrollment)<br>(5) Parenchymal organ surgery and biopsy were performed in the past one month<br>(6) Any active bleeding or recent bleeding (gastrointestinal bleeding, urinary bleeding, etc.) in the past one month

		(7) Undergoing hemodialysis or peritoneal dialysis; Known severe renal insufficiency with glomerular filtration rate <30 ml/min or serum creatinine >220 mmol/L (2.5mg/dl)<br>(8) Brain tumor (with mass effect)<br>(9) The expected survival time is less than 1 year (such as comorbidity with malignant tumor, serious heart and lung diseases, etc.)<br>(10) Participation in other interventional randomized clinical trials that may confound the outcome assessment of the trial<br>(11) Other circumstances that the investigator considers inappropriate for participation in the trial or that may pose significant risks to patients (such as inability to understand and/or follow the study procedures and/or follow up due to mental disorders, cognitive or emotional disorders)<br>**Neuroimaging Exclusion Criteria**<br>(1) Midline shift or herniation, mass effect with effacement of the ventricles<br>(2) Evidence of acute intracranial hemorrhage<br>(3) Acute bilateral strokes or multiple intracranial vessels occlusions
**Treatment Allocation**	**Study Arm**	BMM plus EVT
	**Control Arm**	BMM alone
**Follow-up schedule**		Study visits will take place on day of randomization, at 36 hours (± 12 hours), 7 days (±1 day)/at discharge

	which is earlier, 30 days (±3 days), 90 days (±7 days) and 12 months (± 14 days).
**Subgroup analysis**	Subgroup analysis will be performed based on the following variables:<br>(1) Age (< 70 years vs. ≥70 years)<br>(2) Weak-up stroke or not<br>(3) Last known well to randomization time (< 6 h vs. ≥ 6 h)<br>(4) Stroke severity before randomization (NIHSS<16 points vs. NIHSS≥16 points)<br>(5) IV thrombolysis or not<br>(6) Occlusion site (intracranial ICA vs. M1 segment)<br>(7) Ipsilateral carotid artery occlusion or not<br>(8) ASPECT score (< 3 points vs. ≥3 points)<br>(9) Infarct core volume (< 70ml vs. ≥70ml)<br>(10) Etiological subtype of stroke (cardiac embolism vs. large artery atherosclerosis)
**Study duration**	August 2020 to October 2023 (enrolment completed at October 2022)

Abbreviations

ADC	Apparent Diffusion Coefficient
AE	Adverse Event
aPTT	Activated Partial Thromboplastin Time
ASPECTS	Alberta Stroke Program Early CT Score
BMM	Best Medical Management
CEC	Clinical Events Adjudication Committee
CI	Confidence Interval
CRA	Clinical Research Associate
CRC	Clinical research coordinator
CRF	Case Report Form
CSA	Chinese Stroke Association
CT	Computer Tomography
CTA	Computed Tomography Angiography
CTP	Computed Tomography Perfusion Imaging
DAWN	DWI or CTP Assessment with Clinical Mismatch in the Triage of Wake-Up and Late Presenting Strokes Undergoing Neurointervention with Trevo
DEFUSE 3	Endovascular Therapy Following Imaging Evaluation for Ischemic Stroke 3
DICOM	Digital Imaging and Communications in Medicine
DSA	Digital Subtraction Angiography
DSMB	Data Safety Monitoring Board
DWI	Diffusion Weighted Imaging
ECG	Electrocardiogram
EDC	Electronic Data Capture
EQ-5D-5L	EuroQoL 5-Dimensions 5-Level questionnaire
eTICI	Expanded Thrombolysis in Cerebral Infarction
EVT	Endovascular Therapy
FAS	Full Analysis Set
FLAIR	FLuid Attenuated Inversion Recovery
GCP	Good Clinical Practice
GRE	Gradient Recalled Echo
GSR-ET	German Stroke Registry – Endovascular Treatment
ICA	Internal Carotid Artery
ICH	The International Council for Harmonisation of Technical Requirements for Pharmaceutical for Human Use
ICMJE	International Committee of Medical Journal Editors
IEC	Institutional Ethics Committee
INR	International Normalized Ratio

IRB	Institutional Review Board
ITT	Intention-To-Treat
IV	Intravenous
LICV	Large Infarct Core Volume
LLC	Limited Liability Company
LVO	Large Vessel Occlusive
MCA	Middle Cerebral Artery
MM	Medical Management
MRA	Magnetic Resonance Angiography
MRI	Magnetic Resonance Imaging
mRS	Modified Rankin Scale
NCCT	Non-contrast computed tomography
NCSS	Number Cruncher Statistical System
NIHSS	National Institute of Health stroke scale
NMPA	National Medical Products Administration
OR	Odd Ratio
PASS	Power Analysis and Sample Size
PPS	Per Protocol Set
PROBE	Prospective, Randomized, Open-label, Blinded End-point
PWI	perfusion weighted imaging
SAE	Serious Adverse Event
SAP	Statistical Analysis Plan
SAS	Safety Analysis Set
SELECT	Optimizing Patient's Selection for Endovascular Treatment in Acute Ischemic Stroke
sICH	Symptomatic intracranial hemorrhage
THRACE	Mechanical thrombectomy after intravenous alteplase versus alteplase alone after stroke
TICI	Thrombolysis In Cerebral Infarction
T-NICE	Tiantan Neuroimaging Center of Excellence
TOAST	Trial of ORG 10172 in Acute Stroke Treatment

1. Background

Large clinical trials on early and late window stroke patients have helped to establish the indications for endovascular treatment (EVT) of acute ischemic stroke (AIS) patients with large vessel occlusion (LVO). [1,2] This includes Alberta Stroke Program Early CT Scores (ASPECTS) score ≥ 6 patients within 6 hours,[3-7] and patients meeting DWI or CTP Assessment with Clinical Mismatch in the Triage of Wake-Up and Late Presenting Strokes Undergoing Neurointervention with Trevo (DAWN) and Endovascular Therapy Following Imaging Evaluation for Ischemic Stroke (DEFUSE 3) trial imaging criteria patient at 6-16 or 24 hours.[8,9] Since then, many clinical trials have undergone an expanding the indications of EVT for AIS patients with LVO. Whether patients with large infarct core volume (LICV) are suitable for EVT is one of the unanswered questions.

1.1 The rational of EVT for large infarct core volume

Several retrospective studies, prospective studies, and meta-analyses suggest that patients with LICV may benefit from EVT. The Mechanical thrombectomy after intravenous alteplase versus alteplase alone after stroke (THRACE) trial was one of these early randomized trials to enroll patients with ASPECTS<6.[10] A subgroup analysis of THRACE trial showed among 53 subjects with a diffusion-weighted imaging (DWI) volume of >70 ml, 12 (22.6%) patients of the EVT group had good clinical outcomes (mRS ≤ 2 at 90 days). [11] The prospective German Stroke Registry – Endovascular Treatment (GSR-ET) also showed that 22% of 152 thrombectomy patients with ASPECTS <6 achieved independence with mRS 0-2 at 90 days.[12] The Highly Effective Reperfusion evaluated in Multiple Endovascular Stroke Trials (HERMES) collaboration pooled the data from the six trials. It showed a benefit of EVT over control was observed in patients with ASPECTS 0–4 or DWI-determined infarct core volume ≥ 70 ml. Functional improvement (mRS 0-2 at 90 days) rates in the EVT group compared with the control group were 25% vs. 14% and 30% vs.20%,

respectively.[13,14]

In the Optimizing Patient's Selection for Endovascular Treatment in Acute Ischemic Stroke (SELECT) trial, the prespecified secondary analysis of 105 patients (of whom 62 received EVT) with ASPECTS ≤ 5 or CTP-determined ischemic core volume ≥ 50 ml showed that functional independence was achieved in 31% in the EVT group vs. 14% in the control group.[15] Incidence rates of death, neurologic decline, and symptomatic intracerebral hemorrhage (sICH) were similar in both groups. In addition, EVT was also associated with less infarct growth (44 vs. 98 mL; p=0.006) and smaller final infarct volume (97 vs. 190 mL; p=0.001) than medical management (MM).

One meta-analysis including 17 studies and 1378 patients with ASPECTS 0–6 (1194 EVT, 184 MT) found that mRS 0–2 was achieved in 30.1% of cases after EVT and in 3.2% after MM (OR 4.76, p=0.01).[16] The marked lower rate in the MM group compared to previous RCTs (HERMES: 14%, SELECT: 14%) is likely due to the imbalance of baseline characteristics of the patients in these retrospective studies . for example, the patients in MM group were older (75 years vs 68.7 years), had higher NIHSS scores (19 vs. 18), lower rate of intravenous (IV) thrombolysis (47.8% vs. 56.8%) and longer symptoms onset to admission time (130 min vs. 115 min). Successful recanalization (Thrombolysis in Cerebral Infarction (TICI) grade 2b–3) gave higher odds of mRS 0–2 than unsuccessful reperfusion (OR 5.2, p=0.001). Another pooled random-effect meta-analysis, including 12 studies of large core patients (ASPECTS <6 or ischemic core volume ≥ 50 ml), demonstrated increased functional independence (mRS 0–2) rates with EVT (25% vs 7%; pooled OR: 4.39, 95% CI: 2.53 to 7.64), and decreased mortality (23% vs. 33%; pooled OR: 0.53, 95% CI: 0.40 to 0.71).[17]

In a matched case-control study of 56 patients (28 pairs) with ICA, M1 and M2 occlusion and CTP-determined infarct core > 50mL, EVT led to higher rates of functional independence (90-day mRS 0-2, 25% vs 0%; p=0.04), and smaller final infarct volumes (87 vs 242 mL; p <0.001).[18] One control (4%) and two treatment patients (7%) developed a parenchymal hematoma type 2 (p>0.99). The rates of hemicraniectomy (7% vs 21%; p=0.10) and 90-day mortality (29% vs 48%; p=0.75)

were lower in the EVT arm. Sensitivity analysis for patients with a baseline infarct core volume greater than 70 mL (12 pairs) revealed a significant reduction in final infarct volumes (110 vs. 319 mL; p<0.001) but only a nonsignificant improvement in the overall distribution of mRS scores favoring the treatment group (p=0.18).

Interestingly, one observational cohort study included a consecutive sample of 170 patients with anterior circulation stroke and initial ASPECTS ≤ 5 (99 patients in the EVT group, 71 patients in the MM group). The study showed that clinical outcome after failed or incomplete EVT (TICI 0–2b) was significantly better compared to patients with MM only (median mRS 5, interquartile range 4–6 vs. 5–6, p=0.03). Failed EVT (TICI 0–2a) was not associated with a worse outcome than MM.[19]

1.2 Image modality to identify large infarct core volume

Generally speaking, there were two imaging evaluation methods for large infarct core, one is a semi-quantitative evaluation based on CT/MRI-ASPECTS, and the other was a quantitative evaluation based on CTP/MRI with the aid of automated artificial intelligence software. ASPECTS is a widely accepted tool used to assess infarct volume. In general, ASPECTS < 6 is regarded as a "large core infarct." However, multiple studies have shown low interrater agreement with ASPECTS.[20,21] An inaccurate ASPECTS can mis-assign patients between the EVT and control groups, weakening any trial conclusions. Quantitative determination of infarct core volume using CTP/MRI could compensate for poor consistency of ASPECTS.

Notably, the correlation between the CTP/MRI-determined infarct core volume and ASPECTS is not well established. Therefore, the optimal imaging modality for evaluating patients with LICV in clinical trials remains to be explored. The subgroup analysis in a meta-analysis comparing outcomes between these two imaging modalities did not find significant heterogeneity in the results when LICV was defined based on ASPECTS or ischemic core volume of CTP.[17] While one study[22] found a good correlation between ASPECTS and CTP/MRI volume, others found them to be discordant.[15,17,23] To expedite enrollment, this study allowed the use of non-contrast CT

(NCCT)-ASPECTS and/or CTP/MRI imaging modalities to screen patients with LICV.

1.3 ASPECTS and infarct core volume selection

A recent meta-analysis of 17 studies and 1378 patients reported mRS 0-2 was achieved by 37.7%, 33.3%, 22.1%, and 17.1% of patients with ASPECTS 6, 5, 4 and 0-4 respectively.[16] The studies by Mourand et al.[24] and Inoue et al.[25] showed favorable outcomes in between 16% and 20% of patients with ASPECTS 0–3 after EVT. Another meta-analysis showed that ASPECT 0-2 favored MM instead of EVT.[13] The benefit of EVT gradually declined with decreasing in ASPECTS, especially when ASPECTS < 3, because the infarct core volume was very large and there was less salvageable brain tissue, which might make EVT ineffective.[13,26,27] Therefore, this study limited the ASPECTS of enrolled patients to 3-5.

There is some debate about whether “large core” should be defined as 50ml vs. 70ml on CTP. In ANGEL-ASPECT, the infarct core volume > 70 ml was defined as LICV. Similar to patients with ASPECTS 0-2, patients with excessive infarct core volume may also be less likely to benefit from EVT. Previous studies showed a lack of benefit if CTP-determined core volume exceeded 100 ml and 150 ml.[14,15] Therefore, when patients were enrolled only based on the infarct core volume as assessed by CTP/MRI, this study limited the infarct core volume to 70 ml-100 ml.

1.4 EVT time window for large infarct core volume

Patients in the hyperacute phase of stroke showed increased ASPECTS lesion growth from imaging to recanalization, suggesting a benefit of faster recanalization in these patients.[28] A meta-analysis by Cagnazzo et al. demonstrated that a shorter time from onset to reperfusion was associated with a higher probability of functional independence after EVT in patients with ASPECTS 0–6.[16] The SELECT trial found that patients with LICV had a gradual decline in functional outcomes with prolonged treatment time, with a lower likelihood of benefit from EVT after 12 hours.[15] This

suggested that for LICV patients, the earlier EVT may be more beneficial. However, a recent meta-analysis found that patients with LICV did not show a significant difference in outcomes among studies reporting <6 hours, <12 hours, and <24 hours for stroke to EVT time windows.[17] This may be because most LICV patients presented in the early time window, reducing the power to detect the difference between early and late windows. The reason may also be that the efficacy of MM declines over time, thus preserving the efficacy of EVT. In this context, it is important to study whether EVT also benefits patients with LICV in the late time window, therefore the time window of ANGEL-ASPECT is 0-24 hours.

1.5 ANGEL-ASPECT Trial design

ANGEL-ASPECT trial is a PROBE study initiated by researchers to explore the effectiveness and safety of EVT in patients with anterior circulation large vessel occlusion with ASPECTS 3-5 or infarction core volume 70-100ml within 24 hours. ANGEL-ASPECT trial allows multiple imaging modalities to screen for LICV patients, but at the same time imposes certain limitations on the range of ASEPCTS or infarct core volume. The main purpose was to reduce the risk of EVT while enrolling as many LICV patients as possible. The primary image inclusion criteria of ANGEL-ASPECT was NCCT-ASPECTS 3-5, and the infarct core volume 70 ml-100 ml was used as auxiliary inclusion criteria. Briefly, the inclusion criteria for LICV are: (1) If NCCT-ASPECTS is 3-5 and presentation is within 24 hours of onset, patients are enrolled without limitation of infarct core volume. (2) For NCCT-ASPECTS 0-2 and core infarction volume 70 ml-100 ml, patients are enrolled. (3) If NCCT-ASPECTS is >5 and between 6 to 24 hours from symptom onset, only patients with infarct core volume 70 ml-100 ml are enrolled.

Subgroup analysis will focus on age, LKW to randomization time, NIHSS score, IV thrombolysis, occlusion site, ASPECTS, infarct core volume, and stroke etiology.

ANGEL-ASPECT is the only randomized controlled trial conducted in China for LICV patients so far. The results of this trial will clarify whether EVT is effective and

safe in Chinese LICV patients.

2. Study objective

2.1 Primary objective

To estimate if acute ischemic stroke patients with anterior circulation LVO and large infarct core at 0-24 hours after stroke onset have improved neurological functional outcomes when treated with BMM plus EVT compared to BMM alone.

2.2 Secondary objective

To estimate if acute ischemic stroke patients with anterior circulation LVO and large infarct core at 0-24 hours after stroke onset have increased the risk of sICH when treated with BMM plus EVT compared to BMM alone.

3. Study design

3.1 Study design

Multicenter, Prospective, Randomized, Open-label, Blinded End-point (PROBE) trial design (Figure 1).

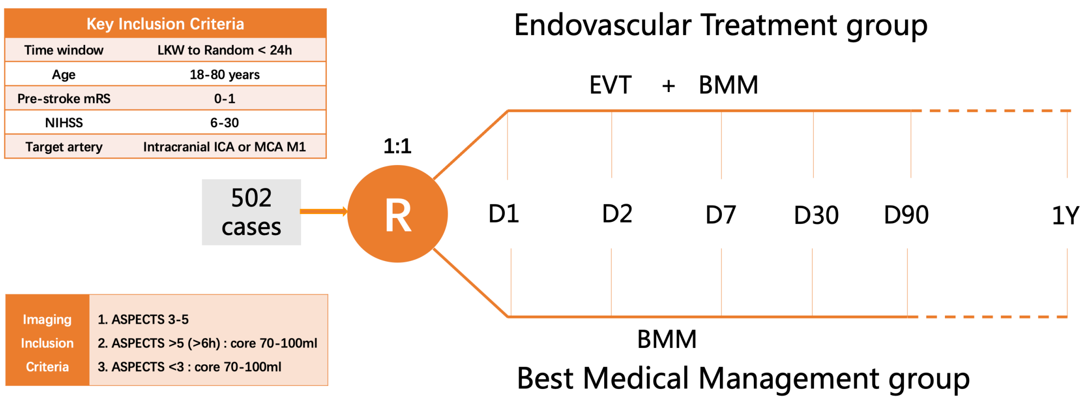

Figure 1. Study design: randomization algorithm

3.2 Randomization

The random code will be generated by a central network randomization system with 24h real-time online service based on the simple randomization method. The researcher in each center will obtain the random code from the central network randomization system according to the enrollment order. Patients who meet the inclusion criteria and in whom written informed consent can be obtained, will be randomly assigned to the following treatment groups in a 1:1 ratio:

- BMM plus EVT group: patients will receive EVT with stent retriever or contact aspiration as first-line devices for thrombectomy plus BMM;
- BMM group: Patients will receive BMM alone.

3.3 Follow-up schedule

(1) Face-to-face visit: day of randomization, 36 hours (±12 hours), 7 days (±1 day) /at discharge whichever is earlier

(2) Telephone visit: 30 days (±3 days), 90 days (±7 days), and 12 months ±14 days

3.4 Blind design

(1) Only the patient and the treating physician are aware of the randomization

information, and the evaluation of information at baseline and in-hospital visits that relate to the study endpoints should be evaluated by an investigator who is not aware of the patient group assignment and the treatment received.

(2) The primary endpoint visits are standardized visits conducted by trained third party personnel who are not aware of the patient's randomization assignment and their actual treatment status. All follow-up calls were recorded, and a follow-up report was formed.

(3) All imaging data related to the study will be collected for central interpretation. The images at each visit site were interpreted independently by a core lab, and the readers were unaware of the patient's baseline information, treatment received (except EVT angiography images), and prognosis.

4. Participant selection

4.1 Inclusion Criteria

4.1.1 Center Inclusion Criteria

(1) Equipped with an emergency department and neurology department for stroke patients

(2) Equipped with a stroke team operating 24/7

(3) Capable of endovascular therapy and IV thrombolysis for acute ischemic stroke patients

4.1.2 Clinical Inclusion Criteria

(1) 18 to 80 years of age

(2) Presenting with symptoms consistent with an acute ischemic stroke

(3) Pre-stroke mRS score 0-1

(4) NIHSS score 6-30 at the time of randomization

(5) Randomization can be finished within 24 hours of stroke onset (stroke onset time is defined as last known well time)

(6) Informed consent signed by the patient or legally authorized representative

4.1.3 Neuroimaging Inclusion Criteria

(1) CTA or MRA proven occlusion of the Internal Carotid Artery (ICA) terminus or M1 segment of the Middle Cerebral Artery (MCA)

(2) Imaging evidence of low ASPECTS (based on NCCT) or large infarct Core (defined as rCBF <30% on CT perfusion or ADC<620 on MRI) fulfill one of the following criteria:

   4) ASPECTS 3-5

   5) ASPECTS >5 (6h-24 h) with infarct core volume 70-100 ml

   6) ASPECTS <3 with infarct core volume 70-100 ml

4.2 Exclusion Criteria

4.2.1 Center Exclusion Criteria

(1) Centers in which the number of acute ischemic stroke cases treated with endovascular procedures are less than 20

(2) Centers that are unable to comply with the research protocol

4.2.2 Clinical Exclusion Criteria

(1) Females who are pregnant, or those of childbearing potential with positive urine or serum beta Human Chorionic Gonadotropin test

(2) Known severe allergy (more than a rash) to contrast media uncontrolled by medication

(3) Refractory hypertension that is difficult to control by medication (defined as persistent systolic blood pressure >185 mmHg or diastolic blood pressure >110 mmHg)

(4) Known hemorrhagic tendency (including but not limited to): Baseline platelet count $< 100\times10^9$/L; Heparin was administered within 48 hours with aPTT≥35s; on anticoagulant therapy with warfarin and International Normalized Ratio (INR) > 1.7 (Patients with no history or suspected coagulopathy do not need to wait for laboratory results of INR or aPTT prior to enrollment)

(5) Parenchymal organ surgery and biopsy were performed in the past one month

(6) Any active bleeding or recent bleeding (gastrointestinal bleeding, urinary bleeding, etc.)

in the past one month

(7) Undergoing hemodialysis or peritoneal dialysis; Known severe renal insufficiency with glomerular filtration rate <30ml/min or serum creatinine >220mmol/L (2.5mg/dl)

(8) Brain tumor (with mass effect)

(9) The expected survival time is less than 1 year (such as comorbidity with malignant tumor, advanced heart or lung disease, etc.)

(10) Participation in another interventional randomized clinical trials that may confound outcome assessment of the study

(11) Other circumstances that the investigator considers inappropriate for participation in the study or that may pose significant risks to patients (such as inability to understand and/or follow the study procedures and/or follow up due to mental disorders, cognitive or emotional disorders)

4.2.3 Neuroimaging Exclusion Criteria

(1) Midline shift or herniation, mass effect with effacement of the ventricles

(2) Evidence of acute intracranial hemorrhage

(3) Acute bilateral strokes or multiple intracranial vessel occlusion

5. Imaging protocol

5.1 Baseline imaging

All researchers were trained in the course of the imaging protocol and the use of RAPID software, and participated in the network training, simulation test and examination of NCCT-ASPECTS before enrollment. The ASPECTS training and test are conducted through the online training system of the trial website (http://angel-aspect.org). Those who pass the exam (accuracy rate more than 80%) will obtain the ASPECTS assessment qualification certificate and be qualified for imaging assessment. During imaging screening, researchers in the sub-center with imaging evaluation qualifications and two trained neuroradiologists from the trial team will conduct real-

time online image evaluation of ASPECTS, occlusion site, infarct core volume to ensure the accuracy of the imaging assessment (Figure 2).

(1) **ASPECTS:** All patients presenting within 24h of symptom onset will undergo a plain CT scan. After the preliminary screening of ASPECTS by trained clinicians in research centers, two dedicated clinicians (insert initials here / names) from the trial team will conduct real-time online evaluation of the prospective patient. When the ASPECTS score reaches a consensus that is between 3 to 5 between the site and central core lab investigators, the patient is then deemed suitable for enrollment into ANGEL-ASPECT. NCCT-ASPECTS will be manually determined independently before RAPID ASPECTS® (version 5.0.4, iSchemaView, CA, USA) assessment.

(2) **Infarct core volume:** The infarct core volume was automatically evaluated by iSchemaView automated RAPID® software (version 5.0.4, iSchemaView, CA, USA), and the infarction core volume was defined as rCBF<30% based on CTP or ADC<620 $\times 10^{-6}$ mm^2/s based on MRI. For patients who present with NCCT-ASPECTS 0-2 within 6 hours from symptom onset, if the infarct core volume is between 70ml and 100ml, then the patient is eligible for enrollment. If the infarct core volume is between 70ml and 100ml in an extend time window (6-24 hours) of stroke onset, the patient is also suitable for inclusion regardless of ASPECTS.

(3) **Target occlusion vascular:** The occluded arterial was determined by CTA or MRA. Occlusion of the ICA or M1 segment of the MCA is suitable for enrollment. Patients with ipsilateral extracranial internal carotid artery occlusion or stenosis with the above artery occlusion will also be included in this trial. As a tandem lesion can be difficult to distinguish on CTA or MRA, patients with tandem lesion in the EVT group will be confirmed during angiography.

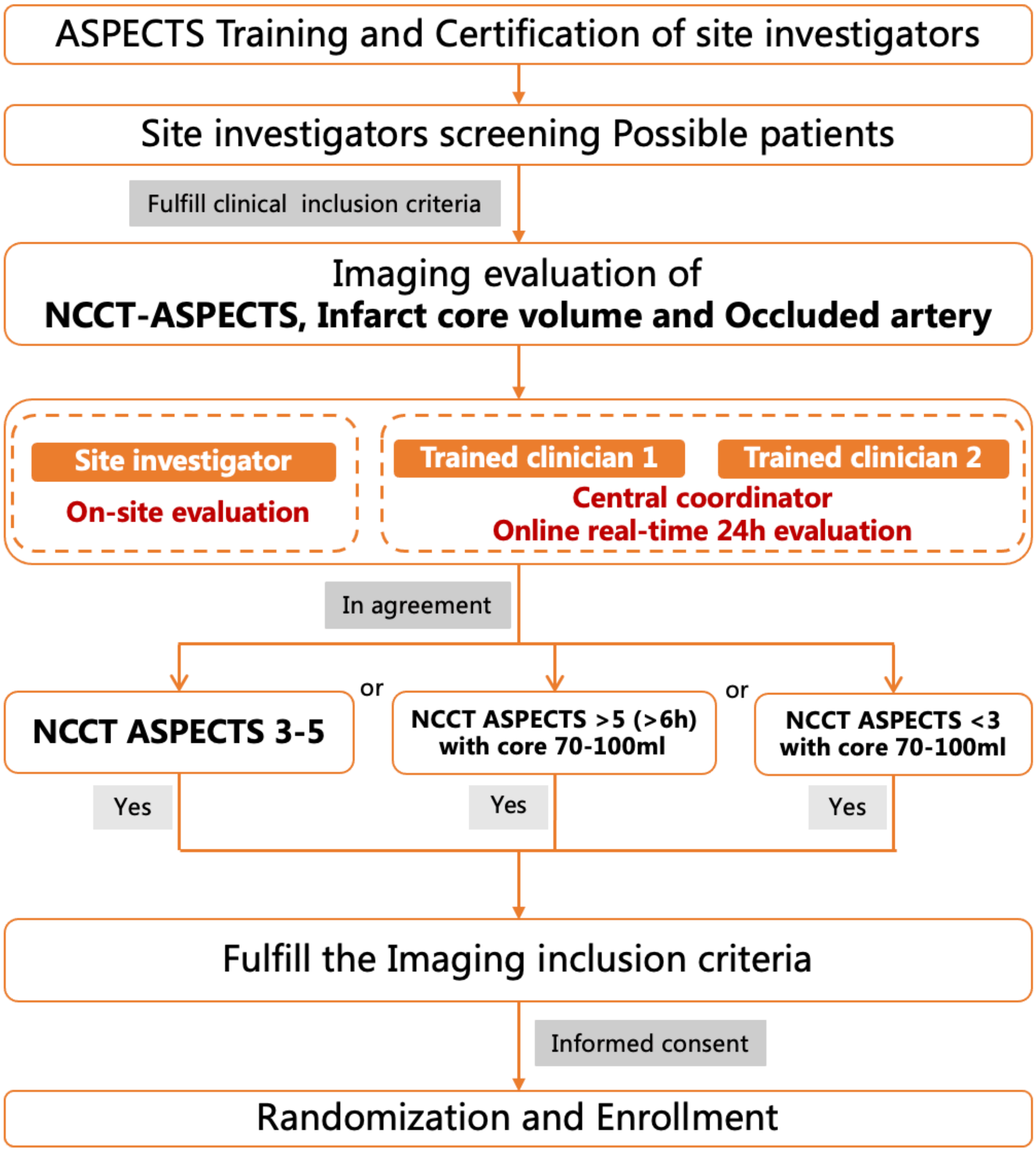

Figure 2. Imaging evaluation working flow

5.2 Intraoperative and follow-up imaging:

(1) **Intraoperative imaging:** In the EVT group, preoperative DSA was required to determine the site of vascular occlusion and eTICI score,[29] and postoperative angiography was required to evaluate the eTICI score. It is recommended to conduct NCCT or cone-beam CT immediately after EVT to exclude bleeding.

(2) **Imaging evaluation of hemorrhage:** The NCCT scan 36 hours (±12 hours) after randomization was taken as the main criterion for the judgment of hemorrhage, and

Heidelberg Bleeding Classification was used to evaluate the classification of hemorrhage.[30]

(3) **Vascular imaging follow-up:** Vascular imaging (CTA/MRA) was conducted with 36 hours (±12 hours) h after randomization to determine vascular patentability.[31]

(4) **Evaluation of postoperative infarct volume:** The infarct core volume was determined at 7 days (±1 day) or at discharge assessed with NCCT or at 36 hours (±12 hours) assessed with MRI, and the infarct core volume was determined manually by imaging core lab using validated automated software.[32]

5.3 Imaging core lab

Tiantan Neuroimaging Center of Excellence (T-NICE) is the imaging core lab of this trial. During the period from onset to discharge, all imaging data (CT, CTA, CTP, MRI, MRA, PWI, DSA) were collected by CRO in DICOM format. T-NICE will conduct quality control. All images will be quality controlled, rendered anonymous and sent to T-NICE for central adjudication. The final results will be reviewed and confirmed by the imaging assessment committee, and then input into the database system.

6. Treatments

Patients meeting the eligibility criteria and signing the informed consent will be randomized. Patients randomized to the interventional arm will receive BMM plus EVT. Artery puncture should be performed within 1 hour of randomization. Patients randomized to the medical arm will receive BMM.

6.1 Endovascular Therapy (EVT)

When the patient's condition permits, local anesthesia is the first choice for rapid initiation of puncture and endovascular therapy. If the condition requires, sedation can be used, and intubation can be considered for patients at high risk of airway collapse.

If the patient is expected to have poor intraoperative cooperation even with sedation or is at high risk of using sedation or airway conditions due to the patient's illness, general anesthesia should be used. Return to the Neuro-Intensive Care Unit (NICU) with intubation or not should be determined according to the surgical results.

Systemic heparinization is not recommended for preoperative and intraoperative treatment. Femoral artery is suggested for arterial puncture, and long sheath, guiding catheter or balloon guiding catheter can be used. Stent retriever (Solitaire, EMBOTRAP, Reco, Captor or other first-line stent retriever systems) and/or contact aspiration (Penumbra aspiration system or other first-line aspiration system) are recommended as the first choice for thrombectomy. If successful reperfusion (eTICI 2b50-3) is not achieved after routine thrombectomy, other techniques are allowed for rescue treatment, including replacement of thrombectomy technique, replacement of thrombectomy device, intra-arterial thrombolysis, balloon angioplasty or stent implantation, etc. The need for rescue treatment is defined (including, but not limited to, a decision made by the investigator based on intraoperative conditions) as follows: three times of thrombectomy with the same thrombectomy device (stent or suction catheter) without successful recanalization; Target vessels were successfully recanalized and reoccluded again; Target vessel dissection or stenosis ≥70%, with any degree of forward blood flow disturbance; New lumen/stent thrombosis resulted in a decreased of eTICI score.

All the above operations should be performed using devices approved by the National Medical Products Administration (NMPA) and should be performed in accordance with the approved intended use and operating instructions.

6.2 Best Medical Management (BMM)

All enrolled patients should receive BMM in accordance with the recommendation of "Chinese Stroke Association guidelines for clinical management of cerebrovascular disorders" by Chinese Stroke Association (CSA).[33] This includes IV thrombolysis therapy for patients meeting the guidelines. Patients who plan to undergo or are undergoing IV thrombolysis therapy can decide whether to terminate IV thrombolysis

therapy in advance according to the investigator's judgment after enrollment. Patients who had completed IV thrombolysis prior to randomization are also eligible for inclusion in this study. All patients will be required to record the name, dosage and time of IV thrombolysis medication in detail. Antiplatelet agents are not recommended within 24 hours after IV thrombolysis unless the patient has undergone balloon dilatation or stent implantation, at which time the antithrombotic strategy is determined by the investigator. Based on the time window and infarct core volume for ANGEL-ASPECT, it is anticipated that most patients enrolled in ANGEL-ASPECT will not have received IV thrombolysis prior to randomization. Non-IV thrombolysis patients will be treated with aspirin, unless an indication for early anticoagulation is present.

7. Study endpoints

7.1 Primary efficacy endpoint

90 days (±7 days) modified Ranking Scale (mRS)

7.2 Secondary efficacy endpoint

(1) 90 days (±7 days) mRS 0-2
(2) 90 days (±7 days) mRS 0-3
(3) 36 hours (±12 hours) NIHSS 0-1 or decrease ≥10 from baseline
(4) Infarct core volume change from baseline, at 7 days (±1 day) or at discharge assessed with NCCT or at 36 hours (±12 hours) assessed with MRI
(5) 36 hours (±12 hours) target artery recanalization rate assessed with CTA or MRA

7.3 Primary safety endpoint

Rate of sICH within 48 hours from randomization (Heidelberg Bleeding Classification, Appendix 5)

7.4 Secondary safety endpoint

(1) All-cause mortality within 90 days (±7 days)
(2) Any intracranial hemorrhage within 48 hours from randomization (Heidelberg Bleeding Classification)
(3) Decompressive hemicraniectomy during hospitalization

8. Data collection and Study procedure

Investigator(s) should keep a record, the eligibility Case Report Form (CRF), of subjects who enter pre-study screening. The sub-center number must be indicated. The screening table will be used to analyze and determine whether the enrolled patients in different study sites are representative. Referring to the procedure manual and data collection guidelines, investigators should guarantee the input of CRF is precise, complete and timely, and answer the queries in time. Brain imaging which includes: CT, CTA, CTP, MRI (T1+T2+DWI+FLAIR+ADC+GRE-T2*/SWI+MRA±PWI) and DSA will be collected as DICOM format. Laboratory results will be collected in photocopies of the reports.

8.1 Screening and Inclusion

- Basic data collection: hospital name, name of patient identification, age, sex, allergy to contrast agent.
- History of present event: time of onset (time of last known well), time of arrival at the hospital, type of onset, IV thrombolysis after onset (initial time, name and dose of the drug).

- Medical history and medication before onset

Medical histories (smoking, drinking, hypertension, diabetes, dyslipidemia, cardiac arrhythmia, valvular heart disease, cardiac insufficiency, coronary atherosclerotic heart disease, peripheral arterial disease, TIA, cerebral infarction, cerebral hemorrhage, intracranial tumors), pre-stroke mRS;

Combination therapy: Antiplatelet drugs (aspirin, clopidogrel, cilostazol, prasugrel, ticagrelor, ticlopidine, etc.), anticoagulants (warfarin, dabigatran, rivaroxaban, apixaban, edoxaban, heparin, argatroban, etc.), statins (atorvastatin, pitavastatin, rosuvastatin, pravastatin, simvastatin, fluvastatin, etc.).

- Physical examination

Height and weight, blood pressure, pulse, neurological evaluation (NIHSS and Glasgow scale), 12-lead electrocardiogram (ECG) .

- Emergency laboratory examinations

Emergency blood routine, emergency renal function, emergency liver function (transaminase), emergency coagulation, random blood glucose, etc.

- Imaging

A head CT is required to rule out hemorrhage and perform ASPECTS evaluation; a CTA or MRA is essential to confirm the occlusion artery; a CTP or MRI is required to calculate the infarct core volume

- All participants or his/her need to sign a written informed consent form
- Included subjects will be randomized

8.2 Data to be collected during procedure

- General anesthesia with intubation or conscious sedation with local anesthesia at start of procedure
- Procedure times: groin puncture time, time of each pass finish, time of initial flow restoration, time of successful recanalization and the end of the procedure
- eTICI score: pre-procedure and post-procedure
- Details of procedure: Accessory and adjunctive devices used (guide catheter, guidewire, intermediate catheter, microcatheter), number and type of devices for thrombectomy, number of recanalization attempt deployments, rescue procedures with medications besides thrombectomy
- Medication during the procedure: heparin, tirofiban, alteplase, urokinase.
- Intraprocedural complications: Presence of vasospasm (time of onset, vessels involved, time resolved, treatment required), evidence of clot migration or embolization, dissections, perforations, etc.

8.3 Post Treatment (Through Hospital Discharge)

A face-to-face observation will be performed at 36 hours (±12 hours) and 7 days (± 1 day)/at discharge which is earlier after randomization.

- Brain imaging performed at 36 (±12) hours after randomization: CT/CTA or MRI/MRA.
- A physical exam, as well as clinical and neurological assessments, will be completed at 36 (±12) hours and 7 (±1) days/at discharge which is earlier after randomization. Data to be collected include: vital signs (blood pressure and heart rate), relevant concomitant medications (including antiplatelet, anticoagulant, and antihypertensive agents), significant findings from clinical assessment and physical exam (i.e. all new, worsening, or improved conditions), all significant neurological findings, NIHSS Score (both), mRS (7 days or discharge only), and adverse event (AE).
- Head CT evaluation is needed at 7 days (±1 day) or at discharge whichever is earlier

- Laboratory examinations will be collected at 24 hours (±12 hours) and 7 days (± 1 day) or at discharge whichever is earlier after randomization, including: blood routine, renal function, liver function, coagulation, fasting blood glucose, etc.

8.4 Follow-up Visit at Day 30 (±3), Day 90 (±7) and 12 Months (±14 days)

These follow-up evaluations can be performed via telephone if it's not convenient for an in-person visit at the investigational site. All subjects entered into the study will undergo a standard neurological assessment by experienced physicians who are blinded to treatment assignment. Data to be collected include:

- mRS
- Patient-reported functional health status and quality of life using EuroQoL 5-Dimensions 5-Level questionnaire (EQ-5D-5L)[34]
- Relevant concomitant medications
- Significant findings from clinical assessment and physical exam (i.e. all new, worsening, or improved conditions since discharge)

8.5 Unscheduled Follow-up Visit

If an unscheduled follow-up visit occurs after randomization at the investigational site during the study, the incidence of any new or unresolved AEs will be assessed. If the visit is due to a change in neurological status, NIHSS and mRS will be completed by a certified rater.

8.6 Schedule of activities and assessments

Measurements	Baseline	36-hour (±12 hours) visit	7±1 day /at discharge visit	30-day (±3 days) visit	90 days (±7 days) visit	12-month ±14 days visit
Informed Consent	x					
Inclusion/Exclusion	x					
Randomization	x					
Demographic characteristics	x					
History of present illness	x					
Past medical history	x					
Relevant Concomitant Medications	x	x	x	x	x	x
mRS	x		x	x	x	x
NIHSS	x	x	x			
Head CT	x		x[3]			
CTA±CTP or MRI*+MRA±PWI	x[1]					
CT+CTA or MRI*+SWI+MRA		x[2]				
Carotid CTA/MRA/ultrasound		x[4]				
ASPECTS on CT	x					
Ischemic volume on CTP/DWI	x	x[5]	x[5]			
Laboratory examinations	x	x	x			
Electrocardiogram	x					
TOAST			x			
EQ-5D-5L scale				x	x	x
AE/SAE		x	x	x	x	x

[1] For all enrolled cases, CT+CTA+CTP examination was the first choice before randomization

[2] All the enrolled cases should be reviewed with multi-mode imaging 24-48 hours after randomization, and the image evaluation method should be the same as before randomization

[3] All enrolled cases should be examined by head CT at 7 days (±1 day) after randomization or at discharge which is earlier

[4] It was only applicable to patients in the standard medical treatment group who did not undergo cervical angiography prior to randomization

[5] Based on head CT or MRI, determined by the core imaging laboratory

*MRI sequence includes T1+T2+DWI+ADC+FLAIR sequence

ADC: apparent diffusion coefficient; AE: adverse event; ASPECTS: Alberta stroke program early computed tomography score; CT: computed tomography; CTA: computed tomography angiography; CTP: computed tomography perfusion; EQ-5D-5L: EuroQoL 5-Dimensions 5-Level questionnaire; FLAIR: fluid attenuated inversion recovery; MRA: magnetic resonance angiography; MRI: magnetic resonance imaging; mRS: modified Rankin scale; NIHSS: National Institutes of Health Stroke Scale; PWI: perfusion weighted imaging; SAE: serious adverse event; SWI: susceptibility weighting imaging; TOAST: Trial of ORG 10172 in Acute Stroke Treatment.

9. Study risk pre-assessment and risk management

9.1 Monitoring of adverse events

All AEs will be managed and reported in compliance with all applicable regulations and will be included in the final Clinical Study Report (CSR).

9.2 Definitions of adverse events

9.2.1 Adverse event (AE)

Adverse Events, as long as they occur from the first visit planned in the Clinical Trial Protocol/signature of the informed consent (i.e., occurring during the washout period) to the last visit planned in the protocol, are adverse medical events or deterioration of qualifying event. AEs include symptoms (ie, nausea, chest pain), signs (ie, tachycardia, liver enlargement) and abnormal laboratory results (ie, laboratory or ECG abnormalities). AEs can be classified as serious adverse events (SAEs) and non-serious AEs.

9.2.2 Serious adverse event (SAE)

A Serious adverse event is refers to：

- Results in death, or
- Is life-threatening, or

Note: The term "life-threatening" in the definition of "serious" refers to an event in which the patient is at risk of death at the time of the event; it does not refer to an event which hypothetically might have caused death if it was more severe.

- Requires inpatient hospitalization or prolongation of existing hospitalization, or
- Results in persistent or significant disability/incapacity, or
- Is a congenital anomaly/birth defect, or
- Is a medically important event

9.3 Recording of adverse events

Non-serious AE: Only some of the non-serious AEs will be collected from time of randomization throughout the treatment/follow-up periods to the Study Closure Visit. Other non-serious AEs are up to the investigator to decide whether to collect.

SAE: All SAEs will be collected and recorded.

9.4 Causal relationship between adverse events and study:

Attribution of: (1) Definite; (2) Probably; (3) Possibly; (4) Unlikely; (5) Not related; (6) Not applicable.

9.5 Obligation of the investigator regarding safety reporting

9.5.1 Adverse events

All AEs will be recorded on the corresponding page(s) in the CRF. Whenever possible, symptoms should be grouped as a single syndrome or diagnosis. The Investigator should specify the date of onset, intensity, action taken with respect to Investigational Product, corrective treatment/therapy given, outcome and his/her opinion about whether it is possible that the AE is caused by the study intervention, related to the index stroke, other cause, or intercurrent condition.

9.5.2 Serious adverse event

For SAEs, the investigator must immediately take corresponding measures:

Immediately notify the representative of the Monitoring Team, send the signed and dated corresponding pages of in the CRF to the representative of the Monitoring Team, and attach a photocopy of all examinations conducted and the examination dates. For laboratory results, include the laboratory normal ranges. The contact information (name, address and fax number) of the representative is on the Clinical Trial Protocol. These measures should be completed no later than **24 hours** after SAE.

Care should be taken to ensure that the patient's identity is protected and the patient's identifiers in the Clinical Trial are properly noted on all copies of source documents provided to the Sponsor.

9.5.3 Follow-up and risk management

The Investigator should take all appropriate measures to ensure the safety of the patients.

Screening of subjects should strictly follow the inclusion and exclusion criteria of the study. If an AE occurs during the study period, relevant evaluations will be performed, including blood routine examination, coagulation, creatinine, hepatic function, renal function, arterial blood gas analysis, ultrasound and computer tomography. Targeted treatment and necessary consultation should be carried out in a timely manner. When dealing with SAEs, it is important to make sure patient’s airway is clear, respiration, blood pressure and heart rate is steady.

Notably, the investigator should follow up the outcome of any AEs (clinical signs, laboratory values or other, etc.) until the patient's condition returns to normal or stabilizes. The follow-up will continue even if the patient withdraws from the clinical trial, and the patient will be interviewed by telephone or face-to-face at the scheduled visit time. The monitoring team may request additional visits and investigations.

10. Statistical Analysis

10.1 Sample size estimation

In this study, a multicenter, open, randomized, parallel control design method was used. The primary measure of efficacy was mRS score at 90 days (±7 days) after enrollment (considered as ordered variable). According to the literature data and clinical experts' opinions, the parameters were set as follows: (1) The proportion of mRS score 0-6 in control group was 3%, 4%, 10%, 17%, 16%, 12% and 38%, respectively; (2) The average treatment effect of EVT improved the outcome with the common OR value for improvement of mRS reached 1.73; (3) Two Interim analysis were considered. Adjusted level $\alpha=0.046$ (two-sided) and power $1-\beta=0.90$. (4) The randomization was allocated to the intervention group and the control group in a 1:1 ratio. Based on these parameters, the total sample size was 452. Considering 10% attrition rate,

the final total sample size was 502 cases, 251 cases in each group.

Interim analysis will be conducted when 1/3 (168) and 2/3 (336) of patients have completed 3-month follow-up. The O'Brien-Fleming boundaries will be used at the interim analysis with a two-sided alpha of 0.0002 (stage 1), 0.0123 (stage 2) and 0.046 (stage 3, final analysis).

The PASS software (NCSS, LLC, version 11) was used to calculate the sample size.

10.2 Data collection and entry

Paper-based CRF and electronic data capture (EDC) system will be used for data collection and input. All the content required by the protocol in the system must be filled, the unfilled content should be explained, and the reason needs to be marked in the EDC system.

10.2.1 Paper-based CRF filled out by the investigator

Site investigators should use black or blue-black recording pens to fill out the paper-based CRF neatly and clearly to ensure that the data is clear and readable. If the paper-based CRF information needs to be modified, it should not be altered or overwritten. The correct information should be written next to the original information, signed and dated by the person who modified it. The clinical research monitor (Clinical Research Associate, CRA) will review the completeness and accuracy of the CRF and guide the investigator to make necessary corrections and supplements.

10.2.2 Data entry to the EDC system by CRC

After the paper-based CRF is completed, the Clinical research coordinator (CRC) will input the content of the paper CRF into the EDC system.

10.2.3 Submission to the EDC system after the approval of the investigator

The paper-based CRF is will be submitted after the investigator has approves it. After the data is submitted, all data revisions and feedback are carried out through the EDC system. If the EDC system has submitted a form that needs to be modified, contact the CRA of this center.

After the CRA opens the form, the investigator can guide the CRC to modify the data in EDC system.

10.2.4 Data monitoring and query by CRA via EDC

10.2.5 Data exportation from the EDC system

After the data from the EDC system is exported to the database, it will be proofread by the data administrator. Obvious errors will be corrected by the data administrator. Other errors or missing values will be filled in the data query form, and the query form will be sent to the participating center for solutions through email, express, telephone or WeChat.

The participating centers are responsible for correcting the data in the EDC system after verifying the original data and related information. Site investigators must answer these queries by verifying or modifying relevant information or data.

10.3 Statistical considerations

This section is an overview of the statistical considerations. It provides the general specifications for the analysis of the data to be collected and presented in the CSR. A final Statistical Analysis Plan (SAP) will be issued prior to database lockdown and before code breaking. The SAP will define all “pre-specified, planned analyses.”

All programming will be performed using SAS Version 9.4.

10.3.1 Analysis sets

(1) Full Analysis Set (FAS):

Based on the principles of the Intent-to-Treat (ITT) analysis, all randomized subjects, either treated with medication or with EVT will be included in the FAS. The primary efficacy endpoint analysis of this study will be performed on the FAS.

(2) Per Protocol Set (PPS)

The PPS is a subset that includes all subjects who were treated with the treatment

assignment to which they were randomized and there are no clinically meaningful deviations from the protocol. Severe deviations from the protocol will be finally defined during the data auditing process, including but not limited to the followings:

1) The subject is not in line with the inclusion criteria.

2) There are other treatments that potentially confound the appraisal of efficacy of the planned treatment.

3) Poor compliance.

4) Follow-up interval exceeds the required time window.

Secondary analysis will be conducted on the PPS. If its result are not consistent with that of the FAS, a detailed analysis to examine the difference(s) will be required.

(3) Safety Analysis Set (SAS)

The SAS consists of all subjects who received treatment with at least one evaluation of the safety outcome.

10.3.2 Statistical considerations

(1) Baseline characteristics comparisons

T-test or Wilcoxon rank sum test will be used for comparison between continuous variables, and Chi-squared tests, Fisher's exact test or Wilcoxon sum rank test will be used for comparison between categorical variables.

(2) Efficacy Analysis

Primary efficacy endpoint: Based on an ITT basis, an ordinal logistic regression model is used to calculate the common odds ratio between the two treatment groups. All statistics will be two-sided with $p<0.046$ considered significant.

Secondary Efficacy Analyses: Endpoints including the 90-day mRS 0-2 will be analyzed using a binary logistic regression model. The infarct core volume change from baseline will be analyzed using student t-test or Wilcoxon rank sum test as appropriate.

(3) Safety Analysis

Safety events in the two treatment groups will be described based on the SAS dataset. Logistic regression will be used to compare the differences in safety endpoints such as intracranial bleeding events between the two groups. Chi-square test and Fisher's exact test will be used to compare the differences in the incidence of AEs and SAEs between the two groups.

(4) Subgroup analysis

The mRS at 90 days will be presented for each level of the covariates listed below:

(1) Age (< 70 vs. ≥70)

(2) Weak-up stroke or not

(3) Last known well to randomization time (< 6h vs. ≥ 6h)

(4) Stroke severity before randomization (NIHSS<16 vs. NIHSS≥16)

(5) IV thrombolysis or not

(6) Occlusion site (ICA vs. M1 segment)

(7) Ipsilateral carotid artery occlusion or not

(8) ASPECTS (< 3 points vs. ≥3 points)

(9) Infarct core volume (< 70ml vs. ≥70ml)

(10) Etiological stroke subtype (Cardiac embolism vs. large artery atherosclerosis)

10.4 Interim analysis

Interim analysis will take place when 1/3 (168 cases) and 2/3 (336 cases) have completed 3-month follow-up. The O'Brien-Fleming boundaries will be used at the interim analysis as follows:

There are no established techniques for the assessment of interim trial efficacy boundaries using an ordinal logistic regression model (proportional odds model). Instead, we will revert to a simple dichotomous analysis of the mRS score at 0-2 defined at 90 days from randomization. The Z-statistic for this analysis shall be derived from the normal approximation of the binomial distribution as an unadjusted two-sample test of proportions. For an RCT comparing two treatment groups with respect to a binary outcome and two interim analysis, corresponding

significance levels based on the O'Brien & Fleming boundary are two-sided 0.0002 (stage 1), 0.0123 (stage 2) and 0.046 (stage 3, final analysis).

With the result of interim analysis, DSMB will make the decision to continue or halt the study according to the test boundaries. The study will stop prematurely for futility if the result from the interim analysis indicate that we can't achieve an effective conclusion with the current sample size. Premature stopping for early success will be achieved if the interim analysis result has already proved the effect of intervention at significance level. Otherwise, the study will be continued until the predefined termination date. In interim analysis, the final sample size is allowed to be adjusted if the estimation of the primary outcome is drastically different from the actual data.

11. Ethical standards

11.1 Ethical standards

This Clinical Trial will be conducted in accordance with the principles laid down by the 18th World Medical Assembly (Helsinki, 1964) and all applicable amendments laid down by the World Medical Assemblies and the ICH guidelines for Good Clinical Practice. Prior to initiating the study, each site will obtain Institutional review board (IRB) or institutional ethics committee (IEC) approval for the protocol, informed consent forms and materials used to recruit subjects. Before each subject is enrolled, the investigator is responsible for fully and comprehensively introducing the purpose, procedures and possible risks of the study to the subject or his/her agent, signing a written informed consent form, and informing the subjects that he has the right to withdraw from this study at any time. The informed consent should be kept as a clinical study document for future reference. The personal privacy and data confidentiality of subjects will be protected during the study process.

11.2 Law and regulations

This Clinical Trial will be conducted in compliance with all international laws and regulations, and Chinese laws and regulations, as well as any applicable guidelines.

11.3 Informed consent

The Investigator/sub-investigator should fully inform the patient of all pertinent aspects of the Clinical Trial, including the written information approved/preferred by the Ethics Committee (IRB/IEC). The Informed Consent Form used by the Investigator for obtaining the patient's informed consent must be reviewed and approved by the Sponsor and then submitted to the Ethics Committee (IRB/IEC) for approval.

All participants should be informed to the fullest extent possible about the study, in language and terms they are able to understand. Prior to a patient's participation in the Clinical Trial, informed Consent Form should be signed and dated by the patient or by the patient's legal representative and by the person who conducted the informed consent discussion. A copy of the signed and dated Informed Consent Form will be provided to the patient.

11.4 Institutional review board/ Institutional ethics committee (IRB/IEC)

The Investigator or the Sponsor must submit this Clinical Trial Protocol to the appropriate Ethics Committee (IRB/IEC), and the Ethics Committee is required to forward to the Sponsor a copy of the written approval/favorable opinion signed and dated by the Chairman with Ethics Committee (IRB/IEC) composition.

The Clinical Trial (study number, Clinical Trial Protocol title and version number), the documents reviewed (Clinical Trial Protocol, Informed Consent Form, Investigator's Brochure, Investigator's CV, etc.), the list of voting members along with their qualification and the date of the review should be clearly stated on the written (IRB/IEC) approval/favorable opinion.

During the Clinical Trial, any amendment or modification to the Clinical Trial Protocol should be submitted to the Ethics Committee (IRB/IEC). It should also be informed of any event likely to affect the safety of patients or the continued conduct of the Clinical Trial, in particular any change in safety. All updates to the Investigator's Brochure will be sent to the Ethics Committee (IRB/IEC). If requested, annual progress report, as well as final summary of the Clinical Trial's outcome at the end of the Clinical Trial, will also be sent to the Ethics Committee (IRB/IEC).

12. Confidentiality and publication of research findings

The principal investigator has complete intellectual property rights. The entire research process and data analysis process will strictly protect the subjects' information. Publication of the results of this trial will be governed by the policies and procedures developed by the Executive Committee. The trial results will be published as soon as possible after database lockdown. This trial will produce detailed data on treatment effects, medical care, and outcomes. Biostatisticians will be consulted to ensure that it is impossible to uniquely identify any participant. Diskettes with the data in comma-delimited text format, along with a data dictionary in a text file, will be sent to interested parties.

13. Study Organization

13.1 Constitution

- **The steering committee**

  - ✓ The steering committee will provide scientific and strategic direction for the trial and will have overall responsibility for its design, execution, and publication.
  - ✓ The steering committee will also be responsible for ensuring that study execution and management are of the highest quality.
  - ✓ It will approve the protocol and the operational guidelines of the trial prior to its commencement.
  - ✓ The steering committee will meet regularly by teleconference or face-to-face meetings to discuss and report the progress of the study.
  - ✓ The composition of the steering committee and its responsibilities are described in a charter which will be finalized before the start of the trial.

Steering Committee

**Member**	**Department**	**Hospital**
Yongjun Wang	Department of Neurology	Beijing Tiantan Hospital, Capital Medical University

Yilong Wang	Department of Neurology	Beijing Tiantan Hospital, Capital Medical University
Liping Liu	Department of Neurology	Beijing Tiantan Hospital, Capital Medical University
David S. Liebeskind	Department of Neurology	University of California at Los Angeles
Zhongrong Miao	Department of Interventional Neuroradiology	Beijing Tiantan Hospital, Capital Medical University
Zeguang Ren	Department of Neurosurgery	The Affiliated Hospital of Guizhou Medical University
Vitor Mendes Pereira	Department of Neurosurgery, Division of Surgery	St Michael's Hospital, University of Toronto

- **Executive committee**

The executive committee is responsible for reviewing the status of the trial and available blinded data and will take appropriate actions regarding the conduct of the study. Executive Committee meetings will be organized to make major decisions. The composition of the Executive Committee and its responsibilities are described in a charter which will be finalized before the start of the trial.

- **Data safety and monitoring board (DSMB)**

The DSMB will meet regularly and monitor the study progress to ensure that the study meets the highest standards of ethics and patient safety. It is composed of Academic Members, including an independent statistician, who does not otherwise participate in the trial. A DSMB charter including membership, role and responsibilities will be approved by both the DSMB and the Executive Committee before the start of the trial.

Written recommendations and their rationale will be provided to the Chairs of the Steering Committee immediately after each DSMB meeting.

Data safety and monitoring board

**Member**	**Department**	**Hospital**
Jianmin Liu	Neurovascular Center	Changhai Hospital, Naval Medical University
Chen Yao	Department of Medical Statistics	Peking University First Hospital
Kangning Chen	Department of Neurology	The Southwest Hospital of Army Medical University

- **Clinical event committee (CEC)**

Clinical events and safety endpoint will be reviewed by CEC. A CEC charter including membership, role and responsibilities will be approved before the start of the trial by the CEC and the Executive Committee.

Clinical event committee

Member	Department	Hospital
Kun Fang	Department of Neurology	Huashan Hospital, Fudan University
Bo Song	Department of Neurology	The First Affiliated Hospital of Zhengzhou University
Yi Dong	Department of Neurology	Huashan Hospital, Fudan University

- **Imaging assessment committee**

Name	Department	Hospital
Jing Jing	Tiantan Neuroimaging Center of Excellence (T-NICE)	China National Clinical Research Center for Neurological Diseases
Zhe Zhang	Tiantan Neuroimaging Center of Excellence (T-NICE)	China National Clinical Research Center for Neurological Diseases
Yingkui Zhang	Tiantan Neuroimaging Center of Excellence (T-NICE)	China National Clinical Research Center for Neurological Diseases
Wei Wu	Department of Neurology	Qilu Hospital, Shandong University

13.2 Site training and certification

Executive committee will provide training to their participating sites in Good Clinical Practice Guidelines and in some outcome assessments. Prior to initiation of patient enrollment, Site Investigators and Coordinators must complete all training programs.

The training programs that need to be completed are as follows:

(1) Study procedures

(2) ANGEL-ASPECT eligibility criteria

(3) mRS

(4) NIHSS

(5) ASPECTS

(6) iSchemaView automated RAPID® software

(7) eTICI

(8) TOAST etiology subtyping

(9) Heidelberg Bleeding Classification

(10) Collecting DICOM imaging data

Successful completion of the training program is a must before a site begin to enroll patients. The conference call will be held intermittently, and PI and key staff will be available to answer questions.

A detailed Manual of Procedures will serve as the primary document describing all study related procedures. It will serve as a guide to train clinical center personnel and will be updated periodically throughout the study on the ANGEL-ASPECT website, as needed. A system composed of members of executive committee and CRA will be implemented for the clinical centers to ask any procedural questions by phone, fax, or e-mail. The ANGEL-ASPECT executive committee and monitoring committee will formulate answers in consultation with the Steering Committee and will periodically distribute to the participating centers a set of frequently asked questions and answers.

The members of executive committee will manage and conduct site visits to ensure the integrity and validity of the data on the CRF. During the trial period, each site should be visited at least once. If there are data quality problems or recruitment problems, it should be visited as needed.

14. Study monitoring and quality assurance control

14.1 Responsibilities of the investigator(s)

The Investigator(s) should conduct the Clinical Trial in accordance with the Clinical Trial Protocol, The International Council for Harmonisation of Technical Requirements for Pharmaceutical for Human Use (ICH) guidelines for Good Clinical Practice and the applicable regulatory requirements.

The Investigator is required to ensure compliance with all procedures required by the Clinical Trial Protocol and with all study procedures provided by the Sponsor (including security rules). The Investigator should provide reliable data and all information requested by the Protocol (with the help of the CRF, Discrepancy Resolution Form or other appropriate instruments) in an accurate and legible manner and ensure direct access to source documents by Sponsor representatives.

The Investigator may appoint other individuals as Sub-Investigators, as he thinks appropriate. All Sub-Investigators shall be appointed and listed in a timely manner and will be supervised by the Investigator. The Investigator will provide them a copy of the Clinical Trial Protocol and all necessary information. The Sponsor is responsible for taking all reasonable steps to ensure the proper conduct of the Clinical Trial Protocol as regards ethics, Clinical Trial Protocol compliance, and integrity and validity of the data on the CRF.

14.2 Study monitoring

The main responsibility of the monitoring team is to help researchers to ensure that all aspects of clinical trials are ethical, scientific, professional, and standardized. According to the ICH guidelines for Good Clinical Practice (GCP), the Monitoring Team must check the CRF entries according to the source documents, except for the pre-identified.

The monitoring team will regularly contact each center through site visits or online webinar, and will send inspectors to evaluate the research progress, adherence of the investigators and patients to the research protocol and to solve urgent problems. During these inspection visits, the inspector will work together with the site-investigators. The main aspects of inspection and monitoring are as follows (not exclusive): patient’s informed consent, patient recruitment and follow-up, documentation and reporting of SAEs and data quality.

15. Data retention

The double reviewed CRF and imaging data will be sent to the trial-designated data management center by CRAs. The person in charge of the data management center will check and sign the receipt form. The CRF will be kept by the research center after data entry is completed.

16. Data Security Monitoring

The data safety monitoring board (DSMB) is established to monitor the safety of participants, protect participants and ensure the integrity of the study. All AEs should be recorded, handled and tracked until they are properly resolved or stabilized. Any SAEs and unexpected events should be reported in a timely manner to the ethics committee in accordance with the relevant provisions, the competent department, the sponsor and the supervisory and administrative departments. The principal researchers should regularly review all AEs and set up meetings to assess the risks and benefits of the study if necessary. An independent data safety monitoring committee will be appointed to review safety data, evaluate the effectiveness of data monitoring, and decide whether to make new proposal.

During the clinical trial, the data of the subjects should be collected anonymously in the CRF. The subjects are identified only by the subject number and the abbreviation of the initials. Due to safety reason and administrative instructions, when the subject's identity is leaked, researchers shall share the responsibility of confidentiality. In the informed consent form, the patient allows authorized research staff, ethics committee, and the authority to refer directly to the relevant original data on the case report (such as the patient's medical file case, booking records, the original laboratory records, etc.). The above personnel shall comply with occupational confidentiality rules and must keep all patient's identity and medical information confidential.

17. Registration and Publication

17.1 Registration of study summary and results

The study representatives register a study summary in ClinicalTrials.gov (https: //clinicaltrials.gov) before the start of the study and update the summary as appropriate according to changes in the protocol or progress of the study. When the study is completed, the study representatives register a study result without delay.

17.2 Publication of study results

When the study is completed, the study database will be closed within one month after the last scheduled follow-up date of the last included patient. A manuscript which describes the study and the answer to the primary research question will be submitted to a major clinical journal within 3 months from closure of the database. The study representatives publish the results of the study after taking necessary measures (e.g., to prevent identification of specific study patients) to protect the human rights of patients and related parties or the rights and benefits of patients and related parties.

The manuscript will be shared with the financial sponsor(s) one month before submission, but the financial sponsor(s) will have no influence on its contents. Author(s) of the paper are determined by the study representatives according to the Uniform Requirements for Manuscripts Submitted to Biomedical Journals (http: //www.icmje.org/) by the International Committee of Medical Journal Editors (ICMJE). All authors should review and agree to the details of the paper prior to submission. The same goes for authors of conference presentations.

18. Ownership and use of data

18.1 Ownership of the data

The results, data, intellectual property rights, etc. obtained in this study belong to

the study representatives and not to the patients. Whether the intellectual property rights of the study representatives belong to the individual or to the study institution is determined by the agreement of the participating hospital.

18.2 Use of collected data

The study Steering Committee determines whether to use the data obtained in this study (hereinafter, “study data”) for further study conducted by the Study representatives or sub-investigators as a secondary analysis of this study.

If the analysis is judged to be beyond the scope of secondary analysis, or if the study data is used by a person except for the study representatives or sub-investigators, the Study Steering Committee prepares a separate protocol and conducts the study after undergoing ethical review in accordance with relevant laws, regulations and ethical guidelines for medical research on human subjects.

19. Funding and conflict of interest

The study was funded by untrestricted grants from Covidien Healthcare International Trading (Shanghai) Co., Ltd., Johnson & Johnson MedTech, Genesis MedTech（Shanghai）Co., Ltd. and Shanghai HeartCare Medical Technology Co., Ltd.

20. Reference

1. Powers WJ, Rabinstein AA, Ackerson T, et al. Guidelines for the Early Management of Patients With Acute Ischemic Stroke: 2019 Update to the 2018 Guidelines for the Early Management of Acute Ischemic Stroke: A Guideline for Healthcare Professionals From the American Heart Association/American Stroke Association. Stroke 2019;50(12):e344-e418. DOI: 10.1161/STR.0000000000000211.
2. Turc G, Bhogal P, Fischer U, et al. European Stroke Organisation (ESO)- European Society for Minimally Invasive Neurological Therapy (ESMINT) guidelines on mechanical thrombectomy in acute ischemic stroke. J Neurointerv Surg 2019;11(6):535-538. DOI: 10.1136/neurintsurg-2018-014568.
3. Berkhemer OA, Fransen PS, Beumer D, et al. A randomized trial of intraarterial treatment for acute ischemic stroke. N Engl J Med 2015;372(1):11-20. DOI: 10.1056/NEJMoa1411587.

4. Campbell BC, Mitchell PJ, Kleinig TJ, et al. Endovascular therapy for ischemic stroke with perfusion-imaging selection. N Engl J Med 2015;372(11):1009-18. DOI: 10.1056/NEJMoa1414792.
5. Goyal M, Demchuk AM, Menon BK, et al. Randomized assessment of rapid endovascular treatment of ischemic stroke. N Engl J Med 2015;372(11):1019-30. DOI: 10.1056/NEJMoa1414905.
6. Jovin TG, Chamorro A, Cobo E, et al. Thrombectomy within 8 hours after symptom onset in ischemic stroke. N Engl J Med 2015;372(24):2296-306. DOI: 10.1056/NEJMoa1503780.
7. Saver JL, Goyal M, Bonafe A, et al. Stent-retriever thrombectomy after intravenous t-PA vs. t-PA alone in stroke. N Engl J Med 2015;372(24):2285-95. DOI: 10.1056/NEJMoa1415061.
8. Nogueira RG, Jadhav AP, Haussen DC, et al. Thrombectomy 6 to 24 Hours after Stroke with a Mismatch between Deficit and Infarct. N Engl J Med 2018;378(1):11-21. DOI: 10.1056/NEJMoa1706442.
9. Albers GW, Marks MP, Kemp S, et al. Thrombectomy for Stroke at 6 to 16 Hours with Selection by Perfusion Imaging. N Engl J Med 2018;378(8):708-718. DOI: 10.1056/NEJMoa1713973.
10. Bracard S, Ducrocq X, Mas JL, et al. Mechanical thrombectomy after intravenous alteplase versus alteplase alone after stroke (THRACE): a randomised controlled trial. Lancet Neurol 2016;15(11):1138-47. DOI: 10.1016/S1474-4422(16)30177-6.
11. Gautheron V, Xie Y, Tisserand M, et al. Outcome After Reperfusion Therapies in Patients With Large Baseline Diffusion-Weighted Imaging Stroke Lesions: A THRACE Trial (Mechanical Thrombectomy After Intravenous Alteplase Versus Alteplase Alone After Stroke) Subgroup Analysis. Stroke 2018;49(3):750-753. DOI: 10.1161/STROKEAHA.117.020244.
12. Deb-Chatterji M, Pinnschmidt H, Flottmann F, et al. Predictors of independent outcome of thrombectomy in stroke patients with large baseline infarcts in clinical practice: a multicenter analysis. J Neurointerv Surg 2020;12(11):1064-1068. DOI: 10.1136/neurintsurg-2019-015641.
13. Roman LS, Menon BK, Blasco J, et al. Imaging features and safety and efficacy of endovascular stroke treatment: a meta-analysis of individual patient-level data. Lancet Neurol 2018;17(10):895-904. DOI: 10.1016/S1474-4422(18)30242-4.
14. Campbell BCV, Majoie C, Albers GW, et al. Penumbral imaging and functional outcome in patients with anterior circulation ischaemic stroke treated with endovascular thrombectomy versus medical therapy: a meta-analysis of individual patient-level data. Lancet Neurol 2019;18(1):46-55. DOI: 10.1016/S1474-4422(18)30314-4.
15. Sarraj A, Hassan AE, Savitz S, et al. Outcomes of Endovascular Thrombectomy vs Medical Management Alone in Patients With Large Ischemic Cores: A Secondary Analysis of the Optimizing Patient's Selection for Endovascular Treatment in Acute Ischemic Stroke (SELECT) Study. JAMA Neurol 2019;76(10):1147-1156. DOI: 10.1001/jamaneurol.2019.2109.
16. Cagnazzo F, Derraz I, Dargazanli C, et al. Mechanical thrombectomy in patients with acute ischemic stroke and ASPECTS </=6: a meta-analysis. J Neurointerv Surg 2020;12(4):350-355. DOI: 10.1136/neurintsurg-2019-015237.
17. Sarraj A, Grotta JC, Pujara DK, Shaker F, Tsivgoulis G. Triage imaging and outcome measures for large core stroke thrombectomy - a systematic review and meta-analysis. J Neurointerv Surg 2020;12(12):1172-1179. DOI: 10.1136/neurintsurg-2019-015509.
18. Rebello LC, Bouslama M, Haussen DC, et al. Endovascular Treatment for Patients With Acute Stroke Who Have a Large Ischemic Core and Large Mismatch Imaging Profile. JAMA Neurol

2017;74(1):34-40. DOI: 10.1001/jamaneurol.2016.3954.
19. Broocks G, Flottmann F, Schonfeld M, et al. Incomplete or failed thrombectomy in acute stroke patients with Alberta Stroke Program Early Computed Tomography Score 0-5 - how harmful is trying? European journal of neurology 2020;27(10):2031-2035. DOI: 10.1111/ene.14358.
20. Nicholson P, Hilditch CA, Neuhaus A, et al. Per-region interobserver agreement of Alberta Stroke Program Early CT Scores (ASPECTS). J Neurointerv Surg 2020;12(11):1069-1071. DOI: 10.1136/neurintsurg-2019-015473.
21. van Horn N, Kniep H, Broocks G, et al. ASPECTS Interobserver Agreement of 100 Investigators from the TENSION Study. Clin Neuroradiol 2021. DOI: 10.1007/s00062-020-00988-x.
22. Demeestere J, Garcia-Esperon C, Garcia-Bermejo P, et al. Evaluation of hyperacute infarct volume using ASPECTS and brain CT perfusion core volume. Neurology 2017;88(24):2248-2253. DOI: 10.1212/WNL.0000000000004028.
23. Sarraj A, Hassan AE, Grotta J, et al. Optimizing Patient Selection for Endovascular Treatment in Acute Ischemic Stroke (SELECT): A Prospective, Multicenter Cohort Study of Imaging Selection. Ann Neurol 2020;87(3):419-433. DOI: 10.1002/ana.25669.
24. Mourand I, Abergel E, Mantilla D, et al. Favorable revascularization therapy in patients with ASPECTS </= 5 on DWI in anterior circulation stroke. J Neurointerv Surg 2018;10(1):5-9. DOI: 10.1136/neurintsurg-2017-013358.
25. Inoue M, Olivot JM, Labreuche J, et al. Impact of diffusion-weighted imaging Alberta stroke program early computed tomography score on the success of endovascular reperfusion therapy. Stroke 2014;45(7):1992-8. DOI: 10.1161/STROKEAHA.114.005084.
26. Han M, Choi JW, Rim NJ, et al. Cerebral infarct volume measurements to improve patient selection for endovascular treatment. Medicine (Baltimore) 2016;95(35):e4702. DOI: 10.1097/MD.0000000000004702.
27. Manceau PF, Soize S, Gawlitza M, et al. Is there a benefit of mechanical thrombectomy in patients with large stroke (DWI-ASPECTS </= 5)? European journal of neurology 2018;25(1):105-110. DOI: 10.1111/ene.13460.
28. Broocks G, Rajput F, Hanning U, et al. Highest Lesion Growth Rates in Patients With Hyperacute Stroke. Stroke 2018:STROKEAHA118023457. DOI: 10.1161/STROKEAHA.118.023457.
29. Goyal M, Fargen KM, Turk AS, et al. 2C or not 2C: defining an improved revascularization grading scale and the need for standardization of angiography outcomes in stroke trials. J Neurointerv Surg 2014;6(2):83-6. DOI: 10.1136/neurintsurg-2013-010665.
30. von Kummer R, Broderick JP, Campbell BC, et al. The Heidelberg Bleeding Classification: Classification of Bleeding Events After Ischemic Stroke and Reperfusion Therapy. Stroke; a journal of cerebral circulation 2015;46(10):2981-6. DOI: 10.1161/STROKEAHA.115.010049.
31. Zaidat OO, Yoo AJ, Khatri P, et al. Recommendations on angiographic revascularization grading standards for acute ischemic stroke: a consensus statement. Stroke; a journal of cerebral circulation 2013;44(9):2650-63. DOI: 10.1161/STROKEAHA.113.001972.
32. Boers AM, Marquering HA, Jochem JJ, et al. Automated cerebral infarct volume measurement in follow-up noncontrast CT scans of patients with acute ischemic stroke. AJNR Am J Neuroradiol 2013;34(8):1522-7. DOI: 10.3174/ajnr.A3463.
33. Liu L, Chen W, Zhou H, et al. Chinese Stroke Association guidelines for clinical management

of cerebrovascular disorders: executive summary and 2019 update of clinical management of ischaemic cerebrovascular diseases. Stroke Vasc Neurol 2020;5(2):159-176. DOI: 10.1136/svn-2020-000378.

34. EuroQol G. EuroQol--a new facility for the measurement of health-related quality of life. Health Policy 1990;16(3):199-208. DOI: 10.1016/0168-8510(90)90421-9.

21. Appendix

Appendix Table 1. Modified Rankin Scale

The modified Rankin Scale (mRS) is an ordinal hierarchical scale ranging from 0 to 5, with higher scores indicating more severe disability. A score of 6 has been added to signify death.

**Category**	**Short description**	**Long description**
0	No symptoms	No symptoms
1	Symptoms, no disability	Minor symptoms that do not interfere with lifestyle
2	Slight disability	Slight disability, symptoms that lead to some restriction in lifestyle, but do not interfere with the patient's capacity to look after himself.
3	Moderate disability	Moderate disability, symptoms that significantly restrict lifestyle and prevent totally independent existence
4	Moderately severe disability	Moderately severe disability, symptoms that clearly prevent independent existence though not needing constant attention
5	Severe disability	Severe disability, totally dependent patient requiring constant attention day and night.
6	Death	Death

Appendix Table 2. Extended Treatment In Cerebral Ischemia (eTICI) Scale

eTICI grade	Short description	Long description
**0**	No perfusion	No antegrade flow beyond the point of occlusion
**1**	Limited reperfusion	Antegrade reperfusion past the initial occlusion, but limited distal branch filling with little or slow distal reperfusion
**2a**	<50% reperfusion	Antegrade reperfusion of less than half of the occluded target artery previously ischemic territory (eg, in 1 major division of the MCA and its territory)
**2b**	≥50% and <90% reperfusion	Antegrade reperfusion of more than half of the previously occluded target artery ischemic territory (eg, in 2 major divisions of the MCA and its territories)
**2c**	≥90% reperfusion	Near complete antegrade reperfusion of the previously occluded target artery ischemic territory, except for slow flow or distal emboli in a few distal cortical vessels
**3**	100% reperfusion	Complete antegrade reperfusion of the previously occluded target artery ischemic territory, with absence of visualized occlusion in all distal branches

MCA: middle cerebral artery; eTICI; extended treatment in cerebral ischemia scale

Appendix Table 3. National Institute of Health Stroke Scale (NIHSS)

The NIHSS is an ordinal hierarchical scale to evaluate the severity of stroke by assessing a patient's performance. Scores range from 0 to 42, with higher scores indicating a more severe deficit. Administer stroke scale items in the order listed. Record performance in each category after each subscale exam. Do not go back and change scores. Follow directions provided for each exam technique. Scores should reflect what the patient does, not what the clinician thinks the patient can do. The clinician should record answers while administering the exam and work quickly. Except where indicated, the patient should not be coached (i.e. repeated requests to patient to make a special effort).

Instructions	Scale definition
**1a. Level of consciousness.** The investigator must choose a response if a full evaluation is prevented by such obstacles as an endotracheal tube, language barrier, orotracheal trauma/bandages. A 3 is scored only if the patient makes no movement (other than reflexive posturing) in response to noxious stimulation.	0 = Alert; keenly responsive.<br>1 = Not alert; but arousable by minor stimulation to obey, answer, or respond.<br>2 = Not alert; required repeated stimulation to attend, or is obtunded and requires strong or painful stimulation to make movements (not stereotyped).<br>3 = Responds only with reflex motor or autonomic effects or totally unresponsive, flaccid and areflexic.
**1b. LOC Questions:** The patient is asked the month and his/her age. The answer must be correct – there is not partial credit for being close. Phasic and stuporous patients who do not comprehend the questions will score 2. Patients unable to speak because of endotracheal intubation, orotracheal trauma, severe dysarthria from any cause, language barrier, or any other problem not secondary to aphasia are given a 1. It is important that only the initial answer be graded and that the examiners not "help" the patient with verbal or non-verbal clues.	0 = Answers both questions correctly.<br>1 = Answers one question correctly.<br>2 = Answers neither question correctly.
**1c. LOC Commands:** The patient is asked to open and close the eyes and then to grip and release the non-paretic hand. Substitute another one step command if the hand cannot be used. Credit is given if an unequivocal attempt is made but not completed due to weakness. If the patient does not respond to command, the task should be demonstrated to him or her (pantomime), and the result scored (i.e. follows none, one or two commands). Patients with trauma, amputation, or other physical impediments should be given suitable one-step	0 = Performs both tasks correctly.<br>1 = Performs one task correctly.<br>2 = Performs neither task correctly.

commands. Only the first attempt is scored.	
**2. Best Gaze:** Only horizontal eye movements will be tested. Voluntary or reflexive (oculocephalic) eye movements will be scored, but caloric testing is not done. If the patient has a conjugate deviation of the eyes that can be overcome by voluntary or reflexive activity, the score will be a 1. If a patient has an isolated peripheral nerve paresis (CN III, IV or VI), score a 1. Gaze is testable in all aphasic patients. Patients with ocular trauma, bandages, preexisting blindness, or other disorder of visual acuity or fields should be tested with reflexive movements, and a choice made by the investigator. Establishing eye contact and then moving about the patient from side to side will occasionally clarify the presence of a partial gaze palsy.	0= Normal.<br>1= Partial gaze palsy; gaze is abnormal in one or both eyes, but forced deviation or total gaze paresis is not present.<br>2= Forced deviation; or total gaze paresis not overcome by the oculocephalic maneuver.
**3. Visual:** Visual fields (upper and lower quadrants) are tested by confrontation, using finger counting or visual threat, as appropriate. Patients may be encouraged, but if they look at the side of the moving finger appropriately, this can be scored as normal. If there is unilateral blindness or enucleation, visual fields in the remaining eye are scored. Score 1 only if a clear-cut asymmetry, including quadrantanopia, is found. If patient is blind from any cause, score 3.<br>Double simultaneous stimulation is performed in this case.<br>If there is extinction, the patient receives a 1, and the results are used to respond to item 11.	0= No visual loss.<br>1= Partial hemianopia.<br>2= Complete hemianopia.<br>3= Bilateral hemianopia (blind including cortical blindness)
**4. Facial palsy:** Ask or use pantomime to encourage the patient to show teeth or raise eyebrows and close eyes. Score symmetry of grimace in response to noxious stimuli in the poorly response or non-comprehending patient. If facial trauma/bandages, orotracheal tube, tape or other physical barriers obscure the face, these should be removed to the extent possible.	0 = Normal symmetrical movements.<br>1= Minor paralysis (flattened nasolabial fold, asymmetry on smiling)<br>2= Partial paralysis (total or near-total paralysis of lower face)<br>3= Complete paralysis of one or both sides (absence of facial movement in the upper and lower face).
**5. Motor arm:** The limb is placed in the appropriate position: extend the arms (palms down) 90 degrees (if sitting) or 45 degrees (if supine). Drift is scored if the arm falls before 10 seconds. The aphasic patient is encouraged using urgency in the voice and pantomime, but not noxious stimulation. Each limb is tested in turn, beginning with the non-paretic arm. Only in the case of amputation or joint fusion at the shoulder, the examiner should record the score as untestable (UN), and clearly write the explanation for this choice.	0= No drift; limb holds 90 (or 45) degrees for full 10 seconds.<br>1= Drift; limb holds 90 (or 45) degrees, but drifts down before full 10 seconds; does not hit bed or other support.<br>2= Some effort against gravity; limb cannot get to or maintain (if cued) 90 (or 45) degrees, drifts down to bed, but has some effort against gravity.<br>3= No effort against gravity; limb falls.<br>4= No movement.<br>UN = Amputation or joint fusion: explain:

	5a = Left Arm.<br>5b = Right arm.
**6. Motor leg:** The limb is placed in the appropriate position: hold the leg at 30 degrees (always tested supine). Drift is scored if the leg falls before 5 seconds. The aphasic patient is encouraged using urgency in the voice and pantomime, but not noxious stimulation. Each limb is tested in turn, beginning with the non-paretic leg. Only in the case of amputation or joint fusion at the hip, the examiner should record the score as untestable (UN), and clearly write the explanation for this choice.	0= No drift; leg holds 30-degree position for full 5 seconds.<br>1= Drift; leg falls by the end of the 5-second period but does not hit bed.<br>2= Some effort against gravity; leg falls to bed by 5 seconds, but has some effort against gravity.<br>3= No effort against gravity; leg falls to bed immediately.<br>4= No movement.<br>UN = Amputation or joint fusion: explain:<br>6a. Left Leg<br>6b. Right Leg.
**7. Limb ataxia:** This item is aimed at finding evidence of a unilateral cerebellar lesion. Test with eyes open. In case of visual defect, ensure testing is done in intact visual field. The finger-nose-finger and heel-shin tests are performed on both sides, and ataxia is scored only if present out of proportion to weakness. Ataxia is absent in the patient who cannot understand or is paralyzed. Only in the case of amputation or joint fusion, the examiner should record the score as untestable (UN), and clearly write the explanation for this choice. In case of blindness, test by having the patient touch nose from extended arm position.	0= Absent.<br>1= Present in one limb.<br>2= Present in two limbs.<br>UN = Amputation or joint fusion: explain:
**8. Sensory:** Sensation or grimace to pinprick when tested, or withdrawal from noxious stimulus in the obtunded or aphasic patient. Only sensory loss attributed to stroke is scored as abnormal and the examiner should test as many body areas (arms [not hands], legs, trunk, face) as needed to accurately check for hemisensory loss. A score of 2, 'severe or total sensory loss', should only be given when a severe or total loss of sensation can be clearly demonstrated. Stuporous and aphasic patients will, therefore, probably score 1 or 0. The patient with brainstem stroke who has bilateral loss of sensation is scored 2. If the patient does not respond and is quadriplegic, score 2.<br>Patients in a coma (item 1a=3) are automatically given a 2 on this item.	0= Normal; no sensory loss.<br>1= Mild-to-moderate sensory loss; patients feels pinprick is less sharp or is dull on the affected side; or there is a loss of superficial pain with pinprick, but patient is aware of being touched.<br>2= Severe to total sensory loss; patient is not aware of being touched in the face, arm and leg.
**9. Best language:** A great deal of information about comprehension will be obtained during the preceding sections of the examination. For this scale item, the patient is asked to describe what is happening in the attached picture, to name the items on the attached naming sheet and to read from the attached	0= No aphasia; normal<br>1= Mild-to-moderate aphasia; some obvious loss of fluency or facility of comprehension, without significant limitation on ideas expressed or form of expression. Reduction of

list of sentences. Comprehension is judged from responses here, as well as to all of the commands in the preceding general neurological exam. If visual loss interferes with the tests, ask the patient to identify objects placed in the hand, repeat, and produce speech. The intubated patient should be asked to write. The patient in a coma (item 1a=3) will automatically score 3 on this item. The examiner must choose a score for the patient with stupor or limited cooperation, but a score of 3 should be used only if the patient is mute and follows no one-step commands.	speech and/or comprehension, however, makes<br>conservation about provided materials difficult or impossible. For example, in conversation about provided materials, examiner can identify picture or naming card content from patient's response.<br>2= Severe aphasia; all communication is through fragmentary expression; great need for inference, questioning, and guessing by the listener. Range of information that can be exchanged is limited; listener carries burden of communication. Examiner cannot identify materials provided from patient response.<br>3 = Mute, global aphasia: no usable speech or auditory comprehension.
**10. Dysarthria:** If patient is thought to be normal, an adequate sample of speech must be obtained by asking patient to read or repeat words from the attached list. If the patient has severe aphasia, the clarity of articulation of spontaneous speech can be rated. Only if patient is intubated or has other physical barriers to producing speech, the examiner should record the score as untestable (UN), and clearly write an explanation for this choice. Do not tell the patient why he or she is being tested.	0= Normal.<br>1= Mild-to-moderate dysarthria; patient slurs at least some words and, at worst, can be understood by some difficulty.<br>2= Severe dysarthria: patient's speech is so slurred as to be unintelligible in the absence of or out of proportion to any dysphasia, or is mute/anarthric.<br>UN = Intubated or other physical barrier.
**11. Extinction and Inattention** (formerly Neglect): Sufficient information to identify neglect may be obtained during the prior testing. If the patient has a severe visual loss preventing visual double simultaneous stimulation, and the cutaneous stimuli are normal, the score is normal. If the patient has aphasia but does appear to attend to both sides, the score is normal. The presence of visual spatial neglect or anosagnosia may also be taken as evidence of abnormality. Since the abnormality is scored only if present, the item is never untestable.	0= No abnormality.<br>1= Visual, tactile, auditory, spatial, or personal inattention or extinction to bilateral simultaneous stimulation in one of the sensory modalities.<br>2= Profound hemi-inattention or extinction to more than one modality; does not recognize own hand or orients to only one side of space.

Appendix Table 4. EuroQoL 5D-5L

Under each heading, please tick the ONE box that best describes your health TODAY.

Mobility

I have no problems in walking about □
I have slight problems in walking about □
I have moderate problems in walking about □
I have severe problems in walking about □
I am unable to walk about □

Self-care

I have no problems washing or dressing myself □
I have slight problems washing or dressing myself □
I have moderate problems washing or dressing myself □
I have severe problems washing or dressing myself □
I am unable to wash or dress myself □

Usual activities (e.g. work, study, housework, family or leisure activities)

I have no problems doing my usual activities □
I have slight problems doing my usual activities □
I have moderate problems doing my usual activities □
I have severe problems doing my usual activities □
I am unable to do my usual activities □

Pain/discomfort

I have no pain or discomfort □
I have slight pain or discomfort □
I have moderate pain or discomfort □
I have severe pain or discomfort □
I have extreme pain or discomfort □

Anxiety/depression

I am not anxious or depressed □
I am slightly anxious or depressed □
I am moderately anxious or depressed □
I am severely anxious or depressed □
I am extremely anxious or depressed □

- We would like to know how good or bad your health is TOADY.
- This scale is numbered from 0 to 100.
- 100 means the best health you can imagine.
  0 means the worst health you can imagine.
- Mark an X on the scale to indicate how your health is TODAY.
- Now, please write the number you marked on the scale in the box below.

YOUR HEALEH TOADY = ☐

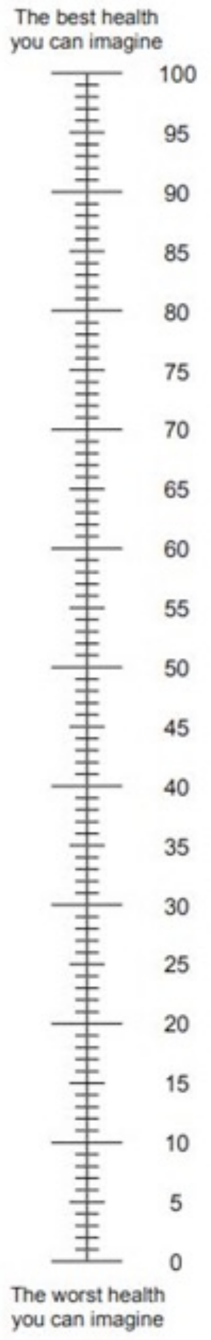

Appendix Table 5. Heidelberg bleeding classification

Symptomatic intracranial hemorrhage (SICH): new intracranial hemorrhage detected by brain imaging associated with any of the items below:

1. ≥4 points decline in the total NIHSS at the time of diagnosis compared to immediately before worsening. Note that a 4 points change is not compared with the baseline admission NIHSS score but instead to the immediate predeterioration neurological status

2. ≥2 point decline in one NIHSS category. The rationale for this is to capture new hemorrhages that produce new neurological symptoms, making them clearly symptomatic but not causing worsening in the original stroke territory. For example, a new remote hemorrhage in the contralateral occipital lobe may cause new hemianopia that is clearly symptomatic but the patient will not have worsening of ≥4 points on the NIHSS score

Leading to intubation/hemicraniectomy/EVD placement or other major medical/surgical intervention.

3. Absence of alternative explanation for deterioration

Anatomic Description of Intracranial Hemorrhages

Class	Type	Description
1	Hemorrhagic transformation of infarcted brain tissue	
1a	HI1	Scattered small petechiae, no mass effect
1b	HI2	Confluent petechiae, no mass effect
1c	PH1	Hematoma within infarcted tissue, occupying <30%, no substantive mass effect
2	Intracerebral hemorrhage within and beyond infarcted brain tissue	
	PH2	Hematoma occupying 30% or more of the infarcted tissue, with obvious mass effect
3	Intracerebral hemorrhage outside the infarcted brain tissue or intracranial-extracerebral hemorrhage	
3a		Parenchymal hematoma remote from infarcted brain tissue
3b		Intraventricular hemorrhage
3c		Subarachnoid hemorrhage
3d		Subdural hemorrhage

HI indicates hemorrhagic infarction; and PH, parenchymatous hematoma.

Appendix Table 6. Alberta Stroke Program Early CT Score (ASPECTS)

The Alberta Stroke Program Early CT Score (ASPECTS) is a semiquantitative method of estimation of infarct size with non-contrast CT during the acute phase. The territory of the middle cerebral artery is allotted 10 points. 1 point is subtracted for an area of early ischaemic change, such as focal swelling, or parenchymal hypoattenuation, for each of the defined regions. A normal CT scan has an ASPECTS value of 10 points. A score of 0 indicates diffuse ischaemia throughout the territory of the middle cerebral artery.

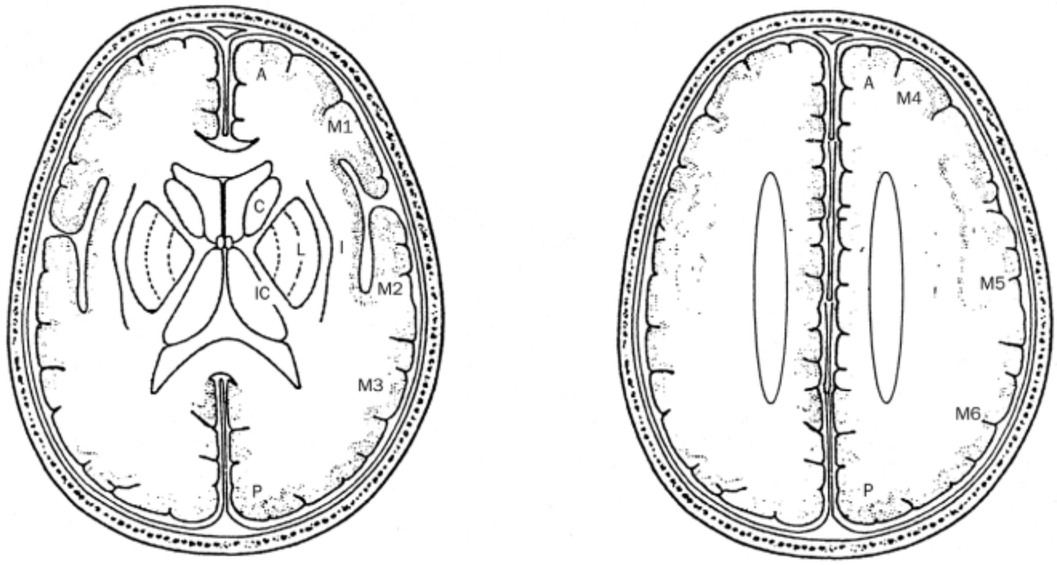

A=anterior circulation; P=posterior circulation; C=caudate; L=lentiform; IC=internal capsule; I=insular ribbon; MCA=middle cerebral artery; M1=anterior MCA cortex; M2=MCA cortex lateral to insular ribbon; M3=posterior MCA cortex; M4, M5, and M6 are anterior, lateral, and posterior MCA territories immediately superior to M1, M2, and M3, rostral to basal ganglia. Subcortical structures are allotted 3 points (C, L, and IC). MCA cortex is allotted 7 points (insular cortex, M1, M2, M3, M4, M5, and M6).

Summary of changes

ANGEL-ASPECT Protocol V6.0-20200723 (original version)

ANGEL-ASPECT Protocol V7.0-20210412 (amended version)

ANGEL-ASPECT Protocol V7.1-20210518 (final version)

Version	Date of revised	Changed	Reasons for change
V7.0	2021/04/12	**Added Trial Registration number (cover page)**: ClinicalTrials.gov NCT04551664	Finished Trial Registration in Clinical trials.gov after the study was approved by the Ethics Committee of the hospital.
		**Delete Neuroimaging Inclusion Criteria (4.1.3):**<br><br>"(3) Mismatch ratio on CT perfusion or MRI (Tmax>6s volume / Ischemic core volume) >1.2"	There is no need to use mismatch ratio select patients.
		**Add subgroup analyses (8.2.4):**<br><br>"(2) Weak-up stroke or not" and "(7) Ipsilateral carotid artery occlusion or not"	To indicate more specifically the subgroup for studies by add important subgroup in the research protocol prior to data fixation.
V7.1	2021/05/18	**Extension of the Study duration (cover page)：** "August 2020 to October 2022 (enrolment completed at October 2021)" → "August 2020 to October 2023 (enrolment completed at October 2022)"	Prolonged duration because of delayed enrollment.

		**Added committee tables of Study Organization (13.1):**<br>"Steering Committee, Data safety and monitoring board, Clinical event committee, Imaging assessment committee	Detailed tables of committee member.
		**The parameters were refined, the interim analysis was adjusted (10.1):**<br>"(2) The average treatment effect of EVT improved the outcome with the common OR value for improvement of mRS reached 1.74; (3) Two Interim analysis were considered. Adjusted level α=0.050 and power 1-β=0.90."<br>→ "(2) The average treatment effect of EVT improved the outcome with the common OR value for improvement of mRS reached 1.73; (3) Two Interim analysis were considered. Adjusted level α=0.046 (two-sided) and power 1-β=0.90."<br><br>**The sample size was recalculated (10.1):**<br>"(4) The sample size was allocated to the intervention group and the control group in a 1:1 ratio. Based on these parameters, the sample size was 219 in each group. Considering 10% attrition rate, the final total sample size was 488 cases, 244 cases in each group."<br>→ "(4) The sample size was allocated to the intervention group and the control group in a 1:1 ratio. Based on these parameters, the total sample size was 452. Considering 10% attrition rate, the final total sample size was 502 cases, 251 cases in each group."	The parameters were refined, the interim analysis was adjusted, and the sample size was recalculated.

		**Replaced Figure 1 (3.1):** Study design: randomization algorithm	The figure of the study design was replaced because the sample size was recalculated
		**Refined parameters (10.3.2):**<br>"All statistics will be with p<0.050 considered significant."<br>→ "All statistics will be two-sided with p<0.046 considered significant."	The parameters were refined.
		**Revised interim analyses plan (10.4):**<br>"Interim analysis will take place when 1/2 (244 cases) and 3/4 (366 cases) have completed 3-month follow-up." and "corresponding significance levels based on O'Brien & Fleming boundary are 0.003 (stage 1), 0.018 (stage 2) and 0.044 (stage 3, final analysis)."<br>→ "Interim analysis will take place when 1/3 (168 cases) and 2/3 (336 cases) have completed 3-month follow-up." and "corresponding significance levels based on O'Brien & Fleming boundary are two-sided 0.0002 (stage 1), 0.0123 (stage 2) and 0.046 (stage 3, final analysis)."	Revise the interim analyses plan according to DSMB's suggestion.

Study of Endovascular Therapy in Acute Anterior Circulation Large VeSsel Occlusive Patients with a LargE InfarCT Core: A Multicenter, Prospective, Open-Label, Blinded-Endpoint, Randomized Controlled Trial (ANGEL-ASPECT)

Statistical Analysis Plan

Principal Investigator

Zhongrong Miao, MD

Beijing Tiantan Hospital, Capital Medical University, Beijing, China

Prepared by

Yuesong Pan, PhD

Aoming Jin, PhD

Xianglong Xiang, MD

Beijing Tiantan Hospital, Capital Medical University, Beijing, China

Version 1.0

July 23, 2020

Table of Contents

1. Introduction..134
2. Study Objective..134
3. Study Endpoint(s)..134
Primary Efficacy Endpoints:..134
Secondary Efficacy Endpoint:..134
Primary Safety Endpoint..135
Secondary Safety Endpoint..135
4. Statistical Hypotheses..135
5. Design..135
6. Sample size estimates..137
7. Analysis populations..138
Full Analysis Set (FAS)..138
Per Protocol Set (PPS)..139
Safety Analysis Set (SAS)..139
8. Treatment comparisons..139
9. General considerations for data analyses..139
Examination of Subgroups..139
Multiple Comparisons and Multiplicity..139
10. Data handling conventions..140
Premature Withdrawal and Missing Data..140
Event Rates..140
11. Study Population..141
Disposition of Subjects..141
Protocol Deviations..141
Demographic and Baseline Characteristics..142
12. Efficacy Analyses..142
Primary Efficacy Analysis..142
Secondary Efficacy Analyses..143
13. Safety Analyses..144
14. References..145

1. Introduction

This statistical analysis plan (SAP) documents the planned statistical analyses for the ANGEL-ASPECT trial and is based on the protocol, together with any subsequent amendments.

This SAP is intended for the use of project team members and should be read in conjunction with the aforementioned protocol.

2. Study Objective

The primary objective of the study is to establish the efficacy of endovascular therapy (EVT) in Acute Anterior Circulation Large Vessel Occlusive (LVO) Patients with a large infarct core.

3. Study Endpoint(s)

Primary Efficacy Endpoints:

The 90 (±7) day modified Ranking scale.

Secondary Efficacy Endpoint:

1) 90 (±7) day mRS 0-2.

2) 90 (±7) day mRS 0-3.

3) 36h（24-48h）NIHSS 0-1 or decrease ≥10 from baseline.

4) 36h（24-48h）infarct volume change (by CT or MRI).

5) 36h（24-48h）target artery recanalization rate (by CTA or MRA).

Primary Safety Endpoint

•Rate of symptomatic intracranial hemorrhage within 48 hours from randomization (Heidelberg Bleeding Classification).

Secondary Safety Endpoint

1) All-cause mortality within 90 days.

2) Any intracranial hemorrhage within 48 hours from randomization (Heidelberg Bleeding Classification).

3) Decompressive hemicraniectomy during hospitalization.

4. Statistical Hypotheses

The primary outcome will be a shift of one or more categories (proportional odds analysis) on the modified Rankin scale determined at 90 days from randomization.
The primary hypothesis is:
H_A: Subjects in the group of best medical management plus EVT will have greater odds of showing improvement on the mRS at 90 days.

5. Design

This is a prospective, randomized, controlled, open label, blinded-endpoint (PROBE) study to compare the efficacy and safety of best medical management plus EVT compared to best medical management alone in Acute Anterior Circulation Large Vessel Occlusive (LVO) Patients with a large infarct core up to 24 hours from symptom onset or last seen well.

Patients who meet the inclusion criteria will be randomized to one of the following two treatment arms: best medical management plus EVT or best medical management alone. Endpoints in this prospective open label study will be assessed blinded to the

treatment assignment of the patient (PROBE design). This study will be conducted in approximately 50 sites in China.

Best medical management plus EVT group: Patients randomized to experimental group will receive EVT plus to best medical management. According to the pathological characteristics of patients and the judgment of researchers, the following treatment methods can be selected: Mechanical thrombectomy, angioplasty and arterial thrombolysis. Stent thrombectomy (Solitaire*, EMBOTRAP#, Trevo or Reco or other first-line stent thrombectomy systems) or aspiration (Penumbra system) is recommended as the first choice. If the recanalization is not successful, it can be replaced with balloon angioplasty or intracranial stent deployment. The need for rescue treatment is defined (including, but not limited to, a decision made by the investigator based on intraoperative conditions) as follows: three times of thrombectomy with the same thrombectomy device (stent or aspiration catheter) without successful recanalization; Target vessel was successfully recanalized and then reoccluded. Target vessel dissection or stenosis degree ≥70%, with any degree of forward flow disturbance; lumen/stent thrombus resulted in a decreased eTICI score.

Best medical management group: All the patients enrolled received standard guideline-directed medical therapy including: monitor vital signs, management of blood pressure, glucose and lipids, antithrombotic (antiplanet or anticoagulant determined by treating physician) therapy if appropriate. Intravenous thrombolysis (IVT) will be performed before EVT for patients who were eligible in compliance with the existing guideline.

The planned sample size is 488 cases, and two analyses will be conducted when the 90-day follow-up of 1/2 (244 cases) and 3/4 (366 cases) of the total sample size is completed, and the study may be terminated in advance based on clear validity or ineffectiveness. The expected duration of each subject’s enrollment is approximately 1 year. Subjects will be followed with assessments at 36 (24-48) hours, hospital discharge (or 7±1 days), 30±3 days, 90±7 days and 12 months ±14 days post randomization.

A blinded core laboratory will assess baseline imaging to confirm vessel occlusion

and determine ASPECT score, 36 (24-48) hours post-randomization to assess presence of ICH, and to measure core infarct volume and determine angiographical variables such as final reperfusion status and residual stenosis.

6. Sample size estimates

In this study, a multicenter, open, randomized, parallel control design method was used. The primary measure of efficacy was mRS score at 90±7 days after enrollment (considered as ordered variable). According to the literature data and clinical experts' opinions, the parameters were set as follows: (1) The proportion of mRS score 0-6 in control group was 3%, 4%, 10%, 17%, 16%, 12% and 38%, respectively; (2) The average treatment effect of EVT improved the outcome with the common OR value for improvement of mRS reached 1.74; (3) Two Interim analysis were considered. Adjusted level α=0.05 and power 1-β=0.90. (4) The sample size was allocated to the intervention group and the control group in a 1:1 ratio. Based on these parameters, the total sample size was 438. Considering 10% attrition rate, the final total sample size was 488 cases, 244 cases in each group.

Interim analysis will take place when 1/2 (244 cases) and 3/4 (366 cases) have completed 3-month follow-up. O'Brien-Fleming boundaries will be used at the interim analysis as follows:

There are no established techniques for the assessment of interim trial efficacy boundaries using an ordinal logistic regression model (proportional odds model). Instead, we will revert to a simple dichotomous analysis of the mRS score at 0-2 defined at 90 days from randomization. The Z-statistic for this analysis shall be derived from the normal approximation of the binomial distribution as an unadjusted two-sample test of proportions. For an RCT comparing two treatment groups with respect to a binary outcome and two interim analysis, corresponding significance levels based on O'Brien & Fleming boundary are two-sided 0.003 (stage 1), 0.018 (stage 2) and 0.044 (stage 3, final analysis).

With the result of interim analysis, DSMB will make the decision to continue or

halt the study according to the test boundaries. The study will stop prematurely for futility if the result from the interim analysis indicate that we can't achieve an effective conclusion with the current sample size. Premature stopping for early success will be achieved if the interim analysis result has proven the effect of intervention at significance level. Otherwise, the study will be continued until the predefined termination date. In interim analysis, the final sample size is allowed to be adjusted if the estimation of the primary outcome is drastically different from the actual data.

7. Analysis populations

Full Analysis Set (FAS)

Based on the principles of Intention-to-Treat analysis (ITT), all randomized subjects, either treated with medication or with EVT will be included in the full analysis set. The primary effectiveness endpoint analysis of this study will be performed on the FAS.

Per Protocol Set (PPS)

The PPS is a subset that includes all subjects who were treated with the treatment to which they were randomized and there are no clinically meaningful deviations from the protocol. Severe derivations from the protocol will be finally defined during the data auditing process, including but not limited to the followings:

1) The subject is not in line with the inclusion criteria.

2) There exist other treatments that potentially confound the appraisal of efficacy of the planned treatment.

3) Poor compliance.

4) Follow-up interval exceeds the required time window.

Secondary analysis will be conducted on the PPS, whenever its result is not consistent with the one from the FAS, a detailed analysis of the difference is needed.

Safety Analysis Set (SAS)

The safety analysis set consists of all subjects who received treatment with at least one time evaluation of safety outcome.

8. Treatment comparisons

The treatment comparison of interest in this study is to assess the safety and efficacy of best medical management plus EVT compared to best medical management alone in patients with AIS due to LVO in anterior circulation up to 24 hours from symptom onset or last seen well.

9. General considerations for data analyses

All analyses will be performed using SAS Version 9.4. All analysis output will use the treatment group naming of best medical management plus EVT group and best medical management group. All statistics were two sided with a $P<0.05$ considered significant.

Examination of Subgroups

The extent to which the treatment effect of improvement in modified Ranking scale varies across levels of each subgroup will be assessed through interaction tests.

Multiple Comparisons and Multiplicity

A single primary efficacy variable has been defined for this study, with all other efficacy variables identified as secondary or other. Similarly, only one treatment comparison is of interest in the study and therefore there are no requirements to adjust for multiple comparisons or multiple endpoints within this study.

10. Data handling conventions

Premature Withdrawal and Missing Data

If any subject withdraws prematurely from the study (prior to the final visit D90±7 days assessment), they are required to complete the withdrawal visit in the CRF. The reasons for withdrawal will be presented in a summary table. For the purposes of summaries and analysis of clinic visit data, this visit will be assigned to the next scheduled clinic visit for that subject, regardless of whether the date falls within the next visit window.

Subjects who withdraw before the end of the study, but who do provide at least one post-baseline measure for a particular endpoint, will be included in the analysis. Subjects who do not attend any visits after randomization will be excluded from analysis of any endpoint, as no post-baseline data will be available.

Outlier values will be evaluated for their validity; all data will be included unless judged to be invalid (e.g., deemed incompatible with life by Medical Monitor and/or deemed invalid by the DSMB).

Efforts will be undertaken at study sites to reduce the amount of missing data. Due to the severity of the condition and the short 90-day follow-up period, very little loss to follow-up is anticipated. For the primary efficacy endpoint, complete data will be used and missing data will not be imputed in the main analysis. Also, sensitivity analyses with missing data imputed by LOCF (Last Observation Carry Forward) and WCCF (Worst Case Carry Forward) methods will be undertaken to explore the effect of missing data on the endpoint and test the robustness of the estimate.

Event Rates

The number of events should be recorded in detail and showing the event rate in 90 days of each treatment group in summary statement.

The event rate for each treatment group will be calculated as: the sum of number of event for all the patients / the sum of number of treatment periods for all the patients.

11.Study Population

Disposition of Subjects

The number of subjects in each analysis population will be presented, subjects to be excluded from the Per Protocol population will be listed, and the total number of subjects attending each clinic visit will also be summarized by treatment group.

The number of subjects randomized, completed and prematurely withdrawn from the study will be presented for each treatment group. The primary reasons for withdrawal both prior to and post randomization will also be presented.

A data display listing and summary of deviations from the inclusion/exclusion criteria will be presented for all subjects who were either entered or randomized into the trial.

Protocol Deviations

Subject data will be examined for evidence of protocol violators in order to assess how well the protocol was followed. Inclusion and exclusion criteria are detailed in the study protocol.

Subjects who commit protocol violations will be included in the FAS Population but excluded from the Per Protocol Population. These protocol violations will be shown in a listing. Subjects can either be full or partial protocol violators. A full protocol violator is completely excluded from the Per Protocol Population. A partial protocol violator has only some data excluded. For subjects who violated the protocol during the treatment period due to unpermitted changes in the medication or prohibited concurrent medication, the analysis will only use data recorded prior to the violation. For all violations which reference the treatment period, the treatment start date will be used as the reference date.

A listing of all possible protocol violators will be produced for clinical review. The final list of subjects who are protocol violators and are therefore excluded from

the Per-Protocol population will be agreed by the study team.

Demographic and Baseline Characteristics

The following demographic information will be listed and summarized for subjects in each treatment group: age, sex, medical history, smoking history, weak-up stroke, randomization time, NIHSS score, intravenous thrombolysis, occlusion site, ipsilateral carotid artery occlusion, ASPECT score, infarct core volume and stroke subtype.

The continuous data followed normal distribution will be presented as mean and standard deviation, and the continuous data followed skewed distribution will be presented as median and interquartile range; categorical data will be presented as n(%). T-test or Wilcoxon rank sum test will be used for comparison between continuous variables, and Chi-squared tests, Fisher's exact test or Wilcoxon sum rank test will be used for comparison between categorical variables.

12. Efficacy Analyses

Primary Efficacy Analysis

The primary endpoint is the modified Ranking scale at 90-day. FAS will be the primary population for efficacy analyses. PPS will be used as secondary population for the efficacy analyses. If the results in the PPS population are inconsistent with the FAS population, detailed analysis of the inconsistent results is required.

Main Model

Based on an intention-to-treat basis, an ordinal logistic regression model with site as a random effect will be used to calculate the common odds ratio between the two treatment groups. If the proportional odds assumption for ordinal logistic regression were not satisfied, the Wilcoxon-Mann-Whitney generalized odds ratio will be calculated using assumption-free ordinal analysis. All statistics will be two-sided with $p<0.046$ considered significant.

Interactions with Subgroups

Summary tables will be produced for the predefined subgroups and interactions between treatment and these subgroups will be investigated, using an ordinal logistic regression model. A separate model will be used for each interaction to determine its significance. This will also be presented graphically on a forest plot.

The predefined subgroups including:

- Age (< 75 vs. ≥75)
- Last known well to randomization time (< 6h vs. ≥ 6h)
- Stroke severity before randomization (NIHSS<16 vs. NIHSS≥16)
- Intravenous thrombolysis or not
- Occlusion site (ICA vs. M1 segment)
- ASPECT score (< 3 points vs. ≥3 points)
- Infarct core volume (< 70ml vs. ≥70ml)
- Etiological stroke subtype (cardiac embolism vs. large artery atherosclerosis)

Secondary Efficacy Analyses

90-day mRS 0-2

The proportion of 90-day mRS 0-2 will be analyzed using a binary logistic regression model with site as a random effect. The odds ratio with 95% CI will be reported.

90-day mRS 0-3

The proportion of 90-day mRS 0-3 will be analyzed using a binary logistic regression model with site as a random effect. The odds ratio with 95% CI will be reported.

36h（24-48h）NIHSS 0-1 or decrease ≥10 from baseline

The proportion of 36h（24-48h）NIHSS 0-1 or decrease ≥10 points from baseline will be analyzed using a binary logistic regression model with site as a random effect. The odds ratio with 95% CI will be reported.

36h（24-48h）infarct volume change (by CT or MRI)

The change of 36h（24-48h）infarct volume change (by CT or MRI) will be analyzed using student t-test or Wilcoxon rank sum test as appropriate.

36h（24-48h）target artery recanalization rate (by CTA or MRA)

The 36h（24-48h）target artery recanalization rate (by CTA or MRA) will be analyzed using a logistic regression model with site as a random effect. The odds ratio with 95% CI will be reported.

13.Safety Analyses

All analyses of safety data will be carried out using the safety set (SS) population.

Primary Safety Endpoints

Rate of symptomatic intracranial hemorrhage within 48 hours from randomization (Heidelberg Bleeding Classification).

Secondary Safety Endpoints

All-cause mortality within 90 days.

Rate of any intracranial hemorrhage within 48 hours from randomization (Heidelberg Bleeding Classification).

Rate of decompressive hemicraniectomy during hospitalization.

For most bleeding events, the binary logistic model with site as a random effect will be used to compare the odds ratio between the two treatments, or Poisson regression or negative binomial regression which are more appropriate for the analysis of rare event. For all-cause mortality within 90 days, the Cox proportional hazards model with site as a random effect will be used to compare the hazard ratio between the two treatments.

Adverse Events

Adverse events (AEs) will be coded using the MedDRA coding dictionary (Version 6.0 or a later release) and grouped by system organ class (as detailed in the study protocol). Separate data display listings and summaries will be presented for adverse events that start prior to first dose of study medication (pre-treatment), whilst on study medication

(during treatment) and after the last dose of study medication (post-treatment).

Within each treatment group, the number and percentage of subjects experiencing an AE will be summarized by system organ class and preferred term and Fisher's Exact test will be used to compare the number of each grouped AE event between treatment groups. In addition, a separate summary will be provided for AEs experienced by more than 5% of subjects in either of the treatment groups.

Serious Adverse Events

Summary tables and data displays will be provided for serious adverse events (as detailed in the study protocol). In addition, all deaths and serious AE's will be documented in a case narrative format in the clinical study report.

The number of adverse events or serious adverse events occurring over the treatment period will be summarized and Fisher's Exact test will be used to compare between treatment groups.

14. References

1. De Mets DL, Furberg CD, Friedman LM. Data monitoring in clinical trials. New York: Springer; 2006.
2. Jennison C, Turnbull BW. Group sequential methods with applications to clinical trials. New York: Chapman & Hall; 2000.
3. Goyal M, Demchuk AM, Menon BK, et al. Randomized assessment of rapid endovascular treatment of ischemic stroke. N Engl J Med. 2015;372(11):1019-103

Study of Endovascular Therapy in Acute Anterior Circulation Large VeSsel Occlusive Patients with a LargE InfarCT Core: A Multicenter, Prospective, Open-Label, Blinded-Endpoint, Randomized Controlled Trial (ANGEL-ASPECT)

Statistical Analysis Plan

Principal Investigator

Zhongrong Miao, MD

Beijing Tiantan Hospital, Capital Medical University, Beijing, China

Prepared by

Yuesong Pan, PhD

Aoming Jin, PhD

Xianglong Xiang, MD

Beijing Tiantan Hospital, Capital Medical University, Beijing, China

Version 2.0

May 18, 2021

Table of Contents

1. Introduction....134

2. Study Objective....134

3. Study Endpoint(s) 134

Primary Efficacy Endpoints:....134
Secondary Efficacy Endpoint: 134
Primary Safety Endpoint....135
Secondary Safety Endpoint....135

4. Statistical Hypotheses....135

5. Design....135

6. Sample size estimates....137

7. Analysis populations....138

Full Analysis Set (FAS) 138
Per Protocol Set (PPS) 138
Safety Analysis Set (SAS) 139

8. Treatment comparisons....139

9. General considerations for data analyses....139

Examination of Subgroups....139
Multiple Comparisons and Multiplicity....139

10. Data handling conventions....140

Premature Withdrawal and Missing Data....140
Event Rates....140

11. Study Population....141

Disposition of Subjects....141
Protocol Deviations....141
Demographic and Baseline Characteristics....142

12. Efficacy Analyses....142

Primary Efficacy Analysis....142
Secondary Efficacy Analyses....143

13. Safety Analyses....144

14. References....145

1. Introduction

This statistical analysis plan (SAP) documents the planned statistical analyses for the ANGEL-ASPECT trial and is based on the protocol, together with any subsequent amendments.

This SAP is intended for the use of project team members and should be read in conjunction with the aforementioned protocol.

2. Study Objective

The primary objective of the study is to establish the efficacy of endovascular therapy (EVT) in Acute Anterior Circulation Large Vessel Occlusive (LVO) Patients with a large infarct core.

3. Study Endpoint(s)

Primary Efficacy Endpoints:

The 90 (±7) day modified Ranking scale.

Secondary Efficacy Endpoint:

1) 90 (±7) day mRS 0-2.

2) 90 (±7) day mRS 0-3.

3) 36h（24-48h）NIHSS 0-1 or decrease ≥10 from baseline.

4) 36h（24-48h）infarct volume change (by CT or MRI).

5) 36h（24-48h）target artery recanalization rate (by CTA or MRA).

Primary Safety Endpoint

•Rate of symptomatic intracranial hemorrhage within 48 hours from randomization (Heidelberg Bleeding Classification).

Secondary Safety Endpoint

1) All-cause mortality within 90 days.
2) Any intracranial hemorrhage within 48 hours from randomization (Heidelberg Bleeding Classification).
3) Decompressive hemicraniectomy during hospitalization.

4. Statistical Hypotheses

The primary outcome will be a shift of one or more categories (proportional odds analysis) on the modified Rankin scale determined at 90 days from randomization.
The primary hypothesis is:
H_A: Subjects in the group of best medical management plus EVT will have greater odds of showing improvement on the mRS at 90 days.

5. Design

This is a prospective, randomized, controlled, open label, blinded-endpoint (PROBE) study to compare the efficacy and safety of best medical management plus EVT compared to best medical management alone in Acute Anterior Circulation Large Vessel Occlusive (LVO) Patients with a large infarct core up to 24 hours from symptom onset or last seen well.

Patients who meet the inclusion criteria will be randomized to one of the following two treatment arms: best medical management plus EVT or best medical management alone. Endpoints in this prospective open label study will be assessed blinded to the

treatment assignment of the patient (PROBE design). This study will be conducted in approximately 50 sites in China.

Best medical management plus EVT group: Patients randomized to experimental group will receive EVT plus to best medical management. According to the pathological characteristics of patients and the judgment of researchers, the following treatment methods can be selected: Mechanical thrombectomy, angioplasty and arterial thrombolysis. Stent thrombectomy (Solitaire*, EMBOTRAP#, Trevo or Reco and other first-line stent thrombectomy systems) and aspiration (Penumbra system) is recommended as the first choice. If the recanalization is not successful, it can be replaced with thrombus, balloon angioplasty or stent implantation. The need for rescue treatment is defined (including, but not limited to, a decision made by the investigator based on intraoperative conditions) as follows: three times of thrombectomy with the same thrombectomy device (stent or aspiration catheter) without successful recanalization; Target vessels were successfully recanalized and then occluded. Target vessel dissection or stenosis degree ≥70%, with any degree of forward flow disturbance; lumen/stent thrombous resulted in a decreased eTICI score.

Best medical management group: All the patients enrolled received standard guideline-directed medical therapy including: monitor vital signs, management of blood pressure, glucose and lipids, antithrombotic (antiplanet or anticoagulant determined by treating physician) therapy if appropriate. Intravenous thrombolysis (IVT) will be performed before EVT for patients who were eligible in compliance with the existing guideline.

The planned sample size is 502 cases, and two analyses will be conducted when the 90-day follow-up of 1/3 (168 cases) and 2/3 (336 cases) of the total sample size is completed, and the study may be terminated in advance based on clear validity or ineffectiveness. The expected duration of each subject's enrollment is approximately 1 year. Subjects will be followed with assessments at 36 (24-48) hours, hospital discharge (or 7±1 days), 30±3 days, 90±7 days and 12 months ±14 days post randomization.

A blinded core laboratory will assess baseline imaging to confirm vessel occlusion

and determine ASPECT score, 36 (24-48) hours post-randomization to assess presence of ICH, and to measure core infarct volume and determine angiographical variables such as final reperfusion status and residual stenosis.

6. Sample size estimates

In this study, a multicenter, open, randomized, parallel control design method was used. The primary measure of efficacy was mRS score at 90±7 days after enrollment (considered as ordered variable). According to the literature data and clinical experts' opinions, the parameters were set as follows: (1) The proportion of mRS score 0-6 in control group was 3%, 4%, 10%, 17%, 16%, 12% and 38%, respectively; (2) The average treatment effect of EVT improved the outcome with the common OR value for improvement of mRS reached 1.73; (3) Two Interim analysis were considered. Adjusted level $\alpha=0.046$ (two-sided) and power $1-\beta=0.90$. (4) The sample size was allocated to the intervention group and the control group in a 1:1 ratio. Based on these parameters, the total sample size was 452. Considering 10% attrition rate, the final total sample size was 502 cases, 251 cases in each group.

Interim analysis will take place when 1/3 (168 cases) and 2/3 (336 cases) have completed 3-month follow-up. O'Brien-Fleming boundaries will be used at the interim analysis as follows:

There are no established techniques for the assessment of interim trial efficacy boundaries using an ordinal logistic regression model (proportional odds model). Instead, we will revert to a simple dichotomous analysis of the mRS score at 0-2 defined at 90 days from randomization. The Z-statistic for this analysis shall be derived from the normal approximation of the binomial distribution as an unadjusted two-sample test of proportions. For an RCT comparing two treatment groups with respect to a binary outcome and two interim analysis, corresponding significance levels based on O'Brien & Fleming boundary are two-sided 0.0002 (stage 1), 0.0123 (stage 2) and 0.046 (stage 3, final analysis).

With the result of interim analysis, DSMB will make the decision to continue or

halt the study according to the test boundaries. The study will stop prematurely for futility if the result from the interim analysis indicate that we can't achieve an effective conclusion with the current sample size. Premature stopping for early success will be achieved if the interim analysis result has already proved the effect of intervention at significance level. Otherwise, the study will be continued until the predefined termination date. In interim analysis, the final sample size is allowed to be adjusted if the estimation of the primary outcome is drastically different from the actual data.

7. Analysis populations

Full Analysis Set (FAS)

Based on the principles of Intention-to-Treat analysis (ITT), all randomized subjects, either treated with medication or with EVT will be included in the full analysis set. The primary effectiveness endpoint analysis of this study will be performed on the FAS.

Per Protocol Set (PPS)

The PPS is a subset that includes all subjects who were treated with the treatment to which they were randomized and there are no clinically meaningful deviations from the protocol. Severe derivations from the protocol will be finally defined during the data auditing process, including but not limited to the followings:

1) The subject is not in line with the inclusion criteria.

2) There exist other treatments that potentially confound the appraisal of efficacy of the planned treatment.

3) Poor compliance.

4) Follow-up interval exceeds the required time window.

Secondary analysis will be conducted on the PPS, whenever its result is not consistent with the one from the FAS, a detailed analysis of the difference is needed.

Safety Analysis Set (SAS)

The safety analysis set consists of all subjects who received treatment with at least one time evaluation of safety outcome.

8. Treatment comparisons

The treatment comparison of interest in this study is to assess the safety and efficacy of best medical management plus EVT compared to best medical management alone in patients with AIS due to LVO in anterior circulation up to 24 hours from symptom onset or last seen well.

9. General considerations for data analyses

All analyses will be performed using SAS Version 9.4. All analysis output will use the treatment group naming of best medical management plus EVT group and best medical management group. All statistics were two sided with a $P<0.05$ considered significant.

Examination of Subgroups

The extent to which the treatment effect of improvement in modified Ranking scale varies across levels of each subgroup will be assessed through interaction tests.

Multiple Comparisons and Multiplicity

A single primary efficacy variable has been defined for this study, with all other efficacy variables identified as secondary or other. Similarly, only one treatment comparison is of interest in the study and therefore there are no requirements to adjust for multiple comparisons or multiple endpoints within this study.

10. Data handling conventions

Premature Withdrawal and Missing Data

If any subject withdraws prematurely from the study (prior to the final visit D90±7 days assessment), they are required to complete the withdrawal visit in the CRF. The reasons for withdrawal will be presented in a summary table. For the purposes of summaries and analysis of clinic visit data, this visit will be assigned to the next scheduled clinic visit for that subject, regardless of whether the date falls within the next visit window.

Subjects who withdraw before the end of the study, but who do provide at least one post-baseline measure for a particular endpoint, will be included in the analysis. Subjects who do not attend any visits after randomization will be excluded from analysis of any endpoint, as no post-baseline data will be available.

Outlier values will be evaluated for their validity; all data will be included unless judged to be invalid (e.g., deemed incompatible with life by Medical Monitor and/or deemed invalid by the DSMB).

Efforts will be undertaken at study sites to reduce the amount of missing data. Due to the severity of the condition and the short 90-day follow-up period, very little loss to follow-up is anticipated. For the primary efficacy endpoint, complete data will be used and missing data will not be imputed in the main analysis. Also, sensitivity analyses with missing data imputed by LOCF (Last Observation Carry Forward) and WCCF (Worst Case Carry Forward) methods will be undertaken to explore the effect of missing data on the endpoint and test the robustness of the estimate.

Event Rates

The number of events should be recorded in detail and showing the event rate in 90 days of each treatment group in summary statement.

The event rate for each treatment group will be calculated as: the sum of number of event for all the patients / the sum of number of treatment periods for all the patients.

11.Study Population

Disposition of Subjects

The number of subjects in each analysis population will be presented, subjects to be excluded from the Per Protocol population will be listed, and the total number of subjects attending each clinic visit will also be summarized by treatment group.

The number of subjects randomized, completed and prematurely withdrawn from the study will be presented for each treatment group. The primary reasons for withdrawal both prior to and post randomization will also be presented.

A data display listing and summary of deviations from the inclusion/exclusion criteria will be presented for all subjects who were either entered or randomized into the trial.

Protocol Deviations

Subject data will be examined for evidence of protocol violators in order to assess how well the protocol was followed. Inclusion and exclusion criteria are detailed in the study protocol.

Subjects who commit protocol violations will be included in the FAS Population but excluded from the Per Protocol Population. These protocol violations will be shown in a listing. Subjects can either be full or partial protocol violators. A full protocol violator is completely excluded from the Per Protocol Population. A partial protocol violator has only some data excluded. For subjects who violated the protocol during the treatment period due to unpermitted changes in the medication or prohibited concurrent medication, the analysis will only use data recorded prior to the violation. For all violations which reference the treatment period, the treatment start date will be used as the reference date.

A listing of all possible protocol violators will be produced for clinical review. The final list of subjects who are protocol violators and are therefore excluded from

the Per-Protocol population will be agreed by the study team.

Demographic and Baseline Characteristics

The following demographic information will be listed and summarized for subjects in each treatment group: age, sex, medical history, smoking history, weak-up stroke, randomization time, NIHSS score, intravenous thrombolysis, occlusion site, ipsilateral carotid artery occlusion, ASPECT score, infarct core volume and stroke subtype.

The continuous data followed normal distribution will be presented as mean and standard deviation, and the continuous data followed skewed distribution will be presented as median and interquartile range; categorical data will be presented as n(%). T-test or Wilcoxon rank sum test will be used for comparison between continuous variables, and Chi-squared tests, Fisher's exact test or Wilcoxon sum rank test will be used for comparison between categorical variables.

12. Efficacy Analyses

Primary Efficacy Analysis

The primary endpoint is the modified Ranking scale at 90-day. FAS will be the primary population for efficacy analyses. PPS will be used as secondary population for the efficacy analyses. If the results in the PPS population are inconsistent with the FAS population, detailed analysis of the inconsistent results is required.

Main Model

Based on an intention-to-treat basis, an ordinal logistic regression model with site as a random effect will be used to calculate the common odds ratio between the two treatment groups. If the proportional odds assumption for ordinal logistic regression were not satisfied, the Wilcoxon-Mann-Whitney generalized odds ratio will be calculated using assumption-free ordinal analysis. All statistics will be two-sided with $p<0.046$ considered significant.

Interactions with Subgroups

Summary tables will be produced for the predefined subgroups and interactions between treatment and these subgroups will be investigated, using an ordinal logistic regression model. A separate model will be used for each interaction to determine its significance. This will also be presented graphically on a forest plot.

The predefined subgroups including:

- Age (< 75 vs. ≥75)
- Weak-up stroke or not
- Last known well to randomization time (< 6h vs. ≥ 6h)
- Stroke severity before randomization (NIHSS<16 vs. NIHSS≥16)
- Intravenous thrombolysis or not
- Occlusion site (ICA vs. M1 segment)
- Ipsilateral carotid artery occlusion or not
- ASPECT score (< 3 points vs. ≥3 points)
- Infarct core volume (< 70ml vs. ≥70ml)
- Etiological stroke subtype (cardiac embolism vs. large artery atherosclerosis)

Secondary Efficacy Analyses

90-day mRS 0-2

The proportion of 90-day mRS 0-2 will be analyzed using a binary logistic regression model with site as a random effect. The odds ratio with 95% CI will be reported.

90-day mRS 0-3

The proportion of 90-day mRS 0-3 will be analyzed using a binary logistic regression model with site as a random effect. The odds ratio with 95% CI will be reported.

36h（24-48h）NIHSS 0-1 or decrease ≥10 from baseline

The proportion of 36h（24-48h）NIHSS 0-1 or decrease ≥10 points from baseline will be analyzed using a binary logistic regression model with site as a random effect. The

odds ratio with 95% CI will be reported.

36h（24-48h）infarct volume change (by CT or MRI)

The change of 36h（24-48h）infarct volume change (by CT or MRI) will be analyzed using student t-test or Wilcoxon rank sum test as appropriate.

36h（24-48h）target artery recanalization rate (by CTA or MRA)

The 36h（24-48h）target artery recanalization rate (by CTA or MRA) will be analyzed using a logistic regression model with site as a random effect. The odds ratio with 95% CI will be reported.

13.Safety Analyses

All analyses of safety data will be carried out using the safety set (SS) population.

Primary Safety Endpoints

Rate of symptomatic intracranial hemorrhage within 48 hours from randomization (Heidelberg Bleeding Classification).

Secondary Safety Endpoints

All-cause mortality within 90 days.

Rate of any intracranial hemorrhage within 48 hours from randomization (Heidelberg Bleeding Classification).

Rate of decompressive hemicraniectomy during hospitalization.

For most bleeding events, the binary logistic model with site as a random effect will be used to compare the odds ratio between the two treatments, or Poisson regression or negative binomial regression which are more appropriate for the analysis of rare event. For all-cause mortality within 90 days, the Cox proportional hazards model with site as a random effect will be used to compare the hazard ratio between the two treatments.

Adverse Events

Adverse events (AEs) will be coded using the MedDRA coding dictionary (Version 6.0 or a later release) and grouped by system organ class (as detailed in the study protocol).

Separate data display listings and summaries will be presented for adverse events that start prior to first dose of study medication (pre-treatment), whilst on study medication (during treatment) and after the last dose of study medication (post-treatment).

Within each treatment group, the number and percentage of subjects experiencing an AE will be summarized by system organ class and preferred term and Fisher's Exact test will be used to compare the number of each grouped AE event between treatment groups. In addition, a separate summary will be provided for AEs experienced by more than 5% of subjects in either of the treatment groups.

Serious Adverse Events

Summary tables and data displays will be provided for serious adverse events (as detailed in the study protocol). In addition, all deaths and serious AE's will be documented in a case narrative format in the clinical study report.

The number of adverse events or serious adverse events occurring over the treatment period will be summarized and Fisher's Exact test will be used to compare between treatment groups.

14. References

1. De Mets DL, Furberg CD, Friedman LM. Data monitoring in clinical trials. New York: Springer; 2006.
2. Jennison C, Turnbull BW. Group sequential methods with applications to clinical trials. New York: Chapman & Hall; 2000.
3. Goyal M, Demchuk AM, Menon BK, et al. Randomized assessment of rapid endovascular treatment of ischemic stroke. N Engl J Med. 2015;372(11):1019-1030.

Revisions to previous SAP version

SAP version 1.0	Changes in SAP version 2.0
5. Design<br>The planned sample size is 488 cases, and two analyses will be conducted when the 90-day follow-up of 1/2 (244 cases) and 3/4 (366 cases) of the total sample size is completed.	5. Design<br>The planned sample size is 502 cases, and two analyses will be conducted when the 90-day follow-up of 1/3 (168 cases) and 2/3 (336 cases) of the total sample size is completed.
6. Sample size estimates<br>…The average treatment effect of EVT improved the outcome with the common OR value for improvement of mRS reached 1.74; (3) Two Interim analysis were considered. Adjusted level α=0.05 and power 1-β=0.90. (4) The sample size was allocated to the intervention group and the control group in a 1:1 ratio. Based on these parameters, the total sample size was 438. Considering 10% attrition rate, the final total sample size was 488 cases, 244 cases in each group.<br>Interim analysis will take place when 1/2 (244 cases) and 3/4 (366 cases) have completed 3-month follow-up. O'Brien-Fleming boundaries will be used at the interim analysis as follows:<br>… For an RCT comparing two treatment groups with respect to a binary outcome and two interim analysis, corresponding significance levels based on O'Brien & Fleming boundary are two-sided 0.003 (stage 1), 0.018 (stage 2) and 0.044 (stage 3, final analysis).	6. Sample size estimates<br>…The average treatment effect of EVT improved the outcome with the common OR value for improvement of mRS reached 1.73; (3) Two Interim analysis were considered. Adjusted level α=0.046 (two-sided) and power 1-β=0.90. (4) The sample size was allocated to the intervention group and the control group in a 1:1 ratio. Based on these parameters, the total sample size was 452. Considering 10% attrition rate, the final total sample size was 502 cases, 251 cases in each group.<br>Interim analysis will take place when 1/3 (18 cases) and 2/3 (336 cases) have completed 3-month follow-up. O'Brien-Fleming boundaries will be used at the interim analysis as follows:<br>… For an RCT comparing two treatment groups with respect to a binary outcome and two interim analysis, corresponding significance levels based on O'Brien & Fleming boundary are two-sided 0.0002 (stage 1), 0.0123 (stage 2) and 0.046 (stage 3,

	final analysis).
The predefined subgroups including:<br>· Age (< 75 vs. ≥75)<br>· Last known well to randomization time (< 6h vs. ≥ 6h)<br>· Stroke severity before randomization (NIHSS<16 vs. NIHSS≥16)<br>· Intravenous thrombolysis or not<br>· Occlusion site (ICA vs. M1 segment)<br>· ASPECT score (< 3 points vs. ≥3 points)<br>· Infarct core volume (< 70ml vs. ≥70ml)<br>· Etiological stroke subtype (cardiac embolism vs. large artery atherosclerosis)	The predefined subgroups including:<br>· Age (< 75 vs. ≥75)<br>· Weak-up stroke or not<br>· Last known well to randomization time (< 6h vs. ≥ 6h)<br>· Stroke severity before randomization (NIHSS<16 vs. NIHSS≥16)<br>· Intravenous thrombolysis or not<br>· Occlusion site (ICA vs. M1 segment)<br>· Ipsilateral carotid artery occlusion or not<br>· ASPECT score (< 3 points vs. ≥3 points)<br>· Infarct core volume (< 70ml vs. ≥70ml)<br>· Etiological stroke subtype (cardiac embolism vs. large artery atherosclerosis)

大梗死核心的前循环大血管闭塞患者血管内治疗研究——多中心、前瞻性、开放标签、终点盲法、随机对照研究（ANGEL-ASPECT）

Study of Endovascular Therapy (EVT) in Acute **A**nterior Circulation Large Ve**S**sel Occlusive (LVO) **P**atients with a larg**E** infar**CT** core：A Multicenter, Prospective, Open-label, Blinded-Endpoint, Randomized Controlled Trial（ANGEL-ASPECT）

研究方案

研究负责单位： 首都医科大学附属北京天坛医院

主要研究者： 缪中荣 教授

共同主要研究者：任泽光、Vitor Mendes Pereira

研究方案版本号： V 7.1

注册号：NCT04551664

研究版本日期： 2021 年 5 月 18 日

目 录

签名页 **169**

研究方案摘要 **170**

缩略词表 **176**

一、研究背景 **179**

1.1 大梗死核心体积血管内治疗的合理性..179

1.2 评价大梗死核心体积的影像方法..181

1.3 ASPECTS 和梗死核心体积选择 182

1.4 大梗死核心体积血管内治疗的时间窗..182

1.5 ANGEL-ASPECT 的试验设计..183

二、研究目的 **184**

2.1 主要研究目的..184

2.2 次要研究目的..184

三、研究设计 **184**

3.1 研究类型..184

3.2 随机分组..185

3.3 访视时间点..185

3.4 盲法设计..185

四、研究对象 **186**

4.1 纳入标准......186
4.1.1 研究中心纳入标准......186
4.1.2 受试者一般纳入标准......186
4.1.3 受试者影像纳入标准......186
4.2 排除标准......187
4.2.1 研究中心排除标准......187
4.2.2 受试者一般排除标准......187
4.2.3 受试者影像排除标准......188
五、影像方案......188
5.1 基线影像......188
5.2 术中及复查影像......190
5.3 核心影像实验室......191
六、治疗方案......191
6.1 血管内治疗（EVT）......191
6.2 最佳内科治疗（BMM）......192
七、评价指标......193
7.1 主要有效性评价指标......193
7.2 次要有效性评价指标......193
7.3 主要安全性评价指标......193

7.4 次要安全性评价指标......193

八、数据采集和研究流程......194

8.1 筛选和入组......195

8.2 术中数据收集......195

8.3 住院期间访视......196

8.4 随机化后第 30 (±3)天，90 (±7)天和 1 年(±14 天)随访......196

8.5 非计划访视......197

8.6 研究流程表......198

九、项目风险的预评估及风险处置......199

9.1 不良事件监测......199

9.2 不良事件定义......199

9.2.1 不良事件......199

9.2.2 严重不良事件......199

9.3 不良事件的记录......200

9.4 不良事件与试验的关系评定......200

9.5 研究者在安全性报告方面的职责......200

9.5.1 不良事件......200

9.5.2 严重不良事件......201

9.5.3 风险处置......201

十、统计学处理 202

10.1 样本量计算......202

10.2 数据采集与录入......202

10.2.1 研究者填写纸质 CRF 202

10.2.2 研究协调员（Clinical research coordinator, CRC）录入 EDC 系统 203

10.2.3 研究者审核通过后提交 EDC 系统表单......203

10.2.4 CRA 通过 EDC 系统进行数据质询......203

10.2.5 EDC 系统数据导出数据库......203

10.3 统计分析......204

10.3.1 统计分析数据集......204

10.3.2 统计分析方法......205

10.4 期中分析......206

十一、伦理原则 206

11.1 伦理原则......206

11.2 法律法规......206

11.3 知情同意书......207

11.4 审查机构/独立的伦理委员会(IRB/IEC)......207

十二、资料保密与结果公布 208

十三、研究组织 208

13.1 研究组织构成......208

13.2 中心培训，认证......210

十四、研究的质量控制与质量保证......211

14.1 研究者职责......211

14.2 研究监查......212

十五、资料保存......212

十六、数据安全监查......213

十七、研究注册及文章发表......213

17.1 研究总结和结果的登记......213

17.2 研究结果的发表......213

十八、数据的所有权和使用......214

18.1 研究结果的所有权......214

18.2 收集数据的使用......214

十九、项目赞助及利益冲突......215

二十、参考文献......215

二十一、附表......218

附录 1. 改良 Rankin 量表......218

附录 2. 脑缺血扩展治疗（eTICI）量表......220

附录 3. 美国国立卫生研究院卒中量表（NIHSS）......221

附录 4. 欧洲五维健康量表（EQ-5D-5L）......226

附录 5. 海德堡出血分型......228

附录 6. Alberta 卒中项目早期 CT 评分（Alberta Stroke Program Early CT Score, ASPECTS）......229

签名页

课题承担单位：首都医科大学附属北京天坛医院

主要研究者：

我参与本项临床研究，并将根据ICH-GCP规定，认真履行研究者职责。

我已阅读过此方案，本项研究将根据《赫尔辛基宣言》和中国GCP规定的道德、伦理和科学原则进行。

主要研究者（签名）:

日期： 年 月 日

研究方案摘要

**研究名称**		大梗死核心的前循环大血管闭塞患者血管内治疗研究——多中心、前瞻性、开放标签、终点盲法、随机对照研究<br>Study of Endovascular Therapy (EVT) in Acute Anterior Circulation Large Vessel Occlusive (LVO) Patients with a large infarct core：A Multicenter, Prospective, Open-label, Blinded-Endpoint, Randomized Controlled Trial
**研究简称**		ANGEL-ASPECT
**承担单位**		首都医科大学附属北京天坛医院
**研究中心**		约 50 家
**研究目的**	**主要目的**	评价最佳内科治疗（Best medical management，BMM）联合 EVT 较单独 BMM，是否能够改善发病 24 小时内、伴大梗死核心的急性前循环大血管闭塞患者的神经功能预后。
	**次要目的**	评价 BMM 联合 EVT 较单独 BMM，是否增加发病 24 小时内、伴大梗死核心的急性前循环大血管闭塞患者症状性颅内出血（symptomatic intracranial hemorrhage，sICH）的风险。
**研究设计**		多中心、前瞻性、开放标签、终点盲法、随机对照研究
**随机化**		本研究采用简单随机的方法，将受试者按照 1:1 比例由中央网络随机系统随机分配至 BMM 联合 EVT 组或单独 BMM 组。
**样本量**		计划样本量为 502 例，当完成总样本量 1/3（168 例）、2/3（336 例）受试者 90 天随访时将进行两次期中分析。
**有效性终点**	**首要终点**	90（±7）天改良 Rankin 评分（modified Rankin scale, mRS）
	**次要终点**	(1) 随机化 90（±7）天 mRS 0-2 比例<br>(2) 随机化 90（±7）天 mRS 0-3 比例

		(3) 随机化 36（±12）小时 NIHSS 0-1 分或较基线减少≥10 分比例<br>(4) 随机化 7（±1）天/出院 NCCT 或 36（±12）小时 MRI 评价的梗死体积较基线的变化<br>(5) 随机化 36（±12）小时 CTA 或 MRA 判定的闭塞血管再通率
**安全性终点**	**首要安全性终点**	随机化 48 小时内发生 sICH 的概率（海德堡出血分型）
	**次要安全性终点**	(1) 随机化 90（±7）天全因死亡率<br>(2) 随机化 48 小时内任何类型的颅内出血（海德堡出血分型）的概率<br>(3) 住院期间实行去骨瓣减压术的概率
**入排标准**	**纳入标准**	研究中心纳入标准<br>（1） 具有急诊科室和收治卒中患者的神经科病房；<br>（2） 具有全天候（24 小时×7 天）卒中急救团队；<br>（3） 有开展急性缺血性卒中静脉溶栓和血管内治疗的能力。<br>受试者一般纳入标准<br>（1）年龄 18-80 岁；<br>（2）临床诊断为急性缺血性卒中；<br>（3）此次卒中前 mRS 0-1；

		（4）接受随机化前 NIHSS 6-30 分；<br>（5）卒中发病后 24h 内能够完成随机（发病时间定义为最后正常时间）；<br>（6）受试者本人或合法代理人签署知情同意书。<br>受试者影像纳入标准<br>（1） CTA 或 MRA 证实的颈内动脉（Internal Carotid Artery ，ICA)颅内段或大脑中动脉（ Middle Cerebral Artery, MCA）M1 段闭塞；<br>（2） 头颅影像证实低 ASPECTS 评分（基于头颅 NCCT)或大梗死核心(基于 CTP 的 rCBF<30%或基于 MRI 的 ADC<620)，符合以下标准之一：<br>1) ASPECTS 3-5；<br>2) ASPECTS>5 (6-24小时)，梗死核心体积70ml-100ml；<br>3) ASPECTS<3，梗死核心体积70ml-100ml。
	**排除标准**	研究中心排除标准<br>（1） 每年急性缺血性卒中血管内治疗小于 20 例的医院；<br>（2） 无法配合研究方案完成研究工作。<br>受试者一般排除标准<br>（1） 已知妊娠期或哺乳期女性，或随机化前妊娠试验阳性；

		（2） 已知对造影剂严重过敏（非轻度皮疹性过敏）；<br>（3） 药物难以控制的顽固性高血压（定义为持续收缩压>185mmHg 或舒张压>110mmHg）；<br>（4） 已知的出血倾向（包括但不限于）：血小板计数 < 100X10^9/L；48 小时内接受肝素治疗，且 aPTT≥35s；正在口服华法林，且 INR > 1.7；（没有凝血功能异常病史或怀疑的凝血功能异常的患者在入组前不需要等待 INR 或 aPTT 的实验室检查结果）；<br>（5） 近 1 个月曾进行实质器官手术、活检术；<br>（6） 近 1 个月有任何活动性出血或近期出血（胃肠道、尿路出血等）；<br>（7） 正在进行血液透析或腹膜透析；已知严重肾功能不全（肾小球滤过率<30ml/min 或血肌酐>220mmol/L（2.5mg/dl））；<br>（8） 脑肿瘤（存在占位效应）；<br>（9） 预期生存时间小于 1 年（如合并恶性肿瘤、严重心肺疾病等）；<br>（10）已经参与可能会对结局评估产生影响的其他干预性临床研究；<br>（11）研究者认为不适合参与本研究或者可能会对患者造成显著风险的其他情形（如因精神疾患、认

		知或情绪障碍无法理解和/或服从研究程序和/或随访）。<br>受试者影像排除标准<br>（1） 已出现大脑中线移位或脑疝，脑室占位效应；<br>（2） 急性颅内出血；<br>（3） 新发双侧急性卒中或颅内多流域大血管闭塞。
**治疗方案**	**试验组**	试验组将在 BMM 基础上接受 EVT 治疗
	**对照组**	对照组仅接受 BMM
**访视计划**		随机化当天、36±12 小时、7±1 天/出院（以先发生时间为准）、30±3 天、90±7 天、12 个月±14 天时进行访视
**亚组分析**		本研究将根据以下变量进行亚组分析：<br>（1） 年龄（＜70 岁 vs ≥70 岁）；<br>（2） 是否醒后卒中；<br>（3） 最后正常时间至随机化时间（＜6h vs ≥ 6h）；<br>（4） 随机化前卒中严重程度（NIHSS<16 vs NIHSS≥16）;<br>（5） 是否接受静脉溶栓；<br>（6） 血管闭塞部位（ICA 颅内段 vs M1 段）；<br>（7） 是否合并同侧颈动脉闭塞；<br>（8） ASEPECT 评分（＜3 分 vs ≥3 分）；<br>（9） 梗死体积（＜70ml vs ≥70ml）；<br>（10）卒中亚型(心源性栓塞型 vs 大动脉粥样硬化型）。

**研究期限**	2020 年 8 月-2023 年 10 月（其中 2022 年 10 月完成入组）

缩略词表

缩写	英文	中文
ADC	Apparent Diffusion Coefficient	表观弥散系数
AE	Adverse Event	不良事件
aPTT	Activated Partial Thromboplastin Time	活化部分凝血活酶时间
ASPECTS	Alberta Stroke Program Early CT Score	Alberta 卒中项目早期 CT 评分
BMM	Best Medical Management	最佳药物治疗
CEC	Clinical Events Adjudication Committee	临床终点事件评审委员会
CI	Confidence Interval	置信区间
CRA	Clinical Research Associate	临床检查员
CRC	Clinical research coordinator	临床协调员
CRF	Case Report Form	病例报告表
CSA	Chinese Stroke Association	中国卒中学会
CT	Computer Tomography	计算机断层扫描
CTA	Computed Tomography Angiography	计算机断层扫描血管造影术
CTP	Computed Tomography Perfusion Imaging	计算机灌注扫描成像术
DAWN	DWI or CTP Assessment with Clinical Mismatch in the Triage of Wake-Up and Late Presenting Strokes Undergoing Neurointervention with Trevo	应用 Trevo 装置血管内治疗经影像评估联合临床不匹配筛选的醒后卒中和晚就诊卒中患者
DEFUSE 3	Endovascular Therapy Following Imaging Evaluation for Ischemic Stroke 3	血管内治疗经影像筛选的急性缺血性卒中 3
DICOM	Digital Imaging and Communications in Medicine	医学数字成像与通信
DSA	Digital Subtraction Angiography	数字减影血管造影术
DSMB	Data Safety Monitoring Board	数据安全监查委员会
DWI	Diffusion Weighted Imaging	弥散加权成像
EDC	Electronic Data Capture	电子数据捕获
EQ-5D-5L	EuroQoL 5-Dimensions 5-Level questionnaire	欧洲五维健康量表
eTICI	Expanded Thrombolysis in Cerebral Infarction	扩展脑梗死溶栓分级
EVT	Endovascular Therapy	血管内治疗
FAS	Full Analysis Set	全分析数据集
FLAIR	FLuid Attenuated Inversion Recovery	液体衰减翻转恢复

GCP	Good Clinical Practice	药物临床试验质量管理规范
GRE	Gradient Recalled Echo	梯度回波序列
GSR-ET	German Stroke Registry – Endovascular Treatment	德国卒中登记-血管内治疗
ICA	Internal Carotid Artery	颈内动脉
ICH	The International Council for Harmonisation of Technical Requirements for Pharmaceutical for Human Use	国际人用药品注册技术协调会
ICMJE	International Committee of Medical Journal Editors	国际医学杂志编辑委员会
IEC	Institutional Ethics Committee	独立伦理委员会
INR	International Normalized Ratio	国际标准化比值
IRB	Institutional Review Board	机构审查委员会
ITT	Intention-To-Treat	意向性分析
IV	Intravenous	静脉内
LICV	Large Infarct Core Volume	大梗死核心体积
LLC	Limited Liability Company	有限责任公司
LVO	Large Vessel Occlusive	大血管闭塞
MCA	Middle Cerebral Artery	大脑中动脉
MM	Medical Management	药物治疗
MRA	Magnetic Resonance Angiography	磁共振血管成像
MRI	Magnetic Resonance Imaging	核磁共振成像
mRS	Modified Rankin Scale	改良 Rankin 量表
NCCT	Non-contrast computed tomography	非增强计算机断层扫描
NCSS	Number Cruncher Statistical System	数字运算统计系统
NIHSS	National Institute of Health stroke scale	美国国立卫生研究院卒中量表
NMPA	National Medical Products Administration	国家药品监督管理局
OR	Odd Ratio	比值比
PASS	Power Analysis and Sample Size	功效分析和样本大小软件
PPS	Per Protocol Set	符合方案集
PROBE	Prospective, Randomized, Open-label, Blinded End-point	前瞻性、随机、开放标签、盲法终点
PWI	perfusion weighted imaging	灌注加权成像
SAE	Serious Adverse Event	严重不良反应
SAP	Statistical Analysis Plan	统计分析计划
SAS	Safety Analysis Set	安全性数据集

SELECT	Optimizing Patient's Selection for Endovascular Treatment in Acute Ischemic Stroke	急性缺血性卒中血管内治疗患者优化选择研究
sICH	Symptomatic intracranial hemorrhage	症状性颅内出血
THRACE	Mechanical thrombectomy after intravenous alteplase versus alteplase alone after stroke	卒中后静脉溶栓联合机械取栓与单独静脉溶栓对比研究
TICI	Thrombolysis In Cerebral Infarction	脑梗死溶栓分级
T-NICE	Tiantan Neuroimaging Center of Excellence	天坛神经影像研究中心
TOAST	Trial of ORG 10172 in Acute Stroke Treatment	急性卒中 Org10172 治疗试验

一、研究背景

多项大型临床试验证实了血管内治疗（Endovascular therapy, EVT）对不同时间窗内急性大血管闭塞（Large vessel occlusion，LVO）患者的有效性。[1,2] 这些研究包括 6 小时时间窗内使用 ASPECTS≥ 6 筛选的患者 [3-7]，以及 6 小时至 16 或 24 小时时间窗内符合应用 Trevo 装置血管内治疗经影像评估联合临床不匹配筛选的醒后卒中和晚就诊卒中患者（DWI or CTP Assessment with Clinical Mismatch in the Triage of Wake-Up and Late Presenting Strokes Undergoing Neurointervention with Trevo，DAWN）研究或血管内治疗经影像筛选的急性缺血性卒中（Endovascular Therapy Following Imaging Evaluation for Ischemic Stroke，DEFUSE 3）研究标准的患者。[8,9] 此后，开展了很多尝试拓展急性 LVO 患者血管内治疗适应症的临床试验。伴有大梗死核心体积（large infarct core volume，LICV）的患者是否适合血管内治疗，就是热点之一。

1.1 大梗死核心体积血管内治疗的合理性

一些回顾性研究、前瞻性研究和荟萃分析表明，LICV 患者可能通过 EVT 获益。在早期主要的随机对照试验中，卒中后静脉溶栓联合机械取栓与单独静脉溶栓对比研究（Mechanical thrombectomy after intravenous alteplase versus alteplase alone after stroke，THRACE）亚组分析提示，在 53 名 DWI 体积>70 ml 的受试者中，EVT 组有 12 人(22.6%)达到 90 天良好临床预后(mRS≤2)。[10,11] 德国卒中血管内治疗前瞻性登记研究(German Stroke Registry – Endovascular Treatment, GSR-ET)也显示，在 152 例 ASPECTS <6 的取栓患者中，22%的患者在 90 天内达到 mRS 0-2 的功能独立。[12] 多项血管内治疗卒中试验评估高效再灌注（Highly Effective Reperfusion evaluated in Multiple Endovascular Stroke Trials，HERMES）

研究对六项试验的数据进行了荟萃分析显示，在 ASPECTS 0-4 或 DWI 核心体积≥70 ml 的患者中，EVT 组相比对照组 90 天神经功能独立（mRS 0-2）率分别为 25% vs. 14%和 30% vs. 20%。[13,14]

急性缺血性卒中血管内治疗患者优化选择研究（Optimizing Patient’s Selection for Endovascular Treatment in Acute Ischemic Stroke, SELECT）共纳入了 105 例 ASPECTS≤5 或 CTP 梗死核心体积≥50 ml 的患者(其中 62 例接受 EVT)，预先设定的二次分析结果显示，EVT 组功能独立的患者达到 31%，而对照组仅为 14%。[15] 两组死亡、神经功能下降和症状性颅内出血(symptomatic intracranial hemorrhage，sICH)的发生率相似。此外，与药物治疗（Medical management，MM）相比，EVT 还与较少的梗死体积增加相关 (44 vs. 98 mL;p=0.006)，并且 EVT 组有更小的最终梗死体积(97 ml vs. 190 ml; p=0.001)。

在一项包括 17 项研究、1378 例 ASPECTS 0-6 的患者 (1194 例 EVT, 184 例 MM)的荟萃分析中，EVT 后 30.1%的患者达到 mRS 0-2，MM 组仅为 3.2% (OR 4.76, p=0.01)。[16] 与之前的研究相比，MM 组良好预后率明显降低（HERMES: 14%, SELECT: 14%），这可能是受试者的基线特征失衡所致，例如 MM 组年龄较大（75 岁 vs. 68.7 岁），NIHSS 较高（19 vs. 18），静脉溶栓率较低（47.8% vs. 56.8%），症状发作至入院时间较长(130 min vs. 115 min)。该项分析还提示，成功再灌注的患者(TICI 2b-3 级)较未成功的患者更容易达到 mRS 0-2 (OR 5.2, p=0.001)。另一项荟萃分析纳入了 12 项包含 LICV 患者的研究 (ASPECTS <6 或梗死核心体积≥50ml)，结果提示 EVT 提高了 mRS 0-2 比率（25% vs. 7%; pooled OR: 4.39, 95% CI: 2.53 ~ 7.64）并降低了死亡率 (23% vs. 33%;pooled OR: 0.53, 95% CI: 0.40~0.71)。[17]

在一项匹配病例对照研究中共纳入了 56 例(28 对)ICA、M1 或 M2 闭塞且梗死核心体积>50ml 的患者，结果提示 EVT 组有更高的神经功能独立率(90 天 mRS 0-2, 25% vs 0%; p=0.04)和更小的最终梗死体积 (87 ml vs. 242 ml; p<0.001)。[18]MM 组和 EVT 组分别有 1 例 (4%)和

2 例 (7%)患者出现 PH2 型脑出血（p>0.99）。EVT 组的去骨瓣减压比例(7% vs. 21%;P =0.10)和 90 天死亡率（29% vs 48%;p=0.75）较低。对基线梗死核心体积大于 70 ml（12 对）的患者进行敏感性分析，结果显示 EVT 组最终梗死体积显著减少（110 ml vs. 319 ml; p<0.001)，但 mRS 评分整体分布未见显著改善（p=0.18）。

一项观察性队列研究连续纳入了 170 名前循环 LVO 且 ASPECTS≤5 的患者(99 例 EVT, 71 例 MM)，结果显示当 EVT 组再通失败或不完全开通 (TICI 0-2b)时，临床结局仍明显优于单独 MM 组(median mRS 5，IQR 4-6 vs. 5-6, p=0.03)，即使再通失败(TICI 0-2a)的患者的预后也不比单独 MM 组的患者差。[19]

1.2 评价大梗死核心体积的影像方法

一般来说，大梗死核心的影像评估方法有两种，一种是基于 CT/MRI-ASPECTS 的半定量评估，另一种是基于 CTP/MRI 并借助自动化人工智能软件的定量评估。ASPECTS 评分因其简单、易推广性是临床上最常使用的评估梗死体积的工具。ASPECTS < 6 被公认为是“大梗死核心”。然而，多项研究显示，ASPECTS 评估的一致性较低。[20,21] 不准确的 ASPECTS 可能会导致在试验组和对照组之间错误分配受试者，弱化试验结论。使用 CTP/MRI 并借助自动化人工智能软件测定的缺血核心体积能弥补 ASPECTS 评分一致性差的缺陷。

值得注意的是，CTP/MRI 测定的梗死体积与 ASPECTS 之间的相关性尚不明确，因此，在临床试验中筛选 LICV 患者的最佳影像模式有待进一步探索。一项荟萃分析比较了使用两种影像模式筛选的 LICV 患者的预后，发现使用 ASPECTS 或 CTP 筛选 LICV 时预后不存在差异性。[17] 另一项研究发现 ASPECTS 与 CTP/MRI 体积之间存在良好的相关性[22]，但也有研究发现两者无相关性[15,17,23]。出于加快入组进度的考虑，本研究允许使用非增强计算机断层扫描（Non-contrast computed tomography，NCCT）-ASPECTS 和/或 CTP/MRI 两种影像模式

来筛选 LICV 患者。

1.3 ASPECTS 和梗死核心体积选择

一项纳入了 17 项研究、1378 例 EVT 患者的荟萃分析显示，ASPECTS 6、5、4 分和 0-4 分患者的 mRS 0-2 比率分别为 37.7%、33.3%、22.1%和 17.1%。[16]Mourand 和 Inoue 等人的研究显示，ASPECTS 0-3 的患者在 EVT 后仅 16%-20%达到良好的预后。[24,25]另一项荟萃分析显示当 ASPECTS 0-2 时，更倾向于 MM 获益，而非 EVT[13]。可见，随着 ASPECTS 的降低，EVT 的获益逐渐下降，特别是当 ASPECTS < 3 时由于梗死核心体积过大、可挽救的脑组织较少可能导致 EVT 无效。[13,26,27]因此，本研究将入组患者的 ASPECTS 限定在 3-5 分。

当采用 CTP/MRI 定量评估梗死核心体积时，对于“大梗死核心”应该定义为 50ml 还是 70ml 以上仍然存在一些争议，本研究将梗死核心体积 > 70ml 定义为 LICV。同 ASPECTS 0-2 的患者一样，对梗死核心体积过大的患者进行 EVT 也可能难以获益。有研究提示，如果 CTP 测定的梗死核心体积超过 100 ml 或 150 ml，EVT 则没有益处[14,15]。因此，当受试者仅依据 CTP/MRI 评估的梗死核心体积入组时，本研究将梗死核心体积的入组标准限定在 70ml-100ml。

1.4 大梗死核心体积血管内治疗的时间窗

脑卒中超急性期患者，从影像到再通时间越长 ASPECTS 评分受累区域越多，提示更快的再通对患者获益的重要性。[28]Cagnazzo 等人的荟萃分析表明，在 ASPECTS 0-6 的患者中，从发病到再灌注的时间越短，EVT 后神经功能独立的可能性越大。[16]SELECT 研究发现，LICV 患者随着治疗时间延长，良好功能预后会逐渐下降，12 小时后 EVT 获益的可能性较低。[15]这提示对于 LICV 患者来说，越早进行 EVT 可能越获益。然而，近期的一项荟萃分析

显示，在 EVT 时间窗<6 小时、<12 小时和<24 小时的研究中，LICV 患者的预后没有显著差异。[17] 其原因除了 > 6 小时的患者数量有限导致未发现统计学差异之外，也可能是由于随着时间的推移 MM 的效果也在下降，从而使 EVT 的疗效相对保留。总之，对于 LICV 患者，晚时间窗 EVT 是否同样获益值得期待，因此本研究设定时间窗为 0-24 小时。

1.5 ANGEL-ASPECT 的试验设计

ANGEL-ASPECT 试验是一项由研究人员发起的 PROBE 研究，旨在探讨在发病 0-24 小时内、ASPECTS 3-5 或梗死核心体积 70ml-100ml 的前循环 LVO 患者中进行 EVT 的有效性和安全性。本研究在应用多种影像模式筛选 LICV 患者的同时又对 ASPECTS 或梗死核心体积进行了一定的限制，其主要目的是希望在纳入尽可能多的 LICV 患者的同时降低 EVT 带来的风险。在对受试者进行筛选时，ANGEL-ASPECT 以 NCCT-ASPECT 评分 3-5 分为主要筛选标准，以梗死核心体积 70ml-100ml 作为辅助筛选标准，即（1）当发病 24 小时内 NCCT-ASPECTS 符合 3-5 分时，入选的患者不受梗死核心体积限制；（2）当 NCCT-ASPECTS 符合 0-2 分时，如果患者的梗死核心体积是 70ml-100ml，允许纳入研究；（3）在发病 6 - 24 小时内，当 NCCT-ASPECTS>5 且梗死核心体积符合 70ml-100 ml 时，允许纳入研究。

在亚组分析中，本研究将重点放在年龄、LKW 到随机时间、NIHSS 评分、静脉溶栓、闭塞部位、ASPECT 评分、梗死核心体积和卒中类型等方面。

ANGEL-ASPECT 是目前唯一一项在中国开展的针对 LICV 患者的随机对照研究，本研究的结果将明确 EVT 在中国 LICV 患者中是否有效、安全。

二、 研究目的

2.1 主要研究目的

评价最佳内科治疗（Best medical management，BMM）联合 EVT 较单独 BMM，是否能够改善发病 24 小时内、伴大梗死核心的急性前循环大血管闭塞患者的神经功能预后。

2.2 次要研究目的

评价 BMM 联合 EVT 较单独 BMM，是否增加发病 24 小时内、伴大梗死核心的急性前循环大血管闭塞患者症状性颅内出血（symptomatic intracranial hemorrhage，sICH）的风险。

三、 研究设计

3.1 研究类型

本研究为多中心、前瞻性、开放标签、终点盲法、随机对照研究 (PROBE 设计) (图 1)。

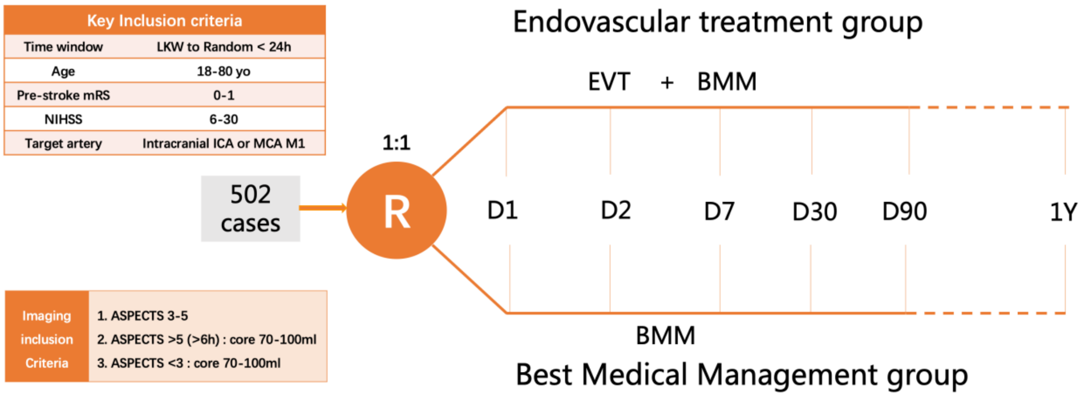

图 1 研究设计图

3.2 随机分组

本研究随机分组采用简单随机的方法，随机代码将由中央随机化系统生成，在线提供 24h 实时随机。符合纳排标准并获得知情同意的受试者，将由研究者依据入组顺序向中央随机化系统索取受试者随机代码，按 1:1 比例随机分配到以下治疗组：

- EVT+BMM 组：受试者在 BMM 的基础上接受 EVT，EVT 首选可回收支架取栓或接触抽吸取栓；
- BMM 组：受试者仅接受 BMM。

3.3 访视时间点

- 面对面访视：第 1 天（随机化当天），36（±12）小时，第 7（±1）天/出院当天（以先发生时间为准）
- 电话访视：第 30（±3）天，第 90（±7）天，第 12 个月（±14 天）

3.4 盲法设计

(1) 仅患者本人及进行治疗的医生知晓随机化分组结果，研究终点相关的基线及住院期间访视的评价指标应由对患者分组和实际治疗情况未知的研究者进行评价。

(2) 终点指标的访视由经过培训的第三方人员，在对患者随机分组和实际治疗情况未知的前提下进行标准化电话访视。所有随访电话均录音并形成随访报告。

(3) 所有研究相关影像资料将回收进行中心化判读。各访视点影像独立判读，判读者对于患者基线情况、所接受治疗（EVT 治疗过程影像除外）及预后情况未知。

四、 研究对象

4.1 纳入标准

4.1.1 研究中心纳入标准

（1） 具有急诊科室和收治卒中患者的神经科病房；

（2） 具有全天候（24 小时×7 天）卒中急救团队；

（3） 有开展急性缺血性卒中静脉溶栓和血管内治疗的能力。

4.1.2 受试者一般纳入标准

（1） 年龄 18-80 岁；

（2） 临床诊断为急性缺血性卒中；

（3） 此次卒中前 mRS 0-1；

（4） 接受随机化前 NIHSS 6-30 分；

（5） 卒中发病后 24h 内能够完成随机；

（6） 受试者本人或合法代理人签署知情同意书。

4.1.3 受试者影像纳入标准

（1） CTA 或 MRA 证实的颈内动脉（Internal Carotid Artery，ICA）颅内段或大脑中动脉（Middle Cerebral Artery, MCA）M1 段闭塞；

（2） 头颅影像证实为低 ASPECTS 评分（基于头颅 NCCT）或大梗死核心(定义为 CTP 的 rCBF<30%或基于 MRI 的 ADC<620)，符合以下标准之一：

1) ASPECTS 3-5；

2) ASPECTS>5 (6-24小时)，梗死核心体积70ml-100ml ；

3) ASPECTS<3，梗死核心体积70ml-100ml。

4.2 排除标准

4.2.1 研究中心排除标准

（1） 每年急性缺血性卒中血管内治疗小于 20 例的医院；

（2） 无法配合研究方案完成研究工作。

4.2.2 受试者一般排除标准

（1） 已知处于妊娠期或哺乳期的女性，或随机化前妊娠试验阳性；

（2） 已知对造影剂严重过敏（非轻度皮疹性过敏）；

（3） 药物难以控制的顽固性高血压（定义为持续收缩压>185mmHg 或舒张压>110mmHg）；

（4） 已知的出血倾向（包括但不限于）：血小板计数 < $100X10^9/L$；48 小时内接受肝素治疗，且 aPTT≥35s；正在口服华法林，且 INR > 1.7（没有凝血功能异常病史或怀疑的凝血功能异常的患者在入组前不需要等待 INR 或 aPTT 的实验室检查结果）；

（5） 近 1 个月曾进行实质器官手术、活检术；

（6） 近 1 个月有任何活动性出血或近期出血（胃肠道、尿路出血等）；

（7） 正在进行血液透析或腹膜透析；已知严重肾功能不全（肾小球滤过率<30ml/min 或血肌酐>220mmol/L（2.5mg/dl））；

（8） 脑肿瘤（存在占位效应）；

（9） 预期生存时间小于 1 年（如合并恶性肿瘤、严重心肺疾病等）；

（10）已经参与可能会对结局评估产生影响的其他干预性临床研究；

（11）研究者认为不适合参与本研究或者可能会对患者造成显著风险的其他情形（如因精神疾患、认知或情绪障碍无法理解和/或服从研究程序和/或随访）。

4.2.3 受试者影像排除标准

（1） 已出现大脑中线移位或脑疝，脑室占位效应；

（2） 急性颅内出血；

（3） 新发双侧急性卒中或颅内多流域 LVO。

五、 影像方案

5.1 基线影像

在项目启动前，全部研究者均进行影像方案课程培训以及RAPID软件使用培训，并参加NCCT-ASPECTS的网络培训、模拟测验及考试。研究者通过项目组提供的在线培训系统（http://angel-aspect.org）进行NCCT-ASPECTS评分培训、练习及考核，考试合格（80分以上）后获得ASPECTS评估资格证书，具备入组筛选资格。在筛选受试者时，由经过培训的分中心研究者初筛，项目组经过培训的2位神经介入医师24h在线进行影像实时评估，确定是否符合入组标准，具体过程如下：

(1) ASPECTS判定：全部患者均进行非增强CT扫描，由经过培训的分中心研究者进行ASPECTS初筛后，及时通知项目组的2位神经介入医师进行实时在线评估。三者达成一致，确定ASPECTS符合入组标准3-5分时，符合入组。NCCT-ASPECTS需在RAPID ASPECTS® (version 5.0.4, iSchemaView, CA, USA)出具结果前由人工判定。

(2) 梗死体积判定：梗死核心体积由RAPID®系统(version 5.0.4, iSchemaView, CA, USA)自动判定，使用基于CTP的rCBF<30%或基于MRI的ADC<620判定梗死体积。如患者在发病6h内且符合ASPECTS 0-2，当梗死核心体积在70ml-100ml时符合入组标准。如果患者在发病6h-24h内，当梗死核心体积在70ml-100ml时，无论ASPECTS评分多少，均符合入组标准。

(3) 血管闭塞部位判定：通过CTA或MRA判定基线血管闭塞部位，闭塞部位符合ICA末端或大脑中动脉M1段时，符合本研究。CTA或MRA表现为同侧颈内动脉颅外段闭塞合并颅内段ICA或M1段闭塞的患者，同样符合本研究。

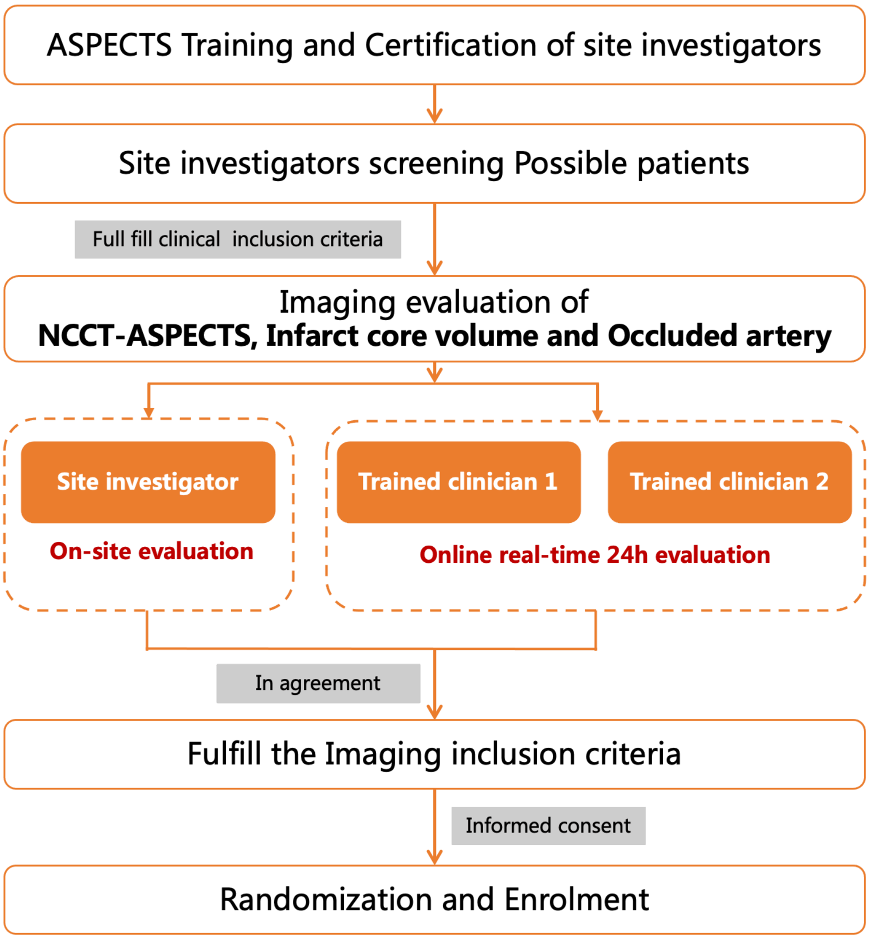

图2. 影像评估工作流程

5.2 术中及复查影像

(1) **术中影像**：EVT组患者，术前需要DSA确定血管闭塞部位、eTICI分级[29]，术后造影评估eTICI分级，并确定是否有术中并发症。建议术后即刻复查头CT或行平板CT排除出血。

(2) **出血的影像判定**：出血判定以48h内的平扫CT为主要判定标准，采用海德堡出血分型评价出血类型并记录。[30]

(3) **复查血管影像**：随机后36（±12）小时复查血管影像（CTA/MRA），明确血管通畅情况。[31]

(4) **随访梗死体积判定**：使用经过验证的自动化软件，以随机后7(±1)天/出院NCCT或36(±12)小时MRI评价随访梗死核心体积。[32]

5.3 核心影像实验室

天坛神经影像研究中心（Tiantan Neuroimaging Center of Excellence，T-NICE）为本研究的核心影像实验室。从病人发病后到出院期间，全部的影像资料（CT，CTA，CTP，MRI，MRA，PWI，DSA），由 CRO 收集 DICOM 格式原始文件后，由 T-NICE 质控、去隐私化后进行中心化判读。影像判读结果由影像判读委员会审核确认后，录入影像数据库系统。

六、 治疗方案

符合入排标准的患者在患者或合法代理人签署纸质版知情同意书之后进行随机化。随机到试验组的患者应在 BMM 的基础上接受 EVT 治疗，随机至穿刺时间应在 1 小时内。

6.1 血管内治疗（EVT）

患者情况允许时，首选局麻方式快速启动穿刺及血管内治疗，如病情需要，可使用清醒镇静方式，对于气道塌陷高危的患者可以考虑插管。如预计即使使用清醒镇静患者在术中配合也较差或由于患者的疾病情况使用清醒镇静剂高危或气道情况高危，应使用全身麻醉。术后根据患者情况，决定是否插管返回神经重症监护病房。

术前及术中不推荐全身肝素化治疗，动脉穿刺选择股动脉，可以使用长鞘、导引导管或球囊导引导管，推荐首选可回收支架取栓（建议选择 Solitaire、EMBOTRAP、Reco 或 Captor

等一线支架取栓系统）或接触抽吸取栓（建议选择 Penumbra 系统等）。若常规取栓操作后未实现成功再通，允许采用其他取栓技术、更换取栓器械、动脉内溶栓、球囊或支架成形术等方式进行补救治疗。需要进行补救治疗的情况规定为（包括但不限于，由研究者根据术中情况做出决策）：同一取栓器（支架或抽吸导管）进行 3 次取栓操作仍未成功再通；靶血管成功再通后再闭塞；靶血管夹层或狭窄程度≥70%，且合并任何程度的前向血流障碍；管腔/支架内新发血栓形成导致 eTICI 评级下降。

以上所有操作均应使用经国家药品监督管理局（National Medical Products Administration, NMPA）批准的器械，且应按照批准预期用途及操作说明使用。

6.2 最佳内科治疗（BMM）

所有入组患者将接受《中国卒中学会脑血管病临床管理指南》推荐的 BMM。[33] 符合静脉溶栓标准的患者应接受静脉溶栓治疗，计划进行或正在进行静脉溶栓的患者，成功入组后由研究者决定是否提前终止静脉溶栓。在随机化前已经完成静脉溶栓的患者同样可以纳入本研究。所有接受静脉溶栓的患者需详细记录静脉溶栓药物名称、实际使用剂量和用药时间。不建议在静脉溶栓后 24 小时内使用抗血小板药物，除非患者进行了球囊扩张成形或支架置入手术，此时由研究者决定抗栓策略。基于 ANGEL-ASPECT 对时间窗和梗死核心体积的规定，预计部分入组患者未接受静脉溶栓治疗。对于这部分患者，除非存在早期抗凝治疗指征，应早期接受阿司匹林治疗。

七、 评价指标

7.1 主要有效性评价指标

随机化 90（±7）天 mRS

7.2 次要有效性评价指标

(1) 随机化 90（±7）天 mRS 0-2 比例

(2) 随机化 90（±7）天 mRS 0-3 比例

(3) 随机化 36（±12）小时 NIHSS 0-1 分或较基线减少≥10 分比例

(4) 随机化 7（±1）天/出院 NCCT 或 36（±12）小时 MRI 评价的梗死体积较基线的变化

(5) 随机化 36（±12）小时 CTA 或 MRA 判定的闭塞血管再通率

7.3 主要安全性评价指标

随机化 48 小时内发生 sICH 的概率（海德堡出血分型，附录 5）

7.4 次要安全性评价指标

(1) 随机化 90（±7）天全因死亡率

(2) 随机化 48 小时内任何类型的颅内出血（海德堡出血分型）的概率

(3) 住院期间实行去骨瓣减压术的概率

八、 数据采集和研究流程

研究者应完成包括所有被筛选对象在内的一份筛选表，需注明分中心编号。筛选表将用于分析判断不同分中心的入组患者是否具有代表性。研究者应遵照研究方案和填写手册将数据及时填写到病例报告表（Case Report Form，CRF）中，并确保按照临床研究协议的要求准确、完整、及时地记录数据和回复数据质疑。所有患者脑部影像检查数据采用 DICOM 格式收集，各序列信息完整，包括：CT, CTA, CTP, MRI（T1+T2+DWI+FLAIR+ADC+GRE-T2*/SWI+MRA±PWI）和 DSA。实验室化验检查等采用原始报告拍照上传。

8.1 筛选和入组

- 基本信息：中心编号，患者编号，年龄，性别，是否造影剂过敏。
- 现病史：起病（最后正常）时间，到达急诊时间，起病形式，起病后静脉溶栓情况（开始时间，药物名称和剂量）。
- 既往病史和发病前合并用药

  既往病史（吸烟、饮酒、高血压、糖尿病、高脂血症、心律失常、心脏瓣膜病、心功能不全、冠心病、外周血管病、短暂性脑缺血发作、脑梗死、脑出血、颅内肿瘤），起病前 mRS;

  合并用药：抗血小板药（阿司匹林、氯吡格雷、西洛他唑、替格瑞洛等），抗凝药（华法林、达比加群、利伐沙班等），他汀类（阿托伐他汀、匹伐他汀、辛伐他汀等）。

- 体格检查

身高，体重，血压，脉搏，神经功能评估（NIHSS 和 Glasgow 评分），12 导联心电图。

- 急诊实验室检查

急诊血常规，肾功能，肝功能，凝血项，随机血糖等。

- 影像检查

需完善头 CT 检查排除出血性疾病，并依此进行 ASPECT 的评估，完善 CTA/MRA 确认 LVO；完善 CTP/MRI 检查计算梗死体积。

- 所有受试者或其合法代理人需签署知情同意书。
- 对纳入的受试者进行随机化。

8.2 术中数据收集

- 麻醉方式：插管的全身麻醉，伴或不伴镇静的局部麻醉。
- 术中时间点：动脉穿刺时间，每次操作结束时间，首次再通时间，成功再通时间或手术结束时间。
- 术前和术后 eTICI 评分
- 手术细节：辅助器械（导引导管，导丝，中间导管，微导管），取栓操作次数，开通操作次数，除取栓外的补救措施。
- 术中用药：肝素，替罗非班，阿替普酶，尿激酶等。
- 术中并发症：血管痉挛 (出现时间、累及血管、解除时间、治疗方案)，血栓移动或栓塞，夹层，穿孔等。

8.3 住院期间访视

需在随机化后 36 (±12)小时和 7 (±1)天或出院时（以先发生时间为准）对受试者进行面对面访视。

- 随机化后 36 (±12)小时完善 CT/CTA 或 MRI/MRA 检查。
- 随机化后 36 (±12)小时和 7 (±1)天或出院时（以提前发生时间为准）需进行体格检查和神经功能评估。需收集的数据包括：生命体征（血压和心率），相关用药（降压药，抗血小板药、抗凝药），临床评估和提个检查的重要发现（如所有新发、恶化或改善的情况），重要的神经系统发现，NIHSS 评分（两次均需评估），mRS（仅 7 天或出院时需评估）和不良事件（Adverse event，AE）。
- 7 (±1)天或出院时（以提前发生时间为准）需进行平扫 CT 评估。
- 随机化后 36 (±12)小时和 7 (±1)天或出院时（以提前发生时间为准）需进行实验室检查，包括：血常规、肾功能、肝功能、凝血项、空腹血糖等。

8.4 随机化后第 30 (±3)天，90 (±7)天和 1 年(±14 天)随访

当受试者无法达到研究中心时，这些访视可以使用电话访视。所有受试者的访视均由受过标准神经功能评估训练的有经验的研究者，在对患者接受治疗方式未知的情况下进行。需收集的数据包括:

- mRS
- 体现患者健康状态和生活质量的欧洲五维健康量表（EuroQoL 5-Dimensions 5-Level questionnaire，EQ-5D-5L）[34]
- 相关合并用药

- 临床评估和体检的发现(即出院后所有新的、恶化的或改善的情况)

8.5 非计划访视

如果研究期间进行了非计划访视，将评估任何新的或未解决的 AE。如果该访视基于神经功能变化的事件而发生，需评估 NIHSS 和 mRS。

8.6 研究流程表

措施	随机化当天	随机化36(±12)小时	随机化7（±1）天/出院	随机化30(±3)天	随机化90(±7)天	随机化12 个月±14天
签署知情同意书	x					
核实入组/排除标准	x					
随机化	x					
人口统计学特征	x					
现病史	x					
既往病史	x					
药物治疗	x	x	x	x	x	x
mRS	x		x	x	x	x
NIHSS	x	x	x			
头颅 CT	x		x[3]			
头颅 CTA±CTP 或 MRI*+MRA±PWI	x[1]					
头颅 CT+CTA 或 MRI*+SWI+MRA		x[2]				
颈动脉 CTA/MRA/超声		x[4]				
ASPECTS	x					
梗死体积	x	x[5]	x[5]			
实验室检查	x	x	x			
心电图	x					
TOAST			x			
EQ-5D-5L 量表				x	x	x
AE/SAE		x	x	x	x	x

[1]所有入组病例，随机前首选NCCT+CTA+CTP检查

[2]所有入组病例，应在随机后24-48小时复查多模影像，影像评估方式建议同随机化前一致

[3]所有入组病例，应在随机后7±1天或出院（以先发生时间为准）时进行头颅CT检查

[4]仅适用于BMM组且随机化前未做颈部血管影像检查的患者

[5]根据头颅CT或MRI，由核心影像实验室判定

*MRI序列包括T1+T2+DWI+ADC+FLAIR序列

ADC: apparent diffusion coefficient; AE: adverse event; ASPECTS: Alberta stroke program early computed tomography score; CT: computed tomography; CTA: computed tomography angiography; CTP: computed tomography perfusion; EQ-5D-5L: EuroQoL 5-Dimensions 5-Level questionnaire; FLAIR: fluid attenuated inversion recovery; MRA: magnetic resonance angiography; MRI: magnetic resonance imaging; mRS: modified Rankin scale; NIHSS: National Institutes of Health Stroke Scale; PWI: perfusion weighted imaging; SAE: serious adverse event; SWI: susceptibility weighting imaging; TOAST: Trial of ORG 10172 in Acute Stroke Treatment.

九、 项目风险的预评估及风险处置

9.1 不良事件监测

所有 AEs 都将按照所适用法规进行管理和报告，并将包含在最终的临床研究报告 (Clinical Study Report, CSR) 中。

9.2 不良事件定义

9.2.1 不良事件

受试者在研究期间出现的不良医学事件或原有医学事件恶化，不论是否与研究治疗有因果关系。AE 可以为临床症状（例如：恶心，胸痛等），体征（例如：心动过速，肝脏肿大等）或异常的检查结果（例如：实验室检查，心电图等）。AE 包括发生在整个临床研究的任何时间段的不良医学事件，包括导入期或洗脱期，甚至尚未给予患者研究治疗。AE 包括严重 AE（Serious adverse event，SAE）和非严重 AE。

9.2.2 严重不良事件

是研究期间出现满足以下一项或以上标准的 AE：

- 导致死亡
- 即刻威胁生命

  备注：危及生命，其严重程度的定义是指事件发生当时即危及患者生命，而并非事件如果更严重时将导致患者死亡。

- 需要住院治疗或是延长此次住院时间

- 导致终生或严重残障/机能不全，或丧失维持正常生活功能的重要能力
- 先天性畸形，先天性缺陷，出生缺陷或生育障碍
- 重大的医学事件，可能危害受试者，或需要医疗干预以避免以上的结果出现。

9.3 不良事件的记录

非严重 AE：从入组开始直至研究结束这一期间内关注的非严重 AE。其它非严重 AE 由研究者决定是否记录。

SAE：所有的 SAE 均需要记录。

9.4 不良事件与试验的关系评定

按 5 级标准评定：(1)肯定有关；(2)很可能有关；(3)可能有关；(4)可能无关；(5)肯定无关；(6)无法判断。

9.5 研究者在安全性报告方面的职责

9.5.1 不良事件

从入组第一次访视或签署知情同意书（如:出现在洗脱期）开始，到研究方案最后一次随访结束为止，患者出现的任何 AE，无论其严重程度，是否与研究相关，都应记录在 CRF 相关页面中。在任何可能的情况下，患者出现的症状都应被分组为某个单独的综合征或临床诊断。研究者应具体描述事件发生的时间、事件强度、采取的相应措施、给予的对症治疗、结局以及他/她关于该 AE 是否有可能由研究导致的判断。

9.5.2 严重不良事件

对于 SAE，研究者必须立即采取相应处理措施：

立即通知申办方的相应负责人，将 CRF 中已签名和标注日期的相应页发送给申办方的相应负责人，附加所有检查的复印件和这些检查完成的日期。同时必须保护患者隐私，确保在提供给申办方的临床原始资料复印件中去除患者身份信息。实验室结果应包括正常范围值。

对任何 SAE 的上报应在发生后**24 小时内**完成。并将 SAE 填写在 CRF 相应位置，签字后最好以传真的方式报告给安全委员会，安全委员会的名称，地址，传真号将在临床试验方案中注明。

9.5.3 风险处置

研究者应采取所有合理的措施来确保患者的安全。

严格按照研究入选及排除标准筛选受试者。在研究过程中出现 AE，必要时可暂停或者终止用药，酌情进行血常规、凝血象、肌酶、肝功能、肾功能、血气分析、超声、CT 等相关辅助检查，积极给予对症处理，必要时请相关专科医生进行会诊。对于 SAE，应首先确保气道通畅、自主呼吸、血压及心率稳定。

对于所有 AE，研究者应继续对患者进行随访，追踪任何与之相关的结果（临床症状、体征、实验室检查结果等），直到相关临床症状完全缓解、实验结果恢复或病情稳定后。患者退出临床试验后仍将继续随访，按照正常访视时间点对患者进行电话或面对面访视。监查团队有可能要求额外研究。在研究终止后，研究者仍应注意任何出现的 SAE。

十、 统计学处理

10.1 样本量计算

本试验采用多中心、开放性、随机、平行对照的试验方法。主要有效性指标为入组后90±7 天的 mRS 评分（视为有序多分类变量）。根据文献数据及临床专家意见，设定参数如下：①对照组患者 mRS 评分 0-6 分的比例分别为 3%、4%、10%、17%、16%、12%和 38%；②EVT 的平均治疗效果会提升试验组患者 mRS 评分下降的几率，使得 common OR 值达到1.73；③考虑进行两次期中分析，校正检验水准 α=0.046（双侧），把握度 1-β=0.90；④试验组与对照组按照 1:1 比例分配样本量。计算得到样本量为 452，考虑 10%的脱落率，最终总样本量为 502 例，每组 251 例。

当完成 1/3（168 例）、2/3（336 例）受试者 90 天随访时将进行两次期中分析。多次检验将使用 O'Brien-Fleming 消耗函数法对检验水准进行调整，对应的检验水准 α 分别为双侧0.0002（第 1 次期中分析）、0.0123（第 2 次期中分析）、0.046（终末分析）。

样本量计算使用PASS 11 (NCSS，LLC)进行。

10.2 数据采集与录入

本研究采用纸质 CRF 和电子数据捕获（Electronic Data Capture，EDC）系统进行数据采集和录入，系统中所有试验方案要求的内容都必须提供，未填写内容应予以解释，需要在EDC 系统每一个表单下备注处填写原因。

10.2.1 研究者填写纸质 CRF

分中心研究者必须使用黑色或蓝黑色记录笔工整清晰填写纸质 CRF，以确保数据清晰

可读。如果纸质 CRF 信息需要修改，不应进行涂改或覆写，正确的信息应写在原始信息旁边，并由修改人签名及注明日期。临床监查员（Clinical Research Associate, CRA）将复核 CRF 的完整性和准确性，并指导研究者进行必要的质疑更正与补充。

10.2.2 研究协调员（Clinical research coordinator, CRC）录入 EDC 系统

纸质 CRF 填写完成后，由 CRC 根据纸质 CRF 填写内容录入至 EDC 系统中。

10.2.3 研究者审核通过后提交 EDC 系统表单

最后由填写纸质 CRF 研究者审核通过后提交，数据提交后，所有数据的修改和反馈均通过 EDC 系统进行。若 EDC 系统已提交表单需要修改，需要联系本中心 CRA，由 CRA 开放表单后，研究者可指导 CRC 进行 EDC 系统数据修改。

10.2.4 CRA 通过 EDC 系统进行数据质询

10.2.5 EDC 系统数据导出数据库

EDC 系统数据导出数据库后，将由数据管理员进行校对，明显的错误将由数据管理员更正，其它错误或漏填项目将填入数据质疑表中通过 email，快递，电话以及微信形式返还给分中心解决。

研究中心查证原始资料及相关信息后在 EDC 系统更改数据。研究者必须通过证实或修改数据的方式来回答这些需求。

10.3　统计分析

这部分是统计学分析的概述。它对如何收集数据和在临床研究报告中陈列数据进行了总体规定。数据库锁定前将完成最终版统计分析计划书（Statistical Analysis Plan，SAP）。SAP将规定所有“预先规定的、计划进行的分析”。

数据统计分析使用SAS 9.4软件完成。

10.3.1 统计分析数据集

（1）全分析集（Full Analysis Set, FAS）

根据意向性分析（Intention-To-Treatt，ITT）的基本原则，将所有随机化入组、并接受了内科治疗或EVT的受试者纳入FAS。FAS是本研究的主要有效性评价人群。

（2）符合方案集（Per Protocol Set, PPS）

PPS包括所有完成方案规定的治疗或没有严重违反试验方案的受试者。严重违反方案的确切定义将在数据审核时最终确定，一般可能包括以下几种情况（但不限于这些情况）:

1)不符合主要入选标准；

2)入选后存在严重干扰疗效评价的治疗；

3)依从性差；

4)随访严重超出时间窗。

PPS是有效性的次要分析人群，但其结果如与FAS不一致，需对不一致结果进行详细分析。

（3）安全性数据集（Safety Analysis Set, SAS）

SAS定义为所有参与试验、接受研究治疗、并且至少有一次安全性评价的受试者。

10.3.2 统计分析方法

（1）基线指标的组间比较

两组计量资料采用t检验或Wilcoxon秩和检验；两组计数资料的比较采用卡方检验、Fisher's精确概率法或Wilcoxon秩和检验。

（2）有效性分析

主要终点：根据 ITT 的基本原则，对于 FAS 数据采用有序 logistic 回归评估疗效，计算共同比值比(common OR)。同时，采用 PPS 数据集作为敏感性分析进行同样的统计分析。所有统计数据都将采用双侧检验，P<0.046 被认为有统计学意义。

次要终点：90 天 mRS 0-2 比例等多数二分类变量次要结局分析将采用 logistic 回归进行分析。梗死核心体积较基线的变化采用 t 检验或 Wilcoxon 秩和检验分析两组差异。

（3）安全性分析

基于 SAS 数据集，采用统计描述两组安全性事件的发生率。采用 logistic 回归比较两组间颅内出血事件等安全性终点的差异。采用卡方检验和 Fisher's 精确概率法等方法比较两组各种不良反应与 SAE 发生率的差异。

（4）亚组分析

既往研究认为以下因素可能影响受试者 90 天 mRS，本研究将根据以下变量进行亚组分析：

(1) 年龄（＜70 岁 vs. ≥70 岁）；

(2) 是否醒后卒中；

(3) 最后正常时间至随机化时间（＜6h vs. ≥ 6h）；

(4) 随机化前卒中严重程度（NIHSS<16 vs. NIHSS≥16）；

(5) 是否接受静脉溶栓；

(6) 血管闭塞部位（ICA 颅内段 vs. M1 段）；

(7) 是否合并同侧颈动脉闭塞；

(8) ASEPECT 评分（＜3 分 vs. ≥3 分）；

(9) 梗死体积（＜70ml vs. ≥70ml）；

(10) 卒中亚型(心源性栓塞型 vs. 大动脉粥样硬化型）。

10.4 期中分析

期中分析将分别在完成首要终点访视的受试者数量达到总样本量的 1/3（168 例）和 2/3（336 例）时进行。多次检验将使用 O'Brien-Fleming 消耗函数法对检验水准进行调整，对应的检验水准 α 分别为双侧 0.0002（第 1 次期中分析）、0.0123（第 2 次期中分析）、0.046（终末分析）。

通过期中分析，数据安全监查委员会（Data Safety Monitoring Board，DSMB）将决定研究继续或终止。针对研究失败的评估，根据现有数据并结合预期最终样本量，如本研究预期无法得出有效性结果，将在当期即时终止研究。针对研究有效的评估，如研究已经达到有效性标准，并符合检验水准的要求，可提前结束研究；如评估疗效预期在研究结束时可达成，则继续进行研究。如果研究中对主要结局的估计值与实际相差较大，则最终样本量允许在期中分析时进行适当的调整。

十一、 伦理原则

11.1 伦理原则

本临床研究将遵循世界医学大会《赫尔辛基宣言》等相关规定。所有应用修正案将在第

18 届世界医学宣言和国际人用药品注册技术协调会（The International Council for Harmonisation of Technical Requirements for Pharmaceutical for Human Use，ICH）指南中的临床试验管理规范指导下展开。在研究开始之前，由伦理委员会批准该试验方案后才实施临床研究。每一位受试者入选本研究前，研究者有责任向受试者或其代理人完整、全面地介绍本研究的目的、程序和可能的风险，并签署书面知情同意书，应让受试者知道他们有权随时退出本研究，知情同意中应作为临床研究文件保留备查。研究过程中将保护受试者的个人隐私与数据机密性。

11.2 法律法规

本临床试验管理将遵循国际法律法规，并遵循试验所在地中国的法律法规以及任何有关应用的指导方针。

11.3 知情同意书

研究者(根据相应法规的要求)或由研究者授权并负责的人员，应该充分告知受试者包括伦理委员会许可的书面信息在内的临床试验的所有相关问题。在递呈给伦理委员会审核之前，研究者所使用的知情同意书应由资助方审阅并批准。

研究者应该用受试者可以理解的语言或术语最大程度的告知其有关研究的信息。在参与临床试验之前，患者本人或其法定代表应与患者讨论知情同意的研究人员共同签署知情同意书（姓名及日期）。研究者应向患者提供已签署的知情同意书的副本。

11.4 审查机构/独立的伦理委员会(IRB/IEC)

在试验开始前，研究者或申办方必须向伦理委员会递交临床试验方案，研究者须向申办

方递交伦理委员会签署的书面同意意见的复印件。

临床试验（研究编号、研究方案名称、版本号），审阅过的文件（包括临床研究方案、知情同意书、研究者操作手册、研究者简历等），投票成员的资格认定及审阅日期等都应清楚地记录在伦理委员会同意书上。

在临床试验过程中，对所有试验方案的任何修改应向伦理委员会报告，经批准后方能执行。在试验中发生的任何可能影响患者安全或临床试验继续展开的事件，尤其是安全性的改变均应向伦理委员会报告。研究者操作手册的更新应递交给伦理委员会。如果需要，每年应向伦理委员会递交临床试验的进展报告和临床试验结束后的临床结果摘要。

十二、 资料保密与结果公布

主要研究者拥有完整的知识产权。整个研究过程及数据分析过程均严格保护受试者信息，执行委员会将按照管理条例和程序来公布该试验的结果。数据库锁定后试验结果将尽快被公布。本试验将对其治疗效果、医疗措施及临床结局详细地进行资料分析。参与本研究的生物统计学专家有权访问数据集但不可能识别出此试验的任何患者。最后，以逗号分隔的文本格式的磁盘存储数据（包括文本格式的数据字典）将送至有合作意向的第三方机构处理。

十三、 研究组织

13.1 研究组织构成

- **筹划指导委员会成员**

✓ 筹划指导委员会将对试验进行科学性和战略性的指导，并且对试验的设计、执行和发

表全权负责。

- ✓ 筹划指导委员会将确保研究质量、研究的执行和管理。
- ✓ 筹划指导委员会将在研究开始前批准研究方案和操作指南。
- ✓ 筹划指导委员会将定期召开电话会议或面对面会议以讨论和汇报研究的进展。
- ✓ 筹划指导委员会的组成及其职责在执照中描述，其最终形式将在试验开始前确定。

筹划指导委员会

成员	科室	单位
王拥军	神经内科	首都医科大学附属北京天坛医院
王伊龙	神经内科	首都医科大学附属北京天坛医院
刘丽萍	神经内科	首都医科大学附属北京天坛医院
David S. Liebeskind	神经内科	洛杉矶加州大学
缪主任	介入神经病学	首都医科大学附属北京天坛医院
任泽光	神经外科	贵阳医科大学附属医院
Vitor Mendes Pereira	神经外科	多伦多大学圣迈克尔医院

- **执行委员会**

评审试验进展情况及收集可用的数据，并对研究进行适当的指导。主要决定将由执行委员会组织面对面会议决策。执行委员会的组成职责及其职责在执照中描述，其最终形式将在试验开始前确定。

- **数据安全监查委员会（DSMB）**

DSMB 将定期监督本研究的进展，以确保该研究达到道德规范和患者安全的最高标准。它由学术成员组成，包括独立的统计学家，他们不单独参与试验。DSMB 资格将在试验开始前经由 DSMB 和执行委员会证明，包括会员资格、角色、职责。

每一次 DSMB 会议后，报告将立即上交给委员会主席。

数据安全监测委员会

成员	科室	中心
刘建民	神经外科	海军军医大学第一附属医院
姚晨	医疗统计学系	北京大学第一医院
陈康宁	神经内科	陆军医科大学西南医院

- **临床事件委员会（CEC)**

临床事件以及安全终点将由临床事件委员会进行复审。临床事件委员会章程应在试验开始之前经由事件仲裁委员会和执行委员会认证。

临床事件委员会

成员	科室	中心
方堃	神经内科	复旦大学华山医院
宋波	神经内科	郑州医科大学附属第一医院
董漪	神经内科	复旦大学华山医院

- **影像判读委员会**

成员	科室	中心
荆京	天坛神经影像研究中心	中国神经系统疾病临床研究中心
张喆	天坛神经影像研究中心	中国神经系统疾病临床研究中心
张英魁	天坛神经影像研究中心	中国神经系统疾病临床研究中心
吴伟	神经内科	山东大学齐鲁医院

13.2 中心培训，认证

执行委员会应确保所有分中心都接受了药物临床试验质量管理规范（Good Clinical Practice，GCP）培训，同时应对所有分中心进行患者筛选、随访以及结局评价的培训（例如: NIHSS 评分,mRS 评分等）。在研究启动之前，分中心主要研究者和协调员按照要求完成培训。

所有的研究者必须完成以下的培训：

(1) 研究流程

(2) ANGEL-ASPECT 入组与排除标准

(3) mRS 评分

(4) NIHSS 评分

(5) ASPECTS

(6) RAPID 软件使用

(7) eTICI 分级

(8) TOAST 病因分型

(9) 海德堡出血分型

(10) 影像学资料收集（DICOM 格式）

每个中心在招募患者之前必须要完成项目培训。研究中心 PI 和主要研究成员将定期参加电话会议以解决研究中遇到的问题。

详尽的研究流程手册作为研究者的主要参考文档。它为研究中心的研究者提供培训指导，并在整个研究期间定期地根据需要在研究网站上进行更新。执行委员会成员与研究协调员将以电话，微信或 e-mail 的形式与中心研究者保持沟通，解决研究中遇到的问题。执行委员会与监督委员会一起回答各分中心遇到或提出的疑问，并将这些疑问和答案整理后分发给所有分中心。

执行委员会成员将管理并实地考察分中心，以确保记录在 CRF 中的数据的完整性与有效性。在整个试验过程中每个研究中心至少被考察一次，如果研究数据质量有疑问或受试者入组有问题会根据需要增加考察次数。

十四、 研究的质量控制与质量保证

14.1 研究者职责

研究者保证在进行临床试验时遵循临床试验方案，遵循 ICH 指南中的临床试验管理规范以及相应法律法规。

研究者应保证遵循临床试验方案中的所有研究操作（包括安全性原则）。研究者应根据相关要求，以一种准确、清晰的方式正确（CRF、偏差解析表及其它方式）提供可靠数据和

临床试验方案要求的所有信息，并确保监查团队可直接查看原始资料。

研究者可能会任命他/她认为合适的人作为协助研究者。协助研究者将根据临床试验方案协助进行临床试验的管理。所有的协助研究者将被及时任命并记录。协助研究者接受研究者的监督和管理。研究者将提供给他们一份临床试验方案和所有必需的信息。临床试验的申办方通过采取所有适当的手段来确保临床试验的合理管理，这些手段如伦理、临床试验方案依从性、记录在 CRF 中数据的完整性和有效性等。

14.2 研究监查

监查团队的主要责任是帮助研究者保证临床试验各个方面的高度的伦理性、科学性、专业性和规范性。根据 ICH 指南中的 GCP 原则，监督团队应对照原始资料对 CRF 进行核查。

监查团队将定期通过现场访视或线上视频会议形式与每个中心联系，将派出代表来评估研究进展、研究者和患者对临床方案的依从性以及解决紧急的问题。在这些监查访问中，监查员将和研究者共同监查，要点如下（并非无遗漏）: 患者的知情同意、SAE 文件的记录和报告，以及数据的质量。

十五、 资料保存

被复查后的 CRF、影像资料由研究监查员送到试验指定的数据管理中心。由数据管理中心负责人员进行核对签收。数据管理中心应对收到的 CRF 进行认真的录入处理并妥善保存。在数据录入完毕后 CRF 由研究中心保存。

十六、 数据安全监查

制定相应的数据安全监察计划，所有 AE 均详细记录，恰当处理并追踪直到妥善解决或病情稳定，按照规定及时向伦理委员会、主管部门、申办者和监督管理部门报告 SAE 与非预期事件等；主要研究者定期对所有 AE 进行累积性回顾，必要时召开研究者会议评估研究的风险与受益；本研究将建立独立的数据安全监察委员会对累积的安全性数据以及有效性数据进行监查，以做出研究是否继续进行的建议。

临床试验过程中，受试者的数据应以匿名方式收集在 CRF 上，受试者只有通过受试者编号和姓名拼音缩写来辨认。由于安全或行政指令原因，例外知道受试者身份时，和研究者应共同承担保密责任。知情同意书允许患者同意已授权的申办方、伦理委员会、权威机构直接查阅 CRF 上相关的原始资料(如患者的医疗档案、预约记录、原始实验室记录等)。上述人员应遵循职业保密规定，必须对患者的所有个人身份信息或医疗信息保密。

十七、 研究注册及文章发表

17.1 研究总结和结果的登记

研究代表在研究开始前在 ClinicalTrials.gov (https: // ClinicalTrials.gov)上注册一份研究摘要，并根据研究方案的变化或研究进展适当更新该摘要。研究结束后，研究代表立即登记研究结果。

17.2 研究结果的发表

研究结束后，研究数据库将在最后一个纳入患者的最后一次预定随访日期后一个月内关

闭。研究代表在采取必要措施(如防止特定研究患者的识别)以保护患者和相关方的人权或患者和相关方的权利和利益。描述该研究和主要研究问题结局的投稿将在数据库关闭后 3 个月内投稿到主要临床杂志，以公布研究结果。

投稿前一个月将与经济资助人共享稿件，但经济资助人不影响稿件内容。论文作者由研究代表根据国际医学期刊编辑委员会(International Committee of Medical Journal Editors, ICMJE)提交生物医学期刊稿件统一要求(http: //www.icmje.org/)确定。所有作者应在提交前审查并同意论文的细节。会议报告的作者也是如此。

十八、 数据的所有权和使用

18.1 研究结果的所有权

本研究获得的结果、数据、知识产权等均属于研究者，不属于患者。研究者的知识产权属于个人还是研究机构，由参与医院协议决定。

18.2 收集数据的使用

研究指导委员会决定是否将本研究中获得的数据(以下简称“研究数据”) 作为本研究的二次分析，用于研究代表或次级研究者进行的研究。

如果分析被判断为超出二次分析的范围，或者研究数据被除本研究的研究代表或次级研究人员以外的其他人使用，研究指导委员会将根据人类受试者医学研究的相关法律法规和伦理指南，准备单独的协议，并在经过伦理审查后进行研究。

十九、 项目赞助及利益冲突

本研究经费由柯惠医疗器材国际贸易（上海）有限公司（“柯惠”），强生医疗有限公司，健适医疗有限公司，上海心玮医疗科技有限公司赞助。在试验设计、实施、统计分析和报告中,不存在可能影响试验结果或本方案编制时的利益冲突。各组的治疗方案不损害参与本试验患者的权利和利益。

二十、 参考文献

1. Warner JJ, Harrington RA, Sacco RL, Elkind MSV. Guidelines for the Early Management of Patients With Acute Ischemic Stroke: 2019 Update to the 2018 Guidelines for the Early Management of Acute Ischemic Stroke. Stroke 2019;50(12):3331-3332. DOI: 10.1161/STROKEAHA.119.027708.
2. Turc G, Bhogal P, Fischer U, et al. European Stroke Organisation (ESO)- European Society for Minimally Invasive Neurological Therapy (ESMINT) guidelines on mechanical thrombectomy in acute ischemic stroke. J Neurointerv Surg 2019;11(6):535-538. DOI: 10.1136/neurintsurg-2018-014568.
3. Berkhemer OA, Fransen PS, Beumer D, et al. A randomized trial of intraarterial treatment for acute ischemic stroke. N Engl J Med 2015;372(1):11-20. DOI: 10.1056/NEJMoa1411587.
4. Campbell BC, Mitchell PJ, Kleinig TJ, et al. Endovascular therapy for ischemic stroke with perfusion-imaging selection. N Engl J Med 2015;372(11):1009-18. DOI: 10.1056/NEJMoa1414792.
5. Goyal M, Demchuk AM, Menon BK, et al. Randomized assessment of rapid endovascular treatment of ischemic stroke. N Engl J Med 2015;372(11):1019-30. DOI: 10.1056/NEJMoa1414905.
6. Jovin TG, Chamorro A, Cobo E, et al. Thrombectomy within 8 hours after symptom onset in ischemic stroke. N Engl J Med 2015;372(24):2296-306. DOI: 10.1056/NEJMoa1503780.
7. Saver JL, Goyal M, Bonafe A, et al. Stent-retriever thrombectomy after intravenous t-PA vs. t-PA alone in stroke. N Engl J Med 2015;372(24):2285-95. DOI: 10.1056/NEJMoa1415061.
8. Albers GW, Marks MP, Kemp S, et al. Thrombectomy for Stroke at 6 to 16 Hours with Selection by Perfusion Imaging. N Engl J Med 2018;378(8):708-718. DOI: 10.1056/NEJMoa1713973.
9. Nogueira RG, Jadhav AP, Haussen DC, et al. Thrombectomy 6 to 24 Hours after Stroke with a Mismatch between Deficit and Infarct. N Engl J Med 2018;378(1):11-21. DOI: 10.1056/NEJMoa1706442.
10. Gautheron V, Xie Y, Tisserand M, et al. Outcome After Reperfusion Therapies in Patients With Large Baseline Diffusion-Weighted Imaging Stroke Lesions: A THRACE Trial (Mechanical

Thrombectomy After Intravenous Alteplase Versus Alteplase Alone After Stroke) Subgroup Analysis. Stroke 2018;49(3):750-753. DOI: 10.1161/STROKEAHA.117.020244.
11. Bracard S, Ducrocq X, Mas JL, et al. Mechanical thrombectomy after intravenous alteplase versus alteplase alone after stroke (THRACE): a randomised controlled trial. Lancet Neurol 2016;15(11):1138-47. DOI: 10.1016/S1474-4422(16)30177-6.
12. Deb-Chatterji M, Pinnschmidt H, Flottmann F, et al. Predictors of independent outcome of thrombectomy in stroke patients with large baseline infarcts in clinical practice: a multicenter analysis. J Neurointerv Surg 2020;12(11):1064-1068. DOI: 10.1136/neurintsurg-2019-015641.
13. Roman LS, Menon BK, Blasco J, et al. Imaging features and safety and efficacy of endovascular stroke treatment: a meta-analysis of individual patient-level data. Lancet Neurol 2018;17(10):895-904. DOI: 10.1016/S1474-4422(18)30242-4.
14. Campbell BCV, Majoie C, Albers GW, et al. Penumbral imaging and functional outcome in patients with anterior circulation ischaemic stroke treated with endovascular thrombectomy versus medical therapy: a meta-analysis of individual patient-level data. Lancet Neurol 2019;18(1):46-55. DOI: 10.1016/S1474-4422(18)30314-4.
15. Sarraj A, Hassan AE, Savitz S, et al. Outcomes of Endovascular Thrombectomy vs Medical Management Alone in Patients With Large Ischemic Cores: A Secondary Analysis of the Optimizing Patient's Selection for Endovascular Treatment in Acute Ischemic Stroke (SELECT) Study. JAMA Neurol 2019;76(10):1147-1156. DOI: 10.1001/jamaneurol.2019.2109.
16. Cagnazzo F, Derraz I, Dargazanli C, et al. Mechanical thrombectomy in patients with acute ischemic stroke and ASPECTS </=6: a meta-analysis. J Neurointerv Surg 2020;12(4):350-355. DOI: 10.1136/neurintsurg-2019-015237.
17. Sarraj A, Grotta JC, Pujara DK, Shaker F, Tsivgoulis G. Triage imaging and outcome measures for large core stroke thrombectomy - a systematic review and meta-analysis. J Neurointerv Surg 2020;12(12):1172-1179. DOI: 10.1136/neurintsurg-2019-015509.
18. Rebello LC, Bouslama M, Haussen DC, et al. Endovascular Treatment for Patients With Acute Stroke Who Have a Large Ischemic Core and Large Mismatch Imaging Profile. JAMA Neurol 2017;74(1):34-40. DOI: 10.1001/jamaneurol.2016.3954.
19. Broocks G, Flottmann F, Schonfeld M, et al. Incomplete or failed thrombectomy in acute stroke patients with Alberta Stroke Program Early Computed Tomography Score 0-5 - how harmful is trying? European journal of neurology 2020;27(10):2031-2035. DOI: 10.1111/ene.14358.
20. Nicholson P, Hilditch CA, Neuhaus A, et al. Per-region interobserver agreement of Alberta Stroke Program Early CT Scores (ASPECTS). J Neurointerv Surg 2020;12(11):1069-1071. DOI: 10.1136/neurintsurg-2019-015473.
21. van Horn N, Kniep H, Broocks G, et al. ASPECTS Interobserver Agreement of 100 Investigators from the TENSION Study. Clin Neuroradiol 2021. DOI: 10.1007/s00062-020-00988-x.
22. Demeestere J, Garcia-Esperon C, Garcia-Bermejo P, et al. Evaluation of hyperacute infarct volume using ASPECTS and brain CT perfusion core volume. Neurology 2017;88(24):2248-2253. DOI: 10.1212/WNL.0000000000004028.
23. Sarraj A, Hassan AE, Grotta J, et al. Optimizing Patient Selection for Endovascular Treatment in Acute Ischemic Stroke (SELECT): A Prospective, Multicenter Cohort Study of Imaging Selection. Ann Neurol 2020;87(3):419-433. DOI: 10.1002/ana.25669.
24. Mourand I, Abergel E, Mantilla D, et al. Favorable revascularization therapy in patients with

ASPECTS </= 5 on DWI in anterior circulation stroke. J Neurointerv Surg 2018;10(1):5-9. DOI: 10.1136/neurintsurg-2017-013358.

25. Inoue M, Olivot JM, Labreuche J, et al. Impact of diffusion-weighted imaging Alberta stroke program early computed tomography score on the success of endovascular reperfusion therapy. Stroke 2014;45(7):1992-8. DOI: 10.1161/STROKEAHA.114.005084.
26. Han M, Choi JW, Rim NJ, et al. Cerebral infarct volume measurements to improve patient selection for endovascular treatment. Medicine (Baltimore) 2016;95(35):e4702. DOI: 10.1097/MD.0000000000004702.
27. Manceau PF, Soize S, Gawlitza M, et al. Is there a benefit of mechanical thrombectomy in patients with large stroke (DWI-ASPECTS </= 5)? European journal of neurology 2018;25(1):105-110. DOI: 10.1111/ene.13460.
28. Broocks G, Rajput F, Hanning U, et al. Highest Lesion Growth Rates in Patients With Hyperacute Stroke. Stroke 2018:STROKEAHA118023457. DOI: 10.1161/STROKEAHA.118.023457.
29. Goyal M, Fargen KM, Turk AS, et al. 2C or not 2C: defining an improved revascularization grading scale and the need for standardization of angiography outcomes in stroke trials. J Neurointerv Surg 2014;6(2):83-6. DOI: 10.1136/neurintsurg-2013-010665.
30. von Kummer R, Broderick JP, Campbell BC, et al. The Heidelberg Bleeding Classification: Classification of Bleeding Events After Ischemic Stroke and Reperfusion Therapy. Stroke; a journal of cerebral circulation 2015;46(10):2981-6. DOI: 10.1161/STROKEAHA.115.010049.
31. Zaidat OO, Yoo AJ, Khatri P, et al. Recommendations on angiographic revascularization grading standards for acute ischemic stroke: a consensus statement. Stroke; a journal of cerebral circulation 2013;44(9):2650-63. DOI: 10.1161/STROKEAHA.113.001972.
32. Boers AM, Marquering HA, Jochem JJ, et al. Automated cerebral infarct volume measurement in follow-up noncontrast CT scans of patients with acute ischemic stroke. AJNR Am J Neuroradiol 2013;34(8):1522-7. DOI: 10.3174/ajnr.A3463.
33. Liu L, Chen W, Zhou H, et al. Chinese Stroke Association guidelines for clinical management of cerebrovascular disorders: executive summary and 2019 update of clinical management of ischaemic cerebrovascular diseases. Stroke Vasc Neurol 2020;5(2):159-176. DOI: 10.1136/svn-2020-000378.
34. EuroQol G. EuroQol--a new facility for the measurement of health-related quality of life. Health Policy 1990;16(3):199-208. DOI: 10.1016/0168-8510(90)90421-9.

二十一、 附表

附录 1. 改良 Rankin 量表

患者状况	评分
完全无症状	0
尽管有症状，但无明显功能障碍，能完成所有日常工作和生活	1
轻度残疾，不能完成病前所有活动，但不需帮助能照料自己的日常事务	2
中度残疾，需部分帮助，但能独立行走	3
中重度残疾，不能独立行走，日常生活需别人帮助	4
重度残疾，卧床，二便失禁，日常生活完全依赖他人	5
死亡	6

改良 Rankin 量表的评定方法

改良 Rankin 量表是用来衡量患者脑卒中后的功能恢复的结果。黑体字显示了每一级别的正式定义。斜体字则给予了进一步指导，以期减少不同观察者间可能产生的误差，但对面谈的架构没有要求。请注意仅考虑自脑卒中以后发生的症状。假如患者无须外界帮助，可在某些辅助装置的帮助下行走，则被视为能够独立行走。

如果两个级别对患者似乎同样适用，并且进一步提问亦不太可能做出绝对正确的选择，则应选择较为严重的一级。

0-完全没有症状

尽管可能会有轻微症状，但患者自脑卒中后，没有察觉到任何新发生的功能受限和症状。

1-尽管有症状，但未见明显残障；能完成所有经常从事的职责和活动

患者有由脑卒中引起的某些症状，无论是身体上或是认知上的（比如影响到讲话、读书、写字；或身体运动；或感觉；或视觉；或吞咽；或情感），但可继续从事所有脑卒中以前从事的工作、社会和

休闲活动。用于区分级别 1 和 2（见下）的关键问题可以是，“是否有些事情你过去经常做，但直到脑卒中以后你不能再做？”。频率超过每月一次的活动被认为是“经常”。

2-轻度残障；不能完成所有以前能从事的活动，但能处理个人事务而不需帮助

某些脑卒中以前可以完成的活动（如开车、跳舞、读书或工作），脑卒中后患者不再能够从事，但仍能每日照顾自己而无须他人协助。患者能够不需要别人的帮助穿衣、行走、吃饭、去卫生间、准备简单的食物、购物、本地出行等。患者生活无需监督。设想这一级别的患者可在无人照顾的情况下单独居家一周或更长时间。

3-中度残障；需要一些协助，但行走不需要协助

在这一级别，患者可以独立的行走（可借助辅助行走的机械）能够独立穿衣、去卫生间、吃饭等，但是更复杂的任务需要在别人协助下完成。例如，需要他人代替完成购物、做饭或打扫卫生的工作，和一周不止一次看望患者以确保完成上述活动。需要协助的不仅是照顾身体，更多的是给予建议：比如，在这一级别的患者将需要监督或鼓励来处理财务。

4-重度残障；离开他人协助不能行走，以及不能照顾自己的身体需要

患者需要其他人帮助打理日常生活，无论是行走、穿衣、去卫生间或吃饭。患者需要每天照看至少一次、通常是二次或更多次，或必须和看护者住得很近。为区分级别 4 和 5（见下），考虑患者是否能够在一天当中，常规单独生活适当的时间。

5-严重残障；卧床不起、大小便失禁、须持续护理和照顾

虽然不需受过培训的护士，但需要有人整个白天和夜间数次照看。

附录 2. 脑缺血扩展治疗（eTICI）量表

eTICI 分级	短描述	长描述
0	无灌注	闭塞点外无顺行血流
1	再灌注有限	初始闭塞后顺行再灌注，但远端分支灌注有限，几乎无远端再灌注或远端再灌注较慢
2a	再灌注＜50%	顺行再灌注不到既往缺血区域闭塞靶动脉的一半（如，MCA 及其区域的一个主要分支）
2b	再灌注≥50% 但＜90%	顺行再灌注超过既往闭塞靶动脉区域的一半(如，MCA 及其区域的两个主要分支)
2c	再灌注≥90%	除血流较慢或少量远端皮质血管中有远端闭塞以外，既往闭塞的靶动脉缺血区域几乎完全达到顺行再灌注
3	再灌注 100%	既往闭塞的靶动脉缺血区域完全达到顺行再灌注，所有远端分支均无可见闭塞

MCA:大脑中动脉；eTICI：脑缺血扩展治疗量表

附录 3. 美国国立卫生研究院卒中量表（NIHSS）

注意每次 NIHSS 评分时要记录实际评定的时间按表评分，记录结果。不要更改记分，记分所反映的是病人实际情况，而不是医生认为病人应该是什么情况。快速检查同时记录结果。除非必要的指点，不要训练病人（如反复要求病人做某种努力）。

如部分项目未评定，应在表格中详细说明。

	检查	评分
**1a**	意识水平:<br>即使不能全面评价（如气管插管、语言障碍、气管创伤、绷带包扎等），检查者也必须选择 1 个反应。只在患者对有害刺激无反应时（不是反射），方记录 3 分。	0= 清醒，反应敏锐<br>1= 嗜睡，最小刺激能唤醒患者完成指令、回答问题或有反应<br>2= 昏睡或反应迟钝，需要强烈反复刺激或疼痛刺激才能有非固定模式的反应<br>3= 仅有反射活动或自发反应，或完全没反应、软瘫、无反应
**1b**	意识水平提问：<br>（仅对最初回答评分，检查者不要提示）询问月份，年龄。回答必须正确，不能大致正常。失语和昏迷者不能理解问题记 2 分，患者因气管插管、气管创伤、严重构音障碍、语言障碍或其他任何原因不能说话者（非失语所致）记 1 分。	0= 都正确<br>1= 正确回答一个<br>2= 两个都不正确或不能说
**1c**	意识水平指令：<br>要求睁眼、闭眼：非瘫痪手握拳、张手。若双手	0= 都正确<br>1= 正确完成一个

	不能检查，用另一个指令（伸舌）。仅对最初的反应评分，有明确努力但未完成也给评分。若对指令无反应，用动作示意，然后记录评分。对创伤、截肢或其他生理缺陷者，应给予一个适宜的指令。	2= 都不正确
**2**	凝视：<br>只测试水平眼球运动。对自主或反射性（眼头）眼球运动记分。若眼球侧视能被自主或反射性活动纠正，记录 1 分。若为孤立性外周神经麻痹（III、IV、V），记 1 分。在失语患者中，凝视是可测试的。对眼球创伤、绷带包扎、盲人或有视觉或视野疾病的患者，由检查者选择一种反射性运动来测试。建立与眼球的联系，然后从一侧向另一侧运动，偶尔能发现凝视麻痹。	0= 正常<br>1= 部分凝视麻痹（单眼或双眼凝视异常，但无被动凝视或完全凝视麻痹）<br>2= 被动凝视或完全凝视麻痹<br>（不能被眼头动作克服）
**3**	视野：<br>用手指数或视威胁方法检测上、下象限视野。如果患者能看到侧面的手指，记录正常。如果单眼盲或眼球摘除，检查另一只眼。明确的非对称盲（包括象限盲），记 1 分。患者全盲（任何原因）记 3 分，同时刺激双眼。若人濒临死亡记 1 分，结果用于回答问题 11。	0= 无视野缺失<br>1= 部分偏盲<br>2= 完全偏盲<br>3= 双侧偏盲（全盲，包括皮质盲）
**4**	面瘫：	0= 正常

	言语指令或动作示意，要求患者示齿、扬眉和闭眼。对反应差或不能理解的患者，根据有害刺激时表情的对称情况评分。有面部创伤/绷带、经口气管插管、胶布或其他物理障碍影响面部检查时，应尽可能移至可评估的状态。	1= 最小（鼻唇沟变平、微笑时不对称）<br>2= 部分（下面部完全或几乎完全瘫痪，中枢性瘫）<br>3= 完全（单或双侧瘫痪，上下面部缺乏运动，周围性瘫）
**5**	上肢运动：<br>上肢伸展：坐位 90º，位卧 45º。要求坚持 10 秒；对失语的患者用语言或动作鼓励，不用有害刺激。评定者可以抬起患者的上肢到要求的位置，鼓励患者坚持。仅评定患侧。	0= 上肢于要求位置坚持 10 秒，无下落<br>1= 上肢能抬起，但不能维持 10 秒，下落时不撞击床或其他支持物<br>2= 能对抗一些重力，但上肢不能达到或维持坐位 90º 或卧位 45º，较快下落<br>3= 不能抗重力，上肢快速下落<br>4= 无运动<br>9= 截肢或关节融合，解释：________________
**6**	下肢运动：<br>下肢卧位抬高 30º，坚持 5 秒；对失语的患者用语言或动作鼓励，不用有害刺激。评定者可以抬起患者的上肢到要求的位置，鼓励患者坚持。仅评定患侧。	0= 于要求位置坚持 5 秒，不下落<br>1= 在 5 秒末下落，不撞击床<br>2= 5 秒内较快下落到床上，但可抗重力<br>3= 快速落下，不能抗重力<br>4= 无运动<br>9= 截肢或关节融合，解释：________________
**7**	共济失调：	0= 没有共济失调

	目的是发现双侧小脑病变的迹象。试验时双眼睁开，若有视觉缺损，应确保试验在无缺损视野内进行。双侧指鼻、跟膝胫研究，共济失调与无力明显不呈比例时记分。如患者不能理解或肢体瘫痪不记分。盲人用伸展的上肢摸鼻。若为截肢或关节融合，记录 9 分，并解释清楚。	1= 一个肢体有<br>2= 两个及两个以上肢体有
**8**	感觉：<br>用针检查。测试时，用针尖刺激和撤除刺激观察昏迷或失语患者的感觉和表情。只对与卒中有关的感觉缺失评分。偏身感觉丧失者需要精确检查，应测试身体多处部位：上肢（不包括手）、下肢、躯干、面部。严重或完全的感觉缺失，记 2 分。昏睡或失语者可记 1 或 0 分。脑干卒中双侧感觉缺失记 2 分。无反应及四肢瘫痪者记 2 分。昏迷患者（1a＝3）记 2 分。	0= 正常，没有感觉缺失<br>1= 轻到中度，患侧针刺感不明显或为钝性或仅有触觉<br>2= 严重到完全感觉缺失，面、上肢、下肢无触觉
**9**	语言：<br>命名、阅读测试。要求患者叫出物品名称、读所列的句子。从患者的反应以及一般神经系统检查中对指令的反应判断理解能力。若视觉缺损干扰测试，可让患者识别放在手上的物品，重复和发音。气管插管者手写回答。昏迷患者（1a＝3），3 分，给恍惚或不合作者选择一个记分，但 3 分	0= 正常，无失语<br>1= 轻到中度：流利程度和理解能力有一些缺损，但表达无明显受限。<br>2= 严重失语，交流是通过患者破碎的语言表达，听者须推理、询问、猜测，能交换的信息范围有限，检查者感交流困难。<br>3= 哑或完全失语，不能讲或不能理解

	仅给哑人或一点都不执行指令的人。	
**10**	构音障碍：<br>不要告诉患者为什么做测试。读或重复附表上的单词。若患者有严重的失语，评估自发语言时发音的清晰度。若患者气管插管或其他物理障碍不能讲话，记 9 分。同时注明原因。	0= 正常<br>1= 轻到中度，至少有一些发音不清，虽有困难，但能被理解<br>2= 言语不清，不能被理解<br>9= 气管插管或其他物理障碍，解释：________________
**11**	忽视症：<br>若患者严重视觉缺失影响双侧视觉的同时检查，皮肤刺激正常，则记分为正常。若患者失语，但确实表现为关注双侧，记分正常。通过检验患者对左右侧同时发生的皮肤感觉和视觉刺激的识别能力来判断患者是否有忽视。把标准图显示给患者，要求他来描述。医生鼓励患者仔细看图，识别图中左右侧的特征。如果患者不能识别一侧图的部分内容，则定为异常。然后，医生请患者闭眼，分别测上或下肢针刺觉来检查双侧皮肤感觉。若患者有一侧感觉忽略则为异常。	0= 没有忽视症<br>1= 视、触、听、空间觉或个人的忽视；或对任何一种感觉的双侧同时刺激消失<br>2= 严重的偏身忽视；超过一种形式的偏身忽视；不认识自己的手，只对一侧空间定位

附录 4. 欧洲五维健康量表（EQ-5D-5L）

以下各项中哪项最能描述您今天的健康状况	
行动能力	□我四处走动没有困难
	□我四处走动有一点困难
	□我四处走动有中度的困难
	□我四处走动有严重的困难
	□我无法四处走动
自我照顾	□我自己洗澡或穿衣没有困难
	□我自己洗澡或穿衣有一点困难
	□我自己洗澡或穿衣有中度的困难
	□我自己洗澡或穿衣有严重的困难
	□我无法自己洗澡或穿衣
日常活动（如工作、学习、家务、家庭或休闲活动）	□我进行日常活动没有困难
	□我进行日常活动有一点困难
	□我进行日常活动有中度的困难
	□我进行日常活动有严重的困难
	□我无法进行日常活动
疼痛或不舒服	□我没有疼痛或不舒服
	□我有一点疼痛或不舒服
	□我有中度的疼痛或不舒服
	□我有严重的疼痛或不舒服
	□我有非常严重的疼痛或不舒服
焦虑或沮丧	□我没有焦虑或沮丧
	□我有一点焦虑或沮丧
	□我有中度的焦虑或沮丧
	□我有严重的焦虑或沮丧
	□我有非常严重的焦虑或沮丧

为了帮助您反映健康状况的好坏，我们画了一个刻度尺（有点像温度计），在这刻度尺

上，100 代表您心目中最好的状况，0 代表您心目中最差的状况。请在右边的刻度尺上标出您今天的健康状况。请从下面方格中画出一条线，连到刻度尺上最能代表您今天健康状况好坏的那一点。

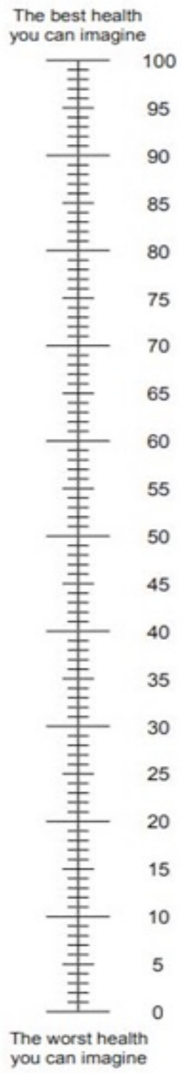

问卷的完成由：

□1 受试者本人　□2 受试者在第三者的帮助下　□3 代理人（受试者的家庭成员）

患者今天的健康状况分值：□□□分

附录 5. 海德堡出血分型

根据 2015 于德国举行的第 12 届缺血性卒中血栓治疗研讨会制定的海德堡标准将症状性颅内出血定义为（同时满足以下条件）:

1、血管内治疗后 48 小时内，由影像学检查（头 CT 或 MRI）发现，经影像科医生证实的，任何形式的颅内出血（包括海德堡标准的 1a、1b、1c、2、3a、3b、3c、3d）;

2、患者出现临床症状恶化：NIHSS 评分相对于恶化前最后的评分升高≥4 分，或 NIHSS 评分表的任何一项升高≥2 分，或导致气管插管、去骨瓣减压、脑室引流或其他重大的医疗/外科干预；

3、除了颅内出血外没有其他原因能够解释临床症状恶化。

海德堡出血分型：

分级	分型	说明
1		梗死脑组织出血
1a	HI1	分散的小出血点，无占位效应
1b	HI2	出血点融合成斑，无占位效应
1c	PH1	梗死组织内血肿<30%梗死体积，无大的占位效应，梗死组织内或超出梗死范围的脑出血
2	PH2	血肿体积≥30%梗死体积，且有明显占位效应
3		梗死组织以外脑出血
3a		梗死远隔部位血肿
3b		脑室出血
3c		蛛网膜下腔出血
3d		硬膜下出血

附录 6. Alberta 卒中项目早期 CT 评分(Alberta Stroke Program Early CT Score, ASPECTS)

Alberta 卒中项目早期 CT 评分（Alberta Stroke Program Early CT Score, ASPECTS）是一项十分制的系统性定量分析评分方法，用于评价 MCA 供应区发生急性缺血性卒中患者 NCCT 的缺血改变。MCA 供血区按照下图进行分区，缺血改变每累及一个区域则从总分 10 分中减去 1 分。10 分代表正常颅脑影像，0 分代表缺血梗死广泛累及 MCA 全部供血区。(Yoo AJ, Lancet Neurology)

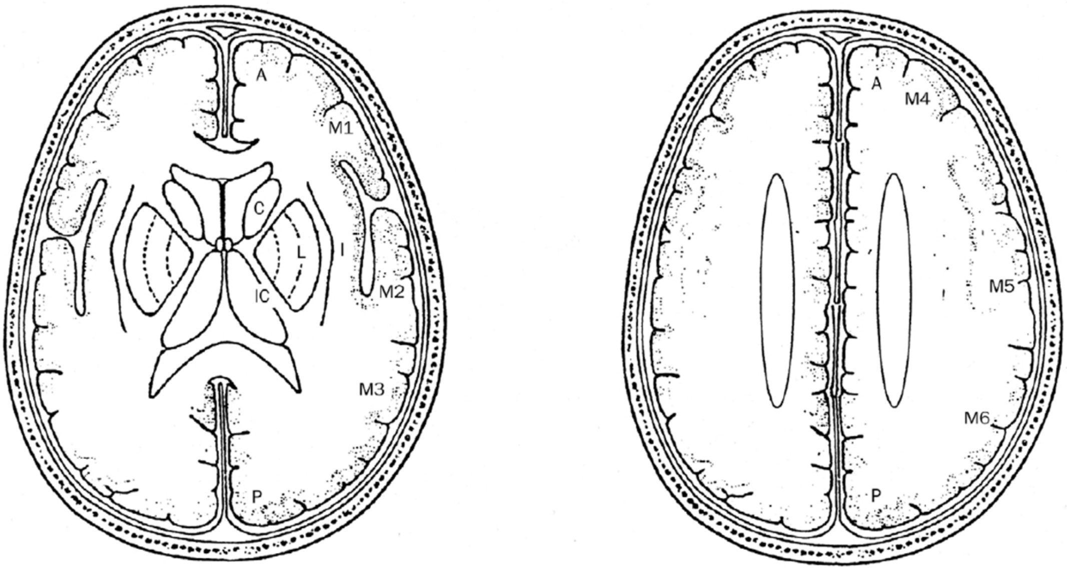

A：前循环；P：后循环。

10 个分区：C=尾状核; L=豆状核; IC=内囊; I=岛叶皮层; M1= MCA 前皮质，相当于岛盖部; M2= MCA 内囊旁皮质，相当于前颞叶；M3= MCA 后皮质，相当于后颞叶；M4=M1 上临的 MCA 前皮质；M5= M2 上临的 MCA 侧皮质; M6= M3 上临的 MCA 后皮质
