# Supplementary material for: Impact of enlarged perivascular spaces on endovascular therapy outcomes in patients with large ischaemic core: A post-hoc analysis of the ANGEL-ASPECT trial
Source: J Transl Int Med. 2026 Mar 26;14(2):294–305. doi: 10.1515/jtim-2026-0036 (PMC13110465; doi:10.1515/jtim-2026-0036)
Supplement: Supplementary file 1 — Supplementary Material Details [file jtim-2026-0036_sm.zip › 10 JTIM-D-25-00291 SI 2.pdf]

Supplement 2

Supplementary materials

Supplementary MATERIALS AND METHODS

Study population

In brief, patient inclusion criteria were 18–80 years of age, acute AIS within 24 hours after stroke onset with baseline National Institutes of Health Stroke Scale (NIHSS) score of 6–30, pre-stroke mRS of 0–1, and LVO in the initial segment of the middle cerebral artery and/or intracranial segment of the internal carotid artery on computed tomography (CT) or magnetic resonance (MR) angiography. A large infarct core volume was defined by an Alberta Stroke Program Early Computed Tomography Score (ASPECTS) of 3–5 on non-contrast CT within 24 hours after stroke onset; ASPECTS of 0–2 on non-contrast CT within 24 hours after stroke onset and an infract core volume of 70–100 mL; or an ASPECTS greater than 5 between 6 and 24 hours after stroke onset and an infarct core volume of 70–100 mL.

Assessments of other cerebral small vessel disease (CSVD) imaging markers

Lacunes were defined as rounder or ovoid lesions with a diameter of 3–15 mm, located in the internal or external capsule, basal ganglia (BG), centrum semiovale (CSO), thalamus, or brainstem, with cerebrospinal fluid signal intensity on T2-weighted and FLAIR images, generally with a hyperintense rim on FLAIR, and without increased signal on diffusion-weighted imaging. White matter hyperintensity (WMH) was rated according to the Fazekas scale and divided into periventricular WMH (PWMH) and deep WMH (DWMH). PWMH was graded as absent (score 0), cap (score 1), smooth halo (score 2), or irregular and extending into the subcortical white matter (score 3), while DWMH was rated as absent (score 0), puncatate foci (score 1), early confluent (score 2), or confluent (score 3).

Supplementary RESULTS

Supplementary analyses: Modification of EVT treatment effect by CSO-EPVS severity

Demographic and clinical characteristics at baseline between the EVT and medical management groups across the three CSO-EPVS subgroups are summarized in Supplementary Table S3. As shown in Supplementary Table S9, univariate and multivariate regression analyses revealed that CSO-EPVS severity was not significantly associated with either functional outcomes or safety endpoints among patients treated with EVT (all $P > 0.05$).

For the primary outcome, a shift in the distribution of 90-day mRS scores toward better outcome in the EVT group compared to the medical management group was observed across all three CSO-EPVS subgroups (adjusted cOR 5.84, 95% CI 2.03–16.82, $P < 0.001$; adjusted cOR 3.40, 95% CI 1.52–7.59, $P = 0.003$; adjusted cOR 3.28, 95% CI 1.23–8.76, $P = 0.018$) (Supplementary Table S10 and Supplementary Figure S2). Importantly, no significant treatment-by-CSO-EPVS interaction was observed across the three CSO-EPVS subgroups ($P\_interaction$ = 0.232). This suggests that, unlike BG-EPVS, the severity of CSO-EPVS may not substantially modify the functional benefit of EVT.

Regarding the secondary outcomes, EVT was associated with higher rates of achieving an mRS score of 0–2 compared with medical management in the none-to-mild (adjusted OR 8.58, 95% CI 2.20–33.40, $P = 0.002$) and moderate (adjusted OR 5.48, 95% CI 1.88–16.00, $P = 0.002$) CSO-EPVS subgroups. In the severe CSO-EPVS subgroup, this association was marginally significant (adjusted OR 3.76, 95% CI 0.84–16.93, $P = 0.084$). Additionally, EVT was associated with higher rates of achieving an mRS score of 0–3 in the none-to-mild CSO-EPVS subgroup (adjusted OR 5.21, 95% CI 1.48–18.36, $P = 0.010$), but not in the moderate (adjusted OR 1.89, 95% CI 0.70–5.13, $P = 0.210$) or severe CSO-EPVS subgroup (adjusted OR 2.43, 95% CI 0.73–8.06, $P = 0.146$). However, no significant treatment-by-CSO-EPVS interaction effects

were observed for either outcome (*P_interaction* for mRS score of 0–2 = 0.479, *P_interaction* for mRS score of 0–3 = 0.245) (Supplementary Table S10).

EVT was associated with higher rates of target artery recanalization at 36 hours compared with medical management across all three CSO-EPVS subgroups (None-to-mild: 91.7% *vs.* 31.6%; Moderate: 89.7% *vs.* 40.8%; Severe: 75.0% *vs.* 37.5%) (Supplementary Table S10). These findings indicate that EVT consistently promoted early vessel recanalization regardless of CSO-EPVS severity.

With regard to safety outcomes, EVT was associated with a significantly higher rate of any ICH within 48 hours compared to medical management in the none-to-mild CSO-EPVS subgroup (adjusted OR 4.91, 95% CI 1.16–20.859, *P* = 0.031). In contrast, this association was not statistically significant in the moderate (adjusted OR 2.92, 95% CI 0.97–8.86, *P* = 0.058) or severe (adjusted OR 2.60, 95% CI 0.63–10.62, *P* = 0.185) CSO-EPVS subgroups. No significant differences were observed between the EVT and medical management groups in the rates of symptomatic ICH or 90-day mortality across any of the CSO-EPVS subgroups (all adjusted *P* > 0.05) (Supplementary Table S10).

We found that further adjustment for additional CSVD imaging markers (i.e., lacunes, PWMH and DWMH) did not materially alter the findings (Supplementary Table S11). In sensitivity analyses that additionally adjusted for study center, MRI field strength, and slice thickness, the effect estimates for EVT and the treatment–by–BG-EPVS interactions remained similar to those of the primary models, and none of the main conclusions was materially altered (Supplementary Table S12).

Supplementary Table S1: List of committees and coordinating centres in ANGEL-ASPECT

Study organization	Member	Department	Hospital
**Steering Committee**	Yongjun Wang	Department of Neurology	Beijing Tiantan Hospital, Capital Medical University
	Yilong Wang	Department of Neurology	Beijing Tiantan Hospital, Capital Medical University

Advisory Board	Liping Liu	Department of Neurology	Beijing Tiantan Hospital, Capital Medical University
	David S. Liebeskind	Department of Neurology	University of California at Los Angeles
	Zhongrong Miao	Department of Interventional Neuroradiology	Beijing Tiantan Hospital, Capital Medical University
	Zeguang Ren	Department of Neurosurgery	The Affiliated Hospital of Guizhou Medical University
	Vitor Mendes Pereira	Department of Neurosurgery, Division of Surgery	St Michael's Hospital, University of Toronto
	Xunming Ji	Department of Neurosurgery	Xuanwu Hospital, Capital Medical University
	Qiang Dong	Department of Neurology	Huashan Hospital, Fudan University
	Anding Xu	Department of Neurology and Stroke Centre	The First Affiliated Hospital, Jinan University
	Xinfeng Liu	Department of Neurology	Affiliated Jinling Hospital, Medical School of Nanjing University
	Qingwu Yang	Department of Neurology	Xinqiao Hospital and The Second Affiliated Hospital, Army Medical University
Independent Imaging Core Lab	Jing Jing (Chair)	Tiantan Neuroimaging Centre of Excellence (TNICE)	China National Clinical Research Centre for Neurological Diseases
	Zhe Zhang	Tiantan Neuroimaging Centre of Excellence (TNICE)	China National Clinical Research Centre for Neurological Diseases
	Yingkui Zhang	Tiantan Neuroimaging Centre of Excellence (TNICE)	China National Clinical Research Centre for Neurological Diseases
	Wei Wu	Department of Neurology	Qilu Hospital, Shandong University
	Dapeng Sun	Department of Interventional Neuroradiology	Beijing Tiantan Hospital, Capital Medical University
	Zhongqi Qi	Department of Interventional Neuroradiology	Beijing Tiantan Hospital, Capital Medical University
	Shuo Li	Department of Interventional Neuroradiology	Beijing Tiantan Hospital, Capital Medical University
	Zhenqiang	Department of	Beijing Tiantan Hospital,

	Liu	Interventional Neuroradiology	Capital Medical University
	Zequan Yu	Department of Interventional Neuroradiology	Beijing Tiantan Hospital, Capital Medical University
	Jingyu Zhang	Department of Interventional Neuroradiology	Beijing Tiantan Hospital, Capital Medical University
	Fangguang Chen	Department of Interventional Neuroradiology	Beijing Tiantan Hospital, Capital Medical University
	Kangyue Li	Department of Interventional Neuroradiology	Beijing Tiantan Hospital, Capital Medical University
	Kai Zhang	Department of Interventional Neuroradiology	Beijing Tiantan Hospital, Capital Medical University
	Mingkai Hu	Department of Interventional Neuroradiology	Beijing Tiantan Hospital, Capital Medical University
**Data Safety and Monitoring Board**	Jianmin Liu (Chair)	Neurovascular Centre	Changhai Hospital, Naval Medical University
	Chen Yao	Department of Medical Statistics	Peking University First Hospital
	Kangning Chen	Department of Neurology	The Southwest Hospital of Army Medical University
**Clinical Event Committee**	Kun Fang (Chair)	Department of Neurology	Huashan Hospital, Fudan University
	Bo Song	Department of Neurology	The First Affiliated Hospital of Zhengzhou University
	Yi Dong	Department of Neurology	Huashan Hospital, Fudan University
**Executive Committee**	Zhongrong Miao	Department of Interventional Neuroradiology	Beijing Tiantan Hospital, Capital Medical University
	Xiaochuan Huo	Department of Interventional Neuroradiology	Beijing Tiantan Hospital, Capital Medical University
	Gaoting Ma	Department of Interventional Neuroradiology	Beijing Tiantan Hospital, Capital Medical University
	Guangxiong Yuan	Department of Emergency	Xiangtan Central Hospital
	Hongxing Han	Department of Neurology	Linyi People's Hospital
	Wenhuo	Department of	Zhangzhou Affiliated Hospital

Chen	Neurology	of Fujian Medical University
Ming Wei	Department of Neurosurgery	Tianjin huanhu hospital
Jiangang Zhang	Department ofNeurology	Anyang People's Hospital
Zhiming Zhou	Department of Neurology	Yijishan Hospital of Wannan Medical College
Xiaoxi Yao	Department of Neurology	The first people's hospital of Chenzhou
Guoqing Wang	Department of Neurology	Binzhou People's Hospital
Weigen Song	Department of Neurology	Yancheng Third People's Hospital
Xueli Cai	Department of Neurology	Lishui Municipal Central Hospital
Guangxian Nan	Department of Neurology	China-Japan Union Hospital of Jilin University
Di Li	Department of Neurointervention	Dalian Municipal Central Hospital affiliated with Dalian Medical University
Yizhou Wang	Department of Neurosurgery	Guangdong Provincial Hospital of Chinese Medicine
Wentong Ling	Department of Neurology	ZhongShan City People's Hospital
Chuwei Cai	Department of Neurology	Shantou Central Hospital
Changming Wen	Department of Neurology	Nanyang Central Hospital
En Wang	Department of Neurology	Taizhou hospital of Zhejiang Province
Liyong Zhang	Department of Neurosurgery	Liaocheng People's Hospital
Changchun Jiang	Department of Neurology	Baotou Centre Hospital
Yajie Liu	Department of Neurology	Shenzhen Hospital, Southern Medical University
Geng Liao	Department of Neurology	Maoming People's Hospital
Xiaohui Chen	Department of Neurology	The Second Affiliated Hospital of GuangZhou Medical University
Tianxiao Li	Department of Cerebrovascular Disease	Henan Provincial People's Hospital, Zhengzhou University
Shudong Liu	Department of Neurology	Yongchuan Hospital of Chongqing Medical University

Jinglun Li	Department of Neurology	The affiliated hospital of South West medical university
Yaxuan Sun	Department of Neurology	Shanxi Provincial People's Hospital
Na Xu	Department of Neurology	The Second Affiliated Hospital to Xiamen Medical College
Zong'en Gao	Department of Neurology	Shengli Oilfield Central Hospital
Dongsheng Ju	Department of Neurology	Songyuan Jilin oil Field Hospital
Cunfeng Song	Department of Interventional Neuroradiology	Liao Cheng the third people's hospital
Jinggang Xuan	Department of Neurology	The First People's Hospital of Changzhou
Feng Zhou	Department of Neurology	Taiyuan Central Hospital
Qing Shi	Department of Neurology	Affiliated Jiangmen Traditional Chinese Medicine Hospital of Ji’nan University
Jun Luo	Department of Neurology	Sichuan Mianyang 404 Hospital
Yan Liu	Department of Neurology	JingJiang People's Hospital, the Seventh Affiliated Hospital of Yangzhou University
Zaiyu Guo	Department of Neurosurgery	Tianjin TEDA Hospital
Tong Li	Department of Neurosurgery	The second Nanning People's Hospital
Hongbo Zheng	Department of Neurology	West China Hospital, Sichuan University
Linzhi Dai	Department of Neurosurgery	First Affiliated Hospital School of Medicine Shihezi University
Junfeng Zhao	Department of Neurology	Siping Central People's Hospital
Liqiang Gui	Emergency and Critical Stroke Ambulance Centre	Langfang Changzheng Hospital
Xiaokun Geng	Department of Neurology	Beijing Luhe Hospital, Capital Medical University
Yufeng Tang	Department of Neurology	Mianyang Central Hospital
Congguo Yin	Department of Neurology	Hangzhou First People’s Hospital
Hua Yang	Department of	The affiliated Hospital of

		Neurosurgery	Guizhou Medical University
**Trial Manager**	Xiaochuan Huo	Department of Interventional Neuroradiology	Beijing Tiantan Hospital, Capital Medical University
	Gaoting Ma	Department of Interventional Neuroradiology	Beijing Tiantan Hospital, Capital Medical University
**Independent Monitoring Committee**	Ruiyang An	Quality Assurance	Ericure Medical Technology. Co., Ltd.
	Yuying Sun	Quality Assurance	Ericure Medical Technology. Co., Ltd.
	Yanan Wu	Quality Assurance	Ericure Medical Technology. Co., Ltd.
	Chunlai Yu	Quality Assurance	Ericure Medical Technology. Co., Ltd.
	Shuangcheng Zheng	Quality Assurance	Ericure Medical Technology. Co., Ltd.
**Statistical and Data Management Center**	Yuesong Pan	China National Clinical Research Centre for Neurological Diseases	Beijing Tiantan Hospital, Capital Medical University
	Aoming Jin	China National Clinical Research Centre for Neurological Diseases	Beijing Tiantan Hospital, Capital Medical University
	Xianglong Xiang	China National Clinical Research Centre for Neurological Diseases	Beijing Tiantan Hospital, Capital Medical University
	Mengxing Wang	China National Clinical Research Centre for Neurological Diseases	Beijing Tiantan Hospital, Capital Medical University
	Hongyi Yan	China National Clinical Research Centre for Neurological Diseases	Beijing Tiantan Hospital, Capital Medical University
**Independent DSMB Statistics Team**	Yuanling He	Senior Statistician	Blueballoon Medical Research Co. LTD
	Chunyang Li	Project department	Blueballoon Medical Research Co. LTD
	Weixia Kong	Medicine department	Blueballoon Medical Research Co. LTD
**Contract Research Organization**	Yuhuan Chen	Project Operation	Wisemed Medical Technology. Co., Ltd.
	Chenhao Guo	Project Operation	Wisemed Medical Technology. Co., Ltd.
	Fengjie Ji	Project Operation	Wisemed Medical Technology. Co., Ltd.
	Pengshan Ji	Project Operation	Wisemed Medical Technology. Co., Ltd.

Lei Liu	Project Operation	Wisemed Medical Technology. Co., Ltd.
Xinghua Lu	Project Operation	Wisemed Medical Technology. Co., Ltd.
Guangkuo Luo	Project Operation	Wisemed Medical Technology. Co., Ltd.
Nanjing Wang	Project Operation	Wisemed Medical Technology. Co., Ltd.
Yu Zhang	Project Operation	Wisemed Medical Technology. Co., Ltd.
Bo Liu	Data Management	Wisemed Medical Technology. Co., Ltd.
Jian Yang	Data Management	Wisemed Medical Technology. Co., Ltd.
Jingjing Deng	Medical Sciences	Wisemed Medical Technology. Co., Ltd.
Juan Wang	Medical Sciences	Wisemed Medical Technology. Co., Ltd.
Wanru Wang	Medical Sciences	Wisemed Medical Technology. Co., Ltd.
Hang Yu	Medical Sciences	Wisemed Medical Technology. Co., Ltd.
Le Cui	IT Support	Wisemed Medical Technology. Co., Ltd.
Wenwen Liu	IT Support	Wisemed Medical Technology. Co., Ltd.
Ziyong Wang	IT Support	Wisemed Medical Technology. Co., Ltd.
Xia Zhao	IT Support	Wisemed Medical Technology. Co., Ltd.
Zhou Zhou	IT Support	Wisemed Medical Technology. Co., Ltd.

Supplementary Table S2: Baseline demographic and clinical characteristics of included *vs.* excluded patients, stratified by BG-EPVS severity

**Baseline characteristics**	**Excluded (*n* = 229)**	**Included (*n* = 226)**	***P* value**
Age, median (IQR), years	69.0 (61.0–74.0)	67.0 (59.0–72.0)	0.042
Male Sex, *n* (%)	104 (45.4)	73 (32.3)	0.004
Hypertension, *n* (%)	140 (61.1)	132 (58.4)	0.553
Diabetes mellitus, *n* (%)	47 (20.5)	36 (15.9)	0.205
Hyperlipidemia, *n* (%)	16 (7.0)	10 (4.4)	0.239

Atrial fibrillation, *n* (%)	63 (27.5)	41 (18.1)	0.017
Coronary heart disease, *n* (%)	46 (20.1)	37 (16.4)	0.305
Ischemic stroke, *n* (%)	37 (16.2)	36 (15.9)	0.947
Cigarette smoking, *n* (%)	64 (28.0)	80 (35.4)	0.088
Wake-up Stroke, *n* (%)	73 (31.9)	74 (32.7)	0.844
SBP, median (IQR), mmHg	150.0 (134.0–169.0)	149.0 (130.0–165.0)	0.234
DBP, median (IQR), mmHg	86.0 (75.0–96.0)	85.0 (76.0–94.0)	0.606
Baseline NIHSS, median (IQR)	16.0 (13.0–20.0)	15.0 (12.0–19.0)	0.005
ASPECTS, median (IQR)	3.0 (3.0–4.0)	3.0 (3.0–4.0)	0.912
Occlusion site, *n* (%)			0.099
ICA	87 (38.0)	77 (34.1)	
M1	142 (62.0)	145 (64.2)	
M2	0 (0.0)	4 (1.8)	
Ipsilateral extracranial ICAO, *n* (%)	37 (16.2)	39 (17.3)	0.753
Intravenous thrombolysis, *n* (%)	61 (26.6)	68 (30.1)	0.414
Stroke classification, *n* (%)			0.084
Atherothrombotic	52 (22.7)	61 (27.0)	
Cardioembolic	117 (51.1)	92 (40.7)	
Undetermined or other	60 (26.2)	73 (32.3)	
Infarct core volume, median (IQR), mL	60.0 (22.6–88.0)	64.0 (40.0–83.0)	0.438
Time from onset to door, median (IQR), min	331.0 (186.0–630.0)	354.5 (198.0–652.0)	0.599
Time from onset to imaging, median (IQR), min	381.0 (238.0–677.0)	423.5 (267.0–733.0)	0.297
Time from onset to randomization, median (IQR), min	446.0 (294.0–712.0)	473.0 (311.0–768.0)	0.366

ASPECTS: Alberta Stroke Program Early CT Scores; BG-EPVS: enlarged perivascular spaces (EPVS) in the basal ganglia (BG); DBP: diastolic blood pressure; EVT: endovascular therapy; IQR: interquartile range; ICA: internal carotid artery; ICAO: internal carotid artery occlusion; M1: main trunk of the middle cerebral artery; M2 segment: first-order branch of the main trunk; MM: medical management; mRS: modified Rankin Scale; NIHSS: National Institutes of Health Stroke Scale; SBP: systolic blood pressure.

Supplementary Table S3: Baseline demographic and clinical characteristics of patients in the ANGEL-ASPECT trial, stratified by CSO-EPVS severity

	None-to-mild CSO-EPVS (*n* = 68)			Moderate CSO-EPVS (*n* = 89)			Severe CSO-EPVS (*n* = 66)		
**Baseline characteristics**	**MM group (*n* =**	**EVT group (*n* =**	***P* value**	**MM group (*n* =**	**EVT group (*n* =**	***P* value**	**MM group (*n* =**	**EVT group (*n* =**	***P* value**

	43)	25)		50)	39)		34)	32)	
Age, median (IQR), years	62.0 (54.0–71.0)	67.0 (61.0–72.0)	0.125	68.0 (57.0–73.0)	67.0 (60.0–73.0)	0.785	68.0 (64.0–72.0)	67.5 (58.0–73.0)	0.603
Male Sex, *n* (%)	30 (69.8)	13 (52.0)	0.143	39 (78.0)	25 (64.1)	0.148	21 (61.8)	24 (75.0)	0.249
Hypertension, *n* (%)	27 (62.8)	15 (60.0)	0.819	22 (44.0)	25 (64.1)	0.059	23 (67.7)	18 (56.3)	0.340
Diabetes mellitus, *n* (%)	4 (9.3)	4 (16.0)	0.409	9 (18.0)	4 (10.3)	0.305	7 (20.6)	8 (25.0)	0.669
Hyperlipidemia, *n* (%)	5 (11.6)	0 (0.0)	0.077	0 (0.0)	3 (7.7)	0.046	1 (2.94)	1 (3.13)	0.965
Atrial fibrillation, *n* (%)	5 (11.6)	3 (12.0)	0.963	10 (20.0)	12 (30.8)	0.243	4 (11.8)	7 (21.9)	0.271
Coronary heart disease, *n* (%)	8 (18.6)	4 (16.0)	0.786	12 (24.0)	8 (20.5)	0.696	2 (5.9)	3 (9.4)	0.592
Ischemic stroke, *n* (%)	6 (14.0)	4 (16.0)	0.818	9 (18.0)	6 (15.4)	0.744	9 (26.5)	2 (6.3)	0.028
Cigarette smoking, *n* (%)	16 (37.2)	10 (40.0)	0.819	18 (36.0)	13 (33.3)	0.793	11 (32.4)	12 (37.5)	0.661
Wake-up Stroke, *n* (%)	13 (30.2)	7 (28.0)	0.846	17 (34.0)	13 (33.3)	0.947	13 (38.2)	11 (34.4)	0.745
SBP, median (IQR), mmHg	156.0 (145.0–171.0)	135.0 (120.0–163.0)	0.018	145.0 (129.0–160.0)	147.0 (138.0–166.0)	0.646	151.0 (130.0–166.0)	149.5 (125.0–163.0)	0.663
DBP, median (IQR), mmHg	85.0 (78.0–94.0)	80.0 (73.0–87.0)	0.113	83.5 (75.0–92.0)	86.0 (78.0–98.0)	0.293	89.5 (79.0–104.0)	83.5 (72.5–93.0)	0.182
Baseline NIHSS, median (IQR)	14.0 (12.0–18.0)	15.0 (13.0–19.0)	0.418	15.5 (12.0–19.0)	15.0 (12.0–19.0)	0.788	14.5 (12.0–16.0)	15.0 (12.5–19.0)	0.276
ASPECTS, median (IQR)	3.0 (3.0–4.0)	4.0 (3.0–4.0)	0.989	3.5 (3.0–4.0)	3.0 (3.0–4.0)	0.699	3.0 (3.0–5.0)	3.0 (3.0–4.0)	0.644
Occlusion site, *n* (%)			0.211			0.113			0.546
ICA	20 (46.5)	7 (28.0)		16 (32.0)	17 (43.6)		9 (26.5)	7 (21.9)	
M1	22 (51.2)	18 (72.0)		34 (68.0)	20 (51.3)		24 (70.6)	25 (78.1)	
M2	1 (2.3)	0 (0.0)		0 (0.0)	2 (5.1)		1 (2.9)	0 (0.0)	
Ipsilateral extracranial ICAO, *n* (%)	8 (18.6)	5 (20.0)	0.888	6 (12.0)	9 (23.1)	0.166	6 (17.7)	5 (15.6)	0.826

Intravenous thrombolysis, *n* (%)	14 (32.6)	8 (32.0)	0.962	13 (26.0)	13 (33.3)	0.450	10 (29.4)	8 (25.0)	0.688
Stroke classification, *n* (%)			0.515			0.206			0.084
Atherothrombotic	12 (27.9)	4 (16.0)		9 (18.0)	11 (28.2)		13 (38.2)	11 (34.4)	
Cardioembolic	16 (37.2)	10 (40.0)		21 (42.0)	19 (48.7)		9 (26.5)	16 (50.0)	
Undetermined or other	15 (34.9)	11 (44.0)		20 (40.0)	9 (23.1)		12 (35.3)	5 (15.6)	
Infarct core volume, median (IQR), mL	68.0 (54.0–87.0)	67.0 (47.0–81.0)	0.571	61.0 (35.0–89.0)	52.0 (34.0–77.0)	0.444	61.5 (28.0–94.0)	61.5 (18.0–78.0)	0.389
Time from onset to door, median (IQR), min	397.0 (198.0–640.0)	355.0 (151.0–720.0)	0.755	357.0 (199.0–652.0)	360.0 (193.0–611.0)	0.937	405.0 (243.0–741.0)	303.0 (183.5–622.5)	0.213
Time from onset to imaging, median (IQR), min	453.0 (282.0–808.0)	403.0 (255.0–639.0)	0.755	465.0 (247.0–702.0)	380.0 (252.0–638.0)	0.701	458.0 (291.0–843.0)	348.5 (245.5–718.0)	0.426
Time from onset to randomization, median (IQR), min	554.0 (317.0–855.0)	463.0 (268.0–701.0)	0.537	495.0 (311.0–755.0)	402.0 (293.0–701.0)	0.698	473.5 (315.0–897.0)	371.5 (277.0–771.5)	0.379
CSVD markers									
D-WMH, median (IQR)	1.0 (1.0–1.0)	1.0 (0.0–1.0)	0.848	1.0 (1.0–1.0)	1.0 (1.0–2.0)	0.235	1.0 (1.0–1.0)	1.0 (1.0–1.0)	0.792
PV-WMH, median (IQR)	1.0 (1.0–1.0)	1.0 (1.0–1.0)	0.993	1.0 (1.0–2.0)	2.0 (1.0–2.0)	0.083	1.5 (1.0–2.0)	1.0 (1.0–2.0)	1.000
Lacune, *n* (%)	8 (18.6)	4 (17.4)	0.903	14 (29.2)	11 (29.0)	0.982	16 (47.1)	10 (32.3)	0.224

CSO-EPVS (none-to-mild) indicated 0~10 perivascular spaces in centrum semiovale (CSO); CSO-EPVS (moderate-to-severe) indicated 11~20 perivascular spaces in CSO; CSO-EPVS (severe) indicated >20 perivascular spaces in CSO. ASPECTS: Alberta Stroke Program Early CT Scores; CSO-EPVS: enlarged perivascular spaces (EPVS) in the centrum semiovale (CSO); CSVD: cerebral small vessel disease; DBP: diastolic blood pressure; D-WMH: deep-WMH; EVT: endovascular therapy; IQR: interquartile range; ICA: internal carotid artery; ICAO: internal carotid artery occlusion; M1: main trunk of the middle

cerebral artery; M2 segment: first-order branch of the main trunk; MM: medical management; mRS: modified Rankin Scale; NIHSS: National Institutes of Health Stroke Scale; PV-WMH: periventricular-WMH; SBP: systolic blood pressure; WMH: white matter hyperintensity.

Supplementary Table S4: Baseline demographic and clinical characteristics of included *vs.* excluded patients, stratified by CSO-EPVS severity

Baseline characteristics	Excluded (*n* = 233)	Included (*n* = 223)	*P* value
Age, median (IQR), years	69.0 (61.0–74.0)	67.0 (59.0–72.0)	0.060
Male Sex, *n* (%)	126 (54.3)	152 (68.2)	0.002
Hypertension, *n* (%)	142 (61.2)	130 (58.3)	0.527
Diabetes mellitus, *n* (%)	47 (20.3)	36 (16.1)	0.256
Hyperlipidemia, *n* (%)	16 (6.9)	10 (4.5)	0.268
Atrial fibrillation, *n* (%)	63 (27.2)	41 (18.4)	0.026
Coronary heart disease, *n* (%)	46 (19.8)	37 (16.6)	0.372
Ischemic stroke, *n* (%)	37 (16.0)	36 (16.1)	0.955
Cigarette smoking, *n* (%)	64 (27.6)	80 (35.9)	0.057
Wake-up Stroke, *n* (%)	73 (31.5)	74 (33.2)	0.695
SBP, median (IQR), mmHg	149.5 (134.0–168.5)	149.0 (130.0–166.0)	0.289
DBP, median (IQR), mmHg	86.0 (75.0–96.0)	85.0 (76.0–94.0)	0.692
Baseline NIHSS, median (IQR)	16.0 (13.0–20.0)	15.0 (12.0–19.0)	0.004
ASPECTS, median (IQR)	3.0 (3.0–4.0)	3.0 (3.0–4.0)	0.968
Occlusion site, *n* (%)			0.095
ICA	88 (37.9)	76 (34.1)	
M1	144 (62.1)	143 (64.1)	
M2	0 (0.0)	4 (1.8)	
Ipsilateral extracranial ICAO, *n* (%)	37 (16.0)	39 (17.5)	0.660
Intravenous thrombolysis, *n* (%)	63 (27.2)	66 (29.6)	0.564
Stroke classification, *n* (%)			0.098
Atherothrombotic	53 (22.8)	60 (26.9)	
Cardioembolic	118 (50.9)	91 (40.8)	
Undetermined or other	61 (26.3)	72 (32.3)	
Infarct core volume, median (IQR), mL	59.0 (22.3–88.5)	64.0 (40.0–83.0)	0.439
Time from onset to door, median (IQR), min	331.0 (189.0–629.5)	355.0 (197.0–659.0)	0.608
Time from onset to imaging, median (IQR), min	380.0 (238.5–675.5)	427.0 (255.0–741.0)	0.281
Time from onset to randomization, median (IQR), min	444.5 (296.5–711.0)	474.0 (308.0–775.0)	0.349

ASPECTS: Alberta Stroke Program Early CT Scores; CSO-EPVS: enlarged perivascular spaces (EPVS) in the centrum semiovale (CSO); DBP: diastolic blood pressure; EVT: endovascular therapy; IQR: interquartile range; ICA: internal carotid artery; ICAO: internal carotid artery occlusion; M1: main trunk of the middle cerebral artery; M2 segment: first-order branch of the main trunk; MM: medical management; mRS: modified Rankin Scale; NIHSS: National Institutes of Health Stroke Scale; SBP:

systolic blood pressure.

Supplementary Table S5: Baseline demographic and clinical characteristics of patients in the ANGEL-ASPECT Trial, stratified by BG-EPVS severity

None-to-mild BG-EPVS (*n* = 136)			Moderate BG-EPVS (*n* = 60)		
MM group (*n* = 87)	EVT group (*n* = 49)	*P* value	MM group (*n* = 25)	EVT group (*n* = 35)	*P* value
64.0 (56.0–72.0)	64.0 (56.0–71.0)	0.883	68.0 (61.0–73.0)	69.0 (65.0–74.0)	0.375
62 (71.3)	37 (75.5)	0.593	15 (60.0)	19 (54.3)	0.660
49 (56.3)	25 (51.0)	0.551	14 (56.0)	25 (71.4)	0.217
11 (12.6)	5 (10.2)	0.672	7 (28.0)	9 (25.7)	0.844
4 (4.6)	1 (2.0)	0.447	1 (4.0)	3 (8.6)	0.484
15 (17.2)	9 (18.4)	0.869	2 (8.0)	9 (25.7)	0.080
13 (14.9)	8 (16.3)	0.830	6 (24.0)	7 (20.0)	0.711
14 (16.1)	2 (4.1)	0.037	7 (28.0)	7 (20.0)	0.470
32 (36.8)	21 (42.9)	0.486	6 (24.0)	12 (34.3)	0.391
32 (36.80	12 (24.5)	0.141	6 (24.0)	14 (40.0)	0.195
151.0 (130.0–166.0)	140.0 (124.0–157.0)	0.033	145.0 (140.0–157.0)	149.0 (128.0–167.0)	0.679
85.0 (78.0–94.0)	83.0 (73.0–91.0)	0.291	85.0 (74.0–96.0)	85.0 (72.0–92.0)	0.775
14.0 (11.0–18.0)	15.0 (13.0–19.0)	0.284	15.0 (12.0–19.0)	16.0 (13.0–19.0)	0.599
3.0 (3.0–4.0)	3.0 (3.0–4.0)	0.623	3.0 (3.0–4.0)	3.0 (3.0–4.0)	0.804
		0.644			0.672
35 (40.2)	16 (32.7)		8 (32.0)	12 (34.3)	
51 (58.6)	32 (65.3)		17 (68.0)	22 (62.9)	
1 (1.2)	1 (2.0)		0 (0.0)	1 (2.9)	
16 (18.4)	11 (22.5)	0.569	4 (16.0)	6 (17.1)	0.907
24 (27.6)	17 (34.7)	0.386	8 (32.0)	9 (25.7)	0.594
		0.683			0.481
20 (23.0)	12 (24.5)		10 (40.0)	12 (34.3)	
34 (39.1)	22 (44.9)		7 (28.0)	15 (42.9)	
33 (37.9)	15 (30.6)		8 (32.0)	8 (22.9)	
62.0 (44.0–85.0)	60.0 (28.0–78.0)	0.335	69.0 (46.0–95.0)	58.0 (34.0–78.0)	0.227
399.0 (241.0–659.0)	355.0 (150.0–599.0)	0.356	344.0 (142.0–733.0)	300.0 (223.0–632.0)	0.893
470.0 (292.0–767.0)	374.0 (233.0–624.0)	0.224	438.0 (226.0–742.0)	420.0 (276.0–701.0)	0.828
530.0 (335.0–814.0)	405.0 (268.0–656.0)	0.157	559.0 (251.0–775.0)	472.0 (316.0–736.0)	0.869
1.0 (1.0–1.0)	1.0 (0.0–1.0)	0.806	1.0 (1.0–1.0)	1.0 (1.0–2.0)	0.180
1.0 (1.0–1.0)	1.0 (1.0–2.0)	0.409	2.0 (1.0–2.0)	2.0 (1.0–2.0)	0.877
19 (22.1)	5 (10.9)	0.111	14 (58.3)	17 (50.0)	0.531

ASPECTS: Alberta Stroke Program Early CT Scores; BG-EPVS: enlarged perivascular spaces (EPVS) in the basal ganglia (BG); CSVD: cerebral small vessel disease; DBP: diastolic blood pressure; D-WMH: deep-WMH; EVT: endovascular therapy; IQR: interquartile range; ICA: internal carotid artery; ICAO:

internal carotid artery occlusion; M1: main trunk of the middle cerebral artery; M2 segment: first-order branch of the main trunk; MM: medical management; mRS: modified Rankin Scale; NIHSS: National Institutes of Health Stroke Scale; PV-WMH: periventricular-WMH; SBP: systolic blood pressure; WMH: white matter hyperintensity. BG-EPVS (none-to-mild) indicated 0-10 perivascular spaces in basal ganglia (BG); BG-EPVS (moderate-to-severe) indicated 11-20 perivascular spaces in BG; BG-EPVS (severe) indicated >20 perivascular spaces in BG.

Supplementary Table S6: Relationship between BG-EPVS severity and functional outcomes and safety endpoints among patients treated with EVT

**Outcome**		**Model 1: Unadjusted Effect size (95% CI)** [b]	***P* value**	**Model 2: Adjust Effect size (95% CI)** [b, c]	***P* value**
**Primary outcome**					
mRS score, median (IQR)					
None-to-mild (*n* = 49)	2 (2–3)	Ref		Ref	
Moderate (*n* = 35)	4 (2–4)	0.43 (0.19 to 0.93)	0.032	0.62 (0.27 to 1.43)	0.259
Severe (*n* = 14)	4 (3–5)	0.20 (0.07 to 0.60)	0.004	0.34 (0.11 to 1.04)	0.058
**Secondary outcomes**					
mRS score 0-2, *n* (%)					
None-to-mild (*n* = 49)	28 (57.1)	Ref		Ref	
Moderate (*n* = 35)	12 (34.3)	0.39 (0.16 to 0.96)	0.041	0.47 (0.18 to 1.27)	0.137
Severe (*n* = 14)	3 (21.4)	0.21 (0.05 to 0.83)	0.026	0.23 (0.05 to 1.03)	0.055
mRS score 0-3, *n* (%)					
None-to-mild (*n* = 49)	39 (79.6))	Ref		Ref	
Moderate (*n* = 35)	16 (45.7)	0.22 (0.08 to 0.57)	0.002	0.32 (0.11 to 0.95)	0.040
Severe (*n* = 14)	4 (28.6)	0.10 (0.03 to 0.40)	0.001	0.12 (0.03 to 0.59)	0.009
ENI, *n* (%)					
None-to-mild (*n* = 49)	9 (18.4)	Ref		Ref	
Moderate (*n* = 35)	2 (5.7)	0.27 (0.05 to 1.33)	0.108	0.26 (0.05 to 1.50)	0.133
Severe (*n* = 14)	0 (0.0)	NA	0.954	NA	0.966
Change in ICV from baseline to follow-up, median (IQR), mL					
None-to-mild (*n* = 49)	48.7 (10.9–86.3)	Ref		Ref	
Moderate (*n* = 35)	50.7 (30.4–98.5)	-8.41 (-41.81 to 25.00)	0.619	1.51 (-35.19 to 38.21)	0.935
Severe (*n* = 14)	63.4 (8.4–149.8)	25.27 (-20.47 to 71.01)	0.276	36.08 (-12.85 to 85.01)	0.146
Target artery recanalization at 36h, *n* (%)					
None-to-mild (*n* = 49)	40 (83.3)	Ref		Ref	
Moderate (*n* = 35)	30 (88.2)	1.50 (0.41 to 5.45)	0.538	1.17 (0.27 to 5.12)	0.834
Severe (*n* = 14)	11 (78.6)	0.73 (0.17 to 3.24)	0.682	0.52 (0.10 to 2.78)	0.448
**Safety**					

SICH within 48h, *n* (%)					
None-to-mild (*n* = 49)	1 (2.0)	Ref		Ref	
Moderate (*n* = 35)	0 (0.0)	NA	0.955	NA	0.924
Severe (*n* = 14)	1 (7.1)	3.69 (0.22 to 63.12)	0.367	0.03 (0.00 to 332.02)	0.451
Any ICH within 48h, *n* (%)					
None-to-mild (*n* = 49)	14 (28.6)	Ref		Ref	
Moderate (*n* = 35)	12 (34.3)	1.30 (0.51 to 3.32)	0.577	0.99 (0.35 to 2.76)	0.982
Severe (*n* = 14)	5 (35.7)	1.39 (0.40 to 4.88)	0.608	1.02 (0.26 to 3.91)	0.982
Death within 90-day, *n* (%)					
None-to-mild (*n* = 49)	4 (8.2)	Ref		Ref	
Moderate (*n* = 35)	2 (5.7)	0.68 (0.12 to 3.69)	0.652	0.53 (0.09 to 3.21)	0.490
Severe (*n* = 14)	3 (21.4)	2.77 (0.62 to 12.40)	0.182	1.96 (0.37 to 10.29)	0.429
Decompressive hemicraniectomy during hospitalization, *n* (%)					
None-to-mild (*n* = 49)	1 (2.0)	Ref		Ref	
Moderate (*n* = 35)	0 (0.0)	NA	0.955	NA	0.965
Severe (*n* = 14)	1 (7.1)	3.69 (0.22 to 63.12)	0.367	NA	0.303

[a] Data are presented as number (percentage) of patients for categorical values and median (IQR) for continuous or ordinal variables.[b] BG-EPVS burden effects are reported as generalized odds ratio (95% CI) for the ordinal shift across the range of mRS scores toward a better outcome by the ordinal logistic regression model (primary outcome), hazard ratio (95% CI) for death by a Cox proportional hazards regression model, mean difference (95% CI) for ICV change by the general linear model, and odds ratio (OR) with the corresponding 95% CIs for other outcomes by the binary logistic regression models.[c] Model 1: Unadjusted; Model 2: Adjusted for age, sex, baseline National Institutes of Health Stroke Scale (NIHSS) score, Alberta Stroke Program Early CT (ASPECT) score, intravenous thrombolysis, time from onset to randomization. BG-EPVS: enlarged perivascular spaces (EPVS) in the basal ganglia (BG); ENI: early neurological improvement; EVT: endovascular therapy; ICH: intracranial hemorrhage; ICV: infarct core volume; mRS: modified Rankin Scale; NA: not applicable; SICH: symptomatic intracranial hemorrhage.

Supplementary Table S7: EVT treatment effects on study outcomes stratified by BG-EPVS severity, with additional adjustment for other CSVD markers

**Outcome**	**MM group (*n* = 128)** [a]	**EVT group (*n* = 98)** [a]	**Adjusted Effect size (95% CI)** [b, c]	***P* value**	***P* value (Interaction)**
**Primary outcome**					
mRS score, median (IQR)					0.074
None-to-mild (*n* = 136)	4.0 (3.0–5.0)	2.0 (2.0–3.0)	5.35 (2.58 to 11.08)	<0.001	
Moderate (*n* = 60)	4.0 (3.0–5.0)	4.0 (2.0–4.0)	4.47 (1.53 to 13.10)	0.006	
Severe (*n* = 30)	3.5 (2.5–5.5)	4.0 (3.0–5.0)	0.80 (0.13 to 4.93)	0.814	

**Secondary outcomes**					
mRS score 0-2, *n* (%)					0.079
None-to-mild (*n* = 136)	15 (17.2)	28 (57.1)	7.65 (2.96 to 19.79)	<0.001	
Moderate (*n* = 60)	1 (4.0)	12 (34.3)	NA	0.074	
Severe (*n* = 30)	4 (25.0)	3 (21.4)	0.55 (0.02 to 14.37)	0.722	
mRS score 0-3, *n* (%)					0.010
None-to-mild (*n* = 136)	40 (46.0)	39 (79.6)	5.12 (1.92 to 13.65)	0.001	
Moderate (*n* = 60)	9 (36.0)	16 (45.7)	3.51 (0.46 to 26.87)	0.226	
Severe (*n* = 30)	8 (50.0)	4 (28.6)	0.07 (0.00 to 4.24)	0.203	
ENI, *n* (%)					0.024
None-to-mild (*n* = 136)	2 (2.3)	9 (18.4)	8.37 (1.50 to 46.70)	0.015	
Moderate (*n* = 60)	1 (4.2)	2 (5.7)	NA	0.592	
Severe (*n* = 30)	1 (6.3)	0 (0.0)	NA	0.879	
Change in ICV from baseline to follow-up, median (IQR), mL					0.607
None-to-mild (*n* = 136)	90.7 (28.9–141.2)	48.7 (10.9–86.3)	-28.44 (-58.61 to 1.73)	0.065	
Moderate (*n* = 60)	92.8 (37.5–152.4)	50.7 (30.4–98.5)	-33.58 (-78.45 to 11.29)	0.139	
Severe (*n* = 30)	62.7 (22.8–168.3)	63.4 (8.4–149.8)	-11.89 (-98.36 to 74.59)	0.777	
Target artery recanalization at 36h, *n* (%)					0.722
None-to-mild (*n* = 136)	30 (37.0)	40 (83.3)	7.30 (2.73 to 19.52)	<0.001	
Moderate (*n* = 60)	9 (39.1)	30 (88.2)	178.00 (5.77 to >999.999)	0.003	
Severe (*n* = 30)	5 (31.3)	11 (78.6)	NA	0.150	
**Safety**					
SICH within 48h, *n* (%)					0.909
None-to-mild (*n* = 136)	1 (1.2)	1 (2.0)	NA	0.406	
Moderate (*n* = 60)	1 (4.0)	0 (0.0)	NA	0.902	
Severe (*n* = 30)	0 (0.0)	1 (7.1)	NA	0.932	
Any ICH within 48h, *n* (%)					0.672
None-to-mild (*n* = 136)	11 (12.6)	14 (28.6)	1.92 (0.68 to 5.41)	0.216	
Moderate (*n* = 60)	4 (16.0)	12 (34.3)	2.76 (0.53 to 14.27)	0.227	
Severe (*n* = 30)	2 (12.5)	5 (35.7)	75.65 (0.75	0.066	

			to >999.999)		
Death within 90-day, *n* (%)					0.854
None-to-mild (*n* = 136)	9 (10.3)	4 (8.2)	0.96 (0.27 to 3.41)	0.954	
Moderate (*n* = 60)	4 (16.0)	2 (5.7)	0.04 (0.00 to 1.32)	0.071	
Severe (*n* = 30)	4 (25.0)	3 (21.4)	7.07 (0.06 to 894.87)	0.428	
Decompressive hemicraniectomy during hospitalization, *n* (%)					0.939
None-to-mild (*n* = 136)	2 (2.3)	1 (2.0)	0.49 (0.02 to 10.69)	0.651	
Moderate (*n* = 60)	0 (0.0)	0 (0.0)	NA	NA	
Severe (*n* = 30)	0 (0.0)	1 (7.1)	NA	0.979	

[a] Data are presented as number (percentage) of patients for categorical values and median (IQR) for continuous or ordinal variables. [b] Treatment effects are reported as generalized odds ratio (95% CI) for the ordinal shift across the range of mRS scores toward a better outcome by the ordinal logistic regression model (primary outcome), hazard ratio (95% CI) for death by a Cox proportional hazards regression model, mean difference (95% CI) for ICV change by the general linear model, and odds ratio (OR) with the corresponding 95% CIs for other outcomes by the binary logistic regression models. [c] Adjusted for age, sex, baseline National Institutes of Health Stroke Scale (NIHSS) score, ischemic stroke, atrial fibrillation, systolic blood pressure (SBP), deep-white matter hyperintensity, periventricular- white matter hyperintensity and lacunar. CSVD: cerebral small vessel disease; BG-EPVS: enlarged perivascular spaces (EPVS) in the basal ganglia (BG); ENI: early neurological improvement; EVT: endovascular therapy; ICH: intracranial hemorrhage; ICV: infarct core volume; MM: medical management; mRS: modified Rankin Scale; NA: not applicable; SICH: symptomatic intracranial hemorrhage.

Supplementary Table S8: EVT treatment effects on study outcomes stratified by BG-EPVS severity, with additional adjustment for study center, MRI field strength, and slice thickness

Outcome	MM group (*n* = 128) [a]	EVT group (*n* = 98) [a]	Adjusted Effect size (95% CI) [b, c]	*P* value	*P* value (Interaction)
**Primary outcome**					
mRS score, median (IQR)					0.072
None-to-mild (*n* = 136)	4.0 (3.0–5.0)	2.0 (2.0–3.0)	5.70 (2.80 to 11.58)	<0.001	
Moderate (*n* = 60)	4.0 (3.0–5.0)	4.0 (2.0–4.0)	5.02 (1.69 to 14.90)	0.004	
Severe (*n* = 30)	3.5 (2.5–5.5)	4.0 (3.0–5.0)	1.33 (0.26 to 6.92)	0.737	
**Secondary outcomes**					
mRS score 0-2, *n* (%)					0.059

None-to-mild (*n* = 136)	15 (17.2)	28 (57.1)	8.12 (3.29 to 20.06)	<0.001	
Moderate (*n* = 60)	1 (4.0)	12 (34.3)	34.41 (2.76 to 429.41)	0.006	
Severe (*n* = 30)	4 (25.0)	3 (21.4)	NA	0.922	
mRS score 0-3, *n* (%)					0.005
None-to-mild (*n* = 136)	40 (46.0)	39 (79.6)	6.13 (2.35 to 16.02)	<0.001	
Moderate (*n* = 60)	9 (36.0)	16 (45.7)	8.86 (1.12 to 70.22)	0.039	
Severe (*n* = 30)	8 (50.0)	4 (28.6)	0.14 (0.00 to 8.27)	0.349	
ENI, *n* (%)					0.021
None-to-mild (*n* = 136)	2 (2.3)	9 (18.4)	9.16 (1.69 to 49.56)	0.010	
Moderate (*n* = 60)	1 (4.2)	2 (5.7)	NA	0.670	
Severe (*n* = 30)	1 (6.3)	0 (0.0)	NA	0.962	
Change in ICV from baseline to follow-up, median (IQR), mL					0.489
None-to-mild (*n* = 136)	90.7 (28.9–141.2)	48.7 (10.9–86.3)	-32.19 (-60.46 to -3.92)	0.026	
Moderate (*n* = 60)	92.8 (37.5–152.4)	50.7 (30.4–98.5)	-32.45 (-79.14 to 14.24)	0.169	
Severe (*n* = 30)	62.7 (22.8–168.3)	63.4 (8.4–149.8)	22.29 (-56.94 to 101.52)	0.563	
Target artery recanalization at 36h, *n* (%)					0.558
None-to-mild (*n* = 136)	30 (37.0)	40 (83.3)	7.32 (2.92 to 18.36)	<0.001	
Moderate (*n* = 60)	9 (39.1)	30 (88.2)	131.18 (6.65 to >999.999)	0.001	
Severe (*n* = 30)	5 (31.3)	11 (78.6)	NA	0.891	
**Safety**					
SICH within 48h, *n* (%)					0.891
None-to-mild (*n* = 136)	1 (1.2)	1 (2.0)	NA	0.957	
Moderate (*n* = 60)	1 (4.0)	0 (0.0)	NA	0.908	
Severe (*n* = 30)	0 (0.0)	1 (7.1)	NA	0.944	
Any ICH within 48h, *n* (%)					0.828
None-to-mild (*n* = 136)	11 (12.6)	14 (28.6)	2.59 (1.01 to 6.61)	0.047	
Moderate (*n* = 60)	4 (16.0)	12 (34.3)	1.81 (0.42 to 7.88)	0.429	
Severe (*n* = 30)	2 (12.5)	5 (35.7)	13.45 (0.63 to 287.60)	0.096	
Death within 90-day, *n* (%)					0.860
None-to-mild (*n* = 136)	9 (10.3)	4 (8.2)	0.82 (0.22 to 3.08)	0.773	

Moderate (*n* = 60)	4 (16.0)	2 (5.7)	0.17 (0.02 to 1.36)	0.095	
Severe (*n* = 30)	4 (25.0)	3 (21.4)	0.78 (0.12 to 5.12)	0.794	
Decompressive hemicraniectomy during hospitalization, *n* (%)					0.949
None-to-mild (*n* = 136)	2 (2.3)	1 (2.0)	0.19 (0.01 to 8.11)	0.389	
Moderate (*n* = 60)	0 (0.0)	0 (0.0)	NA	NA	
Severe (*n* = 30)	0 (0.0)	1 (7.1)	NA	0.918	

[a] Data are presented as number (percentage) of patients for categorical values and median (IQR) for continuous or ordinal variables. [b] Treatment effects are reported as generalized odds ratio (95% CI) for the ordinal shift across the range of mRS scores toward a better outcome by the ordinal logistic regression model (primary outcome), hazard ratio (95% CI) for death by a Cox proportional hazards regression model, mean difference (95% CI) for ICV change by the general linear model, and odds ratio (OR) with the corresponding 95% CIs for other outcomes by the binary logistic regression models. [c] Adjusted for age, sex, baseline National Institutes of Health Stroke Scale (NIHSS) score, ischemic stroke, atrial fibrillation, systolic blood pressure (SBP), study center, MRI field, and slice thickness. CSVD: cerebral small vessel disease; BG-EPVS: enlarged perivascular spaces (EPVS) in the basal ganglia (BG); ENI: early neurological improvement; EVT: endovascular therapy; ICH: intracranial hemorrhage; ICV: infarct core volume; MM: medical management; mRS: modified Rankin Scale; NA: not applicable; SICH: symptomatic intracranial hemorrhage.

Supplementary Table S9: Relationship between CSO-EPVS severity with functional outcomes and safety endpoints among patients treated with EVT

**Characteristics**		**Model 1: Unadjusted Effect size (95% CI)** [b]	***P* value**	**Model 2: Adjust Effect size (95% CI)** [b, c]	***P* value**
**Primary outcome**					
mRS score, median (IQR)					
None-to-mild (*n* = 25)	2.0 (2.0–4.0)	Ref		Ref	
Moderate (*n* = 39)	3.0 (2.0–4.0)	0.76 (0.31 to 1.85)	0.546	0.75 (0.30 to 1.84)	0.527
Severe (*n* = 32)	3.0 (2.0–4.0)	0.60 (0.24 to 1.52)	0.284	0.54 (0.21 to 1.41)	0.212
**Secondary outcomes**					
mRS score 0-2, *n* (%)					
None-to-mild (*n* = 25)	14 (56.0)	Ref		Ref	
Moderate (*n* = 39)	18 (46.2)	0.67 (0.25 to 1.85)	0.443	0.57 (0.20 to 1.69)	0.314
Severe (*n* = 32)	11 (34.4)	0.41 (0.14 to 1.21)	0.106	0.34 (0.11 to 1.08)	0.068
mRS score 0-3, *n* (%)					
None-to-mild (*n* = 25)	18 (72.0)	Ref		Ref	
Moderate (*n* = 39)	22 (56.4)	0.50 (0.17 to 1.48)	0.212	0.37 (0.10 to 1.28)	0.115
Severe (*n* = 32)	18 (56.3)	0.50 (0.16 to 1.53)	0.225	0.34 (0.09 to 1.26)	0.107
ENI, *n* (%)					
None-to-mild (*n* = 25)	3 (12.0)	Ref		Ref	
Moderate (*n* = 39)	5 (12.8)	1.08 (0.23 to 4.97)	0.923	0.86 (0.17 to 4.38)	0.852
Severe (*n* = 32)	3 (9.4)	0.76 (0.14 to 4.13)	0.749	0.59 (0.09 to 3.66)	0.569

Change in ICV from baseline to follow-up, median (IQR), mL					
None-to-mild (*n* = 25)	51.6 (22.1–75.1)	Ref		Ref	
Moderate (*n* = 39)	40.4 (8.9–95.3)	-8.80 (-46.03 to 28.43)	0.640	-12.16 (-50.38 to 26.06)	0.529
Severe (*n* = 32)	63.4 (34.4–104.7)	16.02 (-22.77 to 54.81)	0.414	11.23 (-29.13 to 51.59)	0.582
Target artery recanalization at 36h, *n* (%)					
None-to-mild (*n* = 25)	22 (91.7)	Ref		Ref	
Moderate (*n* = 39)	35 (89.7)	0.80 (0.13 to 4.71)	0.801	0.69 (0.11 to 4.55)	0.703
Severe (*n* = 32)	24 (75.0)	0.27 (0.05 to 1.43)	0.124	0.22 (0.04 to 1.34)	0.101
**Safety**					
SICH within 48h, *n* (%)					
None-to-mild (*n* = 25)	1 (4.0)	Ref		Ref	
Moderate (*n* = 39)	1 (2.6)	0.63 (0.04 to 10.58)	0.749	0.01 (0.00 to 68.13)	0.326
Severe (*n* = 32)	0 (0.0)	NA	0.953	NA	0.944
Any ICH within 48h, *n* (%)					
None-to-mild (*n* = 25)	9 (36.0)	Ref		Ref	
Moderate (*n* = 39)	12 (30.8)	0.79 (0.27 to 2.29)	0.664	0.81 (0.27 to 2.43)	0.704
Severe (*n* = 32)	9 (28.1)	0.70 (0.23 to 2.14)	0.526	0.68 (0.21 to 2.23)	0.526
Death within 90-day, *n* (%)					
None-to-mild (*n* = 25)	2 (8.0)	Ref		Ref	
Moderate (*n* = 39)	3 (7.7)	0.91 (0.15 to 5.47)	0.922	0.83 (0.13 to 5.23)	0.845
Severe (*n* = 32)	4 (12.5)	1.56 (0.29 to 8.51)	0.609	1.33 (0.21 to 8.44)	0.763
Decompressive hemicraniectomy during hospitalization, *n* (%)					
None-to-mild (*n* = 25)	0 (0.0)	Ref		Ref	
Moderate (*n* = 39)	0 (0.0)	NA	1.000	NA	0.925
Severe (*n* = 32)	2 (6.3)	NA	0.935	NA	0.453

[a] Data are presented as number (percentage) of patients for categorical values and median (IQR) for continuous or ordinal variables. [b] CSO-EPVS burden effects are reported as generalized odds ratio (95% CI) for the ordinal shift across the range of mRS scores toward a better outcome by the ordinal logistic regression model (primary outcome), hazard ratio (95% CI) for death by a Cox proportional hazards regression model, mean difference (95% CI) for ICV change by the general linear model, and odds ratio (OR) with the corresponding 95% CIs for other outcomes by the binary logistic regression models. [c] Model 1: Unadjusted; Model 2: Adjusted for age, sex, baseline National Institutes of Health Stroke Scale (NIHSS) score, Alberta Stroke Program Early CT (ASPECT) score, intravenous thrombolysis, time from onset to randomization. CSO-EPVS: enlarged perivascular spaces (EPVS) in the centrum semiovale (CSO); ENI: early neurological improvement; EVT: endovascular therapy; ICH: intracranial hemorrhage;

ICV: infarct core volume; mRS: modified Rankin Scale; NA: not applicable; SICH: symptomatic intracranial hemorrhage.

Supplementary Table S10: EVT treatment effects on study outcomes stratified by CSO-EPVS severity

Outcome	MM group (*n* = 127) [a]	EVT group (*n* = 96) [a]	Model 1: Unadjusted Effect size (95% CI) [b]	*P* value	*P* value (Interaction)	Model 2: Adjusted Effect size (95% CI) [b, c]	*P* value	*P* value (Interaction)
**Primary outcome**								
mRS score, median (IQR)					0.174			0.232
None-to-mild (*n* = 68)	4 (3–5)	2 (2–4)	5.53 (2.11 to 14.44)	0.001		5.84 (2.03 to 16.82)	0.001	
Moderate (*n* = 89)	3 (3–5)	3 (2–4)	1.99 (0.94 to 4.22)	0.071		3.40 (1.52 to 7.59)	0.003	
Severe (*n* = 66)	4 (3–4)	3 (2–4)	2.19 (0.91 to 5.27)	0.081		3.28 (1.23 to 8.76)	0.018	
**Secondary outcomes**								
mRS score 0-2, *n* (%)					0.492			0.479
None-to-mild (*n* = 68)	7 (16.3)	14 (56.0)	6.55 (2.11 to 20.28)	0.001		8.58 (2.20 to 33.40)	0.002	
Moderate (*n* = 89)	9 (18.0)	18 (46.2)	3.91 (1.50 to 10.17)	0.005		5.48 (1.88 to 16.00)	0.002	
Severe (*n* = 66)	4 (11.8)	11 (34.4)	3.93 (1.10 to 14.03)	0.035		3.76 (0.84 to 16.93)	0.084	
mRS score 0-3, *n* (%)					0.242			0.245
None-to-mild (*n* = 68)	16 (37.2)	18 (72.0)	4.34 (1.49 to 12.65)	0.007		5.21 (1.48 to 18.36)	0.010	

Moderate (*n* = 89)	26 (52.0)	22 (56.4)	1.20 (0.52 to 2.77)	0.679		1.89 (0.70 to 5.13)	0.210	
Severe (*n* = 66)	14 (41.2)	18 (56.3)	1.84 (0.69 to 4.88)	0.223		2.43 (0.73 to 8.06)	0.146	
ENI, *n* (%)					0.072			0.040
None-to-mild (*n* = 68)	0 (0.0)	3 (12.0)	NA	0.946		NA	0.917	
Moderate (*n* = 89)	1 (2.0)	5 (12.8)	7.21 (0.81 to 64.46)	0.077		8.33 (0.85 to 81.75)	0.069	
Severe (*n* = 66)	3 (9.1)	3 (9.4)	1.03 (0.19 to 5.55)	0.968		0.79 (0.11 to 5.91)	0.818	
Change in ICV from baseline to follow-up, median (IQR), mL					0.126			0.196
None-to-mild (*n* = 68)	100.4 (43.3–166.8)	51.6 (22.1–75.1)	-54.26 (-95.32 to -13.21)	0.010		-40.45 (-86.04 to 5.14)	0.080	
Moderate (*n* = 89)	64.5 (16.3–123.2)	40.4 (8.9–95.3)	-26.13 (-56.83 to 4.57)	0.094		-30.00 (-61.75 to 1.75)	0.064	
Severe (*n* = 66)	94.1 (32.9–150.8)	63.4 (34.4–104.7)	-17.01 (-55.19 to 21.16)	0.377		-17.23 (-58.23 to 23.76)	0.403	
Target artery recanalization at 36h, *n* (%)					0.070			0.055
None-to-mild (*n* = 68)	12 (31.6)	22 (91.7)	23.83 (4.81 to 118.16)	<0.001		47.17 (5.06 to 439.54)	0.001	
Moderate (*n* = 89)	20 (40.8)	35 (89.7)	12.69 (3.90 to 41.33)	<0.001		20.57 (4.95 to	<0.001	

						85.51)		
Severe (*n* = 66)	12 (37.5)	24 (75.0)	5.00 (1.71 to 14.63)	0.003		5.31 (1.63 to 17.30)	0.006	
**Safety**								
SICH within 48h, *n* (%)					0.398			0.352
None-to-mild (*n* = 68)	1 (2.3)	1 (4.0)	1.75 (0.11 to 29.27)	0.697		0.84 (0.03 to 27.91)	0.923	
Moderate (*n* = 89)	0 (0.0)	1 (2.6)	NA	0.956		NA	0.920	
Severe (*n* = 66)	1 (2.9)	0 (0.0)	NA	0.960		NA	0.948	
Any ICH within 48h, *n* (%)					0.839			0.788
None-to-mild (*n* = 68)	6 (14.0)	9 (36.0)	3.47 (1.06 to 11.38)	0.040		4.91 (1.16 to 20.85)	0.031	
Moderate (*n* = 89)	7 (14.0)	12 (30.8)	2.73 (0.96 to 7.79)	0.061		2.92 (0.97 to 8.86)	0.058	
Severe (*n* = 66)	4 (11.8)	9 (28.1)	2.94 (0.80 to 10.74)	0.104		2.60 (0.63 to 10.62)	0.185	
Death within 90-day, *n* (%)					0.642			0.794
None-to-mild (*n* = 68)	6 (14.0)	2 (8.0)	0.55 (0.11 to 2.75)	0.470		0.57 (0.08 to 4.13)	0.579	
Moderate (*n* = 89)	6 (12.0)	3 (7.7)	0.61 (0.15 to 2.43)	0.482		0.53 (0.13 to 2.21)	0.383	
Severe (*n* = 66)	5 (14.7)	4 (12.5)	0.82 (0.22 to 3.04)	0.763		0.60 (0.14 to 2.61)	0.499	
Decompressive hemicraniectomy during hospitalization, *n* (%)					0.877			0.847
None-to-mild (*n* = 68)	2 (4.7)	0 (0.0)	NA	0.952		NA	0.911	

Moderate (*n* = 89)	0 (0.0)	0 (0.0)	NA	NA		NA	NA
Severe (*n* = 66)	0 (0.0)	2 (6.3)	NA	0.942		NA	0.836

[a] Data are presented as number (percentage) of patients for categorical values and median (IQR) for continuous or ordinal variables.[b] Treatment effects are reported as generalized odds ratio (95% CI) for the ordinal shift across the range of mRS scores toward a better outcome by the ordinal logistic regression model (primary outcome), hazard ratio (95% CI) for death by a Cox proportional hazards regression model, mean difference (95% CI) for ICV change by the general linear model, and odds ratio (OR) with the corresponding 95% CIs for other outcomes by the binary logistic regression models.[c] Model 1: Unadjusted; Model 2: Adjusted for sex, baseline National Institutes of Health Stroke Scale (NIHSS), hyperlipidemia, atrial fibrillation, ischemic stroke and systolic blood pressure (SBP). CSO-EPVS: enlarged perivascular spaces (EPVS) in the centrum semiovale (CSO); ENI: early neurological improvement; EVT: endovascular therapy; ICH: intracranial hemorrhage; ICV: infarct core volume; MM: medical management; mRS: modified Rankin Scale; NA: not applicable; SICH: symptomatic intracranial hemorrhage.

Supplementary Table S11: EVT treatment effects on study outcomes by CSO-EPVS severity, with additional adjustment for other CSVD markers

**Outcome**	**MM group (*n* = 127)** [a]	**EVT group (*n* = 96)** [a]	**Adjusted Effect size (95% CI)** [b, c]	***P* value**	***P* value (Interaction)**
**Primary outcome**					
mRS score, median (IQR)					0.136
None-to-mild (*n* = 68)	4.0 (3.0–5.0)	2.0 (2.0–4.0)	8.43 (2.73 to 25.96)	<0.001	
Moderate (*n* = 89)	3.0 (3.0–5.0)	3.0 (2.0–4.0)	4.09 (1.74 to 9.60)	0.001	
Severe (*n* = 66)	4.0 (3.0–4.0)	3.0 (2.0–4.0)	3.40 (1.24 to 9.27)	0.017	
**Secondary outcomes**					
mRS score 0-2, *n* (%)					0.372
None-to-mild (*n* = 68)	7 (16.3)	14 (56.0)	9.88 (2.34 to 41.62)	0.002	
Moderate (*n* = 89)	9 (18.0)	18 (46.2)	7.13 (2.18 to 23.30)	0.001	
Severe (*n* = 66)	4 (11.8)	11 (34.4)	3.39 (0.69 to 16.68)	0.133	
mRS score 0-3, *n* (%)					0.224

None-to-mild (*n* = 68)	16 (37.2)	18 (72.0)	6.43 (1.59 to 26.01)	0.009	
Moderate (*n* = 89)	26 (52.0)	22 (56.4)	2.40 (0.79 to 7.33)	0.124	
Severe (*n* = 66)	14 (41.2)	18 (56.3)	2.32 (0.66 to 8.17)	0.189	
ENI, *n* (%)					0.036
None-to-mild (*n* = 68)	0 (0.0)	3 (12.0)	NA	0.719	
Moderate (*n* = 89)	1 (2.0)	5 (12.8)	7.14 (0.68 to 75.06)	0.101	
Severe (*n* = 66)	3 (9.1)	3 (9.4)	1.04 (0.11 to 9.99)	0.971	
Change in ICV from baseline to follow-up, median (IQR), mL					0.233
None-to-mild (*n* = 68)	100.4 (43.3–166.8)	51.6 (22.1–75.1)	-42.86 (-90.44 to 4.73)	0.077	
Moderate (*n* = 89)	64.5 (16.3–123.2)	40.4 (8.9–95.3)	-35.43 (-68.30 to -2.57)	0.035	
Severe (*n* = 66)	94.1 (32.9–150.8)	63.4 (34.4–104.7)	-20.45 (-62.98 to 22.08)	0.339	
Target artery recanalization at 36h, *n* (%)					0.094
None-to-mild (*n* = 68)	12 (31.6)	22 (91.7)	45.89 (4.47 to 471.18)	0.001	
Moderate (*n* = 89)	20 (40.8)	35 (89.7)	49.06 (6.34 to 379.68)	<0.001	
Severe (*n* = 66)	12 (37.5)	24 (75.0)	5.80 (1.71 to 19.68)	0.005	
**Safety**					
SICH within 48h, *n* (%)					0.774
None-to-mild (*n* = 68)	1 (2.3)	1 (4.0)	NA	0.839	
Moderate (*n* = 89)	0 (0.0)	1 (2.6)	NA	0.871	
Severe (*n* = 66)	1 (2.9)	0 (0.0)	NA	0.914	
Any ICH within 48h, *n* (%)					0.971
None-to-mild (*n* = 68)	6 (14.0)	9 (36.0)	5.65 (1.02 to 31.28)	0.048	
Moderate (*n* = 89)	7 (14.0)	12 (30.8)	2.49 (0.77 to 8.00)	0.127	

Severe (*n* = 66)	4 (11.8)	9 (28.1)	2.64 (0.57 to 12.15)	0.213	
Death within 90-day, *n* (%)					0.610
None-to-mild (*n* = 68)	6 (14.0)	2 (8.0)	0.45 (0.05 to 3.85)	0.469	
Moderate (*n* = 89)	6 (12.0)	3 (7.7)	0.31 (0.06 to 1.53)	0.152	
Severe (*n* = 66)	5 (14.7)	4 (12.5)	0.56 (0.12 to 2.68)	0.472	
Decompressive hemicraniectomy during hospitalization, *n* (%)					0.781
None-to-mild (*n* = 68)	2 (4.7)	0 (0.0)	NA	0.876	
Moderate (*n* = 89)	0 (0.0)	0 (0.0)	NA	NA	
Severe (*n* = 66)	0 (0.0)	2 (6.3)	NA	0.707	

[a] Data are presented as number (percentage) of patients for categorical values and median (IQR) for continuous or ordinal variables. [b] Treatment effects are reported as generalized odds ratio (95% CI) for the ordinal shift across the range of mRS scores toward a better outcome by the ordinal logistic regression model (primary outcome), hazard ratio (95% CI) for death by a Cox proportional hazards regression model, mean difference (95% CI) for ICV change by the general linear model, and odds ratio (OR) with the corresponding 95% CIs for other outcomes by the binary logistic regression models. [c] Adjusted for sex, baseline NIHSS, hyperlipidemia, atrial fibrillation, ischemic stroke, systolic blood pressure, deep-white matter hyperintensity, periventricular-white matter hyperintensity and lacune. CSO-EPVS: enlarged perivascular spaces (EPVS) in the centrum semiovale (CSO); CSVD: cerebral small vessel disease; ENI: early neurological improvement; EVT: endovascular therapy; ICH: intracranial hemorrhage; ICV: infarct core volume; mRS: modified Rankin Scale; MM: medical management; NA: not applicable; SICH: symptomatic intracranial hemorrhage.

Supplementary Table S12: EVT treatment effects on study outcomes by CSO-EPVS severity, with additional adjustment for study center, MRI field strength, and slice thickness

**Outcome**	**MM group (*n* = 127)** [a]	**EVT group (*n* = 96)** [a]	**Adjusted Effect size (95% CI)** [b, c]	***P* value**	***P* value (Interaction)**
**Primary outcome**					
mRS score, median					0.263

(IQR)					
None-to-mild (*n* = 68)	4.0 (3.0–5.0)	2.0 (2.0–4.0)	5.85 (2.03 to 16.87)	0.001	
Moderate (*n* = 89)	3.0 (3.0–5.0)	3.0 (2.0–4.0)	3.63 (1.61 to 8.19)	0.002	
Severe (*n* = 66)	4.0 (3.0–4.0)	3.0 (2.0–4.0)	3.17 (1.17 to 8.56)	0.023	
**Secondary outcomes**					
mRS score 0-2, *n* (%)					0.406
None-to-mild (*n* = 68)	7 (16.3)	14 (56.0)	10.21 (2.38 to 43.88)	0.002	
Moderate (*n* = 89)	9 (18.0)	18 (46.2)	5.35 (1.81 to 15.76)	0.002	
Severe (*n* = 66)	4 (11.8)	11 (34.4)	3.76 (0.78 to 18.21)	0.100	
mRS score 0-3, *n* (%)					0.250
None-to-mild (*n* = 68)	16 (37.2)	18 (72.0)	5.43 (1.51 to 19.54)	0.010	
Moderate (*n* = 89)	26 (52.0)	22 (56.4)	1.87 (0.68 to 5.14)	0.223	
Severe (*n* = 66)	14 (41.2)	18 (56.3)	2.51 (0.73 to 8.60)	0.143	
ENI, *n* (%)					0.036
None-to-mild (*n* = 68)	0 (0.0)	3 (12.0)	NA	0.937	
Moderate (*n* = 89)	1 (2.0)	5 (12.8)	8.67 (0.76 to 98.41)	0.081	
Severe (*n* = 66)	3 (9.1)	3 (9.4)	0.61 (0.07 to 5.78)	0.669	
Change in ICV from baseline to follow-up, median (IQR), mL					0.194
None-to-mild (*n* = 68)	100.4 (43.3–166.8)	51.6 (22.1–75.1)	-39.59 (-85.07 to 5.89)	0.087	
Moderate (*n* = 89)	64.5 (16.3–123.2)	40.4 (8.9–95.3)	-29.28 (-61.34 to 2.78)	0.073	
Severe (*n* = 66)	94.1 (32.9–150.8)	63.4 (34.4–104.7)	-17.02 (-58.94 to 24.91)	0.419	
Target artery recanalization at 36h, *n* (%)					0.046
None-to-mild (*n* =	12 (31.6)	22 (91.7)	59.09 (5.62	0.001	

68)			to 621.55)		
Moderate (n = 89)	20 (40.8)	35 (89.7)	25.82 (5.67 to 117.85)	<0.001	
Severe (n = 66)	12 (37.5)	24 (75.0)	5.43 (1.51 to 19.53)	0.010	
**Safety**					
SICH within 48h, n (%)					0.312
None-to-mild (n = 68)	1 (2.3)	1 (4.0)	NA	0.820	
Moderate (n = 89)	0 (0.0)	1 (2.6)	NA	0.500	
Severe (n = 66)	1 (2.9)	0 (0.0)	NA	0.962	
Any ICH within 48h, n (%)					0.718
None-to-mild (n = 68)	6 (14.0)	9 (36.0)	5.19 (1.16 to 23.17)	0.031	
Moderate (n = 89)	7 (14.0)	12 (30.8)	3.28 (1.04 to 10.35)	0.042	
Severe (n = 66)	4 (11.8)	9 (28.1)	2.44 (0.58 to 10.25)	0.224	
Death within 90-day, n (%)					0.650
None-to-mild (n = 68)	6 (14.0)	2 (8.0)	0.51 (0.06 to 4.33)	0.537	
Moderate (n = 89)	6 (12.0)	3 (7.7)	0.43 (0.09 to 2.08)	0.293	
Severe (n = 66)	5 (14.7)	4 (12.5)	0.83 (0.14 to 4.72)	0.829	
Decompressive hemicraniectomy during hospitalization, n (%)					0.750
None-to-mild (n = 68)	2 (4.7)	0 (0.0)	NA	0.885	
Moderate (n = 89)	0 (0.0)	0 (0.0)	NA	NA	
Severe (n = 66)	0 (0.0)	2 (6.3)	NA	0.849	

[a]Data are presented as number (percentage) of patients for categorical values and median (IQR) for continuous or ordinal variables. [b]Treatment effects are reported as generalized odds ratio (95% CI) for the ordinal shift across the range of mRS scores toward a better outcome by the ordinal logistic regression model (primary outcome), hazard ratio (95% CI) for death by a Cox proportional hazards regression model, mean difference (95% CI) for ICV change by the general linear model, and odds ratio (OR) with the corresponding 95% CIs for other outcomes by the binary logistic regression models. [c]Adjusted for sex, baseline NIHSS, hyperlipidemia, atrial fibrillation, ischemic stroke, systolic blood pressure, deep-white matter hyperintensity, periventricular-white matter hyperintensity and lacune. CSO-EPVS*:* enlarged perivascular spaces (EPVS) in the centrum semiovale (CSO); CSVD*:* cerebral small vessel disease; ENI*:* early neurological

improvement; EVT: endovascular therapy; ICH: intracranial hemorrhage; ICV: infarct core volume; mRS: modified Rankin Scale; MM: medical management; NA: not applicable; SICH: symptomatic intracranial hemorrhage.

Supplementary Table S13: Comparison of WMH fazekas scores and lacunes prevalence across BG-EPVS and CSO-EPVS SEVERITY SUBGROups

	**BG-EPVS**			**CSO-EPVS**		
**characteristics**	**None-to-mild (*n* = 136)**	**Moderate (*n* = 60)**	**Severe (*n* = 30)**	**None-to-mild (*n* = 68)**	**Moderate (*n* = 89)**	**Severe (*n* = 66)**
Deep WMH Fazekas score, mean ± SD	0.91 ± 0.59	1.33 ± 0.71	1.47 ± 0.63	0.89 ± 0.59	1.22 ± 0.76	1.15 ± 0.57
Periventricular WMH Fazekas score, mean ± SD	1.23 ± 0.54	1.71 ± 0.73	1.73 ± 0.74	1.17 ± 0.51	1.56 ± 0.75	1.51 ± 0.60
Lacunes, *n* (%)	24/132 (18.2%)	31/58 (53.4%)	9/30 (30%)	12/66 (18.2%)	25/86 (29.1%)	26/65 (40%)

Analyses were performed using complete-case data; no imputation was conducted for missing values. WMH Fazekas and lacunes assessments were performed in participants with T2-FLAIR MRI data available. BG-EPVS: basal ganglia enlarged perivascular spaces; CSO-EPVS: centrum semiovale enlarged perivascular spaces; WMH: white matter hyperintensity; SD: standard deviation.

Supplementary Table S14: Association between BG-EPVS Score and DTI-ALPS index in an independent community-based cohort (PRECISE, *n* = 2219)

**Exposure**	**DTI-ALPS index**			
	**Model 1**		**Model 2**	
	Unadjusted β (95% CI)	*P* value	Adjusted β (95% CI)	*P* value
**BG-EPVS scores** (per 1-point increase)	-0.046 (-0.056 to -0.036)	<0.001	-0.016 (-0.026 to -0.006)	0.002

Model 1: unadjusted; Model 2: adjusted for age, sex, body mass index, and vascular risk factors (hypertension, diabetes mellitus, hypercholesterolemia, previous heart disease, previous stroke, current smoking, and current drinking). BG-EPVS score range 0–4; β represents the change in DTI-ALPS index per 1-point increase in BG-EPVS score. DTI-ALPS is a noninvasive index of glymphatic system function; lower values indicate impaired glymphatic function. BG-EPVS: enlarged perivascular spaces in the basal ganglia; DTI-ALPS: diffusion tensor image analysis along the perivascular space; CI: confidence interval.

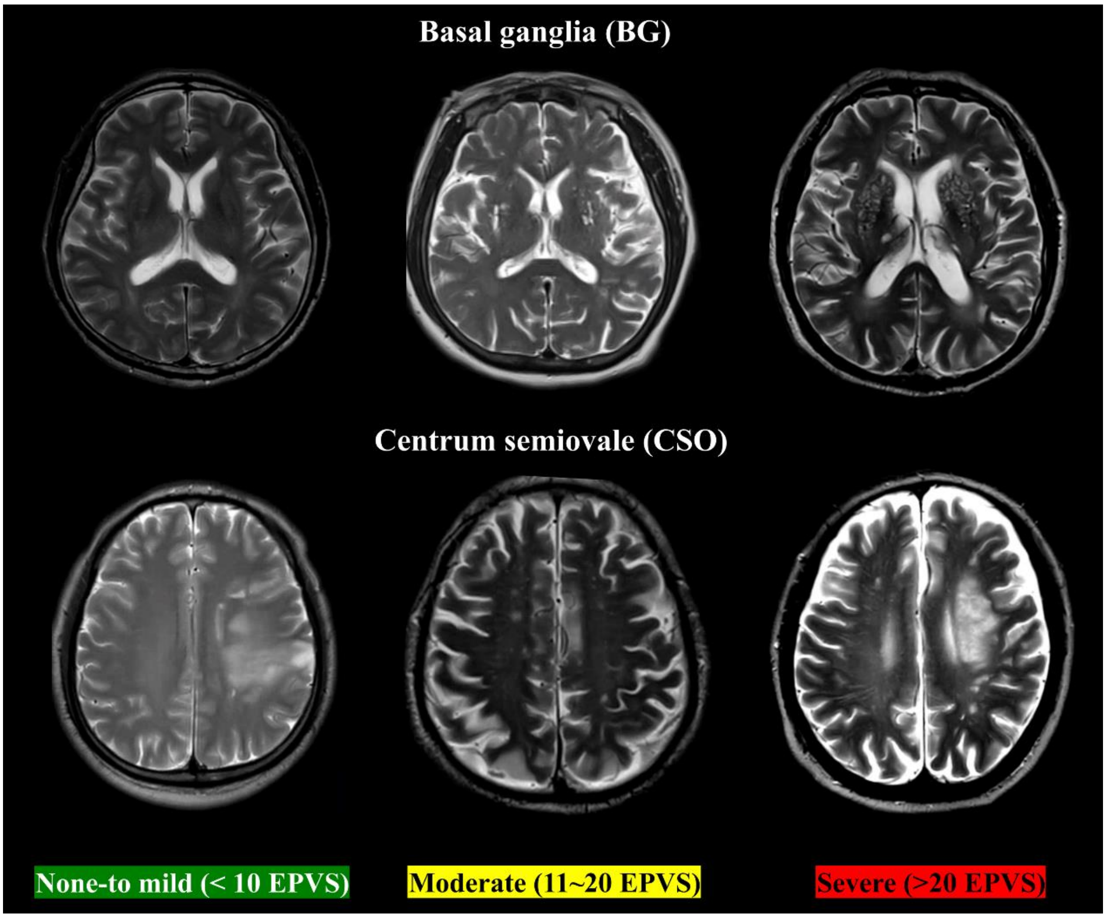

Supplementary Figure S1: Classification of basal ganglia (BG) and centrum semiovale (CSO) EPVS severity on T2-weighted MRI: none-to-mild, moderate, and severe. EPVS: enlarged perivascular spaces.

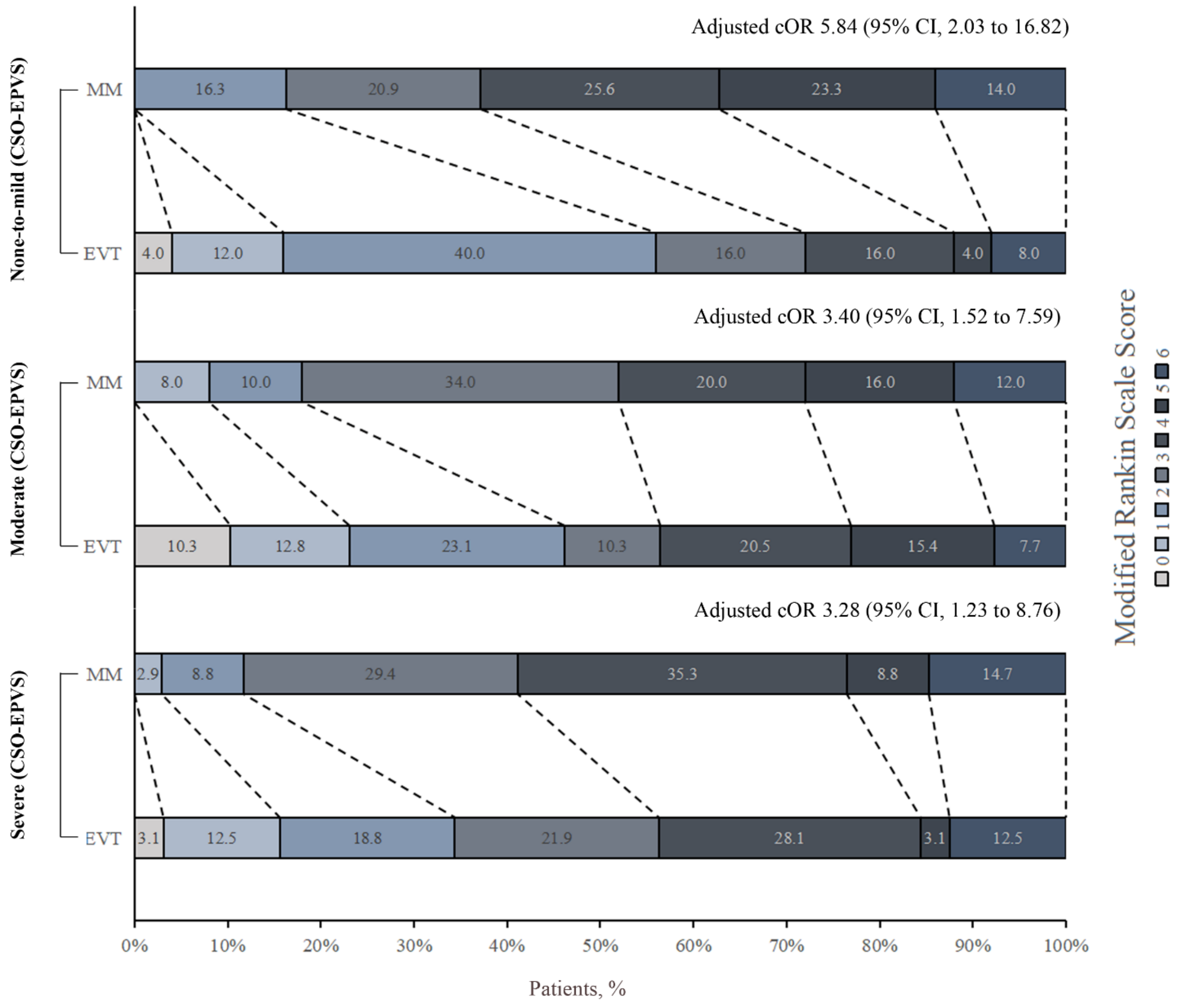

Supplementary Figure S2: Modified Rankin scale score distribution stratified by CSO-EPVS severity. CSO-EPVS: enlarged perivascular spaces in the centrum semiovale; EVT: endovascular therapy; MM: medical management.

Supplementary Table S15: List of participating sites and investigators in ANGEL-ASPECT

Site PIs	Department	Hospital
Zhongrong Miao	Department of Interventional Neuroradiology	Beijing Tiantan Hospital, Capital Medical University
Guangxiong Yuan	Department of Emergency	Xiangtan Central Hospital
Hongxing Han	Department of Neurology	Linyi People's Hospital
Wenhuo Chen	Department of Neurology	Zhangzhou Affiliated Hospital of Fujian Medical University
Ming Wei	Department of	Tianjin huanhu hospital

	Neurosurgery	
Jiangang Zhang	Department of Neurology	Anyang People's Hospital
Zhiming Zhou	Department of Neurology	Yijishan Hospital of Wannan Medical College
Xiaoxi Yao	Department of Neurology	The first people's hospital of Chenzhou
Guoqing Wang	Department of Neurology	Bin zhou People's Hospital
Weigen Song	Department of Neurology	Yancheng Third People's Hospital
Xueli Cai	Department of Neurology	Lishui Municipal Central Hospital
Guangxian Nan	Department of Neurology	China-Japan Union Hospital of Jilin University
Di Li	Department of Neurointervention	Dalian Municipal Central Hospital affiliated with Dalian Medical University
Alvin Yi-Chou Wang	Department of Neurosurgery	Guangdong Provincial Hospital of Chinese Medicine
Wentong Ling	Department of Neurology	ZhongShan City People's Hospital
Chuwei Cai	Department of Neurology	Shantou Central Hospital
Changming Wen	Department of Neurology	Nanyang Central Hospital
En Wang	Department of Neurology	Taizhou hospital of Zhejiang Province
Liyong Zhang	Department of Neurosurgery	Liaocheng People's Hospital
Changchun Jiang	Department of Neurology	Baotou Centre Hospital
Yajie Liu	Department of Neurology	Shenzhen Hospital, Southern Medical University
Geng Liao	Department of Neurology	Maoming People's Hospital
Xiaohui Chen	Department of Neurology	The Second Affiliated Hospital of GuangZhou Medical University
Tianxiao Li	Department of Cerebrovascular Disease	Henan Provincial People's Hospital, Zhengzhou University
Shudong Liu	Department of Neurology	Yongchuan Hospital of Chongqing Medical University
Jinglun Li	Department of Neurology	The affiliated hospital of South West medical university
Yaxuan Sun	Department of Neurology	Shanxi Provincial People's Hospital
Na Xu	Department of Neurology	The Second Affiliated Hospital to Xiamen Medical College
Zong'en Gao	Department of Neurology	Shengli Oilfield Central Hospital
Dongsheng Ju	Department of Neurology	Songyuan Jilin oil Field Hospital

Cunfeng Song	Department of Interventional Neuroradiology	Liao Cheng the third people's hospital
Jinggang Xuan	Department of Neurology	The First People's Hospital of Changzhou
Feng Zhou	Department of Neurology	Taiyuan Central Hospital
Qing Shi	Department of Neurology	Affiliated Jiangmen Traditional Chinese Medicine Hospital of Ji'nan University
Jun Luo	Department of Neurology	Sichuan Mianyang 404 Hospital
Yan Liu	Department of Neurology	JingJiang People's Hospital, the Seventh Affiliated Hospital of Yangzhou University
Zaiyu Guo	Department of Neurosurgery	Tianjin TEDA Hospital
Tong Li	Department of Neurosurgery	The second Nanning People's Hospital
Hongbo Zheng	Department of Neurology	West China Hospital, Sichuan University
Linzhi Dai	Department of Neurosurgery	First Affiliated Hospital School of Medicine Shihezi University
Junfeng Zhao	Department of Neurology	Siping Central People's Hospital
Liqiang Gui	Emergency and Critical Stroke Ambulance Centre	Langfang Changzheng Hospital
Xiaokun Geng	Department of Neurology	Beijing Luhe Hospital, Capital Medical University
Yufeng Tang	Department of Neurology	Mianyang Central Hospital
Congguo Yin	Department of Neurology	Hangzhou First People's Hospital
Hua Yang	Department of Neurosurgery	The affiliated Hospital of Guizhou Medical University
